# Copper-Catalyzed Selective Amino-alkoxycarbonylation of Unactivated Alkenes with CO

**DOI:** 10.1021/jacs.4c13723

**Published:** 2025-02-17

**Authors:** Si-Shun Yan, Ralf Jackstell, Matthias Beller

**Affiliations:** † Leibniz-Institut für Katalyse e.V. an der Universität Rostock, Albert-Einstein-Straße 29a, Rostock 18059, Germany

## Abstract

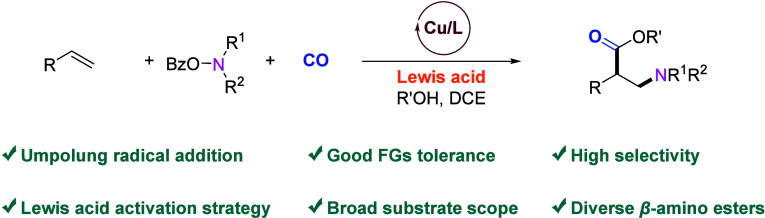

1,2-Amino-difunctionalization reactions of alkenes allow the efficient introduction of different functional groups and the rapid construction of valuable functionalized amines. In this respect, we report a copper-catalyzed 1,2-amino-alkoxycarbonylation of unactivated alkenes with CO and alkylamine precursors in the presence of a Lewis acid additive. The novel protocol allows direct access to valuable β-amino acid derivatives from easily available starting materials. The presented methods feature high chemo- and regioselectivities, good functional group tolerance, and substrate scope including diverse bioactive compounds and drug-like molecules. Mechanistic studies indicate that the Lewis acid additive is the key to realizing the efficient umpolung addition of nucleophilic aminyl radicals to electron-rich alkenes, which represents an elegant activation strategy for aminyl radicals.

## Introduction

**Figure 1:**
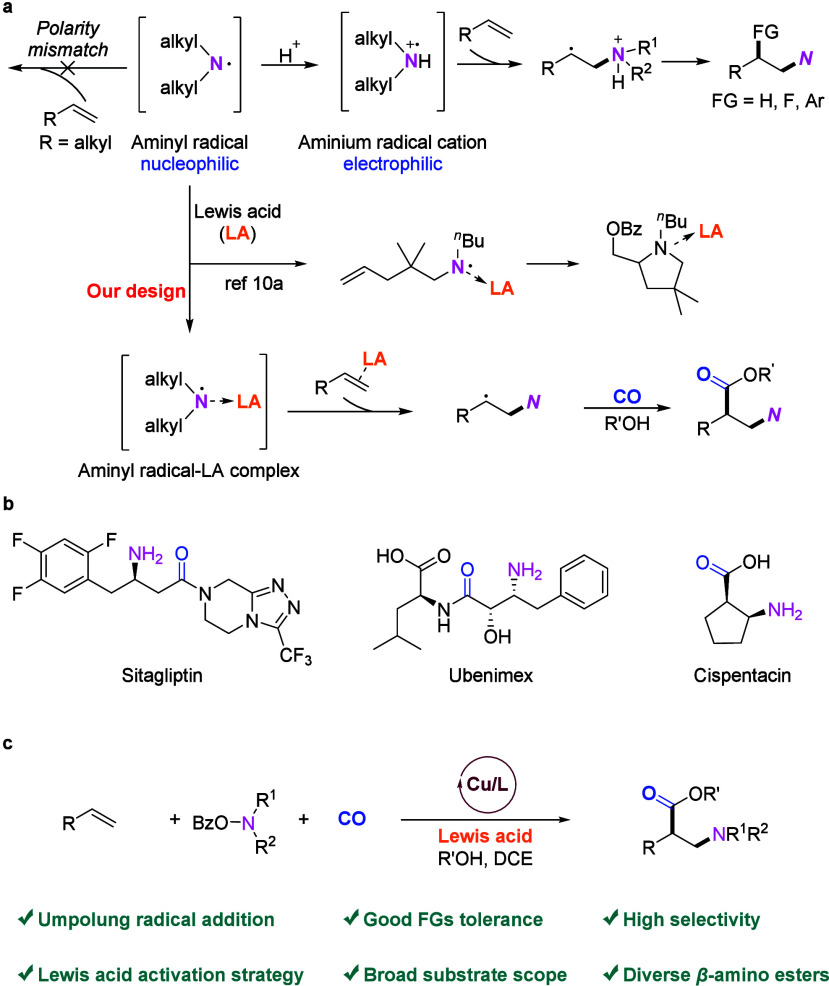
Selected β-amino acid derivatives and strategies for their synthesis. (a) Radical addition of aminyl radical to alkenes. (b) β-Amino acid derivatives in important drugs and bioactive molecules. (c) This work: copper catalyzed 1,2-amino-alkoxycarbonylation of alkenes with CO.

The simultaneous introduction of two or more functional groups into a given substrate not only allows the desired organic synthesis to be carried out in a time- and step-saving manner, but also provides a valuable tool for the rapid construction of complex molecules.^1^ In this respect, 1,2-aminodifunctionalization reactions of alkenes are very attractive due to the wide availability of substrates and the resulting structural motif that can be found in a variety of biologically active compounds.^2^ A classic example of such methodologies is the Os-catalyzed aminohydroxylation of alkenes allowing the straightforward synthesis of 1,2-amino-alcohols.^3^ In addition to such traditional organometallic catalysis processes, aminodifunctionalizations of alkenes via nitrogen-centered radicals (NCRs) have become a powerful strategy for the synthesis of functionalized amines over the past decade.^4^ A number of interesting reaction types have been developed, such as carboamination,^5^ oxyamination,^6^ aminofluorination^7^ and diamination.^8^ In most of these cases, an electrophilic amidyl radical undergoes efficient radical addition to electron-rich alkenes, while nucleophilic iminyl radicals allow the addition to electron-poor alkenes. Therefore, most known examples of such 1,2-amino difunctionalizations of alkenes are based on these two types of radicals. Aminyl radicals are weakly nucleophilic, so that addition to electron-rich alkenes is slow and reversible.^2b,9^ This in turn means that the few known examples are generally limited to intramolecular cyclization reactions.^10^ In 2002, the Göttlich group reported an interesting copper-catalyzed cyclization of unsaturated N-benzoyloxyamines via an aminyl radical activated by a Lewis acid (Figure 1a).^10a^ For more synthetically interesting intermolecular reactions, the current solution is to convert the aminyl radical by protonation into a more electrophilic aminium radical cation, which then undergoes simpler radical addition to alkenes (Figure 1a). Using this strategy, elegant transformations such as hydroamination, aminofluorination and aminoheteroarylation of alkenes have been realized by Knowles, Wang, Fu and other groups.^11^ Despite this progress, there are still no general methods for the direct umpolung addition of nucleophilic aminyl radicals to nonactivated alkenes.

In general, the concurrent addition of carboxyl and amino groups provides the opportunity to prepare β-amino acids.^12^ This class of compounds constitutes core components of many current drugs and biologically active peptides and therefore finds extensive applications in life sciences, specifically in the pharmaceutical industry (Figure 1b). In the last decades, several catalytic methods have been reported for the facile synthesis of β-amino acids and their derivatives, such as hydrogenation of β-carboxylic enamides,^13^ and amination of *α,β*-unsaturated carboxylic acids or esters.^14^ Most of these strategies employ prefunctionalized substrates, which require additional reaction steps and obviously limit the step-efficiency. Clearly, the development of direct carboamination methodologies from easily available starting materials is very attractive for many chemists in academia and industry. As an example, in 2015 the Liu group realized a palladium-catalyzed aminocarbonylation of alkenes to generate β-amino acid derivatives.^15^ Here, stoichiometric amounts of a hypervalent iodine reagent were used to accelerate the intermolecular aminopalladation step. More recently, Yu and co-workers reported an elegant strategy to realize the aminocarboxylation of alkenes with CO_2_.^16^ Using synergistic photocatalysis and copper catalysis, diverse styrenes and acrylates provided the corresponding β-amino acids under mild conditions; however, simple aliphatic alkenes failed to give the desired products due to their lower reactivity. To overcome all these limitations, we became interested in the 1,2-amino-difunctionalization of such demanding substrates. As a result, here we propose a concept for straightforward 1,2-amino-alkoxycarbonylation of unactivated aliphatic alkenes based on a nucleophilic aminyl radical for the first time (Figure 1a). The resulting *N*-alkyl-β-amino ester is of interest for various applications.

Crucial for the success of the desired methodology would be overcoming the polarity mismatch between the nucleophilic aminyl radical and the unactivated alkenes. Moreover, the intrinsic preference of aminyl radicals for H atom abstraction rather than addition to alkenes must be overcome. Considering that Lewis acids can activate the aminyl radical and facilitate the intramolecular cyclization of aminyl radicals onto alkenes,^10a,17,18^ we envisioned that complexes generated from aminyl radicals and Lewis acids might undergo radical addition to unactivated alkenes to provide the corresponding carbon radical intermediates. Subsequent alkoxycarbonylation with CO and alcohols should, in principle, provide valuable β-amino esters (Figure 1c). Notably, several challenges need to be addressed to realize this process. The aminyl radical-Lewis acid complex might impose a high steric barrier in the transition state of the radical addition step. Moreover, the rate of intermolecular radical additions is usually significantly slower compared to intramolecular reactions. In addition, the 1,2-aminooxygenation products might be generated if the CO insertion step is not fast enough. Nevertheless, all these problems can be overcome combining a suitable radical generation system with CO in the presence of Lewis acids. Detailed control experiments demonstrate that this additive is crucial to realize the intermolecular addition of aminyl radical to alkenes.

## Results and Discussion

To realize the desired 1,2-amino-methoxycarbonylation of nonactivated olefins, we investigated the reaction of 1-octene (**1a**) with CO and methanol using *O*-benzoylhydroxylamine (**2a**) as an alkylamine precursor to give **3a** (Table 1). Based on literature precedents,^19^ we planned to generate the corresponding aminyl radicals using a copper catalyst in the presence of 4,4′-dimethoxy-2,2′-bipyridine. However, even applying a high pressure of CO (60 bar), only little conversion of **1a** was observed and trace amounts of **3a** were detected when using copper(II) triflate as catalyst (Table 1, entries 1–3). Following our concept of increasing the reactivity of the piperidinyl radical, the model reaction was performed in the presence of several Lewis acids and different Lewis acid concentrations (Table 1, entries 4–12; Table S1). Indeed, 1-octene underwent 1,2-amino-methoxycarbonylation reaction with **2a** and CO, giving **3a** in 33% yield in the presence of 1.5 equiv of LiBF_4_, which demonstrates the beneficial effect of such an additive in this reaction. Further detailed screening of the type of Lewis acid additive showed that AgBF_4_ provided the best results, and the corresponding β-amino ester **3a** was obtained in 77% GC yield. Lowering the amount of AgBF_4_ to 1.2 equiv slightly increased the yield of **3a** to 79% (Table 1, entry 12). Under these conditions, a trace amount of the anhydride product (**3a′′**) could also be detected. Reducing the catalyst loading to 10 mol % gave a similar result (Table 1, entry 13). Finally, a series of control experiments were performed. Without copper, no conversion of **1a** and **2a** occurred, which indicated that this transformation is a copper-catalyzed reaction (Table 1, entry 14). In the absence of any ligand, **3a** was obtained only in 5% yield, while a 47% yield of 1,2-amino-methoxy product (**3a′**) was generated, which indicates that the ligand plays an important role in the CO insertion step (Table 1, entry 15). Further control experiments proved the necessity of carbon monoxide, demonstrating that the carbonyl source of the β-amino ester comes from CO gas (Table 1, entry 16).

**Table 1:**
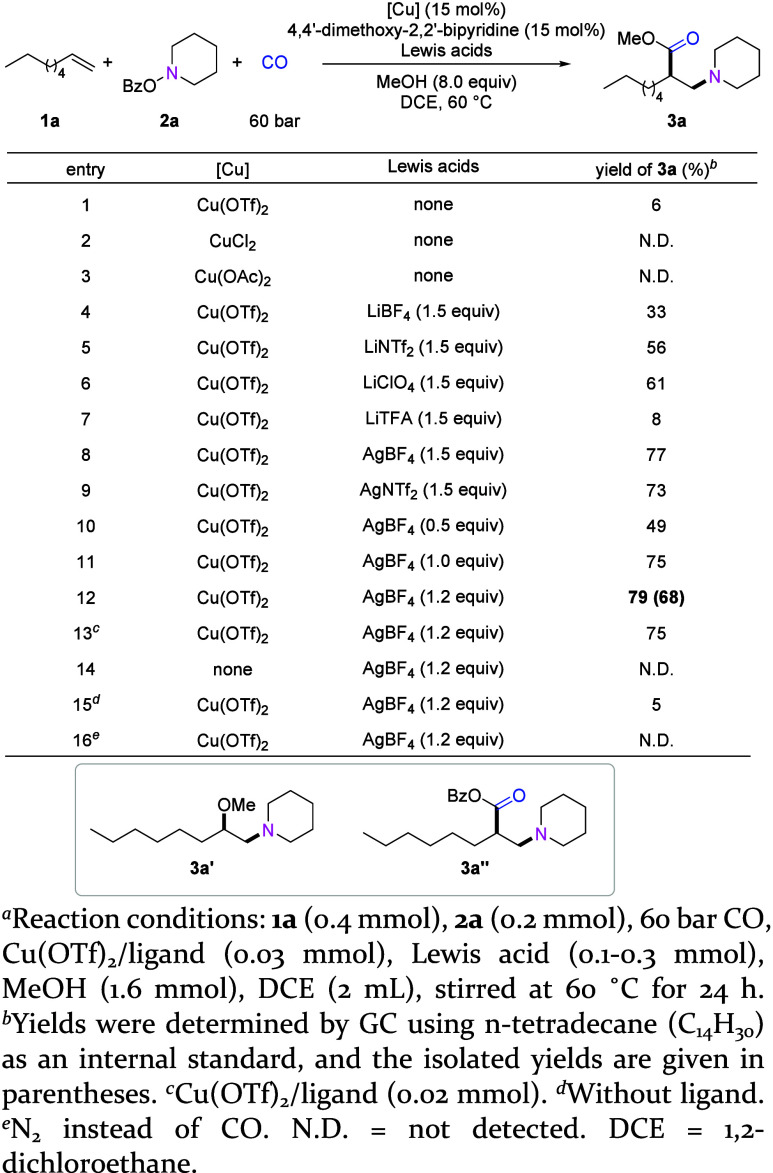
1,2-Amino-methoxycarbonylation of 1-Octene: Variations of Reaction Conditions^a^

**Figure 2:**
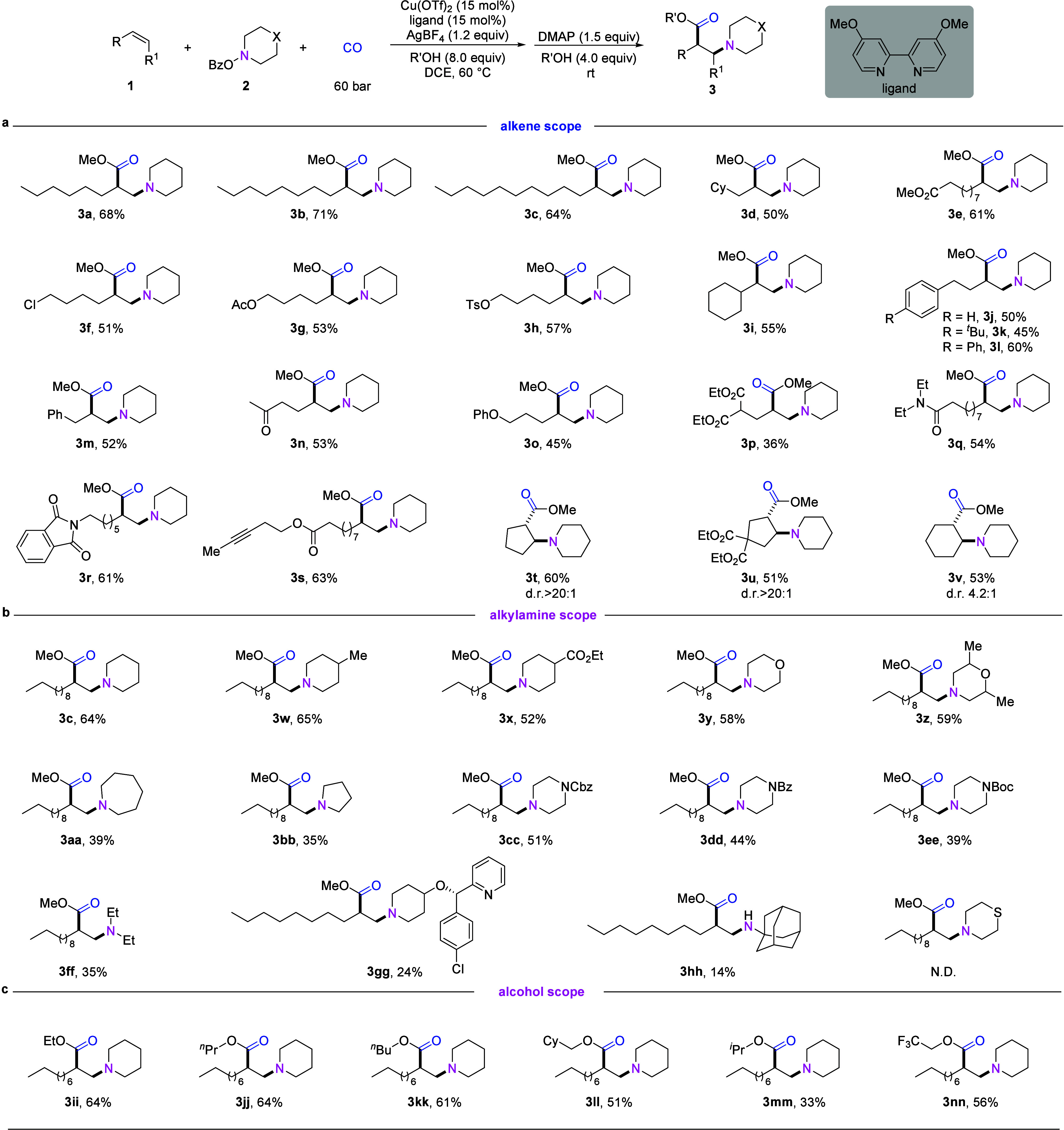
Substrate scope of nonactivited alkenes and alkylamines. Standard reaction conditions: **1** (0.4 mmol), **2** (0.2 mmol), 60 bar of CO, Cu(OTf)_2_/ligand (0.03 mmol), AgBF_4_ (0.24 mmol), R′OH (1.6 mmol), DCE (2 mL), stirred at 60 °C for 24 h. Then, DMAP (0.3 mmol) and R′OH (0.8 mmol) were added, and the mixture was stirred at room temperature for 4 h. Isolated yields are shown.

With the optimal reaction conditions in hand, we first investigated the scope of diverse nonactivated alkenes. As shown in Figure 2a, a variety of aliphatic alkenes with different chain lengths underwent smooth 1,2-amino-methoxycarbonylation reaction to provide valuable β-amino esters in moderate to good yields. To transform the few amounts of formed β-amino anhydride to β-amino ester and to make the purification step easier, DMAP and MeOH were added in the workup procedure. Simple “fatty” alkenes such as 1-decene and 1-dodecene gave the desired products in high yields (**3b**, **3c**). Aliphatic alkenes containing various functional groups, such as ester (**3e**, **3g**, and **3p**), chloro (**3f**), alkyl sulfonate (**3h**), ketone (**3n**), and ether (**3o**), were all applicable to the reaction, producing diverse β-amino esters in one step. Cyclohexyl- (**3d**, **3i**) or phenyl- (**3j**–**3m**) substituted alkenes also provided the desired products. Alkenes containing an amide (**3q**), phthalimide (**3r**) or alkyne (**3s**) group underwent this reaction smoothly, too, which provides opportunities for further transformations. Regarding regioselectivity, all the desired products from terminal alkenes were exclusively 1,2-amino esters, showing the excellent selectivity of this reaction. Besides terminal alkenes, internal alkenes such as cyclopentene and cyclohexene also showed reactivity, giving the corresponding β-amino ester in moderate yield and good diastereoselectivity (**3t**–**3v**). Linear internal alkenes such as trans-2-octene underwent the desired transformation; however, mixtures of regio- and diastereomers were obtained (please see Supporting Information for more details).

Next, we tested the reactivity of various alkylamines with 1-dodecene under optimal reaction conditions (Figure 2b). The reactions of several piperidine-containing aminating reagents provided the desired β-amino esters in good yields (**3c**, **3w**, and **3x**). Morpholine derivatives could also be used in this reaction to give the corresponding products in 58–59% yield (**3y**, **3z**). Both seven- and five-membered cyclic amine precursors participated in this reaction (**3aa**, **3bb**), although the yield decreased with the formation of allylamine or enamine byproducts via β-H elimination. Different functional groups substituted piperazine-derived precursors were also effective, such as *N*-Cbz, *N*-Bz, *N*-Boc, providing a series of valuable β-amino esters in 39–51% yields (**3cc**–**3ee**). An acyclic amine precursor derived from diethylamine underwent this transformation to generate the desired product in 35% isolated yield (**3ff**). Additionally, the amine precursor derived from a bepotastine intermediate was applicable to the reaction, producing the respective β-amino ester in one step (**3gg**). Notably, the primary amine precursor could also provide the desired product (**3hh**), although the yield is low due to the low conversion of primary amine precursor. We also tested the reaction of thiomorpholine precursor; however, in this case no product was obtained. This protocol is applicable to other alcohols as well. As shown in Figure 2c, different primary alcohols reacted efficiently to provide the corresponding β-amino esters in good yields (**3ii**–**3ll**), while secondary alcohol gave a lower yield which might be caused by steric hindrance (**3mm**). To our delight, trifluoroethanol can also be used as a nucleophilic reagent, delivering the desired β-amino ester in a good yield (**3nn**).

**Figure 3:**
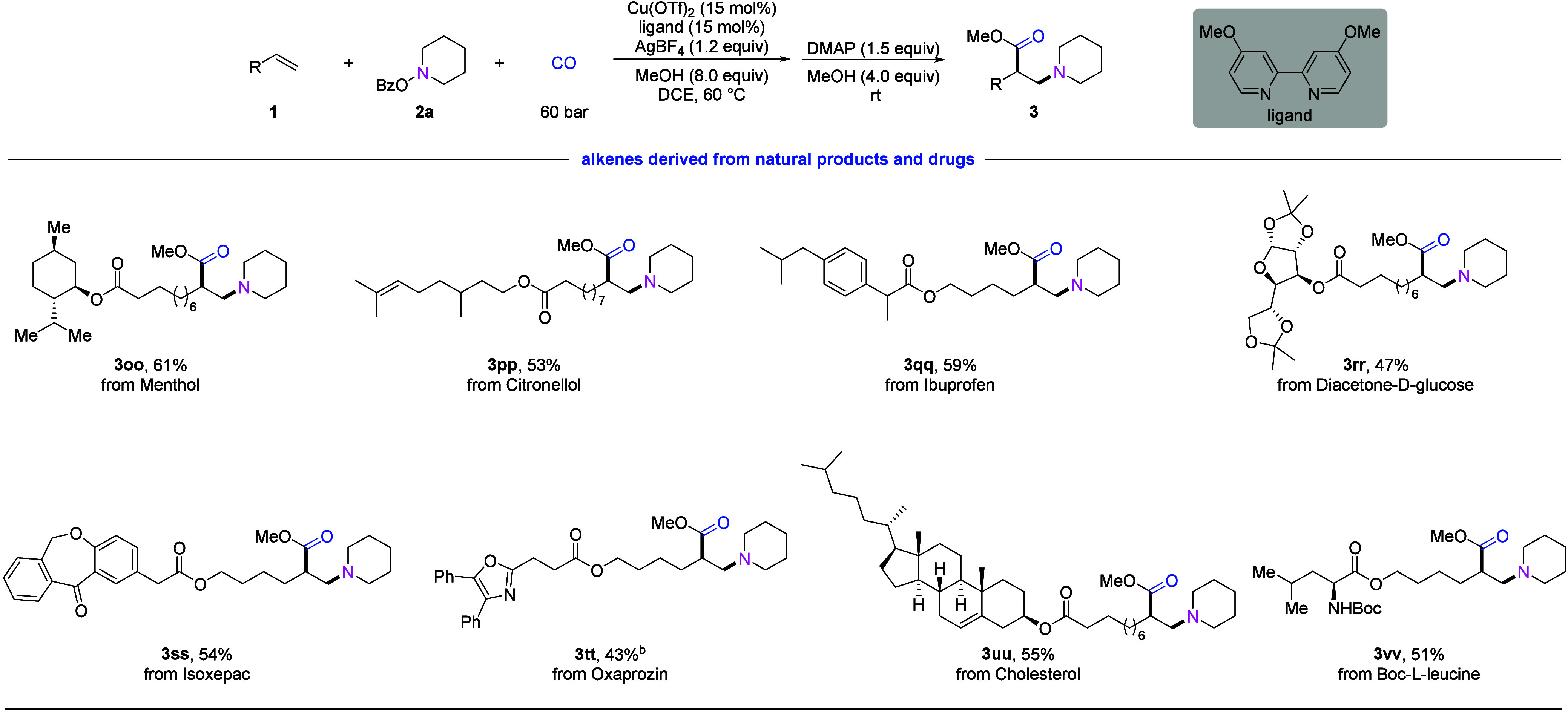
Selected substrate scope of alkenes derived from natural products and drugs.^a^^a^Standard reaction conditions: **1** (0.4 mmol), **2a** (0.2 mmol), 60 bar of CO, Cu(OTf)_2_/ligand (0.03 mmol), AgBF_4_ (0.24 mmol), MeOH (1.6 mmol), and DCE (2 mL), stirred at 60 °C for 24 h. Then, DMAP (0.3 mmol) and MeOH (0.8 mmol) were added, and the mixture was stirred at room temperature for 4 h. Isolated yields are shown. ^b^Alkene **1tt** (0.2 mmol), **2a** (0.24 mmol).

To showcase the functional group tolerance of this novel synthetic procedure, we set out to explore the generality of this protocol for the late-stage modification of different complex molecules (Figure 3). Alkenes derived from natural products such as menthol (**3oo**), citronellol (**3pp**), and cholesterol (**3uu**), as well as pharmaceutical compounds such as Ibuprofen (**3qq**), Isoxepac(**3ss**), and Oxaprozin (**3tt**), can be used in this transformation smoothly, providing advanced β-amino esters in moderate to good yields. In addition, alkenes derived from carbohydrate (**3rr**) and α-amino acid (**3vv**) showed good reactivity and furnished the corresponding β-amino esters. These results indicate the potential of this method for late-stage modifications of bioactive compounds.

**Figure 4:**
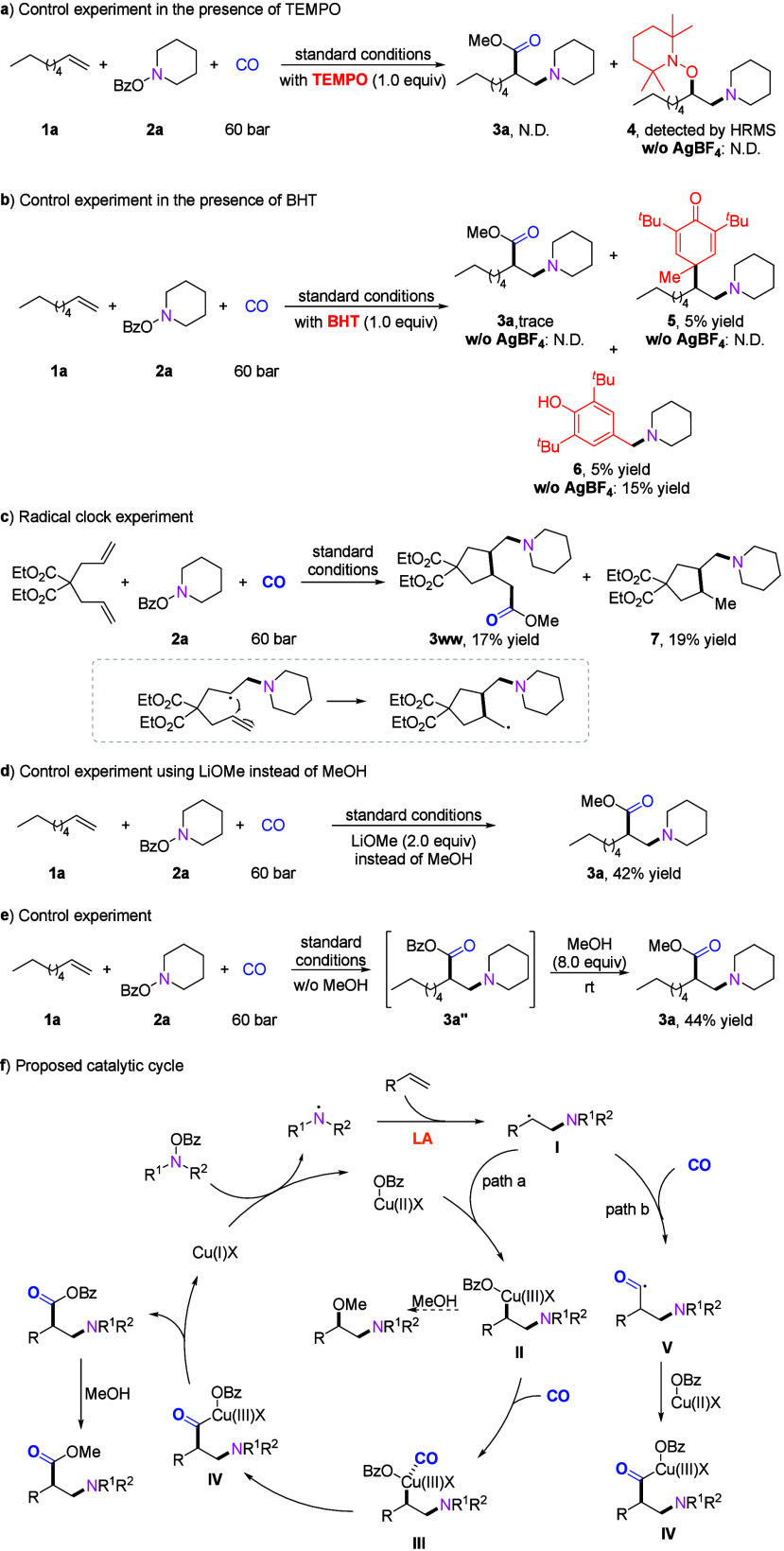
Control experiments and mechanistic studies. Standard reaction conditions: **1** (0.4 mmol), **2a** (0.2 mmol), 60 bar CO, Cu(OTf)_2_/ligand (0.03 mmol), AgBF_4_ (0.24 mmol), MeOH (1.6 mmol), DCE (2 mL), stirred at 60 °C for 24 h. Isolated yields are shown.

To gain insights into the mechanism of the reaction and understand the crucial role of the Lewis acid, a range of mechanistic experiments were carried out (Figure 4). When radical scavenger TEMPO was added under the standard conditions, the reaction was suppressed, and no 1,2-amino-methoxycarbonylation product could be detected. Notably, the corresponding radical trapping adduct **4** was only detected in the presence of AgBF_4_ (Figure 4a), indicating the essential role of Lewis acid for the addition of the aminyl radical to the unactivated alkenes to generate the corresponding carbon radical. The addition of 1 equiv of butylated hydroxytoluene (BHT) to the reaction of **1a** led only to trace amounts of **3a** while two BHT adducts **5** and **6** were detected, suggesting the formation of carbon radical and aminyl radical intermediate (Figure 4b). In the absence of AgBF_4_, only BHT adduct **6** could be generated, demonstrating again that the Lewis acid is necessary for the aminyl radical addition step but not for the generation of the aminyl radical. Next, a radical clock experiment further indicated that this reaction proceeds via a radical pathway (Figure 4c). When using LiOMe instead of MeOH under the standard conditions, 42% yield of **3a** could also be obtained, which demonstrated that MeOH mainly acts as a nucleophile and a proton is not necessary for this reaction (Figure 4d). Finally, we tried a two-step, one-pot transformation, which also provided β-amino ester **3a** in 44% yields (Figure 4e). The β-amino anhydride (**3a′′**) was detected by HRMS in the first step, which implied that β-amino anhydride might be the intermediate (please see Supporting Information for more details).

Based on all the experimental results and literature precedents,^20^ we propose the following possible catalytic cycle (Figure 4f). After in situ generation of Cu(I) species, reaction of the N-radical precursors gives an aminyl radical and a Cu(II) intermediate. In the presence of Lewis acid, the aminyl radical undergoes umpolung radical addition to unactivated alkenes to afford a carbon-centered radical species **I**. This carbon radical might recombine with Cu(II) species to form alkylcopper(III) intermediate **II** (path a). Subsequent coordination and insertion of CO will give carbonylative Cu(III) species **IV**. The CO insertion step might also be accelerated by Lewis acids. The resulting carbonylative Cu(III) species **IV** undergoes reductive elimination to provide the β-amino anhydride and regenerate the Cu(I) catalyst. The anhydride will react with excess MeOH to provide the desired β-amino esters. The carbon radical species **I** might react with CO to furnish acyl radical **V**,^21^ which could also recombine with Cu(II) species to generate intermediate **IV** (path b). If the CO insertion step is not efficient, alkylcopper(III) intermediate **II** would undergo ligand exchange with methanol and finally provide the 1,2-amino-methoxy byproduct. Alkylcopper(III) intermediate **II** might also undergo β-H elimination to give the respective allylamine or enamine as byproduct.^22^

**Figure 5:**
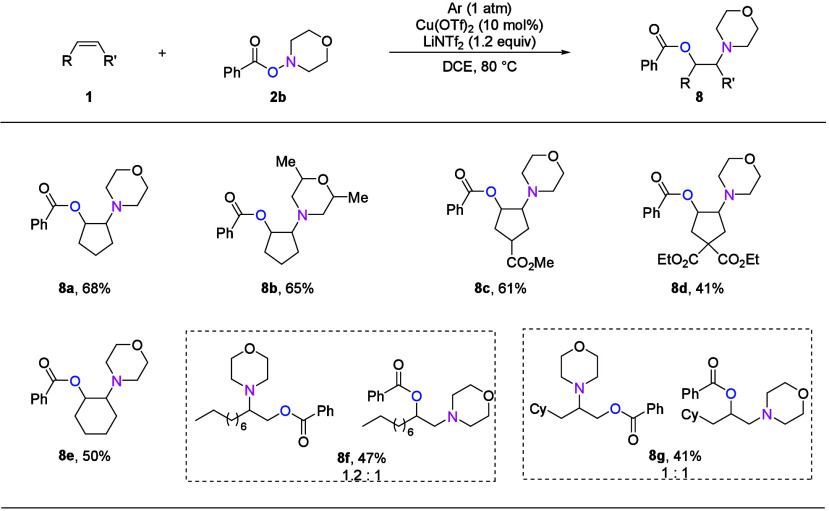
Copper catalyzed acyloxy-amination of nonactivated alkenes. Standard reaction conditions: **1** (0.2 mmol), **2b** (0.3 mmol), Cu(OTf)_2_ (0.02 mmol), LiNTf_2_ (0.24 mmol), and DCE (2 mL), stirred at 80 °C for 20 h. Isolated yields are shown.

After realizing the copper catalyzed 1,2-amino-alkoxycarbonylation of alkenes with CO, we further explored other types of amino-difunctionalization reactions following our general activation concept. Indeed, a copper catalyzed acyloxy-amination of unactivated alkenes could be realized using morpholino benzoate (**2b**) as the source of both aminyl radical and nucleophile. This simultaneous use has obvious advantages regarding atom economy (Figure 5). In this reaction, LiNTf_2_ performed best as a Lewis acid additive, and various β-amino alcohol derivatives were obtained in moderate yields. Using cycloalkenes, the desired products were obtained with good selectivity. However, when applying terminal alkenes, a mixture of two β-amino alcohol derivatives with opposite regioselectivities is generated. This is different from the 1,2-amino-methoxycarbonylation reaction, which might be related to whether the ligand was added. However, when 2,2′-bipyridine type ligands were added to the acyloxy-amination reaction, the yield of the β-amino alcohol product decreased.

## Conclusion

In conclusion, we have developed a copper catalyzed 1,2-amino-alkoxycarbonylation of nonactivated alkenes with CO and alkylamines, which represents a straightforward method to generate valuable β-amino acid derivatives from simple alkenes in one step. A variety of aliphatic alkenes underwent this reaction with high chemo- and regioselectivities. From an application perspective, this protocol can be used for late-stage modifications of complex molecules. Mechanistic studies indicate the Lewis acid additive is the key to realize the efficient addition of aminyl radicals to nonactivated alkenes, which represents a useful activation strategy of aminyl radicals. Using this strategy, we also realized a novel copper catalyzed acyloxy-amination of aliphatic alkenes to provide interesting β-amino alcohol derivatives. This indicates the generality of the proposed activation strategy of aminyl radicals, which could be further employed for realizing other types of 1,2-amino-difunctionalization of alkenes.

## Supporting Information Available

Experimental details, spectroscopic data and copies of NMR spectra for all products (PDF)
