# Supplementary material for: Copper-Catalyzed Selective Amino-alkoxycarbonylation of Unactivated Alkenes with CO
Source: J Am Chem Soc. 2025 Feb 17;147(8):6464–71. doi: 10.1021/jacs.4c13723 (PMC11869293; doi:10.1021/jacs.4c13723)

Supporting Information

Copper-Catalyzed Selective Amino-alkoxycarbonylation of Unactivated Alkenes with CO

Si-Shun Yan,† Ralf Jackstell,† and Matthias Beller*,†
†Leibniz-Institut für Katalyse e.V. an der Universität Rostock, Albert-Einstein-Straße 29a, Rostock 18059, Germany

Table of Contents

1. General information: Materials and methods 3
2. Synthesis of substrates 3
2.1 Preparation of *O*-benzoyl hydroxylamines[1] 3
2.2 Preparation of alkenes derived from natural products and drugs[2] 3
3. Copper catalyzed 1,2-amino-alkoxycarbonylation and acyloxy-amination of non-activated alkenes 4
3.1 General procedure for 1,2-amino-methoxycarbonylation of 4-phenyl-1-butene 4
3.2 Optimization of reaction conditions 4
3.3 General procedure for 1,2-amino-alkoxycarbonylation 7
3.4 Characterization of products 8
3.5 Results for 1,2-amino-methoxycarbonylation of internal alkenes and styrenes 23
3.6 Preliminary results for asymmetric 1,2-amino-methoxycarbonylation of 1-octene 23
3.7 Stereochemistry of major diastereomer of product 3v 24
3.8 General procedure for acyloxy-amination of non-activated alkenes 26
4. Mechanistic studies 29
4.1 Control experiments in the presence of TEMPO 29
4.2 Control experiments in the presence of BHT 30
4.3 Radical clock experiment 31
4.4 Control experiment using LiOMe instead of MeOH 32
4.5 Reaction monitorization and control experiment 33
5. References 33
6. NMR spectra 35

1. General information: Materials and methods

All commercial reagents were ordered from Alfa Aesar, Abcr, Aldrich, TCI or BLD. CO gas was used from Linde. Unless otherwise stated, commercial reagents were used without any purification. Air- and moisture-sensitive syntheses were performed under argon atmosphere in heating gun vacuum dried glassware. Analytical data of literature known compounds were in accordance with reported data. NMR spectra were recorded on Bruker Avance 300 (300 MHz) NMR spectrometers. Multiplets were assigned as s (singlet), d (doublet), t (triplet), dd (doublet of doublet), m (multiplet) and br. s (broad singlet). All measurements were carried out at room temperature unless otherwise stated. High resolution mass spectra (HRMS) were recorded on Agilent 6210 Time-of-Flight LC/MS (Agilent) with electrospray ionization (ESI). The data are given as mass units per charge (m/z) and intensities of signals are given in brackets. For GC analysis, an Agilent 7890A chromatograph with a 30 m HP5 column was used. Thin layer chromatography (TLC) was performed using commercially prepared 100-400 mesh silica gel aluminum plates (GF254), and visualization was affected at 254 nm. Cautionary note: Carbon monoxide is harmful when breathed. Gas charging and releasing should be operated in fume hood.

2. Synthesis of substrates

2.1 Preparation of *O*-benzoyl hydroxylamines[1]

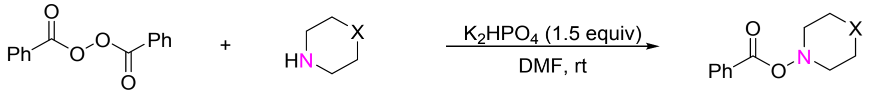

A 50-mL round-bottomed flask equipped with a stirring bar was charged with benzoyl peroxide (1.21 g, 5 mmol), dipotassium hydrogen phosphate (1.31 g, 7.5 mmol), and *N,N*-dimethylformamide (20 mL). The suspension was stirred, and amine (6 mmol) was added via syringe in one portion. The suspension was stirred at room temperature until all benzoyl peroxide was consumed. Deionized water (20 mL) was added, and the contents were stirred vigorously for several minutes until all solids dissolved. The reaction mixture was extracted with 20 mL of ethyl acetate. The organic phase was collected and washed with two 20-mL portions of saturated aq $NaHCO_3$ solution. All the aqueous fractions were combined and extracted with two 20-mL portions of ethyl acetate. All the organic fractions were combined and dried over anhydrous Na_2SO_4 and concentrated by rotary evaporation. The resulting crude product mixture was purified by flash column chromatography using EtOAc/pentanes as solvents to afford the title compound.

2.2 Preparation of alkenes derived from natural products and drugs[2]

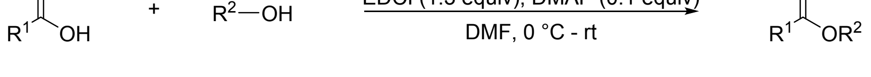

The corresponding acid (8 mmol, 1.0 equiv.) was added to a solution of 1-(3-dimethylaminopropyl)-3-ethylcarbodiimide (EDCI, 1.84 mL, 10.4 mmol, 1.3 equiv.) and DMAP (98 mg, 0.8 mmol, 0.1 equiv.) in CH_2Cl_2 (20 mL) at 0 °C. Alcohol (9.6 mmol 1.2 equiv.) was then added. The reaction mixture was allowed to warm to room temperature overnight. The solution

was diluted with CH_2Cl_2 (20 mL) and washed with 1N HCl (20 mL × 3), saturated $NaHCO_3$ (30 mL), brine (30 mL) sequentially. The organic layer was dried over anhydrous Na_2SO_4. After removal of solvent under reduced pressure, the crude product was purified by column chromatography on silica gel to afford the title compound.

3. Copper catalyzed 1,2-amino-alkoxycarbonylation and acyloxy-amination of non-activated alkenes

3.1 General procedure for 1,2-amino-methoxycarbonylation of 4-phenyl-1-butene

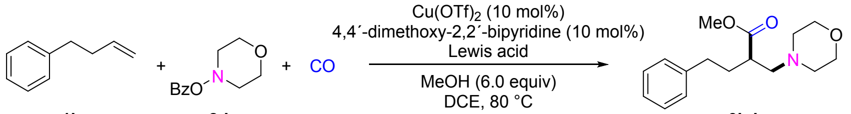

An 8 mL screw-cap vial containing a stirring bar was charged with **2d** (62 mg, 0.3 mmol, 1.5 equiv) and 4,4′-dimethoxy-2,2′-bipyridine (4.3 mg, 0.02 mmol, 10 mol%). The vial was closed by PTFE/white rubber septum (Wheaton 15 mm Septa) and phenolic cap. The reaction vial was then transferred to the glovebox and charged with $Cu(OTf)_2$ (7.2 mg, 0.02 mmol, 10 mol%) and Lewis acid (0.3 – 1.5 equiv). After closed, the reaction vial was moved out of the glovebox and connected to the atmosphere with a needle. The vial was evacuated under vacuum and recharged with argon for three times. Subsequently, anhydrous DCE (2 mL) was added followed by 4-phenyl-1-butene **1k** (30 μL, 0.2 mmol, 1.0 equiv) and MeOH (48 μL, 1.2 mmol, 6.0 equiv) via syringe under argon. The vial was fixed in an alloy plate and put into a Parr 4560 series autoclave (300 mL) under argon atmosphere. At room temperature, the autoclave was flushed with nitrogen for two times and then carbon monoxide for three times. After that, carbon monoxide was charged to set the desired pressure. The reaction was heated to 80 ˚C and stirred for 20 h. Afterwards, the autoclave was cooled to room temperature and the pressure was carefully released. n-Tetradecane (10 μL) was added to the reaction mixture as an internal standard. A sample of the mixture was analyzed by GC. Pure product could be obtained by column chromatography on silica gel visualized by I_2.

3.2 Optimization of reaction conditions

Table S1. The effect of acid additives

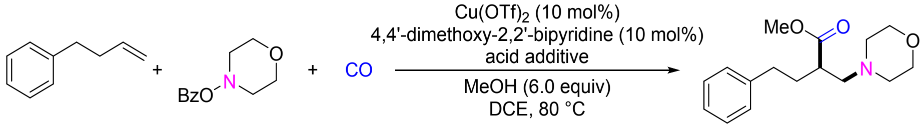

entry	Acid additives	Yield (%)
1	none	Trace
2	$Yb(OTf)_3$ (30 mol%)	17
3	$Zn(OTf)_2$ (30 mol%)	9
4	$Al(OTf)_3$ (30 mol%)	10

5	$Mg(OTf)_2$ (30 mol%)	8
6	$La(OTf)_3$ (30 mol%)	15
7	$LiBF_4$ (1.0 equiv)	26
8	$MgBr_2$ (1.0 equiv)	0
9	$Et_2O{\cdot}BF_3$ (1.0 equiv)	12
10	$AgBF_4$ (1.0 equiv)	39
11	$NaBF_4$ (1.0 equiv)	10
12	$Et_2O{\cdot}HBF_4$ (1.0 equiv)	11
13	TFA (1.0 equiv)	10
14	TfOH (1.0 equiv)	0

Unless otherwise noted, all reactions were performed under 40 bar CO at 80 °C for 20 h in the presence of 4-phenyl-1-butene **1k** (30 µL, 26 mg, 0.2 mmol), morpholino benzoate **2d** (62 mg, 0.3 mmol), $Cu(OTf)_2$ (7.2 mg, 0.02 mmol), ligand (4.3 mg, 0.02 mmol), acid additive (0.06 – 0.2 mmol), MeOH (48 µL, 1.2 mmol) in DCE (2.0 mL). The yields were determined by GC using n-tetradecane ($C_{14}H_{30}$) as an internal standard.

Table S2. The effect of acid additives and pressure

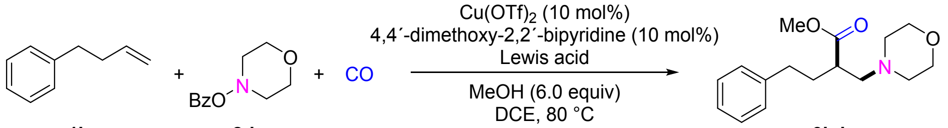

1k **2d** **3kd**

entry	Acid additives	CO pressure (bar)	Yield (%)
1	$LiBF_4$ (1.0 equiv)	50	29
1	$LiBF_4$ (1.0 equiv)	60	33
2	$LiBF_4$ (1.0 equiv)	80	29
3	$AgBF_4$ (1.0 equiv)	80	36
4	$AgBF_4$ (1.0 equiv)	60	51
5	$AgPF_6$ (1.0 equiv)	60	24
6	$LiPF_6$ (1.0 equiv)	60	16
7	$AgSbF_6$ (1.0 equiv)	60	35
8	$AgClO_4$ (1.0 equiv)	60	32

Unless otherwise noted, all reactions were performed at 80 °C for 20 h in the presence of 4-phenyl-1-butene **1k** (30 µL, 26 mg, 0.2 mmol), morpholino benzoate **2d** (62 mg, 0.3 mmol), $Cu(OTf)_2$ (7.2 mg, 0.02 mmol), ligand (4.3 mg, 0.02 mmol), acid additive (0.2 mmol), MeOH (48 µL, 1.2 mmol) in DCE (2.0 mL). The yields were determined by GC using n-tetradecane ($C_{14}H_{30}$) as an internal standard.

Table S3. The effect of other parameters

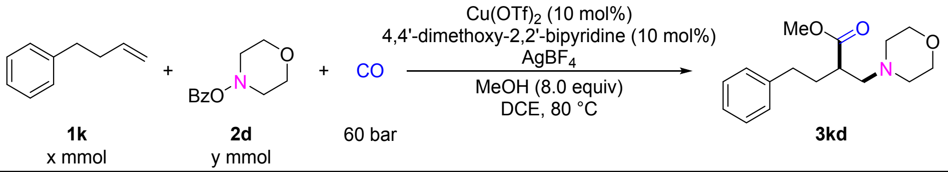

entry	x mmol	y mmol	$AgBF_4$ (mmol)	DCE (mL)	Yield (%)
1	0.2	0.3	0.2	1	43
2	0.2	0.2	0.2	1	42
3	0.3	0.2	0.2	1	44
4	0.4	0.2	0.2	1	52
5	0.4	0.2	0.24	1	53
6	0.4	0.2	0.24	2	57
7	0.4	0.2	0.3	2	58
8	0.4	0.2	0.4	2	55

Unless otherwise noted, all reactions were performed under 60 bar CO at 80 °C for 20 h in the presence of 4-phenyl-1-butene **1k** (x mmol), morpholino benzoate **2d** (y mmol), $Cu(OTf)_2$ (7.2 mg, 0.02 mmol), ligand (4.3 mg, 0.02 mmol), $AgBF_4$ (0.2 – 0.4 mmol), MeOH (65 μL, 1.6 mmol) in DCE (1.0 – 2.0 mL). The yields were determined by GC using n-tetradecane ($C_{14}H_{30}$) as an internal standard.

Table S4. The effect of catalyst loading, ratio of $Cu(OTf)_2$/ligand

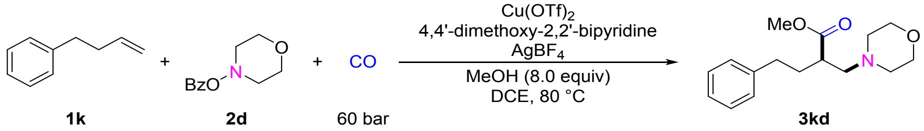

entry	$Cu(OTf)_2$	Ligand	$AgBF_4$ (mmol)	Yield (%)
1	10 mol%	10 mol%	0.24	53
2	10 mol%	0	0.24	trace
3	5 mol%	5 mol%	0.24	32
4	15 mol%	15 mol%	0.24	59
5	15 mol%	15 mol%	0.3	58
6	20 mol%	20 mol%	0.3	58
7	10 mol%	10 mol%	0.3	53
8	10 mol%	8 mol%	0.3	36
9	10 mol%	12 mol%	0.3	56

Unless otherwise noted, all reactions were performed under 60 bar CO at 80 °C for 20 h in the presence of 4-phenyl-1-butene **1k** (53 mg, 0.4 mmol), morpholino benzoate **2d** (41.4 mg, 0.2 mmol), $Cu(OTf)_2$ (0.01 - 0.04 mmol), ligand (0 – 0.04 mmol), $AgBF_4$ (0.24 – 0.3 mmol), MeOH (65 μL, 1.6 mmol) in DCE (2.0 mL). The yields were determined by GC using n-tetradecane ($C_{14}H_{30}$) as an internal standard.

Table S5. The effect of temperature

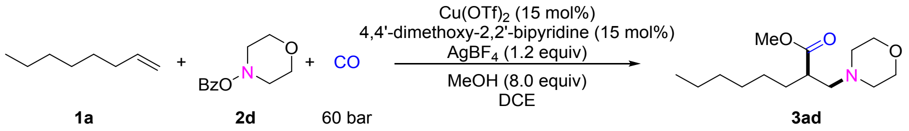

entry	Temp. (°C)	Yield (%)
1	50	51
2	60	65
3	70	59
4	80	56

Unless otherwise noted, all reactions were performed under 60 bar CO for 24 h in the presence of 1-octene **1a** (45 mg, 0.4 mmol), morpholino benzoate **2d** (41.4 mg, 0.2 mmol), $Cu(OTf)_2$ (10.8 mg, 0.03 mmol), ligand (6.5 mg, 0.03 mmol), $AgBF_4$ (46.7 mg, 0.24 mmol), MeOH (65 µL, 1.6 mmol) in DCE (2.0 mL). The yields were determined by GC using n-tetradecane ($C_{14}H_{30}$) as an internal standard.

3.3 General procedure for 1,2-amino-alkoxycarbonylation

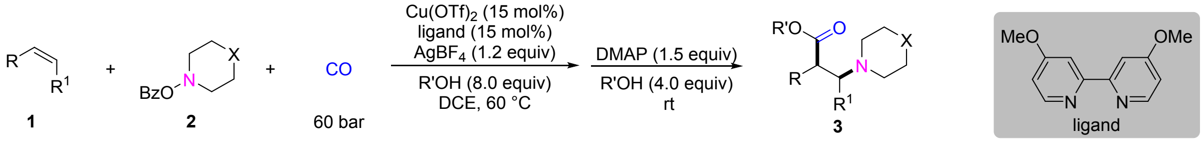

An 8 mL screw-cap vial containing a stirring bar was charged with **2** (0.2 mmol, 1.0 equiv) and 4,4´-dimethoxy-2,2´-bipyridine (6.5 mg, 0.03 mmol, 15 mol%). The vial was closed by PTFE/white rubber septum (Wheaton 15 mm Septa) and phenolic cap. The reaction vial was then transferred to the glovebox and charged with copper triflate (10.8 mg, 0.03 mmol, 15 mol%) and $AgBF_4$ (46.7 mg, 0.24 mmol, 1.2 equiv). After closed, the reaction vial was moved out of the glovebox and connected with atmosphere with a needle. The vial was evacuated under vacuum and recharged with argon for three times. Subsequently, anhydrous DCE (2 mL) was added followed by **1** (0.4 mmol, 2.0 equiv) and MeOH (65 µL, 1.6 mmol, 8.0 equiv) via syringe under argon. The vial was fixed in an alloy plate and put into a Parr 4560 series autoclave (300 mL) under argon atmosphere. At room temperature, the autoclave was flushed with nitrogen for two times and then carbon monoxide for three times. After that, carbon monoxide was charged to 60 bar. The reaction was heated to 60 ˚C and stirred for 24 h. Afterwards, the autoclave was cooled to room temperature and the pressure was carefully released. Then DMAP (36.7 mg, 0.3 mmol, 1.5 equiv) and MeOH (32 µL, 0.8 mmol, 4.0 equiv) were added to the reaction mixture and stirred at room temperature for 4 h. Pure product could be obtained by column chromatography on silica gel visualized by I_2.

3.4 Characterization of products

methyl 2-(piperidin-1-ylmethyl)octanoate (3a)

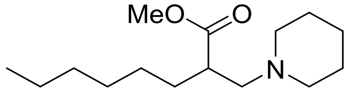

Colorless oil, 68% yield. ^{1}H NMR (300 MHz, $CDCl_3$) δ 3.66 (s, 3H), 2.66 – 2.53 (m, 2H), 2.46 – 2.33 (m, 2H), 2.33 – 2.21 (m, 3H), 1.58 – 1.34 (m, 8H), 1.29 – 1.20 (m, 8H), 0.86 (t, J = 6.8 Hz, 3H).
^{13}C NMR (75 MHz, $CDCl_3$) δ 176.51, 61.47, 54.61, 51.32, 44.00, 31.62, 30.95, 29.14, 27.41, 26.04, 24.36, 22.53, 14.02.
HRMS-ESI (m/z): $[M+H]^+$ calcd for $C_{15}H_{30}NO_2^+$, 256.2271, found 256.2271. The spectral data are consistent with those previously reported in the literature.[3]

methyl 2-(piperidin-1-ylmethyl)decanoate (3b)

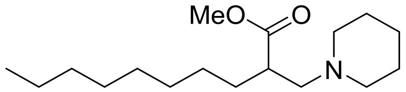

Colorless oil, 71% yield. ^{1}H NMR (300 MHz, $CDCl_3$) δ 3.66 (s, 3H), 2.67 – 2.52 (m, 2H), 2.44 – 2.21 (m, 5H), 1.57 – 1.32 (m, 8H), 1.29 – 1.20 (m, 12H), 0.86 (t, J = 6.7 Hz, 3H).
^{13}C NMR (75 MHz, $CDCl_3$) δ 176.50, 61.48, 54.63, 51.32, 44.02, 31.82, 30.95, 29.49, 29.37, 29.19, 27.45, 26.05, 24.37, 22.63, 14.07.
HRMS-ESI (m/z): $[M+H]^+$ calcd for $C_{17}H_{34}NO_2^+$, 284.2585, found 284.2590.

methyl 2-(piperidin-1-ylmethyl)dodecanoate (3c)

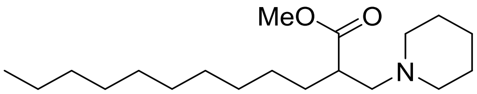

Colorless oil, 64% yield. ^{1}H NMR (300 MHz, $CDCl_3$) δ 3.67 (s, 3H), 2.67 – 2.53 (m, 2H), 2.46 – 2.33 (m, 2H), 2.32 – 2.21 (m, 3H), 1.56 – 1.34 (m, 8H), 1.27 – 1.21 (m, 16H), 0.87 (t, J = 6.8 Hz, 3H).
^{13}C NMR (75 MHz, $CDCl_3$) δ 176.54, 61.49, 54.63, 51.34, 44.01, 31.89, 30.97, 29.57, 29.55, 29.50, 29.43, 29.31, 27.47, 26.05, 24.38, 22.67, 14.10.
HRMS-ESI (m/z): $[M+H]^+$ calcd for $C_{19}H_{38}NO_2^+$, 312.2898, found 312.2901. The spectral data are consistent with those previously reported in the literature.[4]

methyl 3-cyclohexyl-2-(piperidin-1-ylmethyl)propanoate (3d)

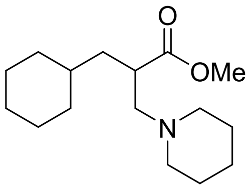

Colorless oil, 50% yield. ^{1}H NMR (400 MHz, $CDCl_3$) δ 3.66 (s, 3H), 2.80 – 2.68 (m, 1H), 2.59 – 2.51 (m, 1H), 2.46 – 2.33 (m, 2H), 2.31 – 2.21 (m, 3H), 1.82 – 1.72 (m, 1H), 1.71 – 1.57 (m, 4H), 1.54 – 1.44 (m, 5H), 1.41 – 1.34 (m, 2H), 1.30 – 1.11 (m, 5H), 0.93 – 0.77 (m, 2H).
^{13}C NMR (101 MHz, $CDCl_3$) δ 176.76, 61.96, 54.59, 51.35, 41.31, 38.54, 35.77, 33.66, 32.79, 26.48, 26.20, 26.16, 26.03, 24.37.
HRMS-ESI (m/z): $[M+H]^+$ calcd for $C_{16}H_{30}NO_2^+$, 268.2272, found 268.2274.

dimethyl 2-(piperidin-1-ylmethyl)undecanedioate (**3e**)

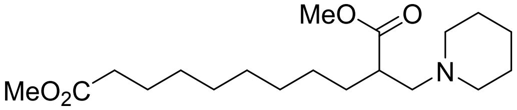

Colorless oil, 61% yield. ^{1}H NMR (300 MHz, $CDCl_3$) δ 3.66 (s, 3H), 3.65 (s, 3H), 2.66 – 2.51 (m, 2H), 2.45 – 2.33 (m, 2H), 2.31 – 2.22 (m, 5H), 1.64 – 1.34 (m, 10H), 1.31 – 1.20 (m, 10H).
^{13}C NMR (75 MHz, $CDCl_3$) δ 176.46, 174.26, 61.45, 54.62, 51.39, 51.32, 43.99, 34.05, 30.90, 29.39, 29.18, 29.10, 29.05, 27.40, 26.04, 24.88, 24.36.
HRMS-ESI (m/z): $[M+H]^+$ calcd for $C_{19}H_{36}NO_4^+$, 342.2639, found 342.2642.

methyl 6-chloro-2-(piperidin-1-ylmethyl)hexanoate (**3f**)

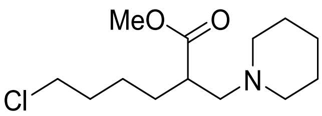

Colorless oil, 51% yield. ^{1}H NMR (300 MHz, $CDCl_3$) δ 3.67 (s, 3H), 3.50 (td, J = 6.7, 0.7 Hz, 2H), 2.67 – 2.51 (m, 2H), 2.43 – 2.23 (m, 5H), 1.81 – 1.70 (m, 2H), 1.60 – 1.33 (m, 10H).
^{13}C NMR (75 MHz, $CDCl_3$) δ 176.09, 61.26, 54.63, 51.42, 44.69, 43.79, 32.38, 29.98, 26.02, 24.73, 24.32.
HRMS-ESI (m/z): $[M+H]^+$ calcd for $C_{13}H_{25}ClNO_2^+$, 262.1569, found 262.1571.

methyl 6-acetoxy-2-(piperidin-1-ylmethyl)hexanoate (**3g**)

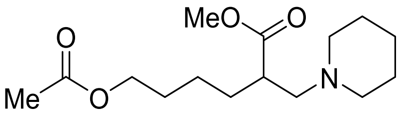

Colorless oil, 53% yield. ^{1}H NMR (300 MHz, $CDCl_3$) δ 4.02 (t, J = 6.6 Hz, 2H), 3.66 (s, 3H), 2.67 – 2.52 (m, 2H), 2.44 – 2.23 (m, 5H), 2.02 (s, 3H), 1.66 – 1.44 (m, 8H), 1.41 – 1.26 (m, 4H).
^{13}C NMR (75 MHz, $CDCl_3$) δ 176.15, 171.11, 64.22, 61.31, 54.63, 51.40, 43.87, 30.40, 28.45, 26.02, 24.33, 23.87, 20.94.

HRMS-ESI (m/z): $[M+H]^+$ calcd for $C_{15}H_{28}NO_4^+$, 286.2013, found 286.2022.

methyl 2-(piperidin-1-ylmethyl)decanoat methyl 2-(piperidin-1-ylmethyl)-6-(tosyloxy)hexanoate (3h)

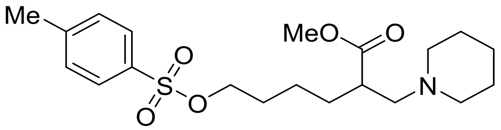

Colorless oil, 57% yield. ^{1}H NMR (300 MHz, $CDCl_3$) δ 7.80 – 7.73 (m, 2H), 7.39 – 7.28 (m, 2H), 3.98 (td, J = 6.5, 0.6 Hz, 2H), 3.64 (s, 3H), 2.65 – 2.51 (m, 2H), 2.44 (s, 3H), 2.42 – 2.24 (m, 5H), 1.70 – 1.56 (m, 2H), 1.56 – 1.46 (m, 5H), 1.45 – 1.34 (m, 3H), 1.33 – 1.24 (m, 2H).
^{13}C NMR (75 MHz, $CDCl_3$) δ 175.90, 144.65, 133.07, 129.79, 127.82, 70.20, 61.02, 54.51, 51.47, 43.52, 30.10, 28.64, 25.81, 24.17, 23.26, 21.58.
HRMS-ESI (m/z): $[M+H]^+$ calcd for $C_{20}H_{32}NO_5S^+$, 398.1996, found 398.2006.

methyl 2-cyclohexyl-3-(piperidin-1-yl)propanoate (3i)

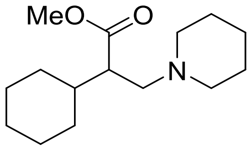

Colorless oil, 55% yield. ^{1}H NMR (300 MHz, $CDCl_3$) δ 3.66 (s, 3H), 2.69 – 2.56 (m, 1H), 2.50 – 2.33 (m, 4H), 2.28 – 2.17 (m, 2H), 1.81 – 1.45 (m, 10H), 1.36 (p, J = 5.3 Hz, 2H), 1.24 – 1.12 (m, 3H), 1.08 – 0.88 (m, 2H).
^{13}C NMR (75 MHz, $CDCl_3$) δ 175.88, 59.27, 54.65, 51.11, 50.00, 39.33, 31.05, 30.91, 26.31, 26.24, 26.17, 26.07, 24.38.
HRMS-ESI (m/z): $[M+H]^+$ calcd for $C_{15}H_{28}NO_2^+$, 254.2115, found 254.2116.

methyl 4-phenyl-2-(piperidin-1-ylmethyl)butanoate (3j)

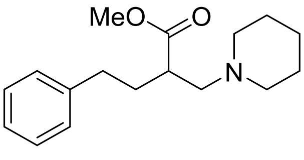

Colorless oil, 50% yield. ^{1}H NMR (300 MHz, $CDCl_3$) δ 7.31 – 7.24 (m, 2H), 7.21 – 7.15 (m, 3H), 3.69 (s, 3H), 2.73 – 2.53 (m, 4H), 2.42 – 2.23 (m, 5H), 1.97 – 1.76 (m, 2H), 1.56 – 1.46 (m, 4H), 1.43 – 1.34 (m, 2H).
^{13}C NMR (75 MHz, $CDCl_3$) δ 176.09, 141.58, 128.39, 128.29, 125.86, 61.22, 54.62, 51.43, 43.47, 33.63, 32.38, 26.03, 24.34.
HRMS-ESI (m/z): $[M+H]^+$ calcd for $C_{17}H_{26}NO_2^+$, 276.1959, found 276.1964.

methyl 4-(4-(*tert*-butyl)phenyl)-2-(piperidin-1-ylmethyl)butanoate (3k)

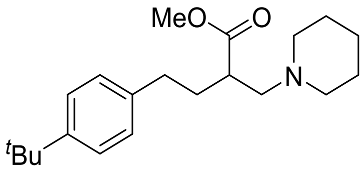

Colorless oil, 45% yield. ^{1}H NMR (300 MHz, $CDCl_3$) δ 7.33 – 7.28 (m, 2H), 7.14 – 7.08 (m, 2H), 3.68 (s, 3H), 2.74 – 2.48 (m, 4H), 2.43 – 2.21 (m, 5H), 1.97 – 1.74 (m, 2H), 1.55 – 1.46 (m, 4H), 1.43 – 1.35 (m, 2H), 1.31 (s, 9H).
^{13}C NMR (75 MHz, $CDCl_3$) δ 176.15, 148.64, 138.54, 128.04, 125.18, 61.23, 54.63, 51.42, 43.60, 34.33, 33.08, 32.40, 31.38, 26.06, 24.36.
HRMS-ESI (m/z): $[M+H]^+$ calcd for $C_{21}H_{34}NO_2^+$, 332.2585, found 332.2589.

methyl 4-([1,1'-biphenyl]-4-yl)-2-(piperidin-1-ylmethyl)butanoate (3l)

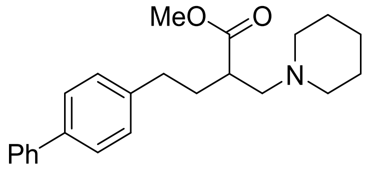

Colorless oil, 60% yield. ^{1}H NMR (300 MHz, $CDCl_3$) δ 7.62 – 7.56 (m, 2H), 7.57 – 7.47 (m, 2H), 7.47 – 7.40 (m, 2H), 7.37 – 7.29 (m, 1H), 7.28 – 7.23 (m, 2H), 3.71 (s, 3H), 2.78 – 2.56 (m, 4H), 2.46 – 2.24 (m, 5H), 2.05 – 1.80 (m, 2H), 1.59 – 1.47 (m, 4H), 1.45 – 1.35 (m, 2H).
^{13}C NMR (75 MHz, $CDCl_3$) δ 176.07, 141.03, 140.71, 138.84, 128.83, 128.68, 127.04, 127.00, 126.95, 61.20, 54.63, 51.47, 43.47, 33.26, 32.35, 26.03, 24.34.
HRMS-ESI (m/z): $[M+H]^+$ calcd for $C_{23}H_{30}NO_2^+$, 352.2272, found 352.2264.

methyl 2-benzyl-3-(piperidin-1-yl)propanoate (3m)

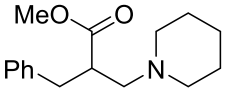

Colorless oil, 52% yield. ^{1}H NMR (300 MHz, $CDCl_3$) δ 7.29 – 7.22 (m, 2H), 7.22 – 7.12 (m, 3H), 3.58 (s, 3H), 3.01 – 2.80 (m, 3H), 2.71 – 2.61 (m, 1H), 2.47 – 2.27 (m, 5H), 1.58 – 1.47 (m, 4H), 1.44 – 1.34 (m, 2H).
^{13}C NMR (75 MHz, $CDCl_3$) δ 175.51, 139.24, 128.78, 128.31, 126.25, 60.59, 54.49, 51.40, 45.81, 36.71, 25.95, 24.29.
HRMS-ESI (m/z): $[M+H]^+$ calcd for $C_{16}H_{24}NO_2^+$, 262.1802, found 262.1797. The spectral data are consistent with those previously reported in the literature.[3]

methyl 5-oxo-2-(piperidin-1-ylmethyl)hexanoate (3n)

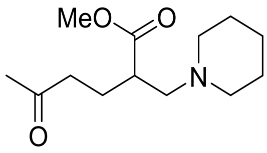

Colorless oil, 53% yield. ^{1}H NMR (300 MHz, $CDCl_3$) δ 3.66 (s, 3H), 2.69 – 2.56 (m, 2H), 2.48 – 2.27 (m, 7H), 2.11 (s, 3H), 1.91 – 1.71 (m, 2H), 1.56 – 1.47 (m, 4H), 1.42 – 1.33 (m, 2H).
^{13}C NMR (75 MHz, $CDCl_3$) δ 207.87, 175.64, 60.88, 54.55, 51.52, 42.83, 40.98, 29.89, 25.89, 24.42, 24.23.
HRMS-ESI (m/z): $[M+H]^+$ calcd for $C_{13}H_{24}NO_3^+$, 242.1751, found 242.1750.

methyl 5-phenoxy-2-(piperidin-1-ylmethyl)pentanoate (**3o**)

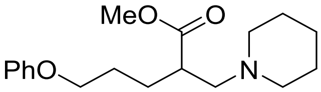

Colorless oil, 45% yield. ^{1}H NMR (400 MHz, $CDCl_3$) δ 7.27 – 7.21 (m, 2H), 6.90 (tt, *J* = 7.5, 1.1 Hz, 1H), 6.87 – 6.82 (m, 2H), 3.91 (td, *J* = 6.2, 2.0 Hz, 2H), 3.65 (s, 3H), 2.72 – 2.62 (m, 1H), 2.62 – 2.55 (m, 1H), 2.44 – 2.22 (m, 5H), 1.79 – 1.65 (m, 4H), 1.54 – 1.45 (m, 4H), 1.40 – 1.32 (m, 2H).
^{13}C NMR (101 MHz, $CDCl_3$) δ 176.07, 158.87, 129.35, 120.53, 114.41, 67.27, 61.28, 54.63, 51.44, 43.64, 27.27, 27.14, 26.02, 24.32.
HRMS-ESI (m/z): $[M+H]^+$ calcd for $C_{18}H_{28}NO_3^+$, 306.2064, found 306.2071.

1,1-diethyl 3-methyl 4-(piperidin-1-yl)butane-1,1,3-tricarboxylate (**3p**)

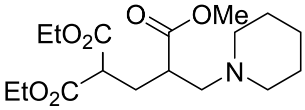

Colorless oil, 36% yield. ^{1}H NMR (300 MHz, $CDCl_3$) δ 4.23 – 4.14 (m, 4H), 3.67 (s, 3H), 3.45 (dd, *J* = 8.8, 6.4 Hz, 1H), 2.75 – 2.64 (m, 1H), 2.61 – 2.52 (m, 1H), 2.43 – 2.26 (m, 5H), 2.24 – 2.06 (m, 2H), 1.55 – 1.46 (m, 4H), 1.42 – 1.34 (m, 2H), 1.26 (td, *J* = 7.1, 2.7 Hz, 6H).
^{13}C NMR (75 MHz, $CDCl_3$) δ 175.08, 169.07, 61.46, 61.42, 61.08, 54.58, 51.65, 49.98, 41.30, 29.30, 25.99, 24.31, 14.04, 14.01.
HRMS-ESI (m/z): $[M+H]^+$ calcd for $C_{17}H_{30}NO_6^+$, 344.2068, found 344.2072.

methyl 11-(diethylamino)-11-oxo-2-(piperidin-1-ylmethyl)undecanoate (**3q**)

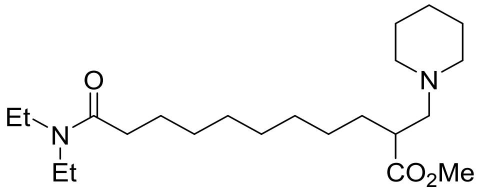

Colorless oil, 54% yield. ^{1}H NMR (300 MHz, $CDCl_3$) δ 3.63 (s, 3H), 3.29 (dq, J = 20.0, 7.1 Hz, 4H), 2.63 – 2.49 (m, 2H), 2.41 – 2.30 (m, 2H), 2.30 – 2.15 (m, 5H), 1.63 – 1.31 (m, 10H), 1.27 – 1.17 (m, 10H), 1.12 (t, J = 7.1 Hz, 3H), 1.06 (t, J = 7.1 Hz, 3H).
^{13}C NMR (75 MHz, $CDCl_3$) δ 176.40, 172.16, 61.38, 54.53, 51.25, 43.90, 41.84, 39.89, 33.03, 30.85, 29.37, 29.35, 29.27, 29.18, 27.34, 25.94, 25.36, 24.27, 14.30, 13.01.
HRMS-ESI (m/z): $[M+H]^+$ calcd for $C_{22}H_{43}N_2O_3^+$, 383.3269, found 383.3270.

methyl 8-(1,3-dioxoisoindolin-2-yl)-2-(piperidin-1-ylmethyl)octanoate (3r)

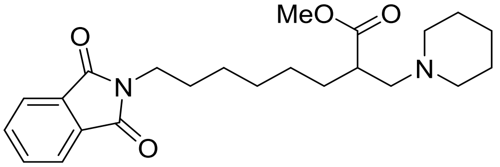

Colorless oil, 61% yield. ^{1}H NMR (300 MHz, $CDCl_3$) δ 7.87 – 7.73 (m, 2H), 7.71 – 7.61 (m, 2H), 3.67 – 3.55 (m, 5H), 2.65 – 2.50 (m, 2H), 2.46 – 2.17 (m, 5H), 1.67 – 1.39 (m, 8H), 1.36 – 1.18 (m, 8H).
^{13}C NMR (75 MHz, $CDCl_3$) δ 176.22, 168.29, 133.73, 132.03, 123.02, 61.16, 54.45, 51.31, 43.67, 37.84, 30.76, 28.93, 28.38, 27.15, 26.54, 25.79, 24.17.
HRMS-ESI (m/z): $[M+H]^+$ calcd for $C_{23}H_{33}N_2O_4^+$, 401.2435, found 401.2437.

1-methyl 11-(pent-3-yn-1-yl) 2-(piperidin-1-ylmethyl)undecanedioate (3s)

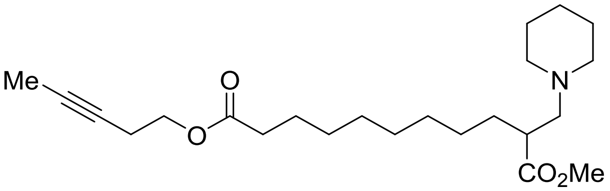

Colorless oil, 63% yield. ^{1}H NMR (300 MHz, $CDCl_3$) δ 4.10 (t, J = 7.0 Hz, 2H), 3.65 (s, 3H), 2.67 – 2.51 (m, 2H), 2.47 – 2.33 (m, 4H), 2.34 – 2.19 (m, 5H), 1.75 (t, J = 2.5 Hz, 3H), 1.64 – 1.55 (m, 2H), 1.55 – 1.41 (m, 6H), 1.40 – 1.33 (m, 2H), 1.29 – 1.19 (m, 10H).
^{13}C NMR (75 MHz, $CDCl_3$) δ 176.44, 173.57, 77.13, 74.71, 62.53, 61.41, 54.58, 51.32, 43.93, 34.16, 30.90, 29.37, 29.17, 29.10, 29.00, 27.38, 25.97, 24.85, 24.31, 19.19, 3.40.
HRMS-ESI (m/z): $[M+H]^+$ calcd for $C_{23}H_{40}NO_4^+$, 394.2952, found 394.2955.

methyl 2-(piperidin-1-yl)cyclopentane-1-carboxylate (3t)

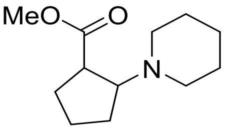

Colorless oil, 60% yield, d.r. > 20:1 (determined by GC and GC-MS analysis). ^{1}H NMR (300 MHz, $CDCl_3$) δ 3.65 (s, 3H), 3.15 – 3.05 (m, 1H), 2.80 – 2.69 (m, 1H), 2.40 (t, J = 5.3 Hz, 4H), 2.02 – 1.89 (m, 2H), 1.81 – 1.71 (m, 1H), 1.70 – 1.61 (m, 2H), 1.59 – 1.49 (m, 5H), 1.43 – 1.35 (m, 2H).

^{13}C NMR (75 MHz, $CDCl_3$) δ 177.56, 70.80, 52.31, 51.83, 46.34, 31.15, 31.01, 26.13, 24.77, 24.48.
HRMS-EI: calcd for $C_{12}H_{21}NO_2$, 211.1567, found 211.1562.

1,1-diethyl 3-methyl 4-(piperidin-1-yl)cyclopentane-1,1,3-tricarboxylate (**3u**)

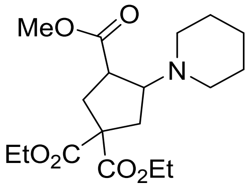

Colorless oil, 51% yield. d.r. > 20:1 (determined by GC and GC-MS analysis). ^{1}H NMR (300 MHz, $CDCl_3$) δ 4.23 – 4.09 (m, 4H), 3.66 (s, 3H), 3.36 – 3.26 (m, 1H), 2.89 (q, J = 8.6 Hz, 1H), 2.66 – 2.55 (m, 2H), 2.46 – 2.29 (m, 5H), 2.06 (dd, J = 13.5, 9.9 Hz, 1H), 1.56 – 1.46 (m, 4H), 1.43 – 1.34 (m, 2H), 1.21 (td, J = 7.1, 1.5 Hz, 6H).
^{13}C NMR (75 MHz, $CDCl_3$) δ 175.40, 171.59, 171.46, 69.11, 61.75, 61.73, 57.93, 52.02, 51.58, 45.18, 36.58, 36.45, 26.24, 24.51, 14.10, 14.08.
HRMS-EI: calcd for $C_{18}H_{29}NO_6$, 355.1989, found 355.1987.

methyl 2-(piperidin-1-yl)cyclohexane-1-carboxylate (**3v**)

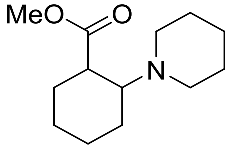

Colorless oil, 53% yield, d.r. 4.2:1 (determined by GC and GC-MS analysis). *trans*-**3v**: ^{1}H NMR (300 MHz, $CDCl_3$) δ 3.65 (s, 3H), 2.74 – 2.40 (m, 4H), 2.38 – 2.21 (m, 2H), 1.91 – 1.74 (m, 3H), 1.73 – 1.64 (m, 1H), 1.57 – 1.31 (m, 7H), 1.24 – 1.06 (m, 3H).
^{13}C NMR (75 MHz, $CDCl_3$) δ 176.39, 66.60, 51.08, 49.80, 48.08, 29.43, 26.94, 25.48, 25.23, 25.06, 23.96.
HRMS-ESI (m/z): $[M+H]^+$ calcd for $C_{13}H_{24}NO_2^+$, 226.1802, found 226.1808.

methyl 2-((4-methylpiperidin-1-yl)methyl)dodecanoate (**3w**)

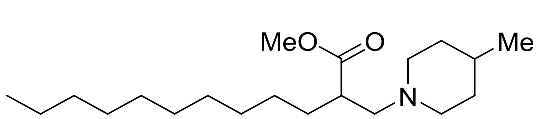

Colorless oil, 65% yield. ^{1}H NMR (300 MHz, $CDCl_3$) δ 3.66 (s, 3H), 2.92 – 2.82 (m, 1H), 2.76 – 2.68 (m, 1H), 2.66 – 2.54 (m, 2H), 2.35 – 2.24 (m, 1H), 1.90 (dtd, J = 25.3, 11.4, 2.6 Hz, 2H), 1.59 – 1.41 (m, 4H), 1.28 – 1.11 (m, 19H), 0.90 – 0.84 (m, 6H).
^{13}C NMR (75 MHz, $CDCl_3$) δ 176.50, 61.10, 54.39, 53.74, 51.33, 44.12, 34.45, 34.39, 31.88, 30.97, 30.75, 29.56, 29.54, 29.49, 29.42, 29.30, 27.46, 22.66, 21.88, 14.09.
HRMS-ESI (m/z): $[M+H]^+$ calcd for $C_{20}H_{40}NO_2^+$, 326.3054, found 326.3057.

ethyl 1-(2-(methoxycarbonyl)dodecyl)piperidine-4-carboxylate (3x)

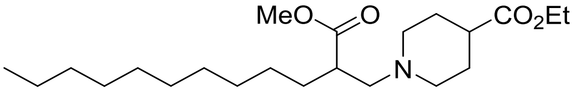

Colorless oil, 52% yield. ^{1}H NMR (400 MHz, $CDCl_3$) δ 4.10 (q, J = 7.2 Hz, 2H), 3.66 (s, 3H), 2.93 – 2.85 (m, 1H), 2.77 – 2.69 (m, 1H), 2.65 – 2.54 (m, 2H), 2.35 – 2.15 (m, 2H), 2.04 (td, J = 11.3, 2.8 Hz, 1H), 1.93 (td, J = 11.3, 2.7 Hz, 1H), 1.86 – 1.77 (m, 2H), 1.73 – 1.61 (m, 2H), 1.57 – 1.36 (m, 2H), 1.29 – 1.19 (m, 19H), 0.86 (t, J = 6.9 Hz, 3H).
^{13}C NMR (101 MHz, $CDCl_3$) δ 176.26, 175.22, 60.83, 60.19, 53.49, 52.67, 51.37, 44.08, 41.16, 31.86, 30.75, 29.54, 29.52, 29.47, 29.39, 29.28, 28.35, 28.31, 27.43, 22.64, 14.18, 14.08.
HRMS-ESI (m/z): $[M+H]^+$ calcd for $C_{22}H_{42}NO_4^+$, 384.3109, found 384.3108.

methyl 2-(morpholinomethyl)dodecanoate (3y)

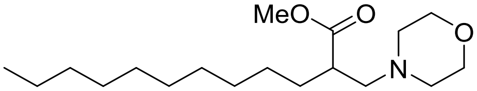

Colorless oil, 58% yield. ^{1}H NMR (400 MHz, $CDCl_3$) δ 3.74 – 3.55 (m, 7H), 2.66 – 2.56 (m, 2H), 2.53 – 2.42 (m, 2H), 2.39 – 2.25 (m, 3H), 1.62 – 1.37 (m, 2H), 1.29 – 1.17 (m, 16H), 0.87 (t, J = 6.9 Hz, 3H).
^{13}C NMR (101 MHz, $CDCl_3$) δ 176.07, 67.01, 61.05, 53.69, 51.39, 43.67, 31.86, 30.67, 29.54, 29.52, 29.46, 29.39, 29.28, 27.41, 22.64, 14.08.
HRMS-ESI (m/z): $[M+H]^+$ calcd for $C_{18}H_{36}NO_3^+$, 314.2690, found 314.2692.

methyl 2-((2,6-dimethylmorpholino)methyl)dodecanoate (3z)

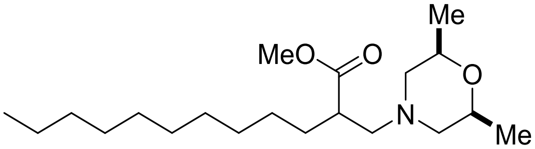

Colorless oil, 59% yield. ^{1}H NMR (300 MHz, $CDCl_3$) δ 3.61 (s, 3H), 3.57 – 3.47 (m, 2H), 2.70 (dt, J = 10.9, 2.0 Hz, 1H), 2.62 – 2.48 (m, 3H), 2.26 – 2.16 (m, 1H), 1.70 (dd, J = 11.1, 10.0 Hz, 1H), 1.59 (dd, J = 10.9, 10.0 Hz, 1H), 1.52 – 1.32 (m, 2H), 1.23 – 1.15 (m, 16H), 1.06 (t, J = 6.2 Hz, 6H), 0.81 (t, J = 6.7 Hz, 3H).
^{13}C NMR (75 MHz, $CDCl_3$) δ 176.14, 71.66, 60.66, 60.07, 59.04, 51.37, 43.71, 31.87, 30.69, 29.55, 29.52, 29.46, 29.39, 29.29, 27.42, 22.65, 19.11, 19.01, 14.08.
HRMS-ESI (m/z): $[M+H]^+$ calcd for $C_{20}H_{40}NO_3^+$, 342.3003, found 342.2998.

methyl 2-(azepan-1-ylmethyl)dodecanoate (3aa)

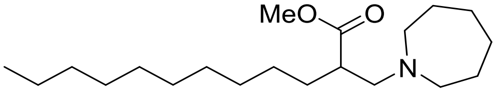

Colorless oil, 39% yield. ^{1}H NMR (300 MHz, $CDCl_3$) δ 3.67 (s, 3H), 2.78 – 2.46 (m, 7H), 1.61 – 1.39 (m, 10H), 1.28 – 1.23 (m, 16H), 0.87 (t, J = 6.7 Hz, 3H).
^{13}C NMR (75 MHz, $CDCl_3$) δ 176.64, 60.68, 55.46, 51.28, 45.41, 31.90, 30.55, 29.58, 29.55, 29.54, 29.44, 29.31, 28.58, 27.54, 27.07, 22.67, 14.10.
HRMS-ESI (m/z): $[M+H]^+$ calcd for $C_{20}H_{40}NO_2^+$, 326.3054, found 326.3058.

methyl 2-(pyrrolidin-1-ylmethyl)dodecanoate (**3bb**)

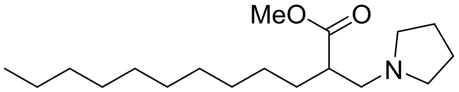

Colorless oil, 35% yield. ^{1}H NMR (300 MHz, $CDCl_3$) δ 3.67 (s, 3H), 2.77 (dd, J = 11.6, 9.2 Hz, 1H), 2.65 – 2.34 (m, 6H), 1.80 – 1.64 (m, 4H), 1.60 – 1.42 (m, 2H), 1.27 – 1.21 (m, 16H), 0.87 (t, J = 6.7 Hz, 3H).
^{13}C NMR (75 MHz, $CDCl_3$) δ 176.41, 58.71, 54.26, 51.43, 45.76, 31.88, 31.06, 29.56, 29.54, 29.48, 29.43, 29.30, 27.44, 23.51, 22.66, 14.09.
HRMS-ESI (m/z): $[M+H]^+$ calcd for $C_{18}H_{36}NO_2^+$, 298.2741, found 298.2741.

benzyl 4-(2-(methoxycarbonyl)dodecyl)piperazine-1-carboxylate (**3cc**)

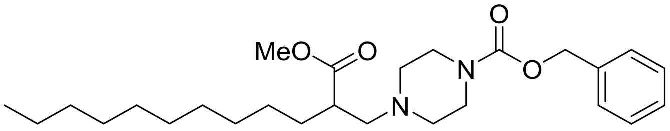

Colorless oil, 51% yield. ^{1}H NMR (300 MHz, $CDCl_3$) δ 7.42 – 7.25 (m, 5H), 5.12 (s, 2H), 3.67 (s, 3H), 3.51 – 3.39 (m, 4H), 2.71 – 2.56 (m, 2H), 2.55 – 2.39 (m, 2H), 2.41 – 2.21 (m, 3H), 1.62 – 1.38 (m, 2H), 1.30 – 1.19 (m, 16H), 0.87 (t, J = 6.7 Hz, 3H).
^{13}C NMR (75 MHz, $CDCl_3$) δ 175.98, 155.16, 136.70, 128.43, 127.94, 127.82, 67.02, 60.52, 52.84, 51.42, 43.82, 31.85, 30.69, 29.52, 29.50, 29.43, 29.37, 29.26, 27.36, 22.63, 14.07.
HRMS-ESI (m/z): $[M+H]^+$ calcd for $C_{26}H_{43}N_2O_4^+$, 447.3218, found 447.3217.

methyl 2-((4-benzoylpiperazin-1-yl)methyl)dodecanoate (**3dd**)

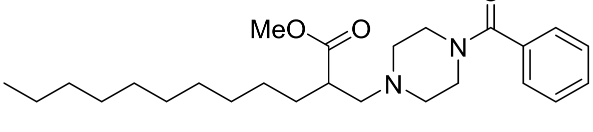

Colorless oil, 44% yield. ^{1}H NMR (300 MHz, $CDCl_3$) δ 7.45 – 7.32 (m, 5H), 3.91 – 3.54 (m, 5H), 3.52 – 3.18 (m, 2H), 2.75 – 2.24 (m, 7H), 1.61 – 1.37 (m, 2H), 1.29 – 1.19 (m, 16H), 0.86 (t, J = 6.7 Hz, 3H).
^{13}C NMR (75 MHz, $CDCl_3$) δ 175.91, 170.18, 135.82, 129.56, 128.39, 126.97, 60.42, 51.42, 43.89, 31.84, 30.64, 29.51, 29.49, 29.42, 29.35, 29.25, 27.36, 22.62, 14.06.
HRMS-ESI (m/z): $[M+H]^+$ calcd for $C_{25}H_{41}N_2O_3^+$, 417.3112, found 417.3115.

***tert*-butyl 4-(2-(methoxycarbonyl)dodecyl)piperazine-1-carboxylate (3ee)**

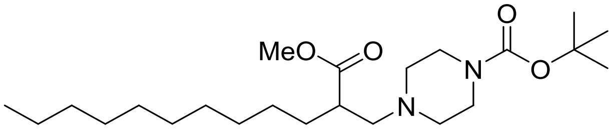

Colorless oil, 39% yield. ^{1}H NMR (300 MHz, $CDCl_3$) δ 3.66 (s, 3H), 3.41 – 3.27 (m, 4H), 2.68 – 2.55 (m, 2H), 2.48 – 2.37 (m, 2H), 2.35 – 2.22 (m, 3H), 1.56 – 1.41 (m, 11H), 1.29 – 1.20 (m, 16H), 0.87 (t, J = 6.7 Hz, 3H).
^{13}C NMR (75 MHz, $CDCl_3$) δ 176.06, 154.74, 79.48, 60.62, 53.00, 51.40, 43.91, 31.87, 30.69, 29.54, 29.52, 29.45, 29.39, 29.28, 28.39, 27.40, 22.64, 14.08.
HRMS-ESI (m/z): $[M+H]^+$ calcd for $C_{23}H_{45}N_2O_4^+$, 413.3374, found 413.3376.

methyl 2-((diethylamino)methyl)dodecanoate (3ff)

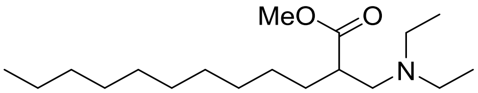

Colorless oil, 35% yield. ^{1}H NMR (300 MHz, $CDCl_3$) δ 3.66 (s, 3H), 2.78 – 2.63 (m, 1H), 2.61 – 2.34 (m, 6H), 1.57 – 1.40 (m, 2H), 1.27 – 1.22 (m, 16H), 0.97 (t, J = 7.1 Hz, 6H), 0.87 (t, J = 6.7 Hz, 3H).
^{13}C NMR (75 MHz, $CDCl_3$) δ 176.47, 55.71, 51.30, 47.31, 45.15, 31.89, 30.82, 29.57, 29.55, 29.53, 29.43, 29.31, 27.54, 22.67, 14.10, 11.86.
HRMS-ESI (m/z): $[M+H]^+$ calcd for $C_{18}H_{38}NO_2^+$, 300.2898, found 300.2905.

methyl 2-((4-((*S*)-(4-chlorophenyl)(pyridin-2-yl)methoxy)piperidin-1-yl)methyl)decanoate (3gg)

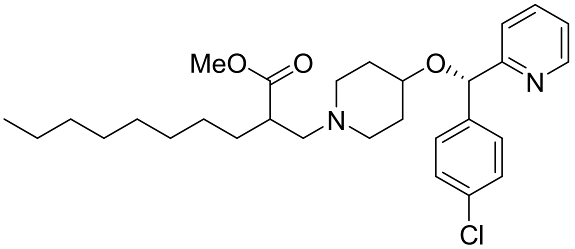

Colorless oil, 24% yield. ^{1}H NMR (300 MHz, $CDCl_3$) δ 8.56 – 8.45 (m, 1H), 7.70 – 7.63 (m, 1H), 7.56 – 7.50 (m, 1H), 7.37 – 7.31 (m, 2H), 7.29 – 7.24 (m, 2H), 7.15 (ddd, J = 7.5, 4.9, 1.3 Hz, 1H), 5.58 (s, 1H), 3.66 (s, 3H), 3.41 (tt, J = 8.2, 4.0 Hz, 1H), 2.87 – 2.72 (m, 1H), 2.69 – 2.52 (m, 3H), 2.36 – 2.24 (m, 1H), 2.21 – 2.11 (m, 1H), 2.09 – 1.99 (m, 1H), 1.91 – 1.76 (m, 2H), 1.66 (dtq, J = 13.3, 9.0, 4.6 Hz, 2H), 1.56 – 1.38 (m, 2H), 1.30 – 1.19 (m, 12H), 0.87 (t, J = 6.7 Hz, 3H).
^{13}C NMR (75 MHz, $CDCl_3$) δ 176.30, 162.16, 148.84, 140.42, 136.88, 133.19, 128.47, 128.18, 122.40, 120.62, 80.75, 60.50, 51.39, 50.88, 44.21, 31.81, 31.45, 30.83, 29.47, 29.36, 29.19, 27.42, 22.63, 14.08.
HRMS-ESI (m/z): $[M+H]^+$ calcd for $C_{29}H_{42}ClN_2O_3^+$, 501.2879, found 501.2887.

methyl 2-((((3s,5s,7s)-adamantan-1-yl)amino)methyl)decanoate (3hh)

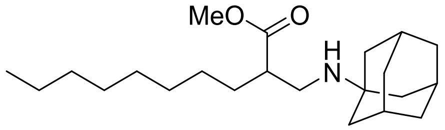

Colorless oil, 14% yield. ^{1}H NMR (300 MHz, $CDCl_3$) δ 3.68 (s, 3H), 2.82 (dd, J = 11.3, 8.8 Hz, 1H), 2.64 (dd, J = 11.3, 5.0 Hz, 1H), 2.51 – 2.40 (m, 1H), 2.04 (s, 3H), 1.68 – 1.55 (m, 12H), 1.50 – 1.44 (m, 3H), 1.28 – 1.22 (m, 12H), 0.87 (t, J = 6.7 Hz, 3H).
^{13}C NMR (75 MHz, $CDCl_3$) δ 176.52, 51.57, 50.47, 47.41, 42.88, 42.62, 36.90, 31.99, 30.71, 29.73, 29.54, 29.38, 27.52, 22.80, 14.25.
HRMS-ESI (m/z): $[M+H]^+$ calcd for $C_{22}H_{40}NO_2^+$, 350.3054, found 350.3059.

ethyl 2-(piperidin-1-ylmethyl)decanoate (**3ii**)

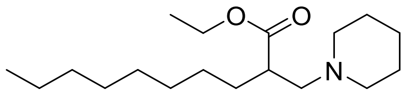

Colorless oil, 64% yield. ^{1}H NMR (300 MHz, $CDCl_3$) δ 4.14 (q, J = 7.1 Hz, 2H), 2.64 – 2.52 (m, 2H), 2.46 – 2.35 (m, 2H), 2.33 – 2.21 (m, 3H), 1.56 – 1.35 (m, 8H), 1.27 – 1.22 (m, 15H), 0.86 (t, J = 6.7 Hz, 3H).
^{13}C NMR (75 MHz, $CDCl_3$) δ 176.17, 61.70, 60.08, 54.77, 44.21, 31.98, 31.10, 29.65, 29.54, 29.36, 27.57, 26.16, 24.53, 22.79, 14.49, 14.23.
HRMS-EI: calcd for $C_{18}H_{35}NO_2$, 297.2662, found 297.2654.

propyl 2-(piperidin-1-ylmethyl)decanoate (**3jj**)

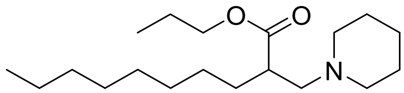

Colorless oil, 64% yield. ^{1}H NMR (300 MHz, $CDCl_3$) δ 4.04 (t, J = 6.6 Hz, 2H), 2.66 – 2.53 (m, 2H), 2.47 – 2.35 (m, 2H), 2.33 – 2.21 (m, 3H), 1.71 – 1.58 (m, 2H), 1.55 – 1.34 (m, 8H), 1.28 – 1.21 (m, 12H), 0.94 (t, J = 7.4 Hz, 3H), 0.86 (t, J = 6.7 Hz, 3H).
^{13}C NMR (75 MHz, $CDCl_3$) δ 176.26, 65.80, 61.76, 54.79, 44.31, 31.98, 31.15, 29.65, 29.55, 29.36, 27.60, 26.16, 24.54, 22.79, 22.23, 14.23, 10.62.
HRMS-EI: calcd for $C_{19}H_{37}NO_2$, 311.2819, found 311.2813.

butyl 2-(piperidin-1-ylmethyl)decanoate (**3kk**)

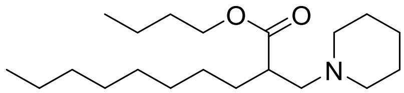

Colorless oil, 61% yield. ^{1}H NMR (400 MHz, $CDCl_3$) δ 4.08 (t, J = 6.5 Hz, 2H), 2.64 – 2.53 (m, 2H), 2.47 – 2.34 (m, 2H), 2.31 – 2.19 (m, 3H), 1.64 – 1.56 (m, 2H), 1.54 – 1.34 (m, 10H), 1.28 – 1.21 (m, 12H), 0.92 (t, J = 7.4 Hz, 3H), 0.86 (t, J = 6.9 Hz, 3H).

^{13}C NMR (101 MHz, $CDCl_3$) δ 176.26, 64.00, 61.77, 54.80, 44.30, 31.99, 31.16, 30.93, 29.65, 29.55, 29.36, 27.60, 26.16, 24.54, 22.79, 19.30, 14.23, 13.83.
HRMS-EI: calcd for $C_{20}H_{39}NO_2$, 325.2975, found 325.2967.

cyclohexylmethyl 2-(piperidin-1-ylmethyl)decanoate (**3ll**)

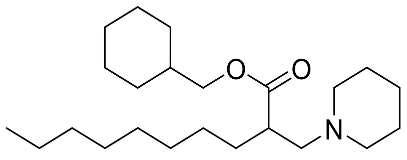

Colorless oil, 51% yield. ^{1}H NMR (400 MHz, $CDCl_3$) δ 3.95 – 3.83 (m, 2H), 2.68 – 2.51 (m, 2H), 2.49 – 2.34 (m, 2H), 2.33 – 2.20 (m, 3H), 1.78 – 1.61 (m, 6H), 1.54 – 1.35 (m, 8H), 1.28 – 1.19 (m, 15H), 1.02 – 0.91 (m, 2H), 0.87 (t, J = 6.9 Hz, 3H).
^{13}C NMR (101 MHz, $CDCl_3$) δ 176.26, 69.42, 61.83, 54.82, 44.37, 37.33, 32.00, 31.19, 29.84, 29.82, 29.65, 29.57, 29.37, 27.62, 26.55, 26.16, 25.85, 24.56, 22.80, 14.24.
HRMS-EI: calcd for $C_{23}H_{43}NO_2$, 365.3288, found 365.3283.

isopropyl 2-(piperidin-1-ylmethyl)decanoate (**3mm**)

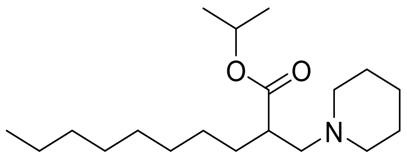

Colorless oil, 33% yield. ^{1}H NMR (300 MHz, $CDCl_3$) δ 5.15 – 4.94 (m, 1H), 2.62 – 2.50 (m, 2H), 2.47 – 2.35 (m, 2H), 2.33 – 2.20 (m, 3H), 1.55 – 1.35 (m, 8H), 1.27 – 1.21 (m, 18H), 0.87 (t, J = 6.7 Hz, 3H).
^{13}C NMR (75 MHz, $CDCl_3$) δ 175.67, 67.11, 61.77, 54.75, 44.35, 31.99, 31.12, 29.64, 29.56, 29.37, 27.53, 26.16, 24.55, 22.80, 22.03, 14.24.
HRMS-EI: calcd for $C_{19}H_{37}NO_2$, 311.2819, found 311.2814.

2,2,2-trifluoroethyl 2-(piperidin-1-ylmethyl)decanoate (**3nn**)

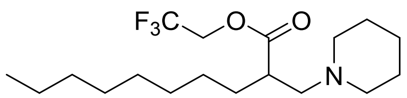

Colorless oil, 56% yield. ^{1}H NMR (300 MHz, $CDCl_3$) δ 4.58 – 4.36 (m, 2H), 2.79 – 2.68 (m, 1H), 2.64 – 2.55 (m, 1H), 2.49 – 2.35 (m, 2H), 2.34 – 2.20 (m, 3H), 1.63 – 1.44 (m, 6H), 1.43 – 1.34 (m, 2H), 1.29 – 1.22 (m, 12H), 0.87 (t, J = 6.7 Hz, 3H).
^{13}C NMR (75 MHz, $CDCl_3$) δ 174.47, 123.27 (q, J = 277.1 Hz), 61.51, 60.16 (q, J = 36.4 Hz), 54.85, 44.23, 31.97, 30.77, 29.56, 29.48, 29.32, 27.48, 26.12, 24.49, 22.79, 14.22.
^{19}F NMR (282 MHz, $CDCl_3$) δ -73.70 (t, J = 8.7 Hz).
HRMS-ESI (m/z): $[M+H]^+$ calcd for $C_{18}H_{33}F_3NO_2^+$, 352.2458, found 352.2468.

11-((1*R*,2*S*,5*R*)-2-isopropyl-5-methylcyclohexyl) 1-methyl 2-(piperidin-1-ylmethyl)undecanedioate (**3oo**)

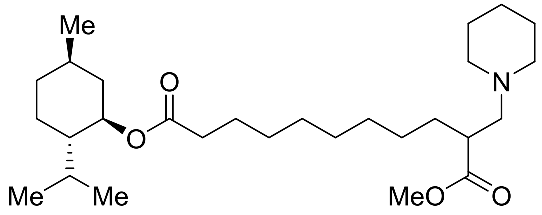

Colorless oil, 61% yield. ^{1}H NMR (300 MHz, $CDCl_3$) δ 4.65 (td, *J* = 10.9, 4.4 Hz, 1H), 3.65 (s, 3H), 2.66 – 2.50 (m, 2H), 2.43 – 2.32 (m, 2H), 2.30 – 2.20 (m, 5H), 2.00 – 1.91 (m, 1H), 1.85 (pd, *J* = 7.0, 2.7 Hz, 1H), 1.71 – 1.55 (m, 4H), 1.54 – 1.43 (m, 6H), 1.41 – 1.33 (m, 3H), 1.30 – 1.20 (m, 11H), 1.11 – 0.85 (m, 9H), 0.74 (d, *J* = 7.0 Hz, 3H).
^{13}C NMR (75 MHz, $CDCl_3$) δ 176.45, 173.38, 73.81, 61.45, 54.60, 51.31, 46.98, 43.98, 40.92, 34.70, 34.24, 31.33, 30.90, 29.40, 29.22, 29.12, 29.04, 27.41, 26.19, 26.03, 25.06, 24.35, 23.36, 21.99, 20.73, 16.24.
HRMS-ESI (m/z): $[M+H]^+$ calcd for $C_{28}H_{52}NO_4^+$, 466.3891, found 466.3893.

11-(3,7-dimethyloct-6-en-1-yl) 1-methyl 2-(piperidin-1-ylmethyl)undecanedioate (**3pp**)

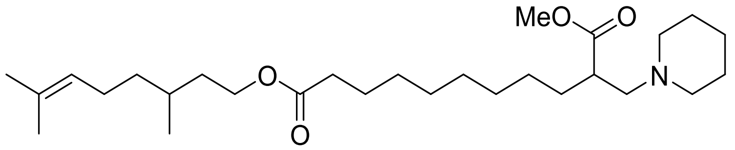

Colorless oil, 53% yield. ^{1}H NMR (300 MHz, $CDCl_3$) δ 5.07 (tp, *J* = 7.1, 1.4 Hz, 1H), 4.15 – 4.01 (m, 2H), 3.66 (s, 3H), 2.65 – 2.52 (m, 2H), 2.44 – 2.33 (m, 2H), 2.31 – 2.21 (m, 5H), 2.04 – 1.90 (m, 2H), 1.69 – 1.56 (m, 9H), 1.55 – 1.44 (m, 6H), 1.43 – 1.33 (m, 4H), 1.31 – 1.22 (m, 11H), 1.19 – 1.11 (m, 1H), 0.90 (d, *J* = 6.4 Hz, 3H).
^{13}C NMR (75 MHz, $CDCl_3$) δ 176.45, 173.90, 131.25, 124.54, 62.72, 61.45, 54.61, 51.31, 43.99, 36.93, 35.44, 34.36, 30.90, 29.45, 29.40, 29.20, 29.13, 29.07, 27.41, 26.03, 25.67, 25.35, 24.94, 24.35, 19.37, 17.60.
HRMS-ESI (m/z): $[M+H]^+$ calcd for $C_{28}H_{52}NO_4^+$, 466.3891, found 466.3890.

methyl 6-((2-(4-isobutylphenyl)propanoyl)oxy)-2-(piperidin-1-ylmethyl)hexanoate (**3qq**)

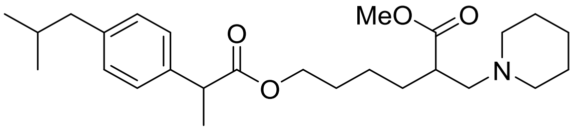

Colorless oil, 59% yield. ^{1}H NMR (300 MHz, $CDCl_3$) δ 7.23 – 7.15 (m, 2H), 7.11 – 7.05 (m, 2H), 4.03 (tt, *J* = 6.6, 1.5 Hz, 2H), 3.75 – 3.58 (m, 4H), 2.65 – 2.49 (m, 2H), 2.46 – 2.22 (m, 7H), 1.85 (dq, *J* = 13.9, 7.0 Hz, 1H), 1.60 – 1.45 (m, 10H), 1.43 – 1.35 (m, 2H), 1.31 – 1.19 (m, 3H), 0.89 (d, *J* = 6.6 Hz, 6H).

^{13}C NMR (75 MHz, $CDCl_3$) δ 176.15, 174.72, 140.41, 137.79, 129.24, 127.09, 64.39, 64.37, 61.30, 54.63, 51.38, 45.13, 45.01, 43.83, 30.35, 30.14, 28.38, 26.02, 24.33, 23.75, 23.73, 22.36, 18.47.
HRMS-ESI (m/z): $[M+H]^+$ calcd for $C_{26}H_{42}NO_4^+$, 432.3109, found 432.3115.

11-((3a*R*,5*R*,6*S*,6a*R*)-5-((*R*)-2,2-dimethyl-1,3-dioxolan-4-yl)-2,2-dimethyltetrahydrofuro[2,3-*d*][1,3]dioxol-6-yl) 1-methyl 2-(piperidin-1-ylmethyl)undecanedioate (3rr)

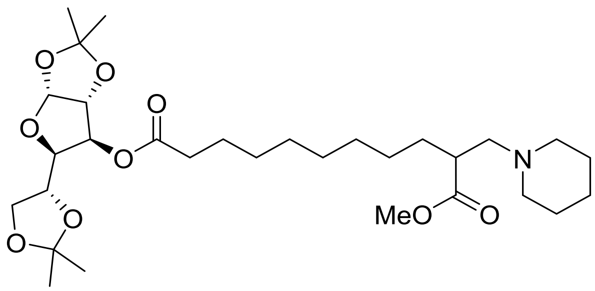

Colorless oil, 47% yield. ^1H NMR (300 MHz, $CDCl_3$) δ 5.85 (d, *J* = 3.7 Hz, 1H), 5.27 – 5.23 (m, 1H), 4.46 (dd, *J* = 3.7, 0.6 Hz, 1H), 4.21 – 4.17 (m, 2H), 4.10 – 4.04 (m, 1H), 4.03 – 3.96 (m, 1H), 3.66 (s, 3H), 2.66 – 2.51 (m, 2H), 2.43 – 2.21 (m, 7H), 1.66 – 1.45 (m, 11H), 1.41 – 1.33 (m, 5H), 1.31 – 1.23 (m, 16H).
^{13}C NMR (75 MHz, $CDCl_3$) δ 176.44, 172.31, 112.23, 109.26, 105.03, 83.37, 79.84, 75.79, 72.40, 67.24, 61.43, 54.60, 51.34, 43.97, 34.21, 30.91, 29.41, 29.18, 29.10, 28.96, 27.40, 26.78, 26.70, 26.17, 26.00, 25.23, 24.81, 24.33.
HRMS-ESI (m/z): $[M+H]^+$ calcd for $C_{30}H_{52}NO_9^+$, 570.3637, found 570.3642.

methyl 6-(2-(11-oxo-6,11-dihydrodibenzo[*b,e*]oxepin-2-yl)acetoxy)-2-(piperidin-1-ylmethyl)hexanoate (3ss)

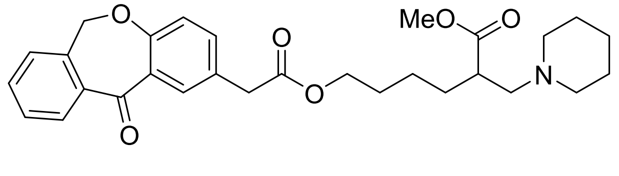

Colorless oil, 54% yield. ^1H NMR (300 MHz, $CDCl_3$) δ 8.10 (dd, *J* = 2.4, 0.5 Hz, 1H), 7.88 (ddd, *J* = 7.6, 1.5, 0.5 Hz, 1H), 7.58 – 7.51 (m, 1H), 7.49 – 7.39 (m, 2H), 7.37 – 7.32 (m, 1H), 7.02 (d, *J* = 8.3 Hz, 1H), 5.17 (s, 2H), 4.07 (t, *J* = 6.6 Hz, 2H), 3.65 (s, 3H), 3.62 (s, 2H), 2.68 – 2.51 (m, 2H), 2.45 – 2.20 (m, 5H), 1.68 – 1.43 (m, 8H), 1.40 – 1.26 (m, 4H).
^{13}C NMR (75 MHz, $CDCl_3$) δ 190.72, 176.09, 171.37, 160.40, 140.39, 136.26, 135.52, 132.69, 132.37, 129.44, 129.19, 127.85, 127.74, 125.07, 120.98, 73.57, 64.72, 61.26, 54.59, 51.39, 43.80, 40.19, 30.34, 28.39, 25.98, 24.29, 23.79.
HRMS-ESI (m/z): $[M+H]^+$ calcd for $C_{29}H_{36}NO_6^+$, 494.2538, found 494.2534.

methyl 6-((3-(4,5-diphenyloxazol-2-yl)propanoyl)oxy)-2-(piperidin-1-ylmethyl)hexanoate (3tt)

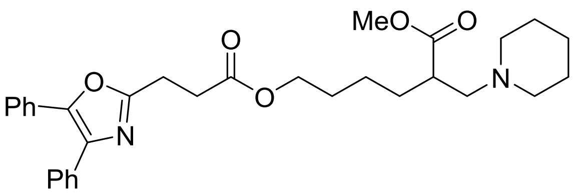

Colorless oil, 43% yield. ^{1}H NMR (300 MHz, $CDCl_3$) δ 7.66 – 7.60 (m, 2H), 7.59 – 7.54 (m, 2H), 7.39 – 7.28 (m, 6H), 4.10 (t, *J* = 6.6 Hz, 2H), 3.66 (s, 3H), 3.22 – 3.13 (m, 2H), 2.94 – 2.86 (m, 2H), 2.66 – 2.52 (m, 2H), 2.43 – 2.33 (m, 2H), 2.32 – 2.18 (m, 3H), 1.69 – 1.45 (m, 8H), 1.42 – 1.29 (m, 4H).
^{13}C NMR (75 MHz, $CDCl_3$) δ 176.12, 171.96, 161.71, 145.34, 135.08, 132.42, 128.94, 128.58, 128.49, 128.39, 127.99, 127.83, 126.41, 64.55, 61.28, 54.60, 51.40, 43.80, 31.11, 30.36, 28.43, 25.99, 24.30, 23.81, 23.51.
HRMS-ESI (m/z): $[M+H]^+$ calcd for $C_{31}H_{39}N_2O_5^+$, 519.2854, found 519.2862.

11-((3*S*,8*S*,9*S*,10*R*,13*R*,14*S*,17*R*)-10,13-dimethyl-17-((*R*)-6-methylheptan-2-yl)-2,3,4,7,8,9,10,11,12,13,14,15,16,17-tetradecahydro-1*H*-cyclopenta[a]phenanthren-3-yl) 1-methyl 2-(piperidin-1-ylmethyl)undecanedioate (**3uu**)

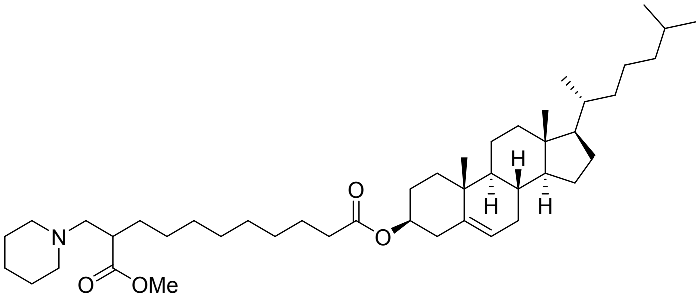

Colorless oil, 55% yield. ^{1}H NMR (300 MHz, $CDCl_3$) δ 5.40 – 5.32 (m, 1H), 4.67 – 4.52 (m, 1H), 3.66 (s, 3H), 2.66 – 2.53 (m, 2H), 2.45 – 2.19 (m, 9H), 2.04 – 1.90 (m, 2H), 1.89 – 1.75 (m, 3H), 1.64 – 1.43 (m, 14H), 1.42 – 1.24 (m, 17H), 1.17 – 0.93 (m, 13H), 0.90 (d, *J* = 6.6 Hz, 3H), 0.86 (d, *J* = 1.5 Hz, 3H), 0.84 (d, *J* = 1.3 Hz, 3H), 0.66 (s, 3H).
^{13}C NMR (75 MHz, $CDCl_3$) δ 176.43, 173.20, 139.66, 122.53, 73.62, 61.43, 56.65, 56.09, 54.59, 51.31, 49.99, 43.96, 42.27, 39.69, 39.48, 38.12, 36.96, 36.55, 36.14, 35.75, 34.65, 31.86, 31.82, 30.91, 29.40, 29.19, 29.12, 29.02, 28.19, 27.97, 27.77, 27.40, 26.01, 24.98, 24.34, 24.24, 23.79, 22.78, 22.53, 20.99, 19.28, 18.68, 11.81.
HRMS-ESI (m/z): $[M+H]^+$ calcd for $C_{45}H_{78}NO_4^+$, 696.5926, found 696.5931.

methyl 6-(((*tert*-butoxycarbonyl)-*L*-leucyl)oxy)-2-(piperidin-1-ylmethyl)hexanoate (**3vv**)

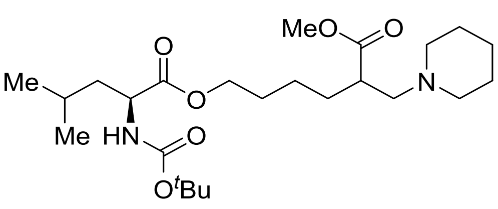

Colorless oil, 51% yield. ^{1}H NMR (300 MHz, CDCl$_3$) δ 4.91 (d, J = 8.3 Hz, 1H), 4.36 – 4.18 (m, 1H), 4.08 (t, J = 6.6 Hz, 2H), 3.66 (s, 3H), 2.67 – 2.51 (m, 2H), 2.44 – 2.20 (m, 5H), 1.73 – 1.55 (m, 4H), 1.54 – 1.28 (m, 20H), 0.93 (dd, J = 6.5, 2.2 Hz, 6H).
^{13}C NMR (75 MHz, CDCl$_3$) δ 176.14, 173.53, 155.39, 79.72, 64.94, 61.34, 54.67, 52.12, 51.45, 43.86, 41.93, 30.38, 28.41, 28.32, 26.04, 24.79, 24.35, 23.83, 22.81, 21.96.
HRMS-ESI (m/z): [M+H]$^+$ calcd for $C_{24}H_{45}N_2O_6^+$, 457.3273, found 457.3275.

3.5 Results for 1,2-amino-methoxycarbonylation of internal alkenes and styrenes

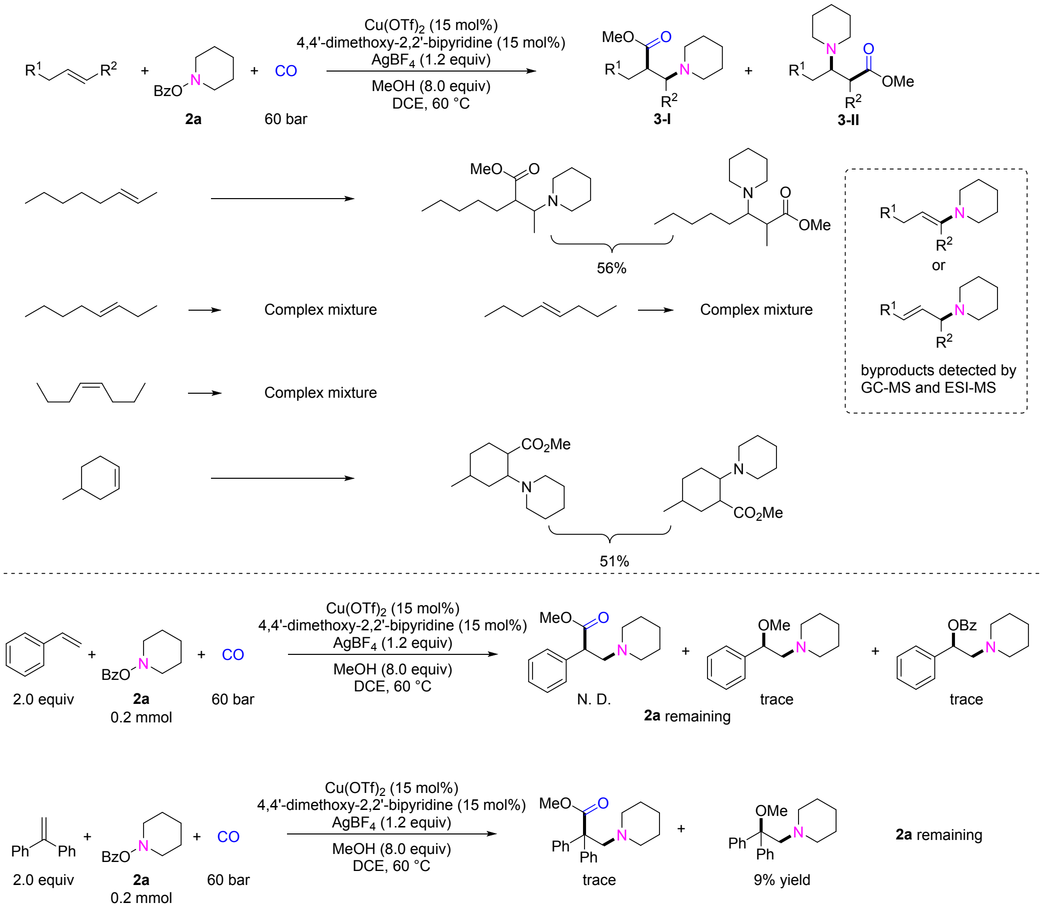

The internal alkenes underwent the desired transformation; however, mixtures of regio- and diastereomers were obtained, which are very difficult to separate. As an example, using trans-2-octene provided 4 diastereomers in 56% total yield. In case of trans-3-octene, trans-4-octene, and cis-4-octene lower reactivity was observed and additional side-products were detected.

3.6 Preliminary results for asymmetric 1,2-amino-methoxycarbonylation of 1-octene

The asymmetric 1,2-amino-methoxycarbonylation of 1-octene was carried out using five commercially available chiral nitrogen ligands. In general, all the ligands gave lower yields (< 20%) compared to the standard bipyridine derivatives. More importantly, no or very low

enantioselectivity was detected after establishing a suitable chiral analysis. The current conditions are not suitable for an asymmetric version.

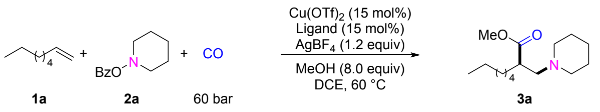

entry	Ligand	yield of **3a** (%)[a]	e. r. of **3a** (%)[b]
1	**L1**	19	50.5:49.5
2	**L2**	10	Not determined
3	**L3**	19	50:50
4	**L4**	19	52:48
5	**L5**	8	Not determined

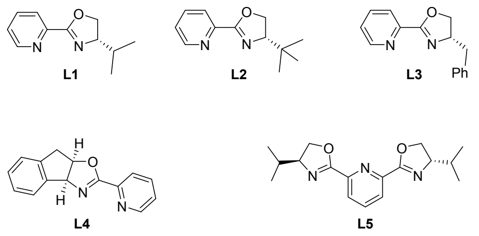

3.7 Stereochemistry of major diastereomer of product 3v

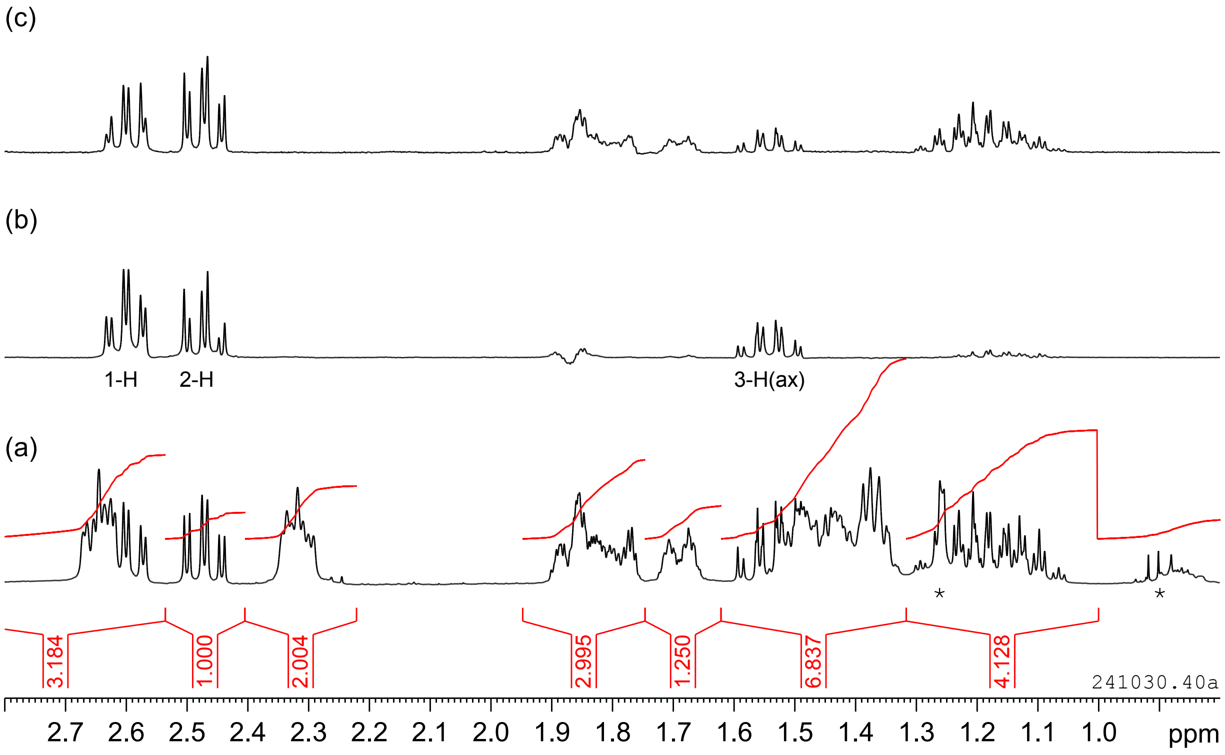

Figure S1. ^{1}H NMR spectra (400.13 MHz, $CDCl_3$) of product **3v**. (a) Standard spectrum showing the region of cyclohexane and piperidine protons; * denotes impurities. (b) TOCSY spectrum with selective excitation at 2.47 ppm (2-H), mixing time 15 ms, showing transfer to the next neighbours of this atom; ^3J(1-H,2-H) amounts to 11 to 11.5 Hz. (c) TOCSY spectrum with selective excitation at 2.47 ppm (2-H), mixing time 45 ms, showing transfer to the entire cyclohexane spin system.

The well-known relation for the chemical shifts of H atoms at the "chair" cyclohexane, $\delta(H_{ax}) < \delta(H_{eq})$, was used to assist assignment. The signals of these protons also exhibit characteristic shapes. H_{ax} appear quartet-like, the shape is dominated by the three large coupling constants $^3J_{trans}$ (occurring twice, magnitude about 11 Hz) and ^2J (occurring once, of similar magnitude) whereas the two couplings $^3J_{gauche}$ are small. H_{eq} appear doublet like, the shape is dominated by the large

^2J, and the four ^3J are only small. For product **3v**, there are four CH_2 groups represented as described above (one "doublet" and one "quartet" each). The two CH groups are represented by two triplets of doublets with a triplet splitting of 11-11.5 Hz and a doublet splitting of 3-3.5 Hz (Figure S1).

One of the large couplings has been proven to be ^3J across the C1-C2 bond. This means that each of the CH groups must have one H of the attached CH_2 groups in a *trans* (the second large coupling) and the other one in a *gauche* orientation (the small coupling). This is only possible if the two substituents reside on opposite sides of the cyclohexane plane (Figure S2). As usual, the preferred conformation is the one with the large groups in equatorial positions which leads to an orientation of the cyclohexane hydrogen atoms as derived from the coupling pattern.

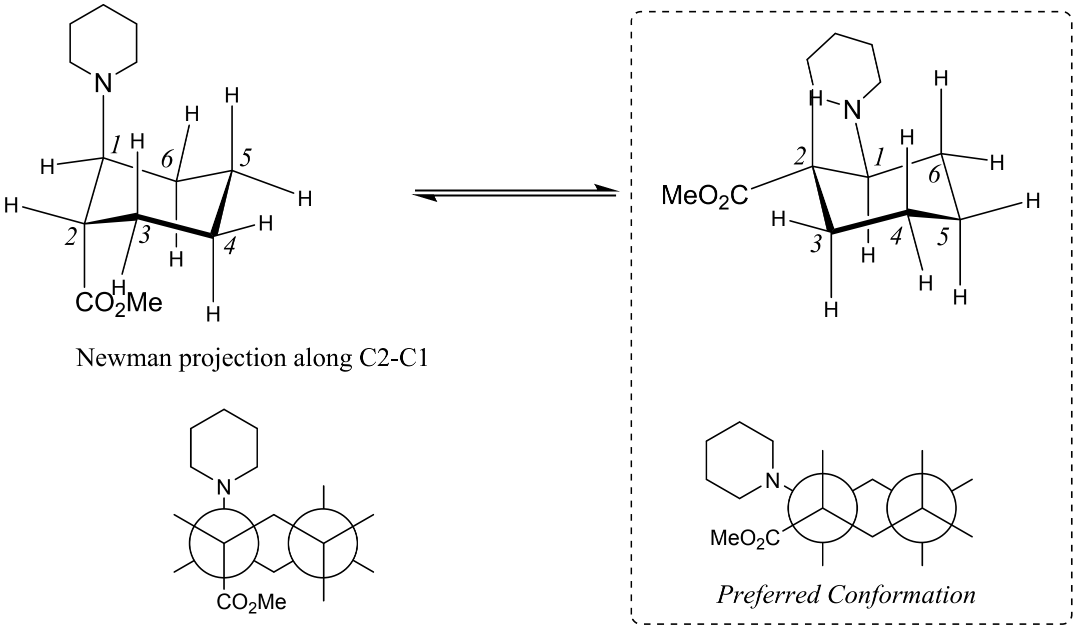

Figure S2. Transoid arrangement of the substituents in product **3v**.

When product **3v** was treated with $PhCO_2H$, a co-crystal of **3v**-acid and $PhCO_2H$ was obtained. X-ray crystal structure analysis of co-crystal of **3v**-acid and $PhCO_2H$

Diffraction data were collected on a Bruker Kappa APEX II Duo diffractometer. The structure was solved by intrinsic phasing (SHELXT: Sheldrick, G. M. *Acta Cryst.* **2015**, *A71*, 3.) and refined by full-matrix least-squares procedures on F^2 (SHELXL: Sheldrick, G. M. *Acta Cryst.* **2015**, *C71*, 3.). XP (Bruker AXS) was used for graphical representation.

CCDC 2405837 contains the supplementary crystallographic data for this paper. These data are provided free of charge by the joint Cambridge Crystallographic Data Centre and Fachinformationszentrum Karlsruhe Access Structures service www.ccdc.cam.ac.uk/structures.

Crystal data of co-crystal of **3v**-acid and $PhCO_2H$: $C_{19}H_{27}NO_4$, M = 333.41, monoclinic, space group $P2_1/c$, a = 10.7164(10), b = 15.0823(14), c = 11.7145(10) Å, b = 114.5998(13)°, V =

1721.5(3) Å^3, $T = 150(2)$ K, $Z = 4$, 29394 reflections measured, 4668 independent reflections (R_{int} = 0.029), final R values ($I > 2\sigma(I)$): $R_1 = 0.0449$, $wR_2 = 0.1130$, final R values (all data): R_1 = 0.0590, $wR_2 = 0.1211$, 282 parameters. 62 restraints are used to improve the geometry of the disordered part of the molecular structure. Lower occupied atoms are refined isotropically.

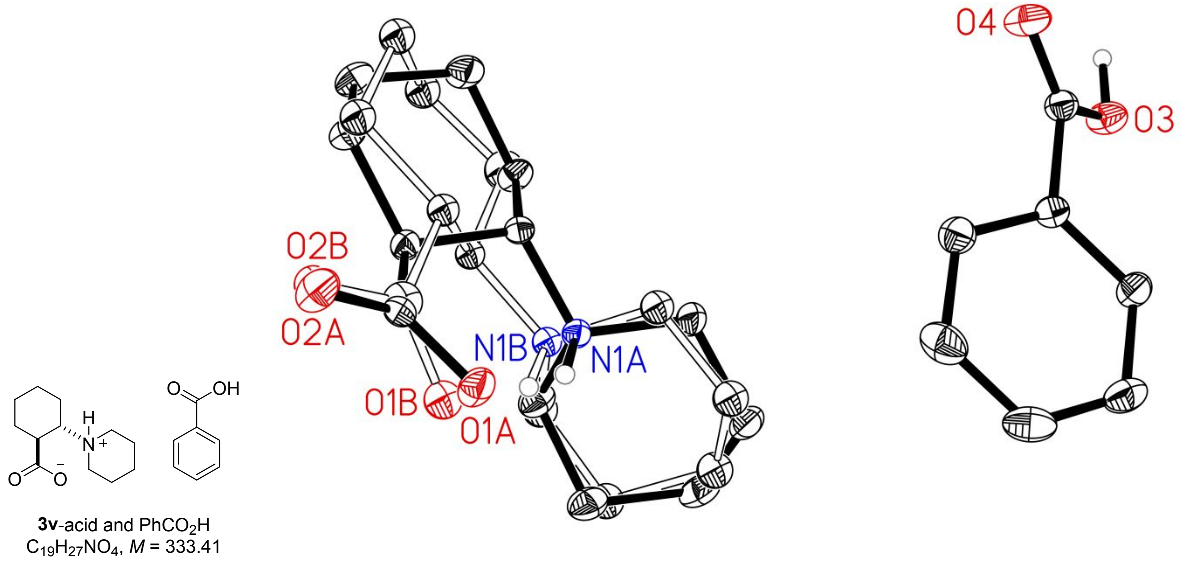

Figure S3. Molecular structure of co-crystal of **3v**-acid and $PhCO_2H$. Displacement ellipsoids correspond to 30% probability. C-bound hydrogen atoms are omitted for clarity. Lower occupied parts of disorder are shown with unfilled lines.

3.8 General procedure for acyloxy-amination of non-activated alkenes

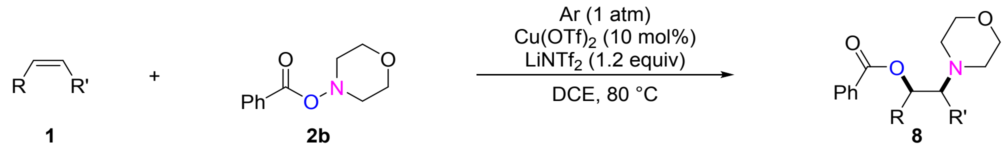

A 30 mL Schlenk tube containing a stirring bar was charged with **2b** (62 mg, 0.3 mmol, 1.5 equiv). The reaction tube was transferred to the glovebox and charged with copper(II) triflate (7.2 mg, 0.02 mmol, 10 mol%) and lithium bis(trifluoromethanesulfonyl)imide (69 mg, 0.24 mmol, 1.2 equiv). After closed, the reaction tube was moved out of the glovebox and the tube was then evacuated and back-filled with argon for 3 times. Subsequently, anhydrous DCE (2 mL) was added followed by **1** (0.2 mmol, 1.0 equiv) via syringe under argon. Once added, the Schlenk tube was sealed at atmospheric pressure of argon (1 atm). The reaction was heated to 80 ˚C and stirred for 20 h. Afterwards, the tube was cooled to room temperature. Then, DMAP (36.7 mg, 0.3 mmol, 1.5 equiv) were added to the reaction mixture and stirred at room temperature for 10 min. Pure products were obtained by column chromatography on silica gel visualized by I_2.

2-morpholinocyclopentyl benzoate (8a)

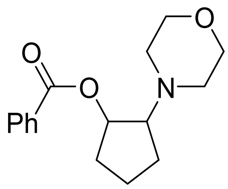

Colorless oil, 68% yield. ^1H NMR (300 MHz, acetone-d_6) δ 8.06 – 7.97 (m, 2H), 7.67 – 7.58 (m, 1H), 7.54 – 7.46 (m, 2H), 5.32 – 5.24 (m, 1H), 3.58 (t, *J* = 4.7 Hz, 4H), 2.88 (ddd, *J* = 8.5, 7.4, 4.7 Hz, 1H), 2.55 – 2.45 (m, 4H), 2.19 – 2.06 (m, 1H), 2.02 – 1.94 (m, 1H), 1.79 – 1.67 (m, 3H), 1.63 – 1.50 (m, 1H).
^{13}C NMR (75 MHz, acetone-d_6) δ 165.94, 133.57, 131.27, 129.87, 129.11, 78.95, 72.33, 67.24, 52.58, 32.67, 22.68.
HRMS-ESI (m/z): $[M+H]^+$ calcd for $C_{16}H_{22}NO_3^+$, 276.1595, found 276.1602.

2-(2,6-dimethylmorpholino)cyclopentyl benzoate (**8b**)

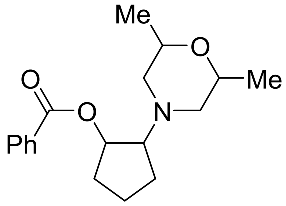

Colorless oil, 65% yield. ^1H NMR (300 MHz, $CDCl_3$) δ 8.09 – 7.96 (m, 2H), 7.63 – 7.51 (m, 1H), 7.50 – 7.33 (m, 2H), 5.34 (ddd, *J* = 7.5, 4.6, 3.0 Hz, 1H), 3.73 – 3.57 (m, 2H), 2.94 – 2.74 (m, 3H), 2.22 – 1.97 (m, 2H), 1.87 – 1.71 (m, 5H), 1.64 – 1.49 (m, 1H), 1.15 (d, *J* = 6.3 Hz, 3H), 1.10 (d, *J* = 6.3 Hz, 3H).
^{13}C NMR (75 MHz, $CDCl_3$) δ 165.99, 132.89, 130.51, 129.49, 128.34, 78.59, 71.68, 71.60, 71.58, 57.83, 57.77, 32.58, 29.07, 22.54, 19.23, 19.18.
HRMS-ESI (m/z): $[M+H]^+$ calcd for $C_{18}H_{26}NO_3^+$, 304.1908, found 304.1914.

4-(methoxycarbonyl)-2-morpholinocyclopentyl benzoate (**8c**)

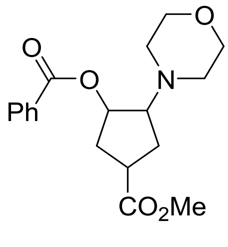

Colorless oil, 61% yield. ^1H NMR (300 MHz, $CDCl_3$) δ 8.08 – 7.95 (m, 2H), 7.65 – 7.51 (m, 1H), 7.51 – 7.38 (m, 2H), 5.43 (ddd, *J* = 7.1, 4.2, 2.5 Hz, 1H), 3.74 – 3.63 (m, 7H), 3.12 – 2.88 (m, 2H), 2.66 – 2.46 (m, 4H), 2.44 – 2.26 (m, 2H), 2.12 – 1.99 (m, 1H), 1.93 – 1.84 (m, 1H).
^{13}C NMR (75 MHz, $CDCl_3$) δ 174.69, 165.62, 133.13, 130.09, 129.55, 128.42, 77.07, 71.61, 66.91, 51.91, 40.83, 35.82, 32.59.
HRMS-ESI (m/z): $[M+H]^+$ calcd for $C_{18}H_{24}NO_5^+$, 334.1649, found 334.1657.

diethyl 3-(benzoyloxy)-4-morpholinocyclopentane-1,1-dicarboxylate (8d)

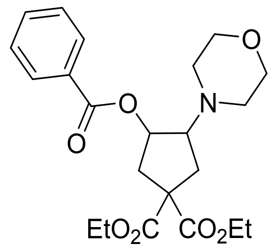

Colorless oil, 41% yield. ^{1}H NMR (300 MHz, $CDCl_3$) δ 8.09 – 7.92 (m, 2H), 7.62 – 7.51 (m, 1H), 7.49 – 7.38 (m, 2H), 5.49 – 5.36 (m, 1H), 4.28 – 4.07 (m, 4H), 3.67 (t, *J* = 4.7 Hz, 4H), 3.10 (ddd, *J* = 8.9, 7.3, 4.5 Hz, 1H), 2.88 (dd, *J* = 14.9, 7.4 Hz, 1H), 2.74 (ddd, *J* = 13.4, 7.3, 1.5 Hz, 1H), 2.66 – 2.48 (m, 4H), 2.47 – 2.37 (m, 1H), 2.14 (dd, *J* = 13.4, 8.9 Hz, 1H), 1.25 (t, *J* = 7.1 Hz, 3H), 1.17 (t, *J* = 7.1 Hz, 3H).

^{13}C NMR (75 MHz, $CDCl_3$) δ 171.39, 170.98, 165.62, 133.15, 129.97, 129.59, 128.40, 76.37, 70.11, 66.94, 61.82, 61.74, 57.59, 51.57, 38.94, 35.43, 14.01, 13.90.

HRMS-ESI (m/z): $[M+H]^+$ calcd for $C_{22}H_{30}NO_7^+$, 420.2017, found 420.2030.

2-morpholinocyclohexyl benzoate (8e)

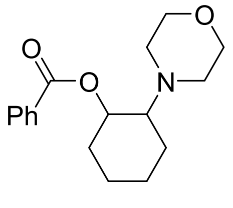

Colorless oil, 50% yield. ^{1}H NMR (300 MHz, $CDCl_3$) δ 8.12 – 7.97 (m, 2H), 7.60 – 7.50 (m, 1H), 7.49 – 7.38 (m, 2H), 5.09 (td, *J* = 10.4, 4.6 Hz, 1H), 3.49 (t, *J* = 4.6 Hz, 4H), 2.77 – 2.68 (m, 2H), 2.54 – 2.42 (m, 3H), 2.17 – 2.06 (m, 1H), 1.96 – 1.85 (m, 1H), 1.84 – 1.72 (m, 2H), 1.58 – 1.44 (m, 1H), 1.39 – 1.25 (m, 3H).

^{13}C NMR (75 MHz, $CDCl_3$) δ 166.11, 132.54, 131.18, 129.50, 128.23, 72.38, 67.65, 67.54, 49.45, 31.68, 24.91, 24.89, 24.27.

HRMS-ESI (m/z): $[M+H]^+$ calcd for $C_{17}H_{24}NO_3^+$, 290.1751, found 290.1757.

2-morpholinodecyl benzoate (8f-1) and **1-morpholinodecan-2-yl benzoate (8f-2)**

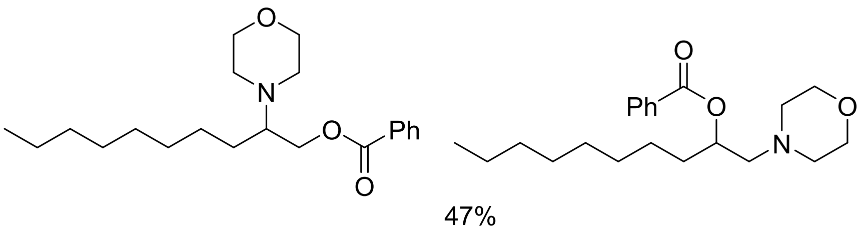

Colorless oil, 47% total yield, 1.2 : 1. ^{1}H NMR (300 MHz, $CDCl_3$) δ 8.09 – 7.99 (m, 4.3H), 7.60 – 7.50 (m, 2.2H), 7.49 – 7.39 (m, 4.4H), 5.28 (tt, J = 7.2, 4.9 Hz, 1H), 4.51 – 4.39 (m, 1.2H), 4.35 – 4.26 (m, 1.2H), 3.72 – 3.59 (m, 9H), 2.86 – 2.69 (m, 3.8H), 2.68 – 2.53 (m, 5.4H), 2.52 – 2.40 (m, 3H), 1.82 – 1.20 (m, 33H), 0.91 – 0.82 (m, 6.8H).
^{13}C NMR (75 MHz, $CDCl_3$) δ 166.47, 166.21, 132.93, 132.72, 130.75, 130.25, 129.54, 129.51, 128.39, 128.29, 71.90, 67.66, 67.03, 64.29, 62.78, 62.24, 54.07, 49.61, 32.76, 31.84, 31.80, 29.66, 29.49, 29.41, 29.24, 29.18, 28.34, 26.67, 25.32, 22.64, 22.61, 14.09, 14.06.
HRMS-ESI (m/z): $[M+H]^+$ calcd for $C_{21}H_{34}NO_3^+$, 348.2534, found 348.2542.

3-cyclohexyl-2-morpholinopropyl benzoate (8g-1) and **1-cyclohexyl-3-morpholinopropan-2-yl benzoate (8g-2)**

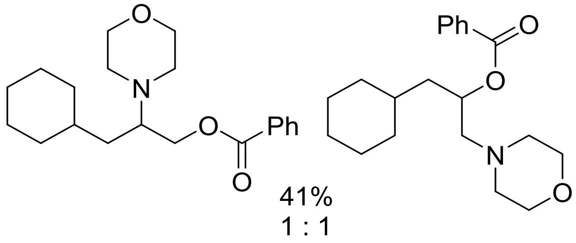

Colorless oil, 41% total yield, 1: 1. ^{1}H NMR (300 MHz, $CDCl_3$) δ 8.15 – 7.92 (m, 4H), 7.61 – 7.51 (m, 2H), 7.49 – 7.37 (m, 4H), 5.39 (ddt, J = 8.5, 7.1, 4.7 Hz, 1H), 4.49 – 4.23 (m, 2H), 3.72 – 3.59 (m, 8H), 2.96 (qd, J = 7.0, 4.4 Hz, 1H), 2.81 – 2.70 (m, 2H), 2.67 – 2.53 (m, 5H), 2.50 – 2.40 (m, 3H), 1.89 – 1.55 (m, 13H), 1.53 – 1.36 (m, 3H), 1.28 – 1.12 (m, 6H), 1.02 – 0.83 (m, 4H).
^{13}C NMR (75 MHz, $CDCl_3$) δ 166.49, 166.16, 132.92, 132.69, 130.82, 130.28, 129.55, 129.52, 128.40, 128.29, 69.74, 67.71, 67.04, 64.57, 62.81, 59.87, 54.06, 49.48, 40.48, 35.89, 34.51, 34.17, 33.90, 33.65, 33.34, 32.90, 26.58, 26.44, 26.30, 26.27, 26.22, 26.08.
HRMS-ESI (m/z): $[M+H]^+$ calcd for $C_{19}H_{28}NO_3^+$, 318.2064, found 318.2072.

4. Mechanistic studies

4.1 Control experiments in the presence of TEMPO

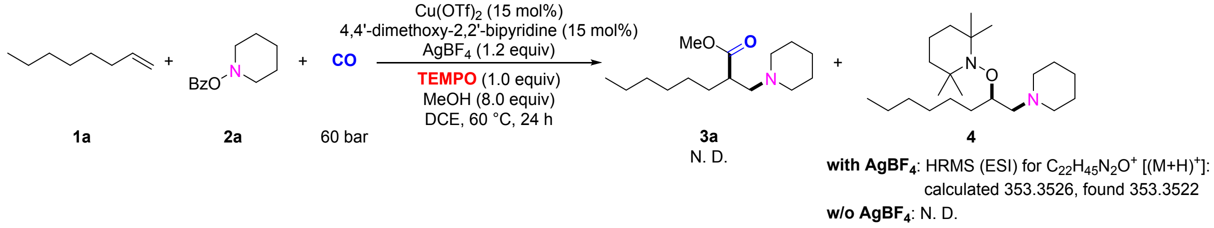

Procedures: An 8 mL screw-cap vial containing a stirring bar was charged with **2a** (0.2 mmol, 1.0 equiv), 4,4′-dimethoxy-2,2′-bipyridine (6.5 mg, 0.03 mmol, 15 mol%) and 2,2,6,6-tetramethyl-1-piperidyloxy (TEMPO) (31 mg, 0.2 mmol, 1.0 equiv). The vial was closed by PTFE/white rubber septum (Wheaton 15 mm Septa) and phenolic cap. The reaction vial was then transferred to the glovebox and charged with copper triflate (10.8 mg, 0.03 mmol, 15 mol%) and $AgBF_4$ (46.7 mg, 0.24 mmol, 1.2 equiv). After closed, the reaction vial was moved out of the glovebox and

connected with atmosphere with a needle. The vial was evacuated under vacuum and recharged with argon for three times. Subsequently, anhydrous DCE (2 mL) was added followed by **1a** (0.4 mmol, 2.0 equiv) and MeOH (65 µL, 1.6 mmol, 8.0 equiv) via syringe under argon. The vial was fixed in an alloy plate and put into a Parr 4560 series autoclave (300 mL) under argon atmosphere. At room temperature, the autoclave was flushed with nitrogen for two times and then carbon monoxide for three times. After that, carbon monoxide was charged to 60 bar. The reaction was heated to 60 ˚C and stirred for 24 h. Afterwards, the autoclave was cooled to room temperature and the pressure was carefully released. A sample of the reaction mixture was analyzed by HRMS.

4.2 Control experiments in the presence of BHT

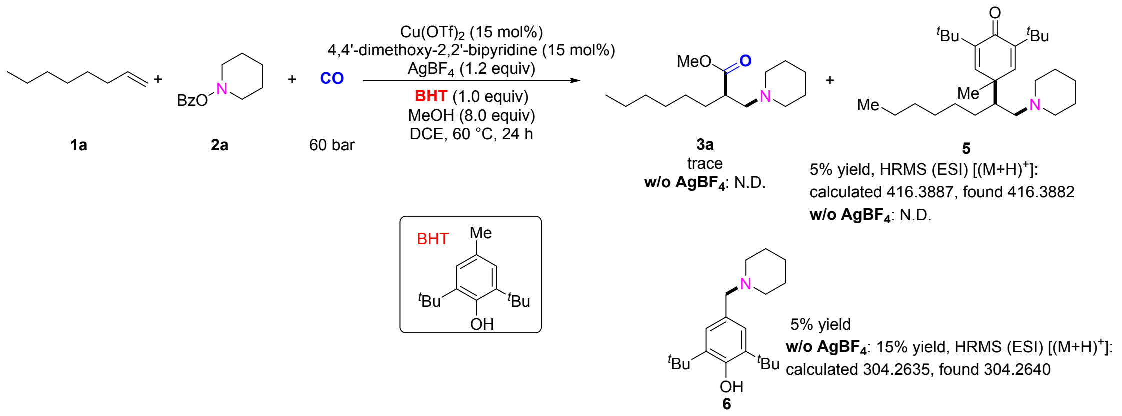

Procedures: An 8 mL screw-cap vial containing a stirring bar was charged with **2a** (0.2 mmol, 1.0 equiv), 4,4′-dimethoxy-2,2′-bipyridine (6.5 mg, 0.03 mmol, 15 mol%) and butylated hydroxytoluene (BHT) (44.1 mg, 0.2 mmol, 1.0 equiv). The vial was closed by PTFE/white rubber septum (Wheaton 15 mm Septa) and phenolic cap. The reaction vial was then transferred to the glovebox and charged with copper triflate (10.8 mg, 0.03 mmol, 15 mol%) and $AgBF_4$ (46.7 mg, 0.24 mmol, 1.2 equiv). After closed, the reaction vial was moved out of the glovebox and connected with atmosphere with a needle. The vial was evacuated under vacuum and recharged with argon for three times. Subsequently, anhydrous DCE (2 mL) was added followed by **1a** (0.4 mmol, 2.0 equiv) and MeOH (65 µL, 1.6 mmol, 8.0 equiv) via syringe under argon. The vial was fixed in an alloy plate and put into a Parr 4560 series autoclave (300 mL) under argon atmosphere. At room temperature, the autoclave was flushed with nitrogen for two times and then carbon monoxide for three times. After that, carbon monoxide was charged to 60 bar. The reaction was heated to 60 ˚C and stirred for 24 h. Afterwards, the autoclave was cooled to room temperature and the pressure was carefully released. A sample of the reaction mixture was analyzed by HRMS. Product **5** and **6** could be obtained by column chromatography on silica gel visualized by I_2.

2,6-di-*tert*-butyl-4-methyl-4-(1-(piperidin-1-yl)octan-2-yl)cyclohexa-2,5-dien-1-one (5)

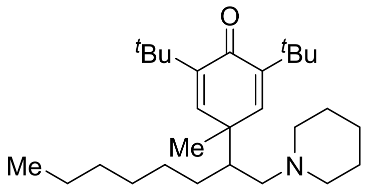

Colorless oil. 5% yield. ^{1}H NMR (300 MHz, $CDCl_3$) δ 6.55 (d, J = 2.9 Hz, 1H), 6.48 (d, J = 2.9 Hz, 1H), 2.36 – 2.15 (m, 4H), 2.06 (dd, J = 12.9, 5.6 Hz, 1H), 1.86 (dd, J = 12.8, 5.7 Hz, 1H), 1.65 – 1.46 (m, 6H), 1.44 – 1.35 (m, 4H), 1.32 – 1.25 (m, 7H), 1.23 (s, 9H), 1.23 (s, 9H), 1.17 (s, 3H), 0.88 (t, J = 6.8 Hz, 3H).
^{13}C NMR (75 MHz, $CDCl_3$) δ 186.75, 147.51, 146.47, 145.64, 144.99, 61.60, 55.14, 45.34, 42.72, 34.74, 34.64, 31.82, 30.42, 29.56, 29.53, 29.46, 28.83, 26.17, 24.91, 24.56, 22.65, 14.09.
HRMS-ESI (m/z): $[M+H]^+$ calcd for $C_{28}H_{50}NO^+$, 416.3887, found 416.3882.

2,6-di-tert-butyl-4-(piperidin-1-ylmethyl)phenol (6)

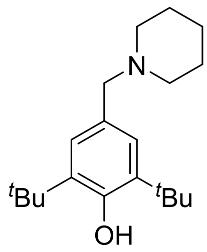

Colorless oil. 15% yield. ^{1}H NMR (300 MHz, $CDCl_3$) δ 7.13 (s, 2H), 5.18 (s, 1H), 3.64 (s, 2H), 2.61 (s, 4H), 1.70 (p, J = 5.6 Hz, 4H), 1.52 – 1.44 (m, 2H), 1.41 (s, 18H).
^{13}C NMR (75 MHz, $CDCl_3$) δ 153.49, 135.77, 129.65, 126.99, 62.85, 53.41, 34.39, 30.43, 24.95, 23.90.
HRMS-ESI (m/z): $[M+H]^+$ calcd for $C_{20}H_{34}NO^+$, 304.2635, found 304.2640. The spectral data are consistent with those previously reported in the literature.[5]

4.3 Radical clock experiment

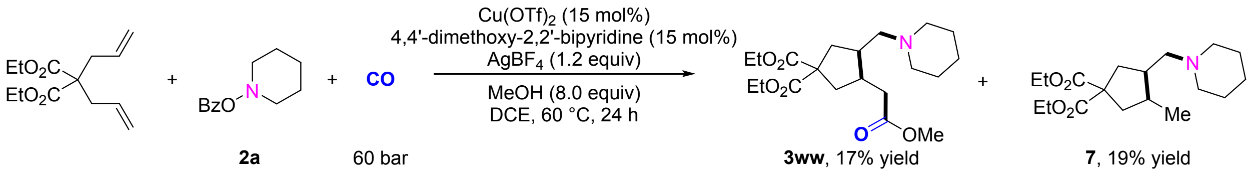

2a 60 bar **3ww**, 17% yield **7**, 19% yield

Procedures: An 8 mL screw-cap vial containing a stirring bar was charged with **2a** (0.2 mmol, 1.0 equiv) and 4,4′-dimethoxy-2,2′-bipyridine (6.5 mg, 0.03 mmol, 15 mol%). The vial was closed by PTFE/white rubber septum (Wheaton 15 mm Septa) and phenolic cap. The reaction vial was then transferred to the glovebox and charged with copper triflate (10.8 mg, 0.03 mmol, 15 mol%) and $AgBF_4$ (46.7 mg, 0.24 mmol, 1.2 equiv). After closed, the reaction vial was moved out of the glovebox and connected with atmosphere with a needle. The vial was evacuated under vacuum and recharged with argon for three times. Subsequently, anhydrous DCE (2 mL) was added followed by **1b** (0.4 mmol, 2.0 equiv) and MeOH (65 μL, 1.6 mmol, 8.0 equiv) via syringe under argon. The vial was fixed in an alloy plate and put into a Parr 4560 series autoclave (300 mL) under argon atmosphere. At room temperature, the autoclave was flushed with nitrogen for two times and then carbon monoxide for three times. After that, carbon monoxide was charged to 60 bar. The reaction was heated to 60 °C and stirred for 24 h. Afterwards, the autoclave was cooled

to room temperature and the pressure was carefully released. The mixture of compounds **3ww** and **7** could be obtained by column chromatography on silica gel visualized by I_2.

diethyl 3-(2-methoxy-2-oxoethyl)-4-(piperidin-1-ylmethyl)cyclopentane-1,1-dicarboxylate (3ww) and **diethyl 3-methyl-4-(piperidin-1-ylmethyl)cyclopentane-1,1-dicarboxylate (7)**

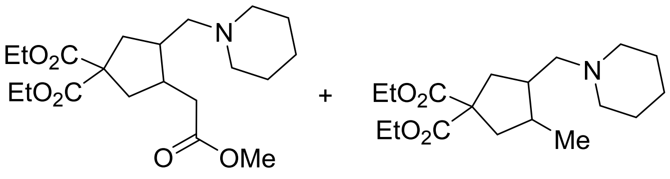

Colorless oil. ^{1}H NMR (300 MHz, $CDCl_3$) δ 4.21 – 4.10 (m, 8.6H), 3.65 (s, 3H for **3ww**), 2.58 – 2.47 (m, 2.2H), 2.44 – 2.13 (m, 19.8H), 2.09 – 1.97 (m, 4.7H), 1.49 (dq, *J* = 11.0, 5.8 Hz, 8.3H), 1.42 – 1.32 (m, 4.2H), 1.21 (td, *J* = 7.1, 0.8 Hz, 14.2H), 0.84 (d, *J* = 6.6 Hz, 3.5H for **7**).
^{13}C NMR (75 MHz, $CDCl_3$) δ 173.45, 173.08, 172.88, 172.65, 172.57, 61.36, 61.22, 61.19, 59.39, 59.30, 58.84, 58.59, 54.88, 54.77, 51.36, 41.43, 39.59, 38.88, 37.91, 37.87, 37.51, 37.46, 35.35, 34.02, 26.02, 25.97, 24.49, 14.83, 13.97.
HRMS-ESI (m/z): **3ww**, $[M+H]^+$ calcd for $C_{20}H_{34}NO_6^+$, 384.2381, found 384.2388; **7**, $[M+H]^+$ calcd for $C_{18}H_{32}NO_4^+$, 326.2326, found 326.2333.

4.4 Control experiment using LiOMe instead of MeOH

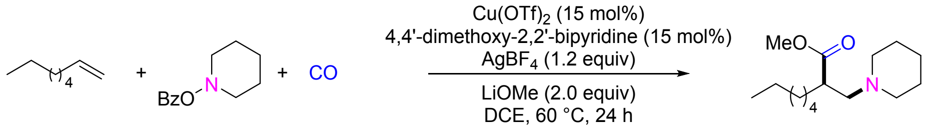

Procedures: An 8 mL screw-cap vial containing a stirring bar was charged with **2a** (0.2 mmol, 1.0 equiv) and 4,4′-dimethoxy-2,2′-bipyridine (6.5 mg, 0.03 mmol, 15 mol%). The vial was closed by PTFE/white rubber septum (Wheaton 15 mm Septa) and phenolic cap. The reaction vial was then transferred to the glovebox and charged with copper triflate (10.8 mg, 0.03 mmol, 15 mol%), $AgBF_4$ (46.7 mg, 0.24 mmol, 1.2 equiv) and LiOMe (15.2 mg, 0.4 mmol, 2.0 equiv). After closed, the reaction vial was moved out of the glovebox and connected with atmosphere with a needle. The vial was evacuated under vacuum and recharged with argon for three times. Subsequently, anhydrous DCE (2 mL) was added followed by **1a** (0.4 mmol, 2.0 equiv) via syringe under argon. The vial was fixed in an alloy plate and put into a Parr 4560 series autoclave (300 mL) under argon atmosphere. At room temperature, the autoclave was flushed with nitrogen for two times and then carbon monoxide for three times. After that, carbon monoxide was charged to 60 bar. The reaction was heated to 60 ˚C and stirred for 24 h. Afterwards, the autoclave was cooled to room temperature and the pressure was carefully released. Then DMAP (36.7 mg, 0.3 mmol, 1.5 equiv) was added to the reaction mixture and stirred at room temperature for 4 h. Pure product could be obtained by column chromatography on silica gel visualized by I_2.

4.5 Reaction monitorization and control experiment

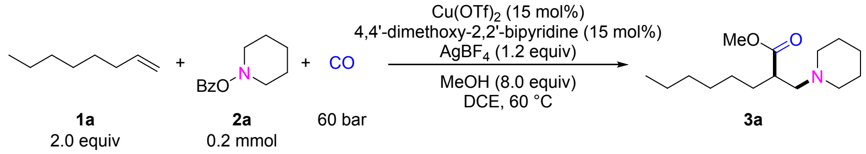

entry	reacton time	GC yield of **3a** (%)
1	3 h	27
2	8 h	53
3	14 h	65
4	24 h (**2a** full conversion)	79

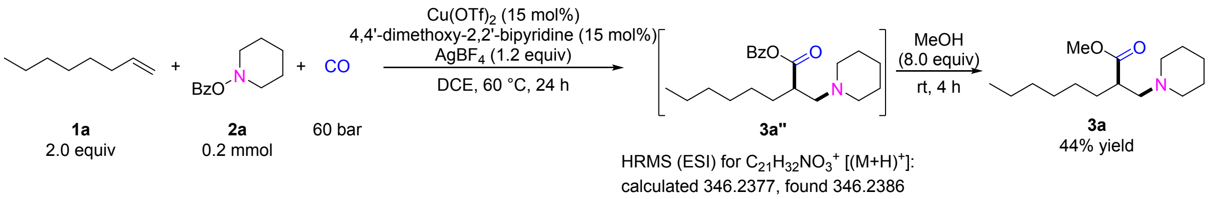

Procedures: An 8 mL screw-cap vial containing a stirring bar was charged with **2a** (0.2 mmol, 1.0 equiv) and 4,4′-dimethoxy-2,2′-bipyridine (6.5 mg, 0.03 mmol, 15 mol%). The vial was closed by PTFE/white rubber septum (Wheaton 15 mm Septa) and phenolic cap. The reaction vial was then transferred to the glovebox and charged with copper triflate (10.8 mg, 0.03 mmol, 15 mol%), $AgBF_4$ (46.7 mg, 0.24 mmol, 1.2 equiv). After closed, the reaction vial was moved out of the glovebox and connected with atmosphere with a needle. The vial was evacuated under vacuum and recharged with argon for three times. Subsequently, anhydrous DCE (2 mL) was added followed by **1a** (0.4 mmol, 2.0 equiv) via syringe under argon. The vial was fixed in an alloy plate and put into a Parr 4560 series autoclave (300 mL) under argon atmosphere. At room temperature, the autoclave was flushed with nitrogen for two times and then carbon monoxide for three times. After that, carbon monoxide was charged to 60 bar. The reaction was heated to 60 ˚C and stirred for 24 h. Afterwards, the autoclave was cooled to room temperature and the pressure was carefully released. A sample of the reaction mixture in anhydrous acetonitrile was analyzed by HRMS. Then MeOH (65 µL, 1.6 mmol, 8.0 equiv) was added to the reaction mixture and stirred at room temperature for 4 h. Pure product could be obtained by column chromatography on silica gel visualized by I_2. (3a’’ is highly unstable and we were only able to determine it qualitatively by HRMS analysis of the reaction mixture. Isolation or detection of 3a’’ by GC, LC or NMR analysis failed.)

5. References

(1) Berman, A. M.; Johnson, J. S. Copper-Catalyzed Electrophilic Amination of Organozinc Nucleophiles: Documentation of *O*-Benzoyl Hydroxylamines as Broadly Useful $R_2N(+)$ and RHN(+) Synthons. *J. Org. Chem.* **2006**, *71*, 219-224.
(2) Li, P.; Kou, G.; Feng, T.; Wang, M.; Qiu, Y. Electrochemical NiH-Catalyzed $C(sp^3)$-$C(sp^3)$ Coupling of Alkyl Halides and Alkyl Alkenes. *Angew. Chem., Int. Ed.* **2023**, *62*, e202311941;

(3) Zheng, S.; Wang, W.; Yuan, W. Remote and Proximal Hydroaminoalkylation of Alkenes Enabled by Photoredox/Nickel Dual Catalysis. *J. Am. Chem. Soc.* **2022**, *144*, 17776-17782.
(4) Wang, W.; Yan, X.; Ye, F.; Zheng, S.; Huang, G.; Yuan, W. Nickel/Photoredox Dual-Catalyzed Regiodivergent Aminoalkylation of Unactivated Alkyl Halides. *J. Am. Chem. Soc.* **2023**, *145*, 23385-23394.
(5) (a) Mastalir, M.; Pittenauer, E.; Allmaier, G.; Kirchner, K. Manganese-Catalyzed Aminomethylation of Aromatic Compounds with Methanol as a Sustainable C1 Building Block. *J. Am. Chem. Soc.* **2017**, *139*, 8812-8815. (b) Zhao, H.; Zhao, S.; Li, X.; Deng, Y.; Jiang, H.; Zhang, M. Cobalt-Catalyzed Selective Functionalization of Aniline Derivatives with Hexafluoroisopropanol. *Org. Lett.* **2019**, *21*, 218-222.

6. NMR spectra

methyl 2-(piperidin-1-ylmethyl)octanoate (3a)

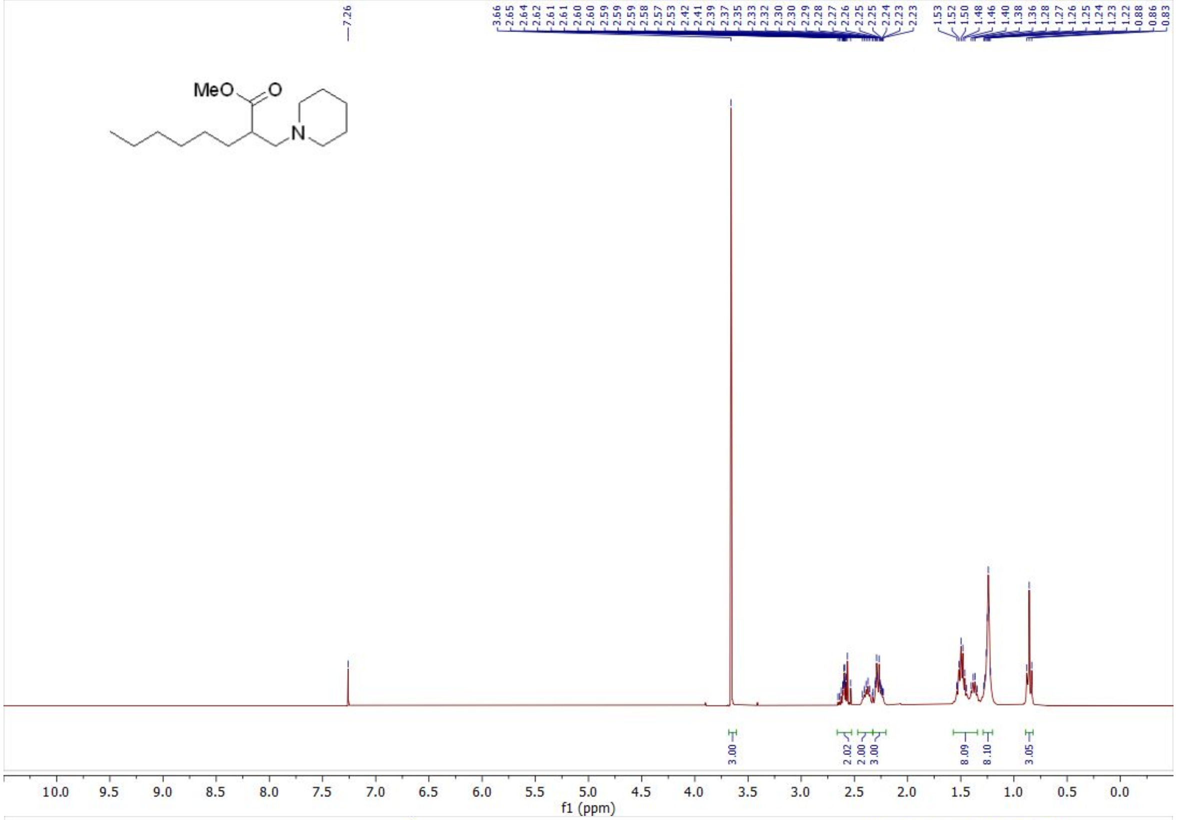

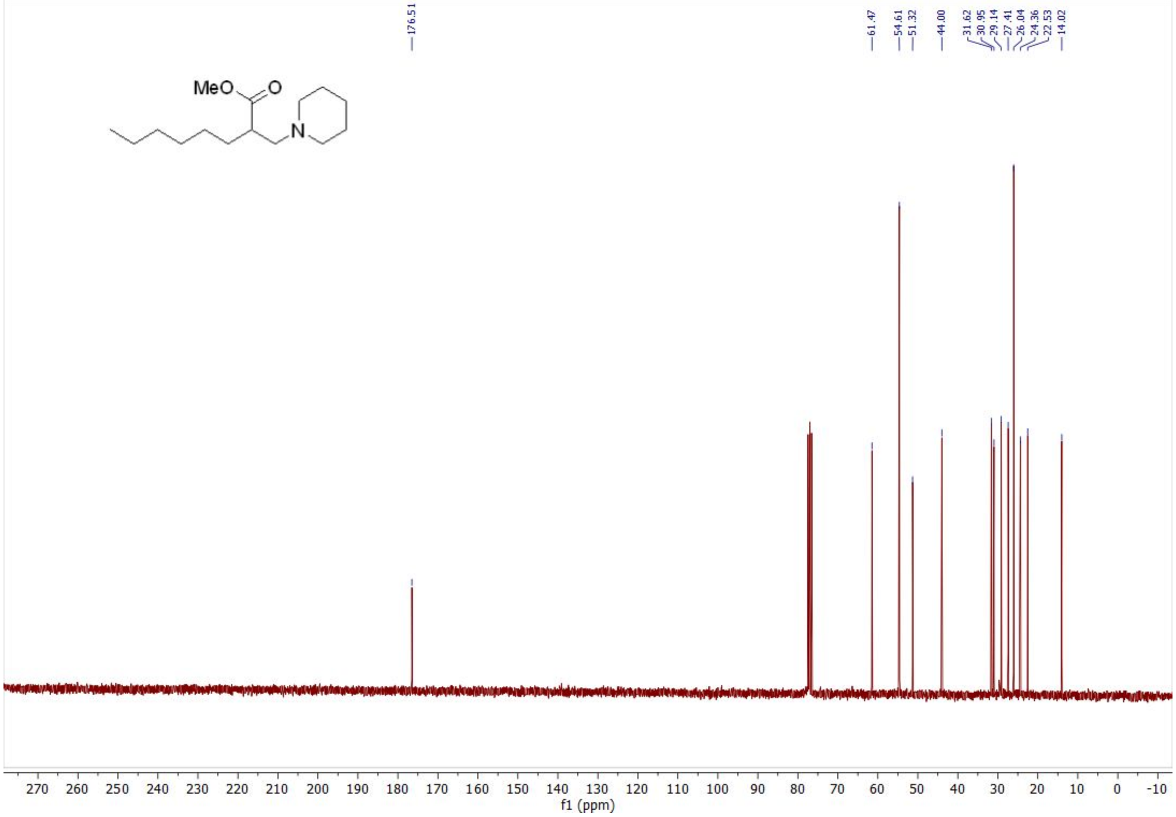

methyl 2-(piperidin-1-ylmethyl)decanoate (3b)

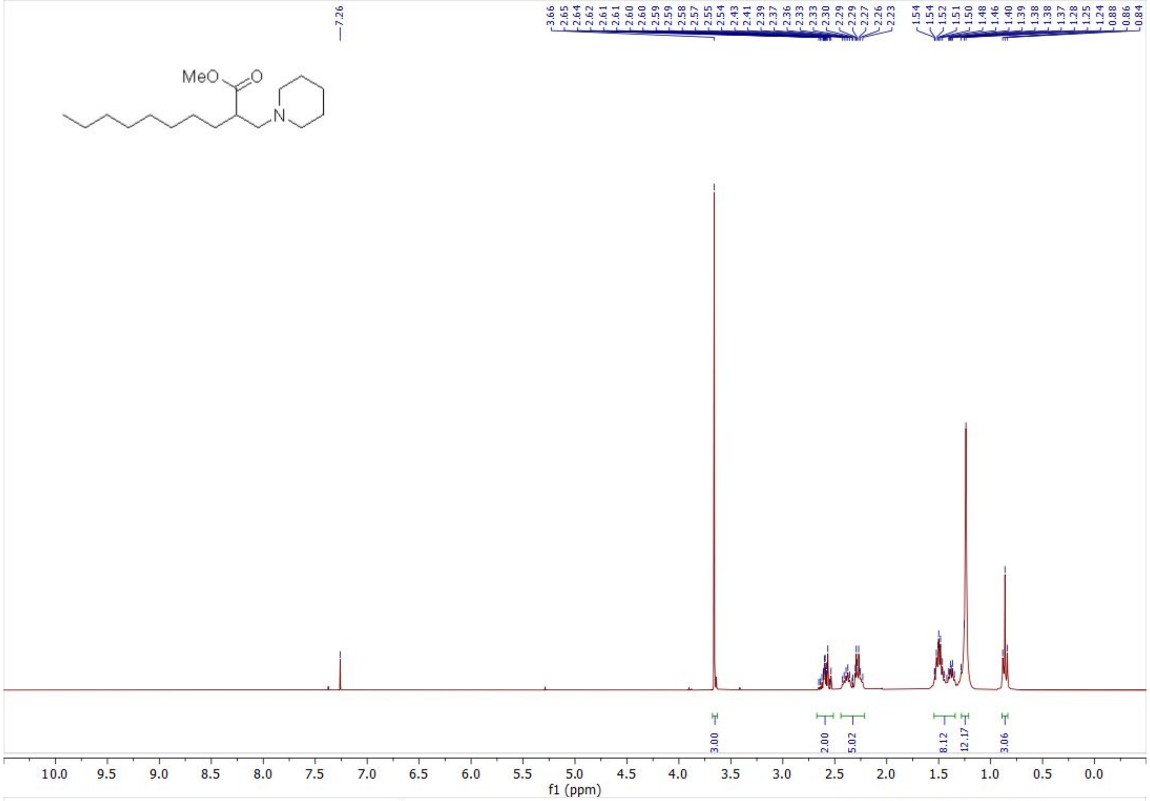

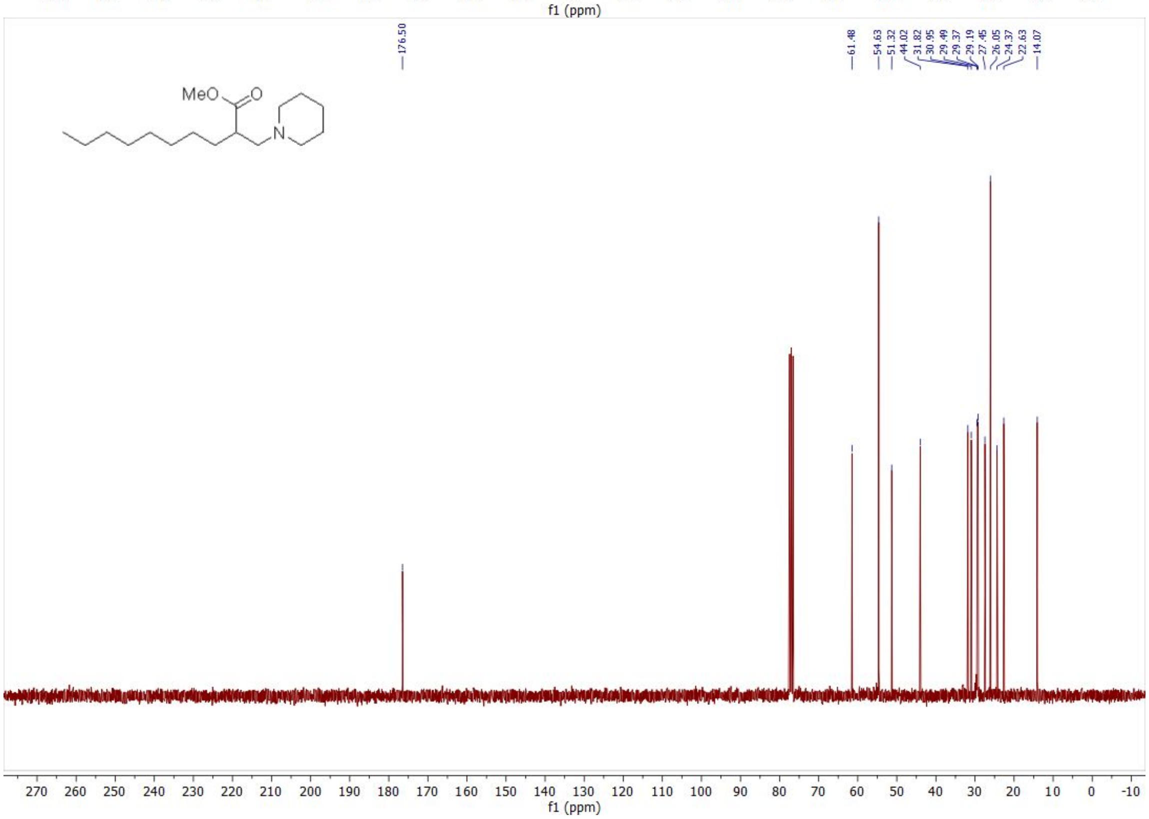

methyl 2-(piperidin-1-ylmethyl)dodecanoate (3c)

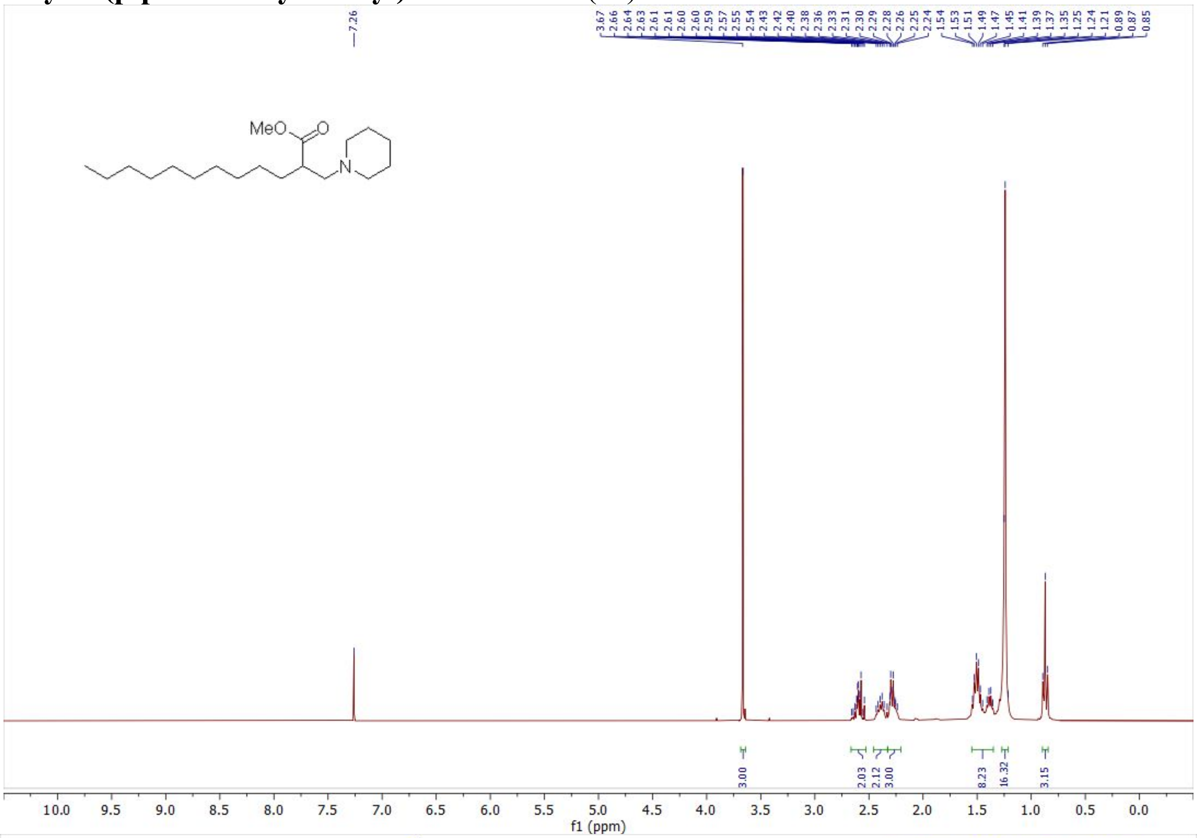

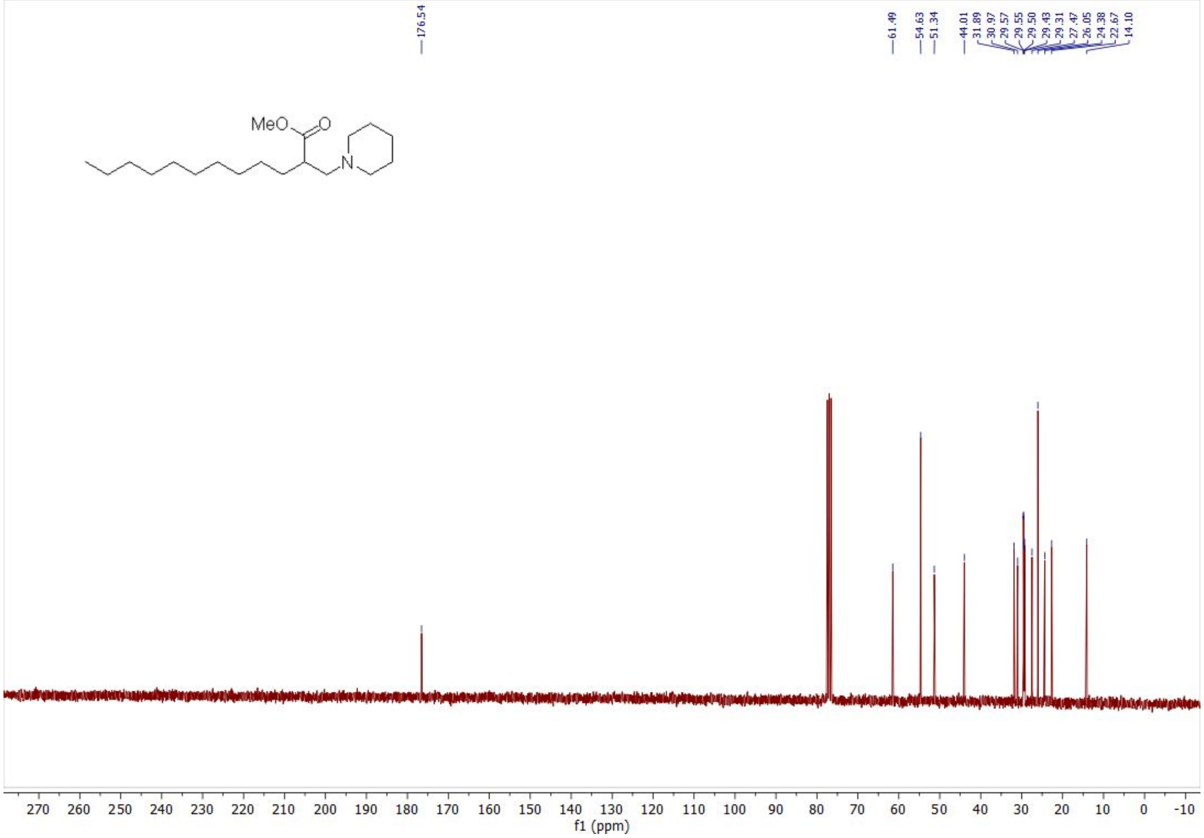

methyl 3-cyclohexyl-2-(piperidin-1-ylmethyl)propanoate (3d)

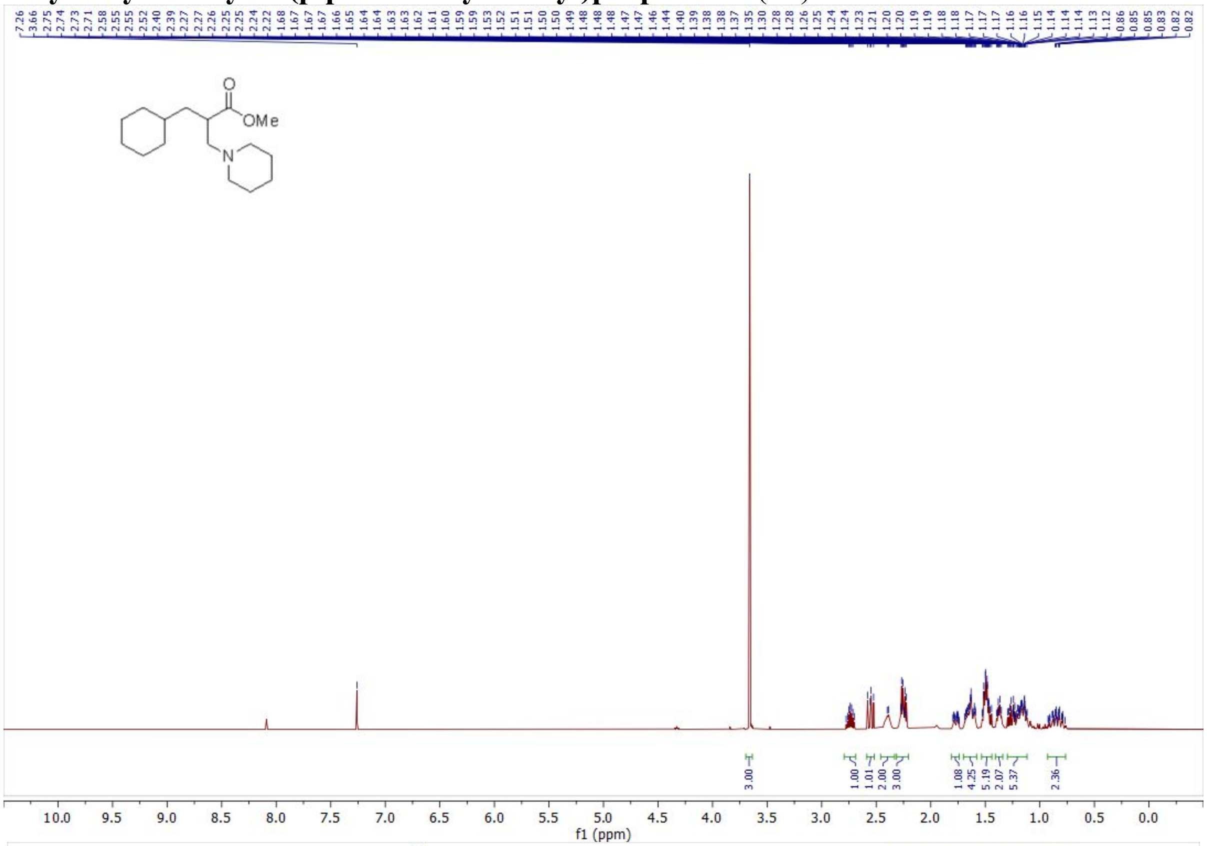

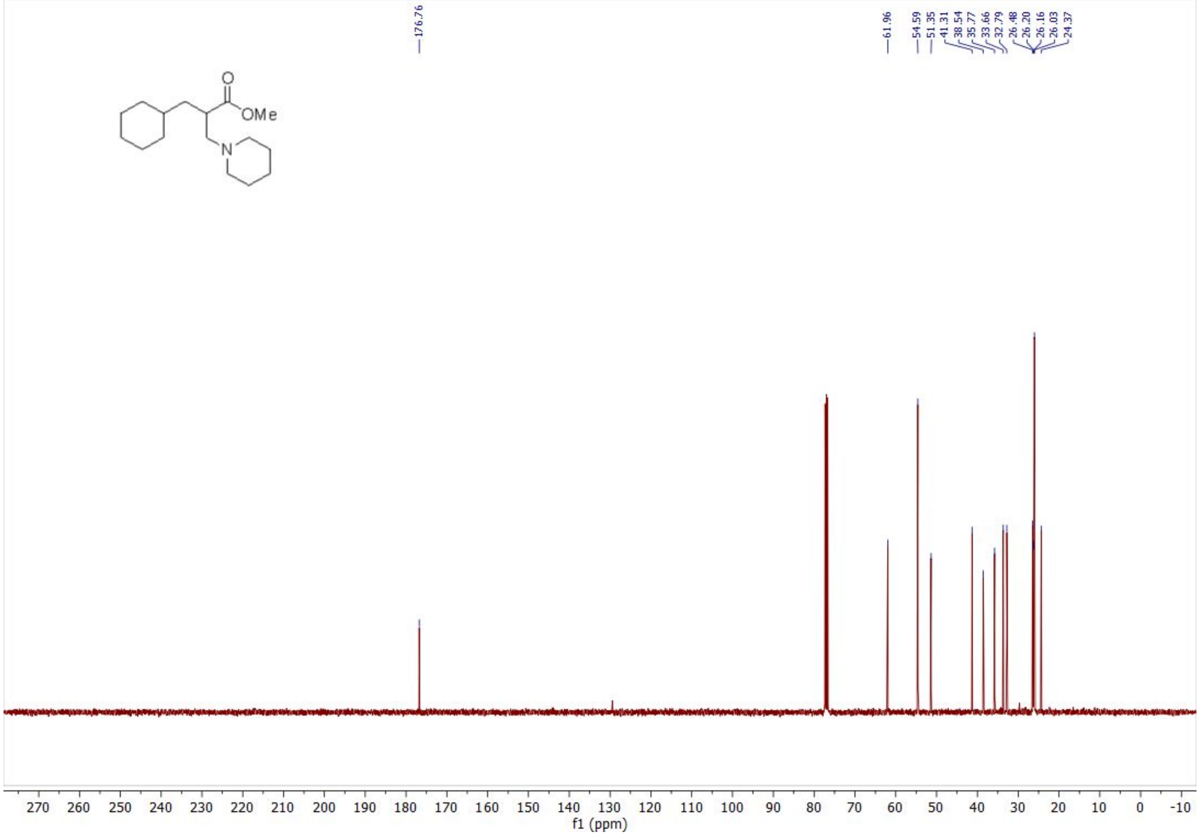

dimethyl 2-(piperidin-1-ylmethyl)undecanedioate (3e)

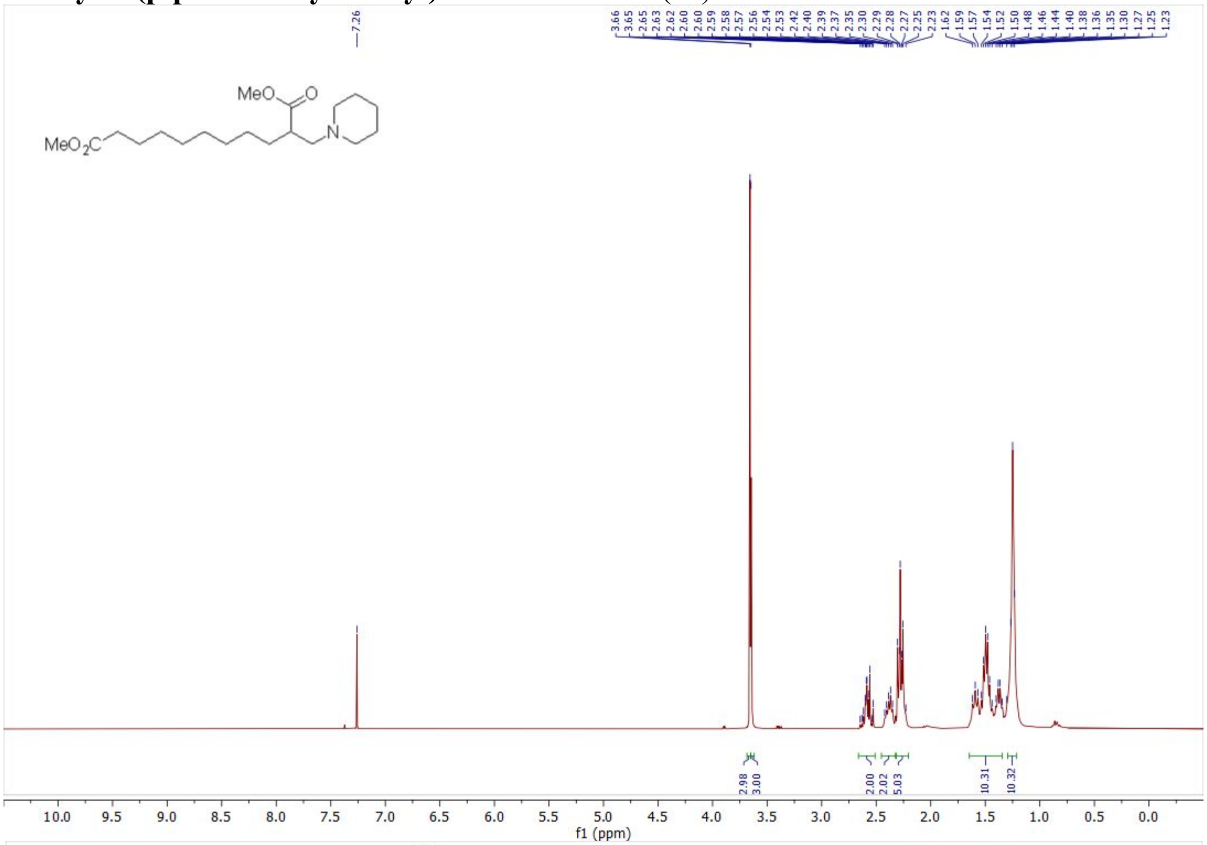

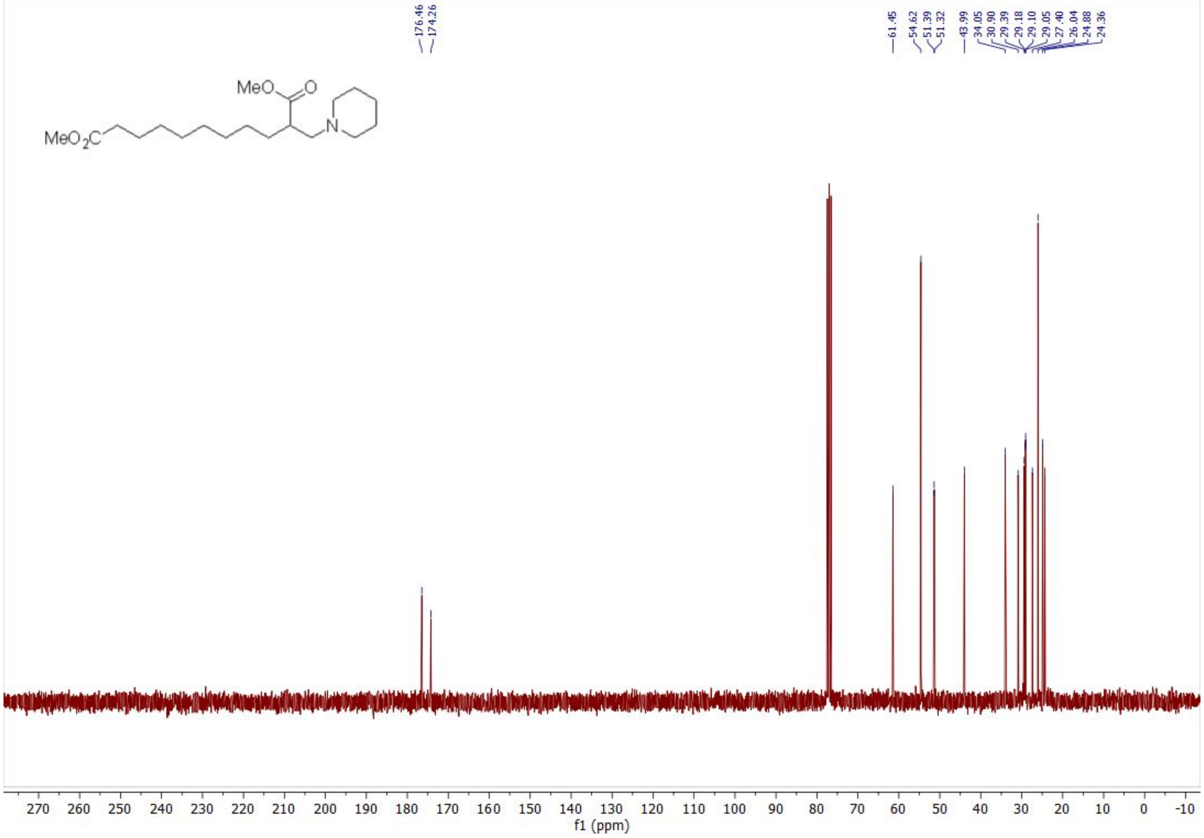

methyl 6-chloro-2-(piperidin-1-ylmethyl)hexanoate (3f)

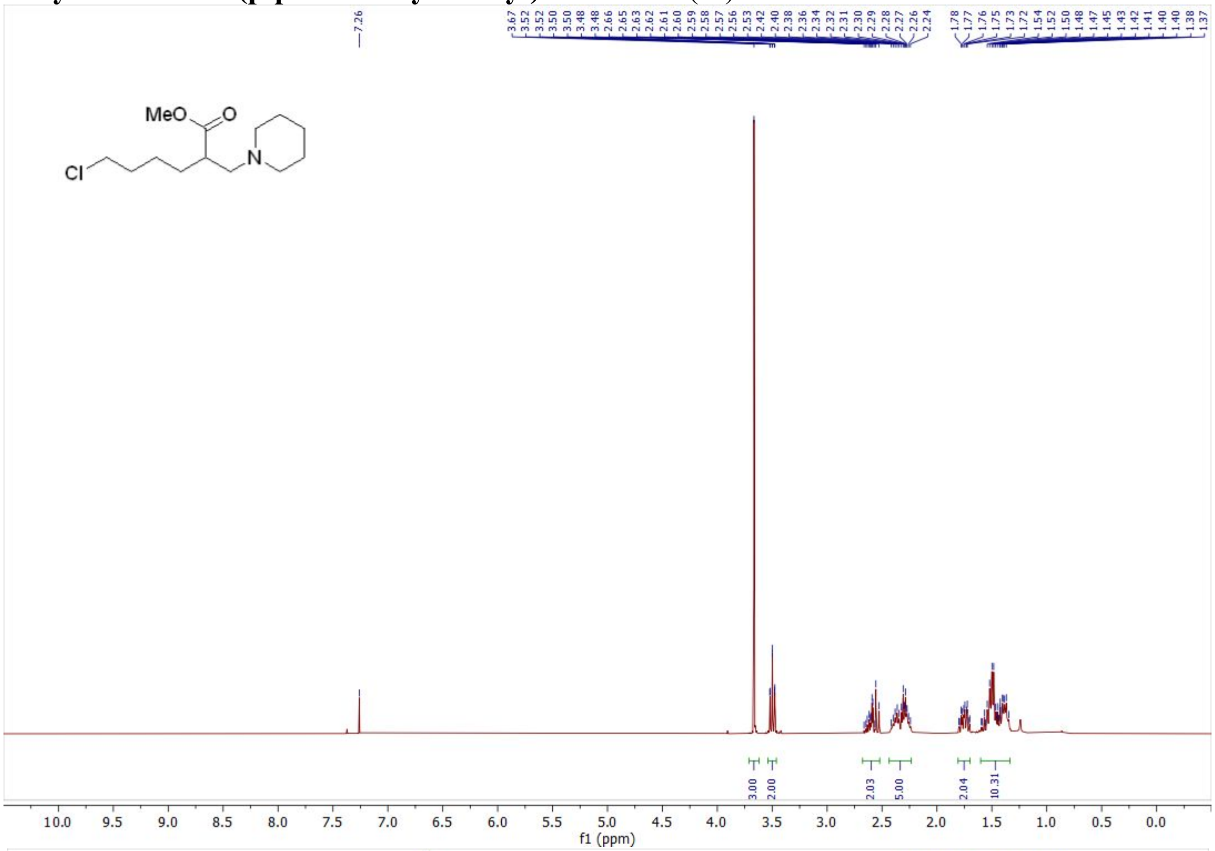

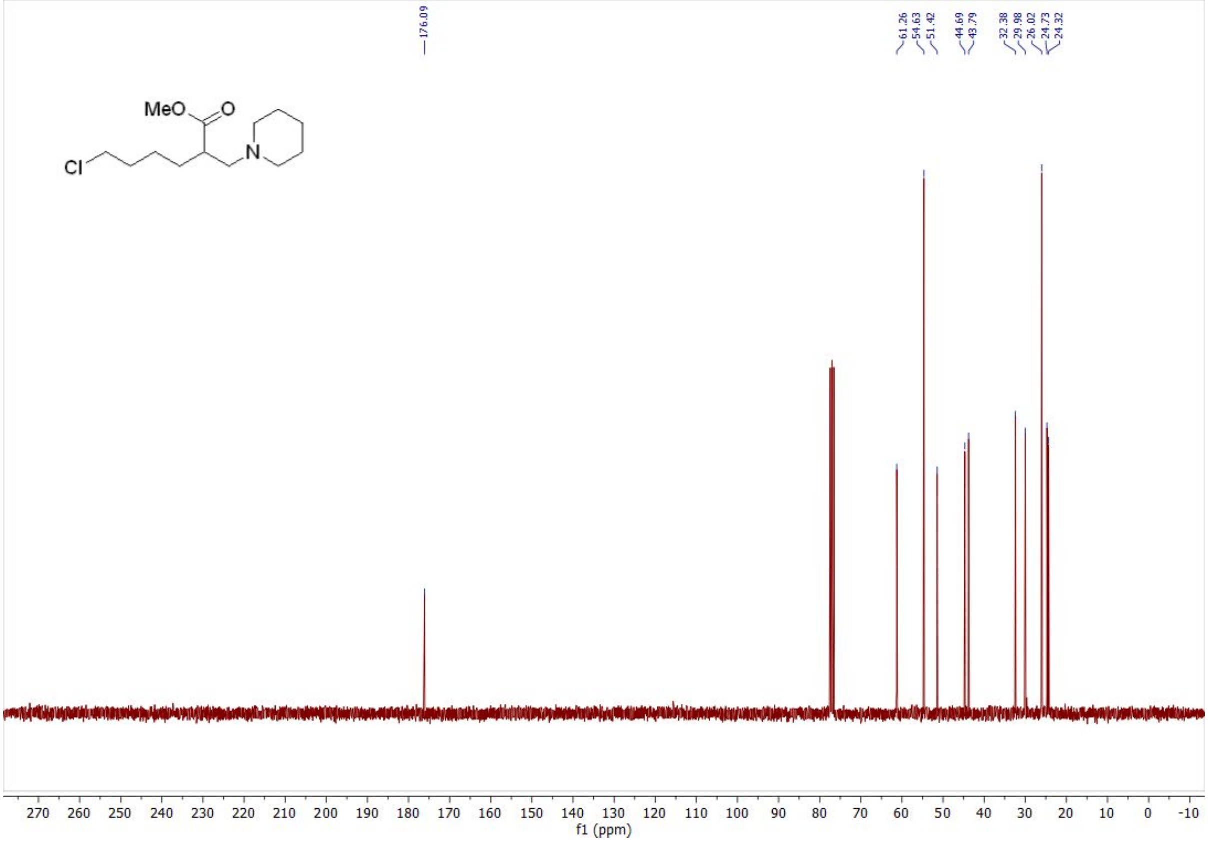

methyl 6-acetoxy-2-(piperidin-1-ylmethyl)hexanoate (3g)

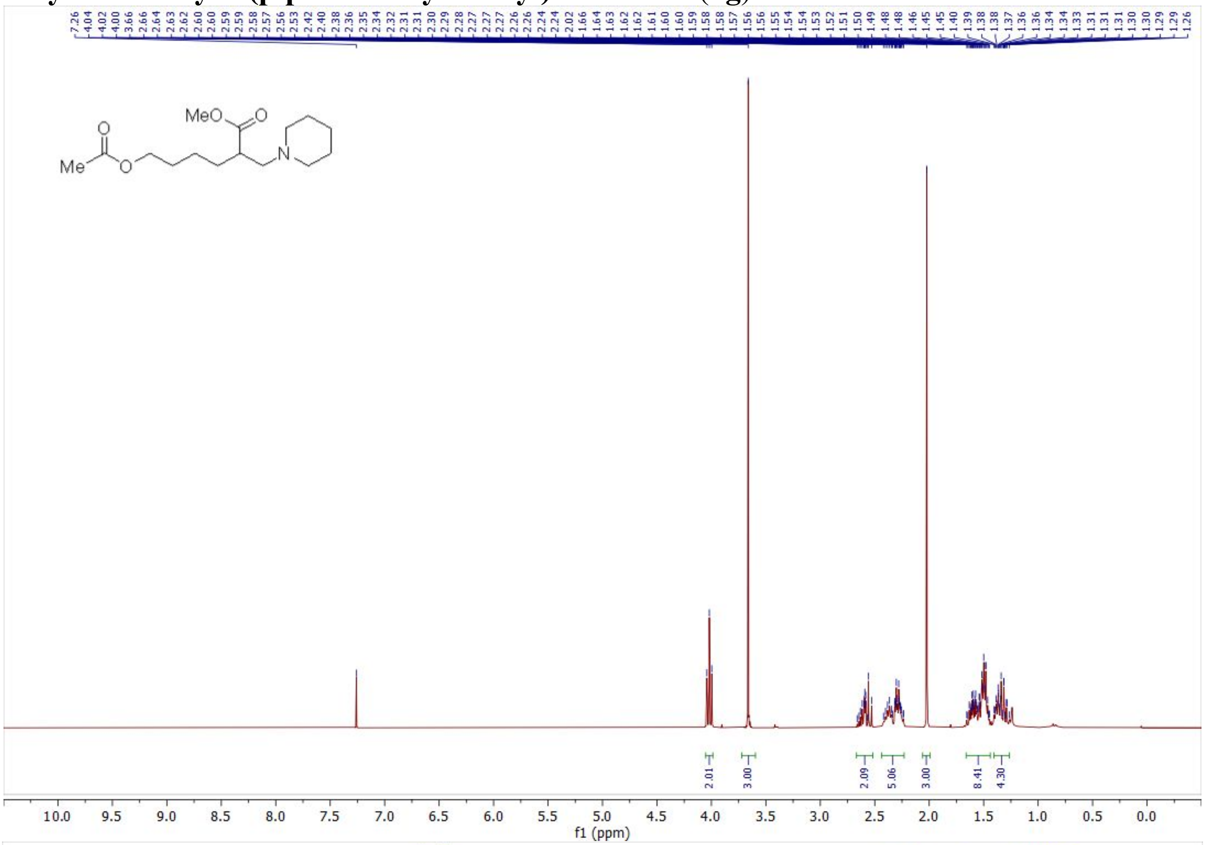

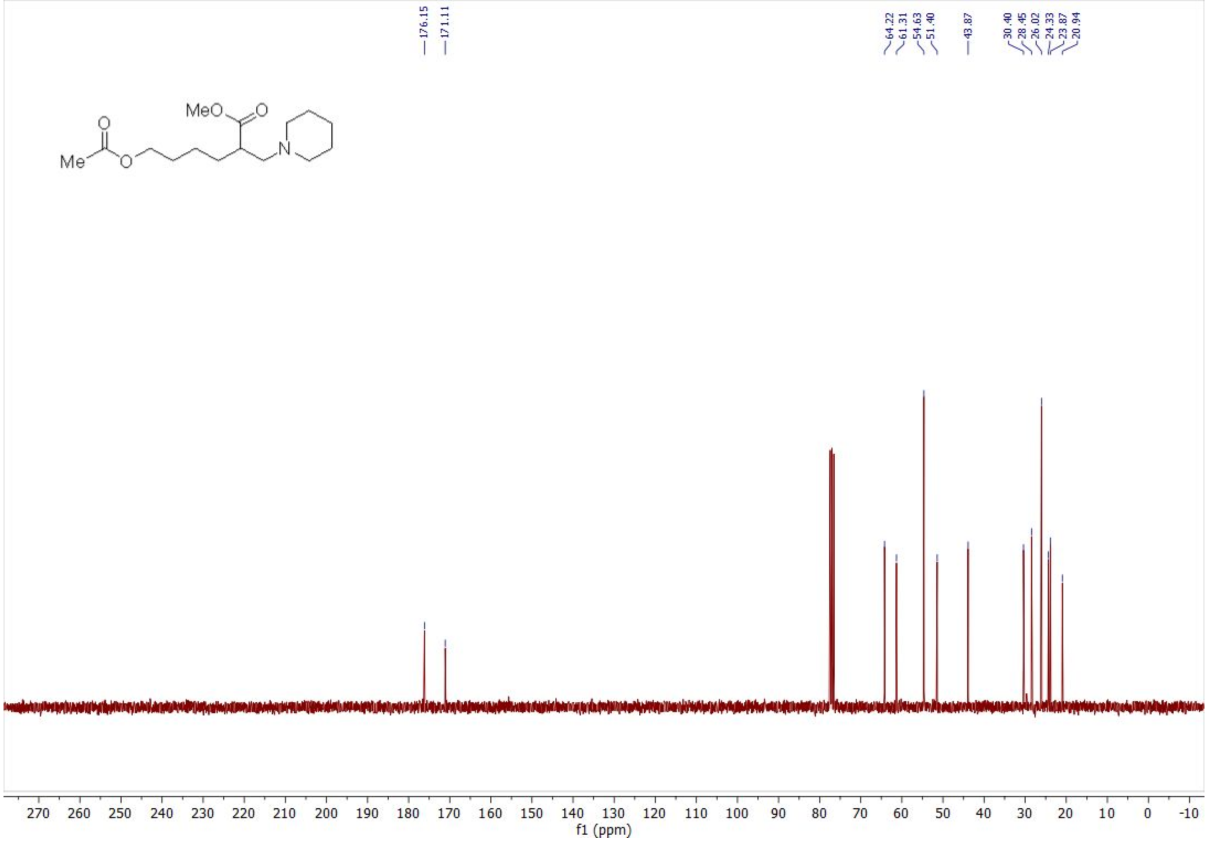

methyl 2-(piperidin-1-ylmethyl)decanoat methyl 2-(piperidin-1-ylmethyl)-6-(tosyloxy)hexanoate (3h)

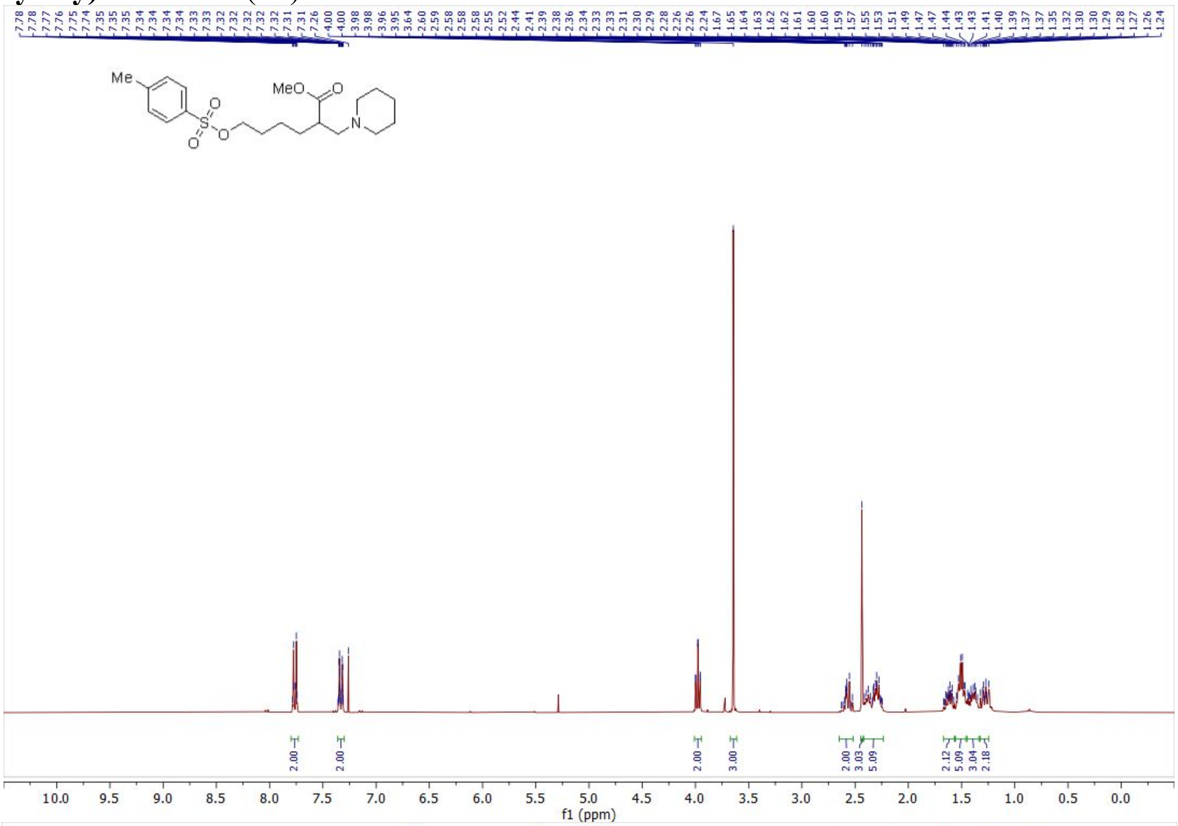

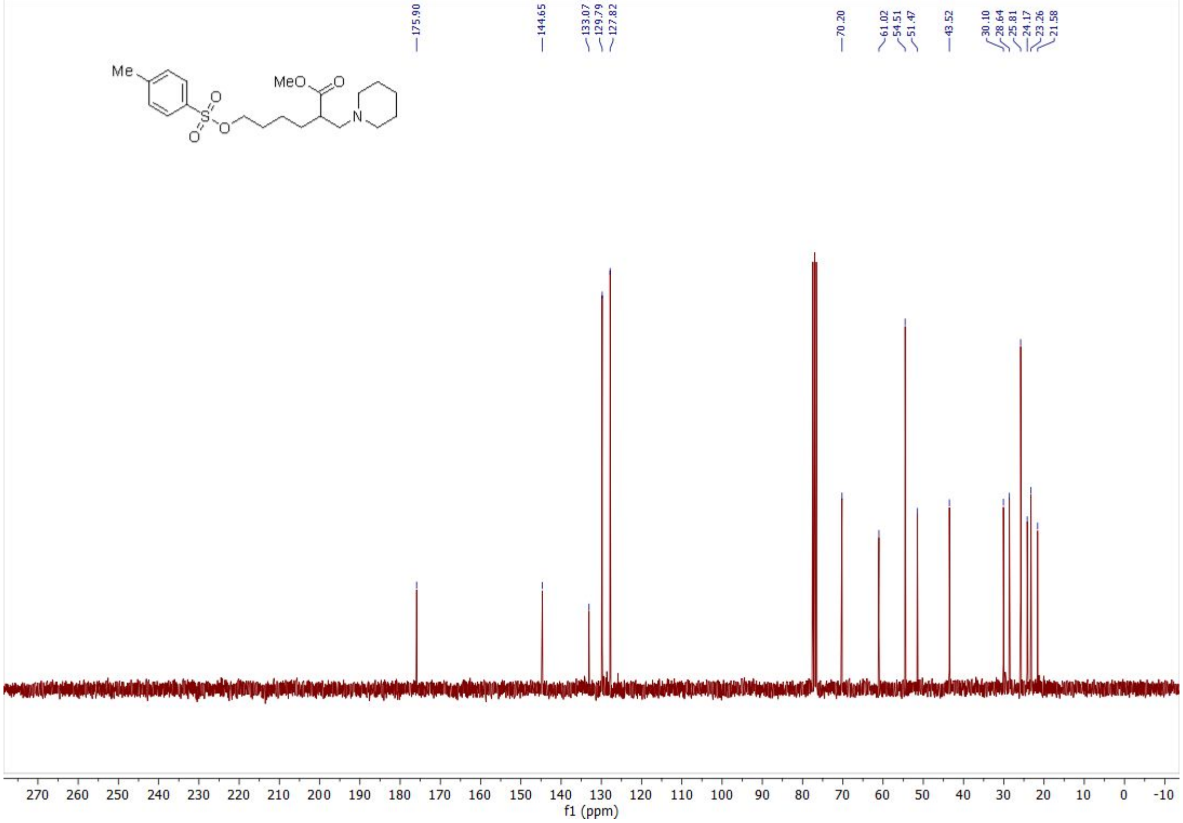

methyl 2-cyclohexyl-3-(piperidin-1-yl)propanoate (3i)

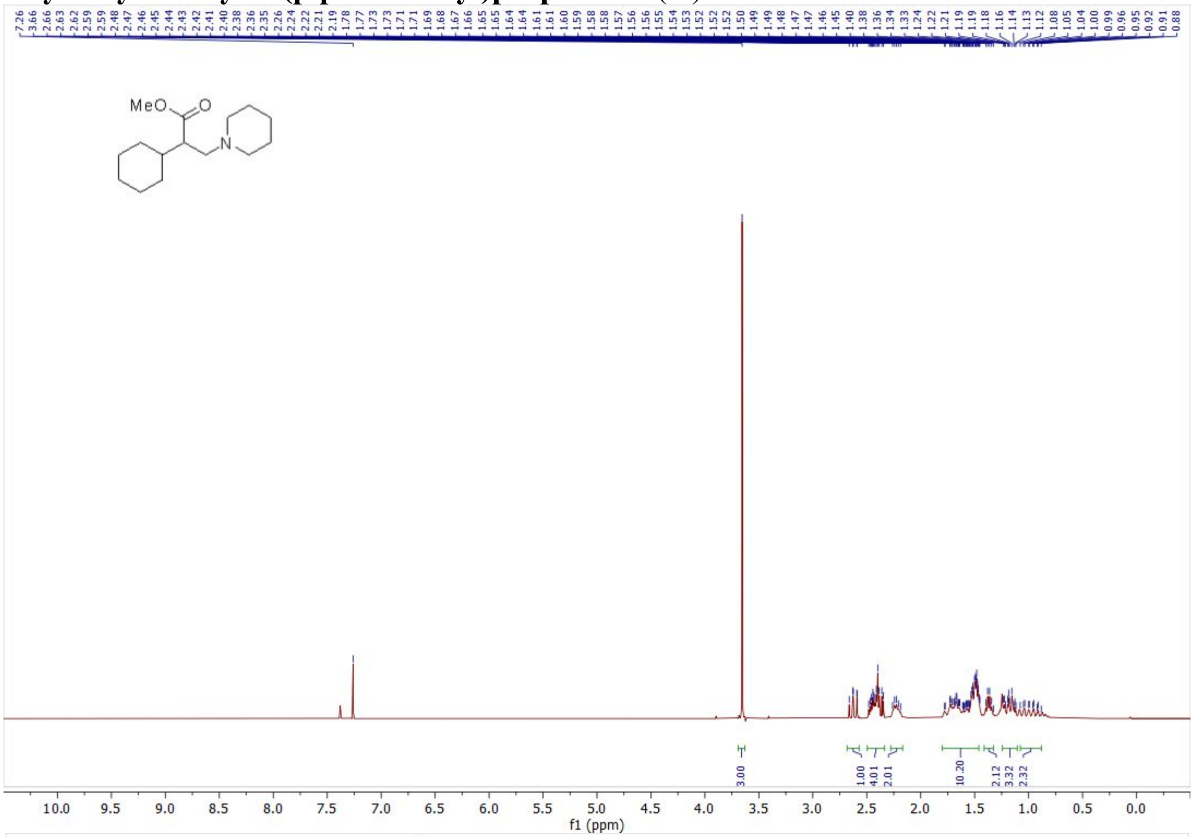

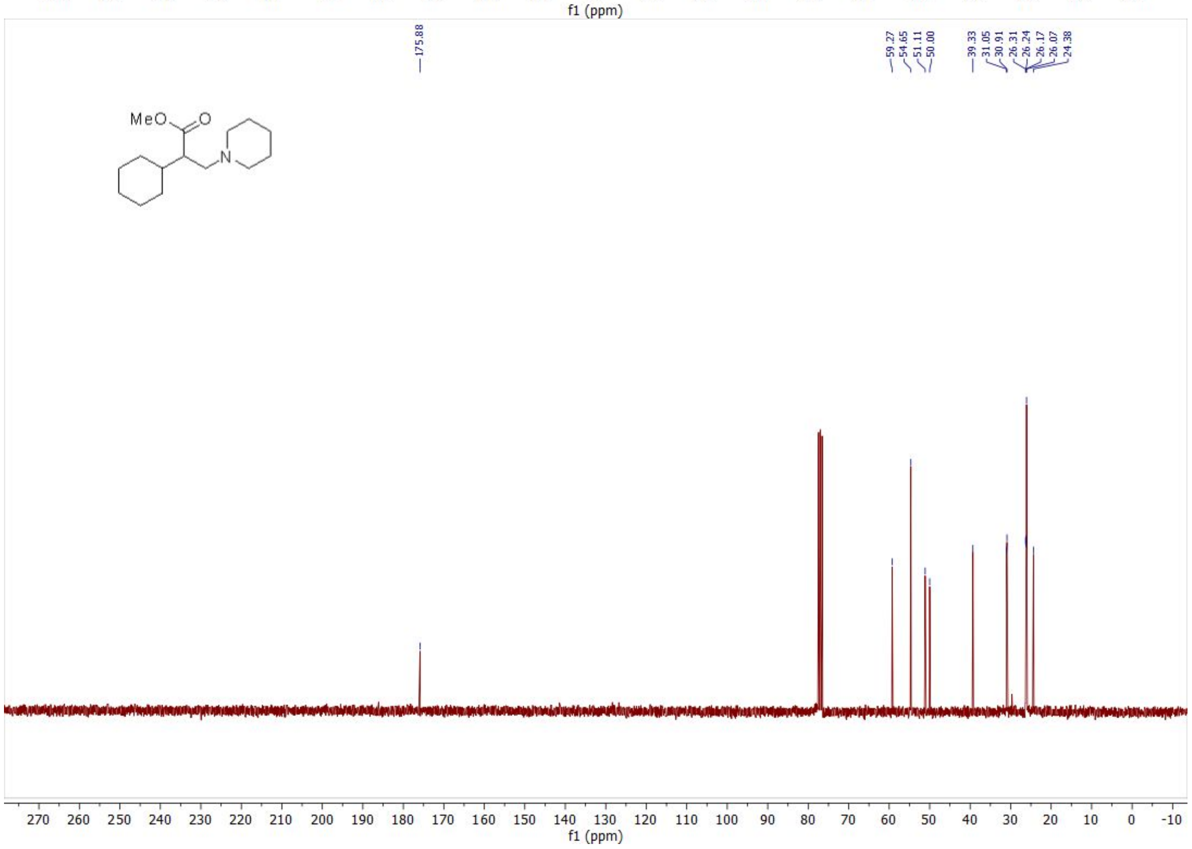

methyl 4-phenyl-2-(piperidin-1-ylmethyl)butanoate (3j)

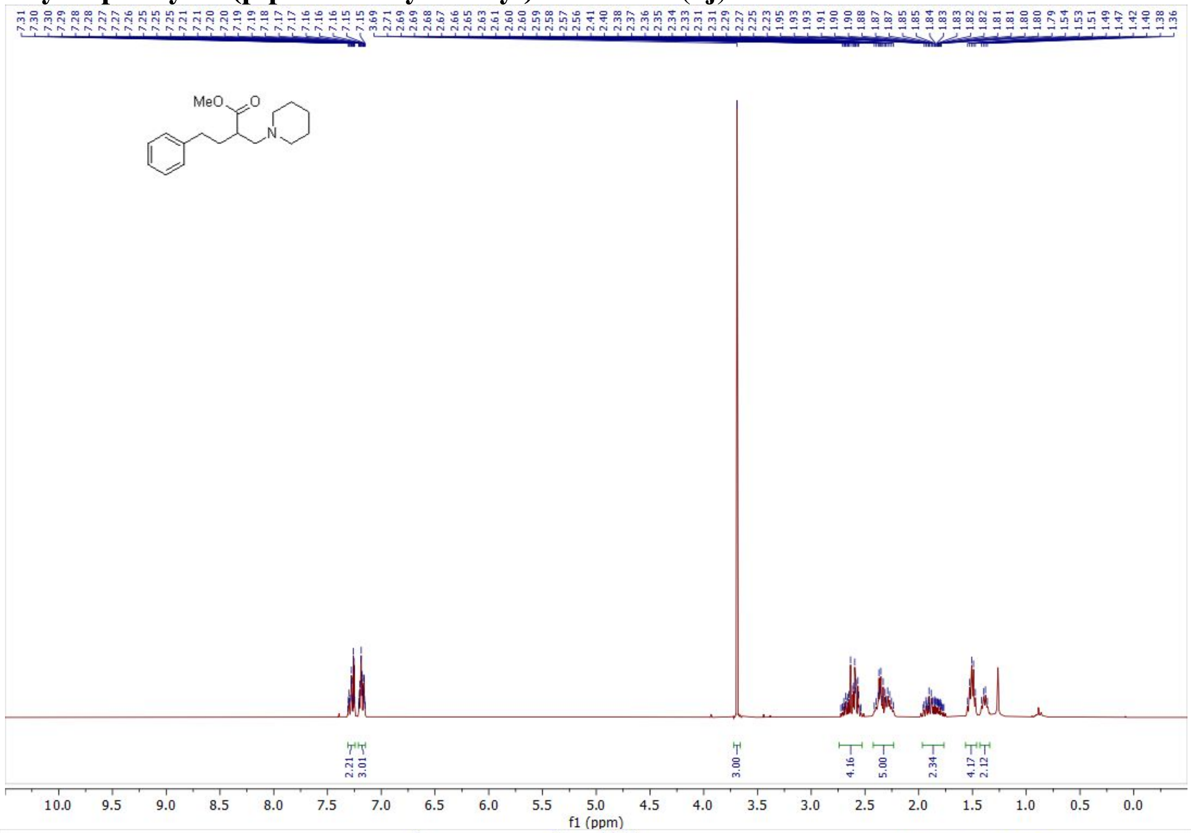

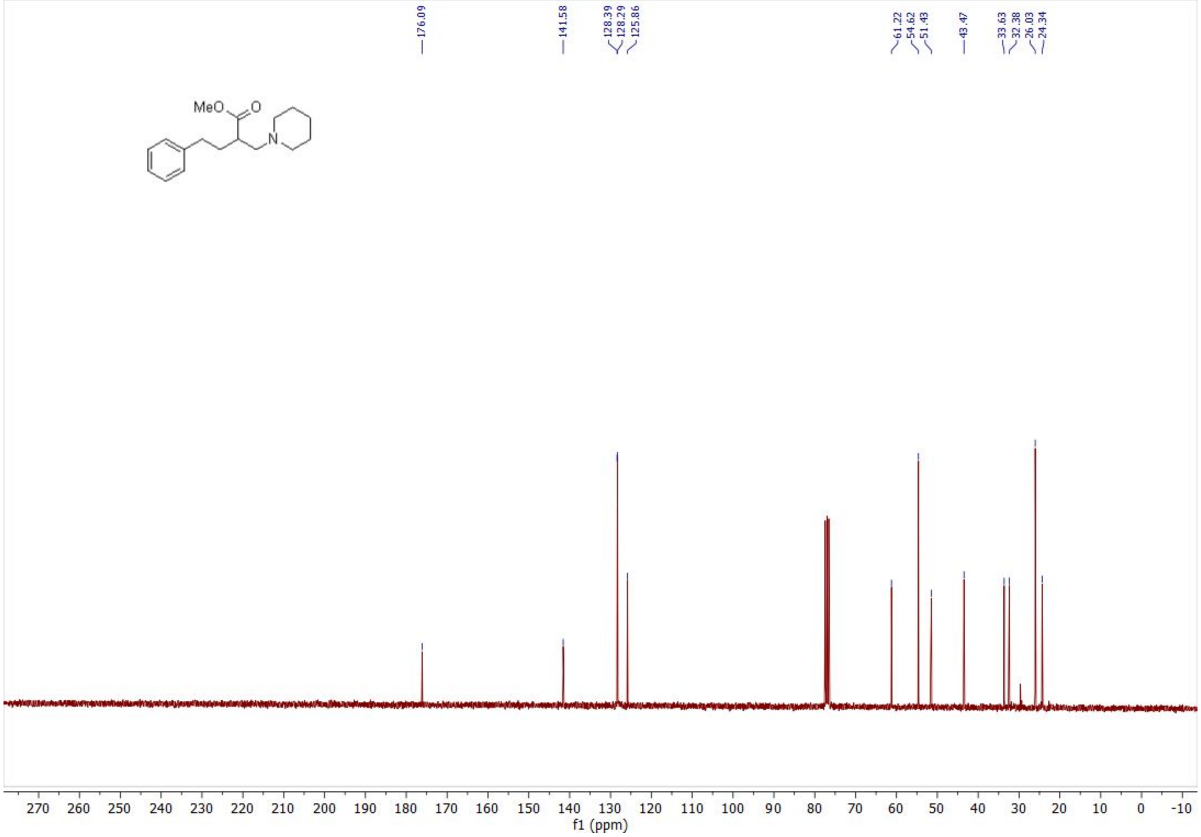

methyl 4-(4-(*tert*-butyl)phenyl)-2-(piperidin-1-ylmethyl)butanoate (3k)

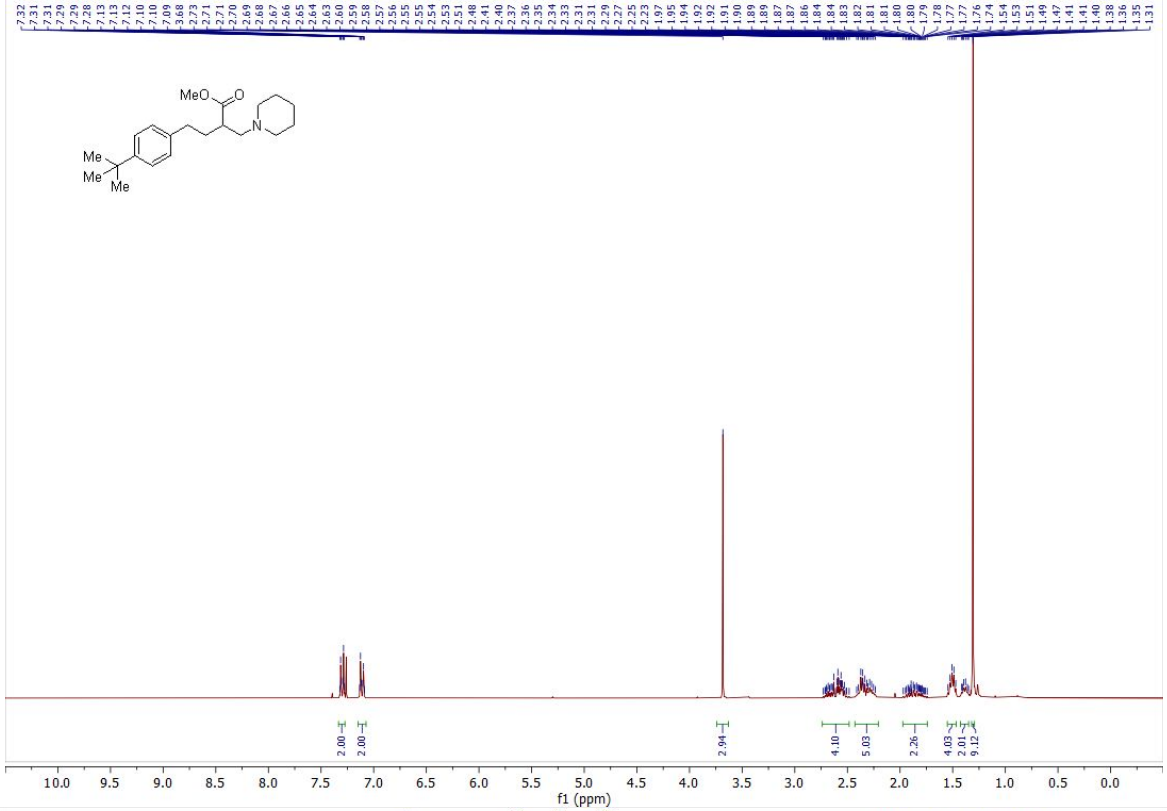

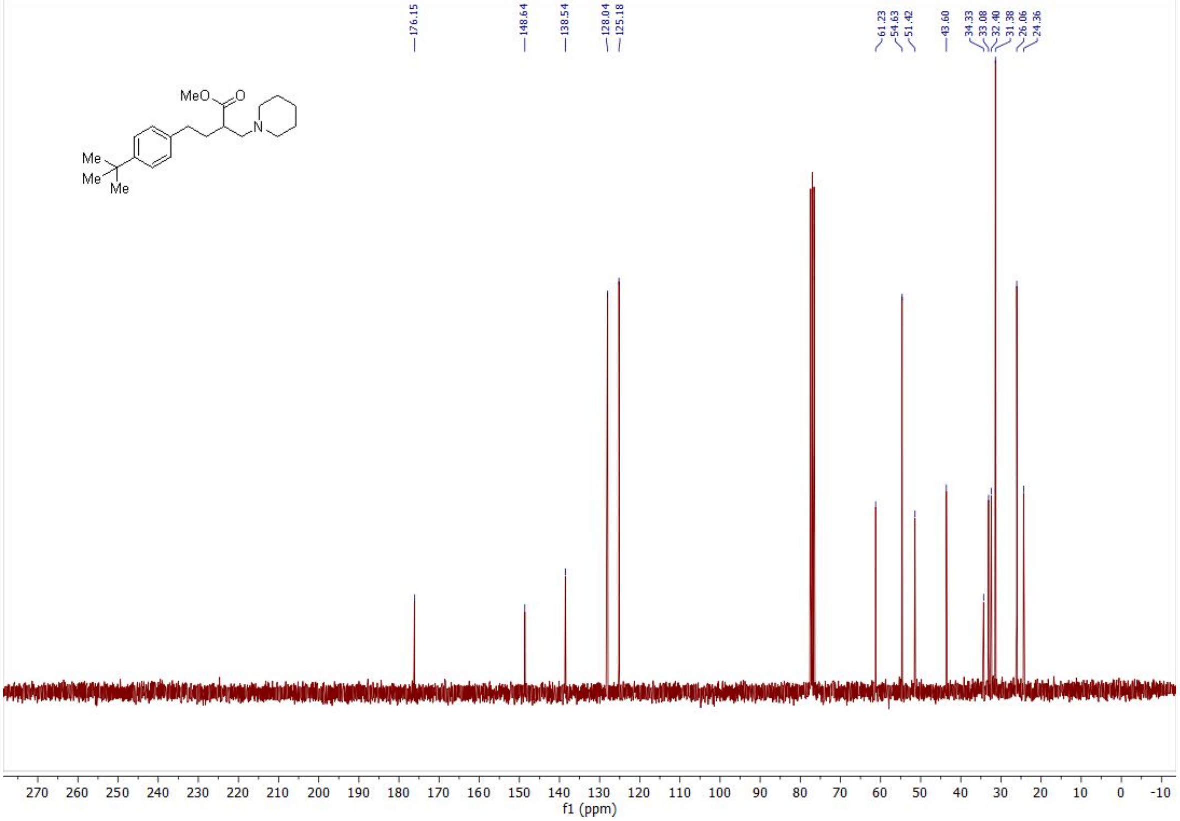

methyl 4-([1,1'-biphenyl]-4-yl)-2-(piperidin-1-ylmethyl)butanoate (3l)

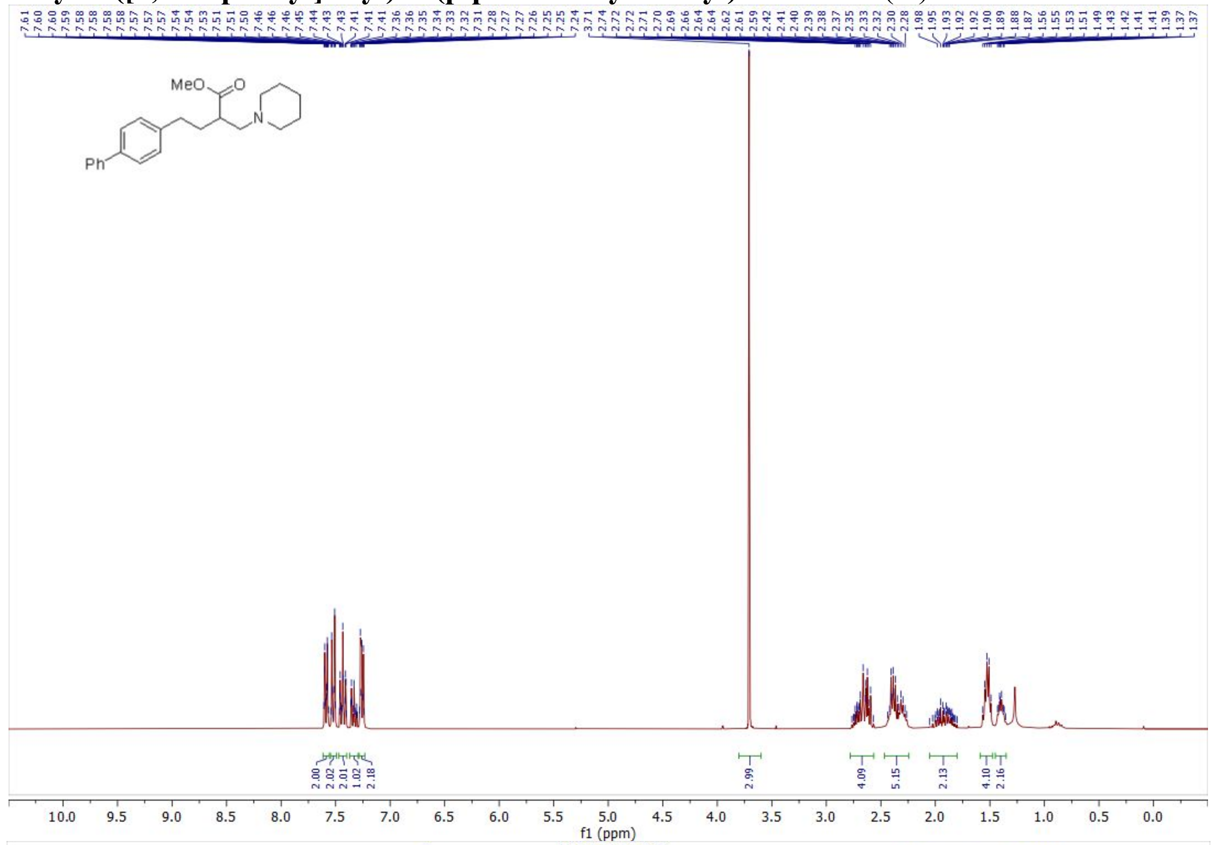

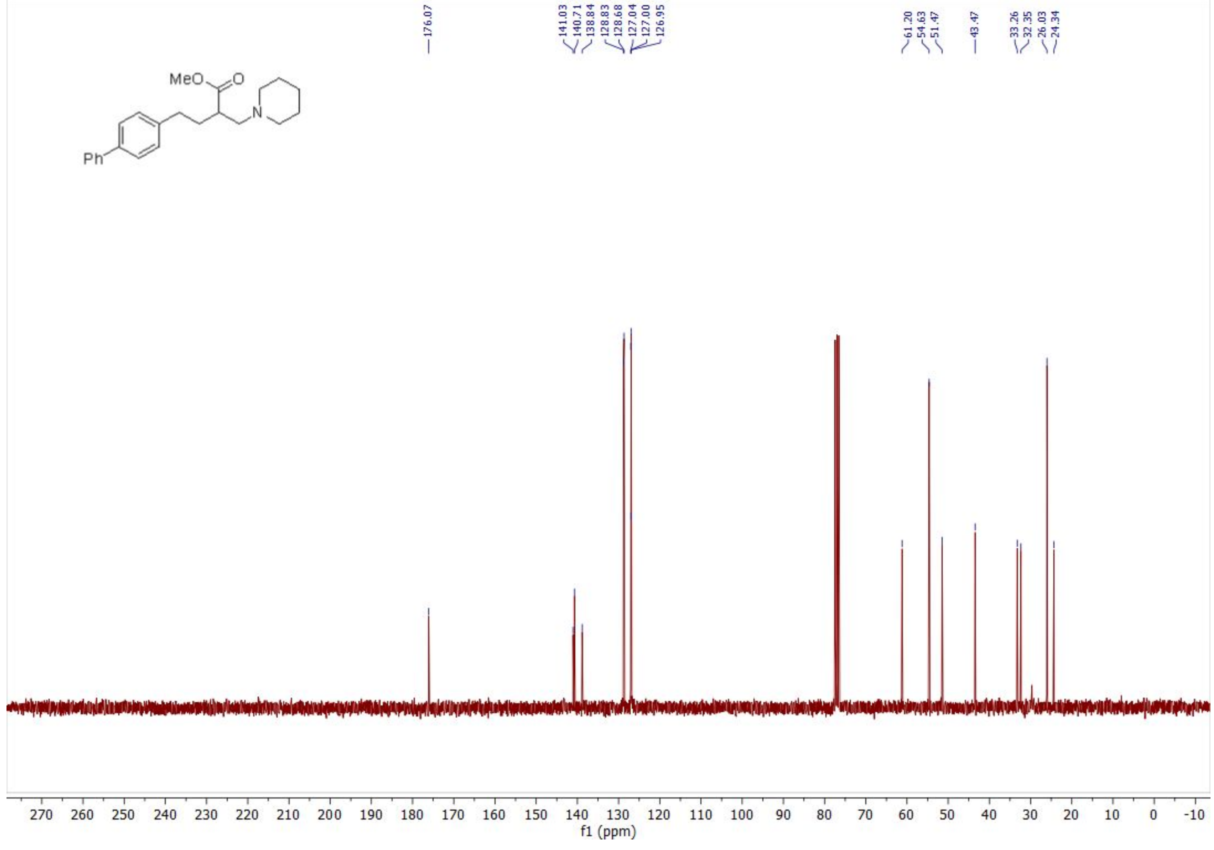

methyl 2-benzyl-3-(piperidin-1-yl)propanoate (3m)

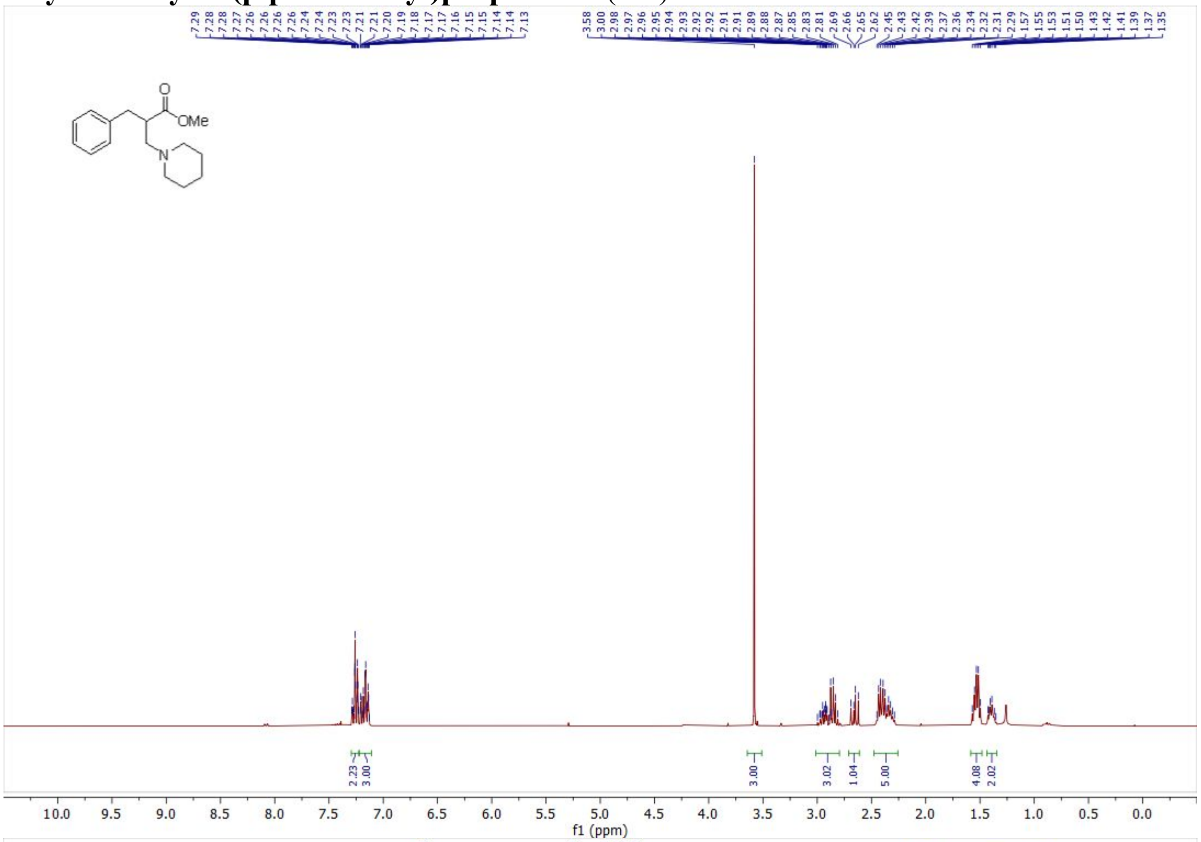

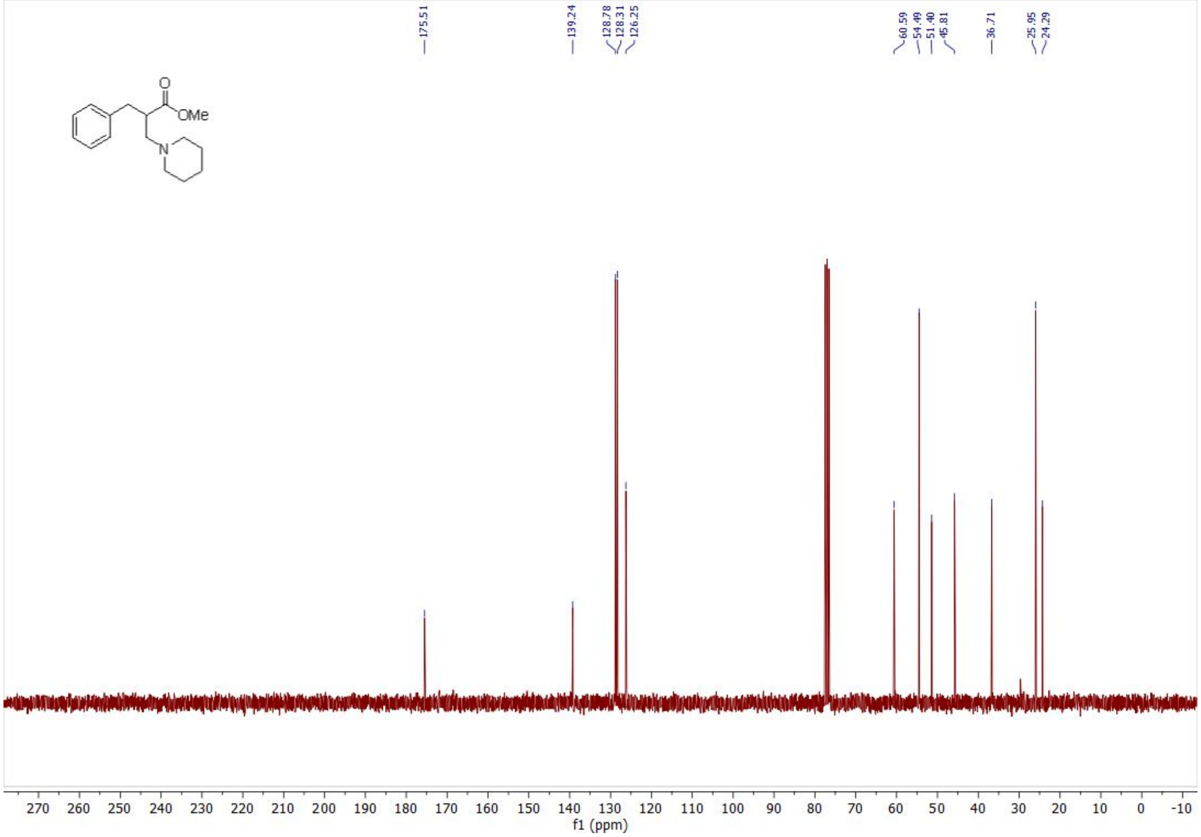

methyl 5-oxo-2-(piperidin-1-ylmethyl)hexanoate (3n)

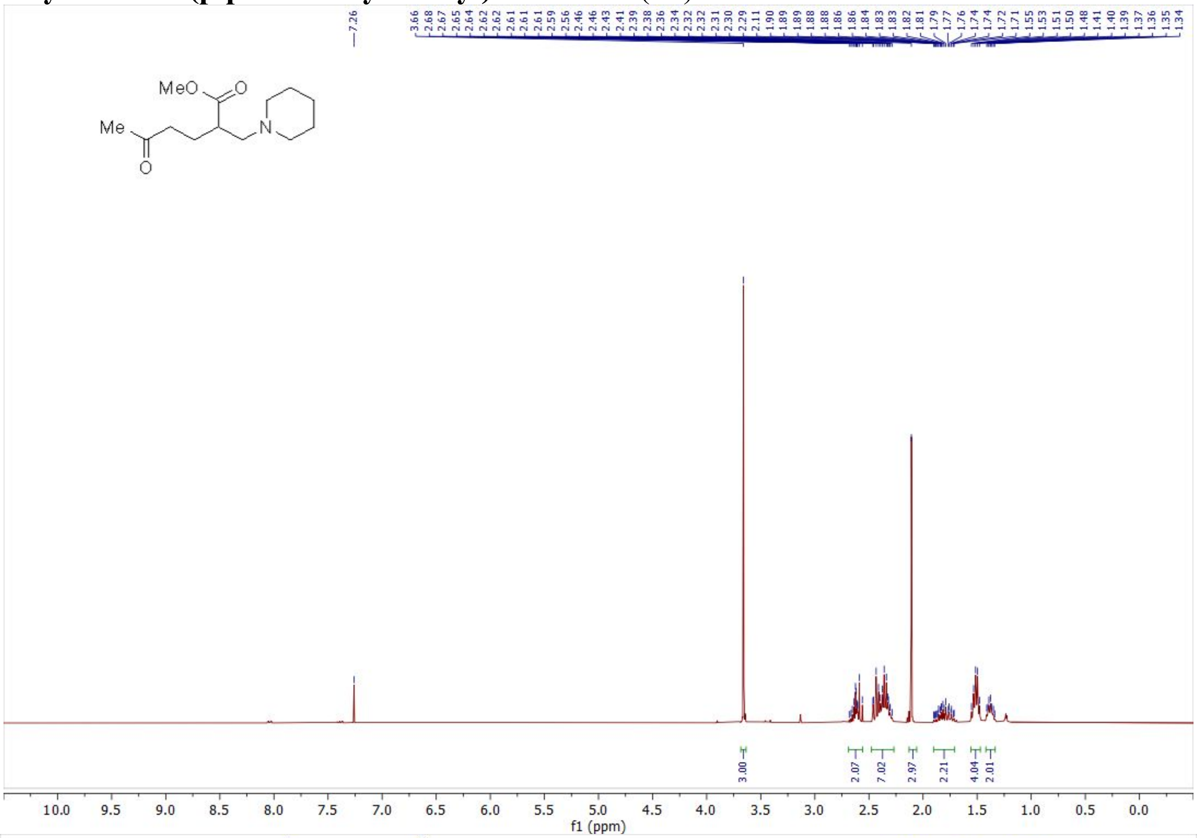

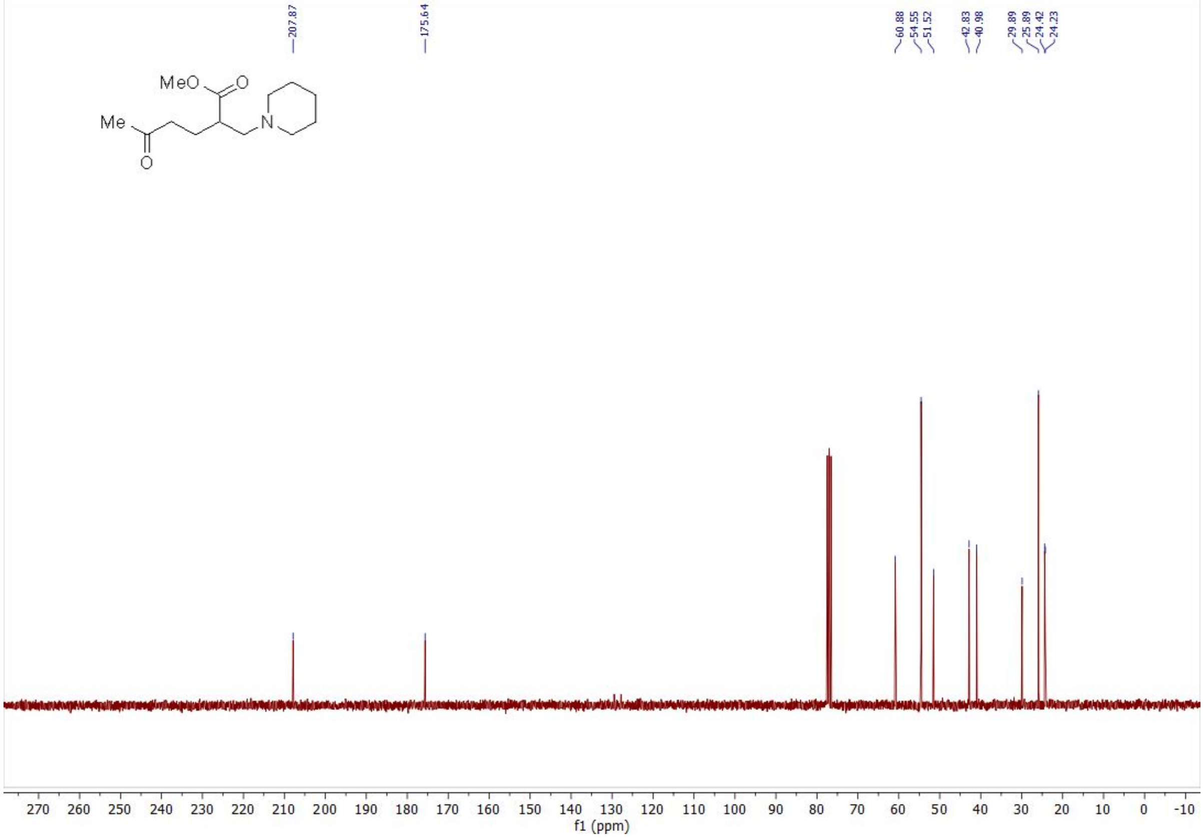

methyl 5-phenoxy-2-(piperidin-1-ylmethyl)pentanoate (3o)

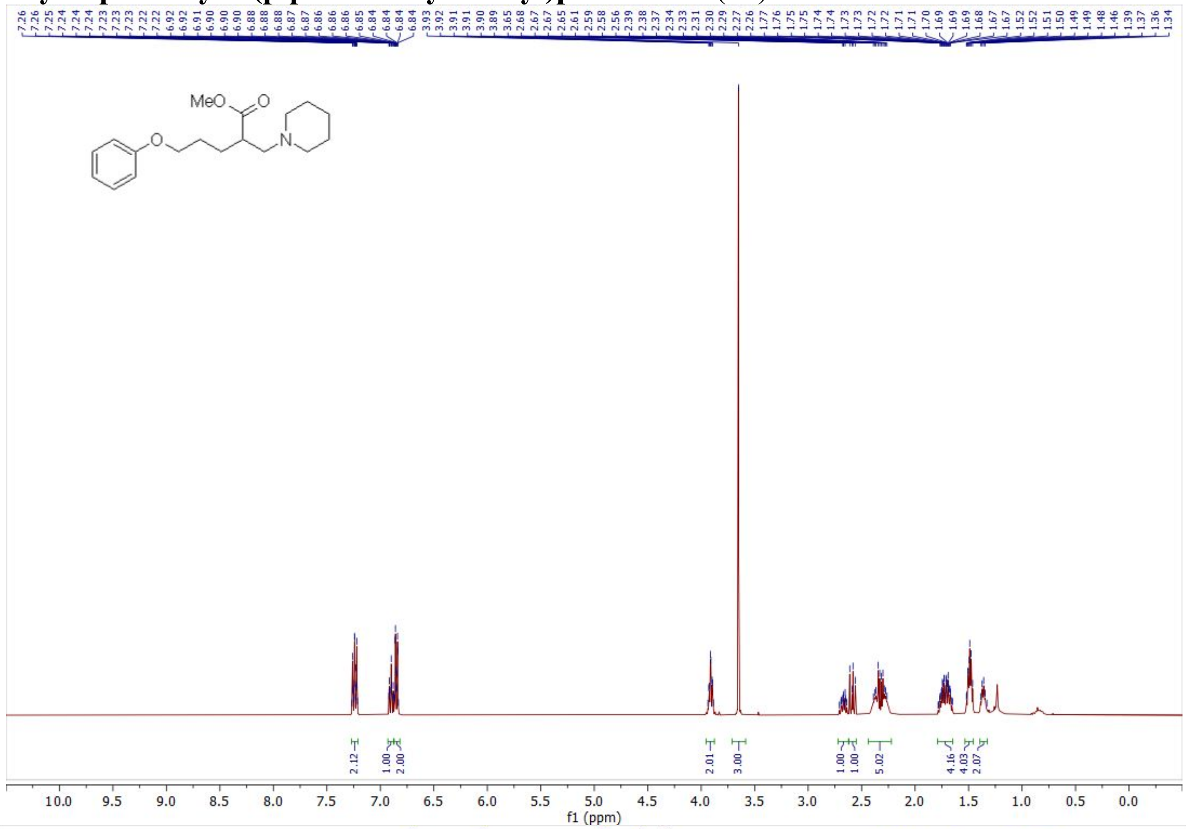

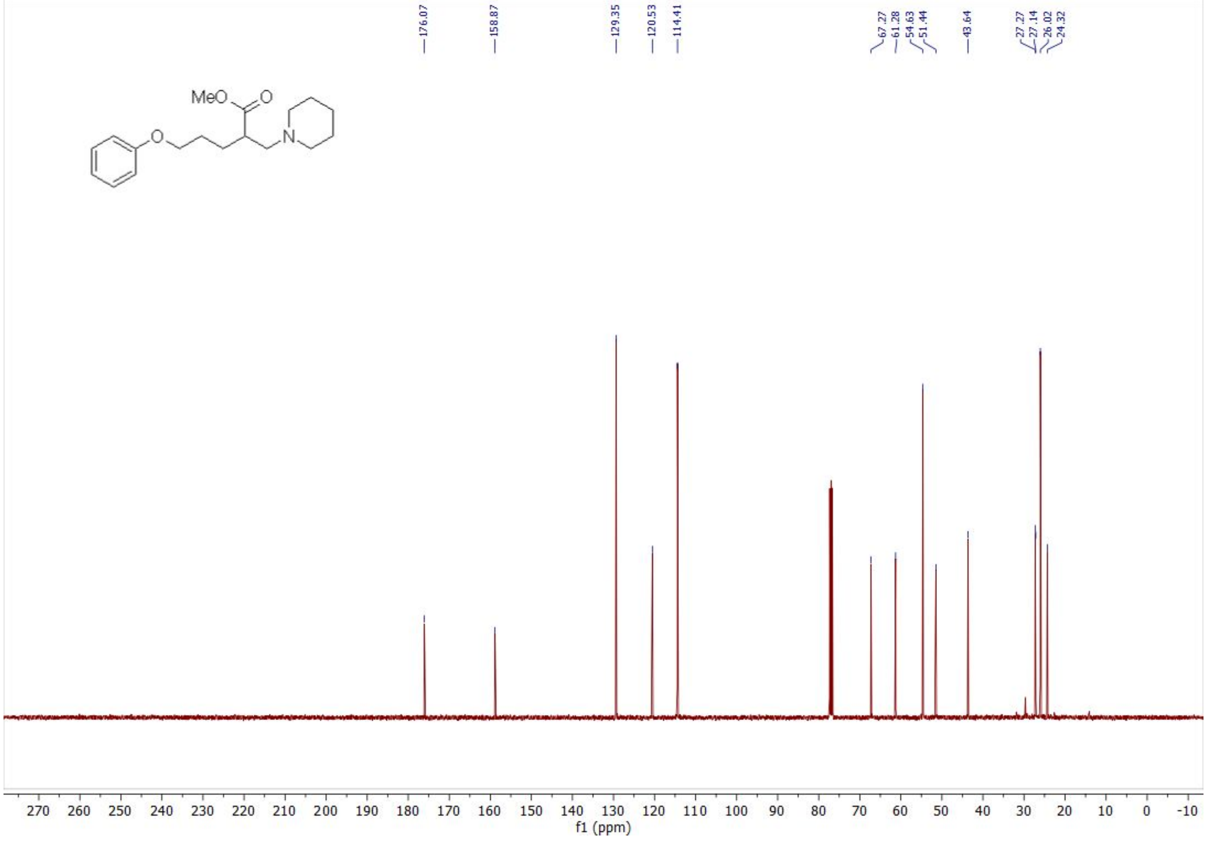

1,1-diethyl 3-methyl 4-(piperidin-1-yl)butane-1,1,3-tricarboxylate (3p)

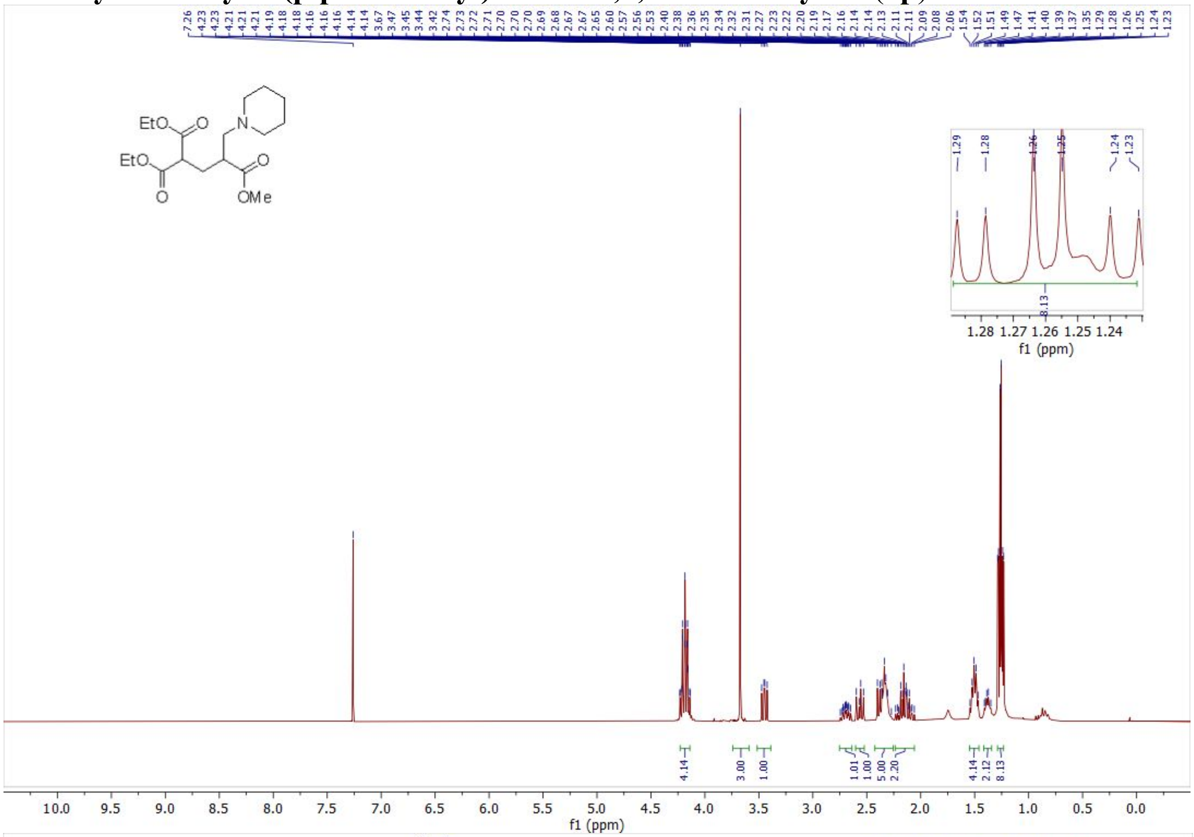

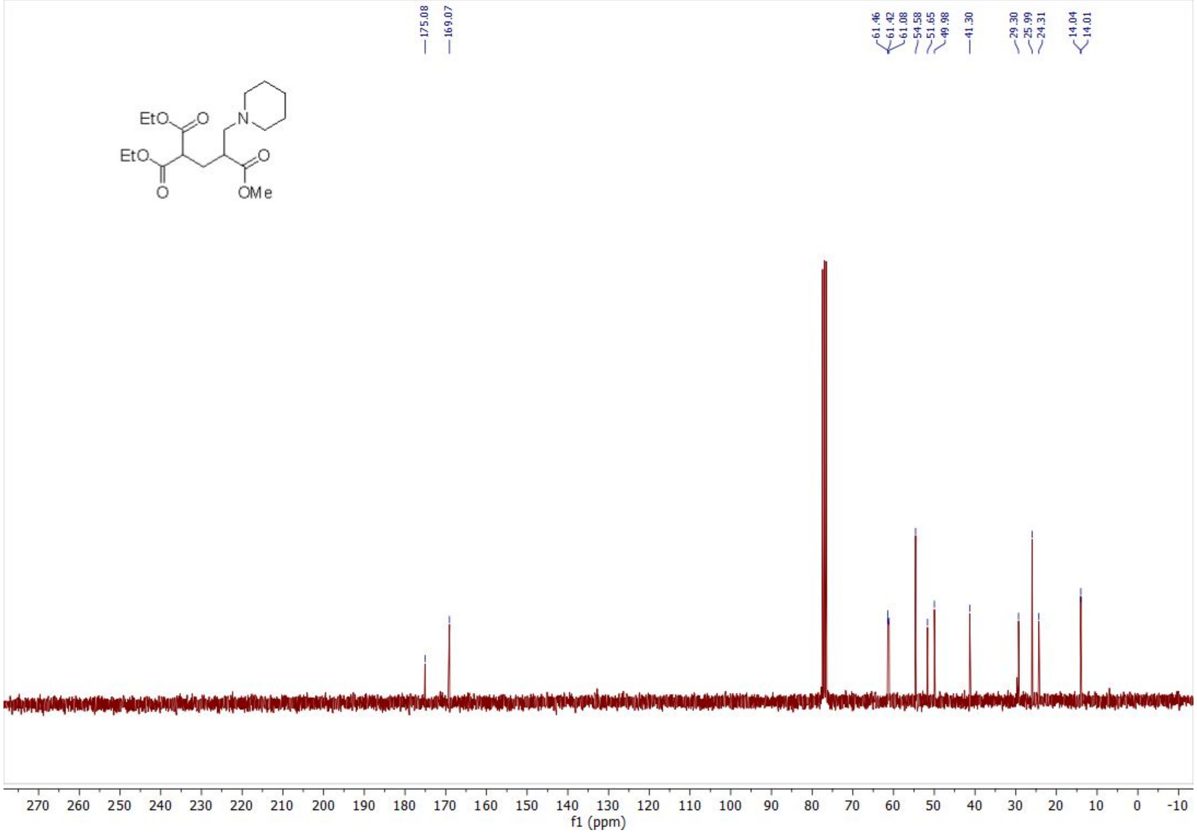

methyl 11-(diethylamino)-11-oxo-2-(piperidin-1-ylmethyl)undecanoate (3q)

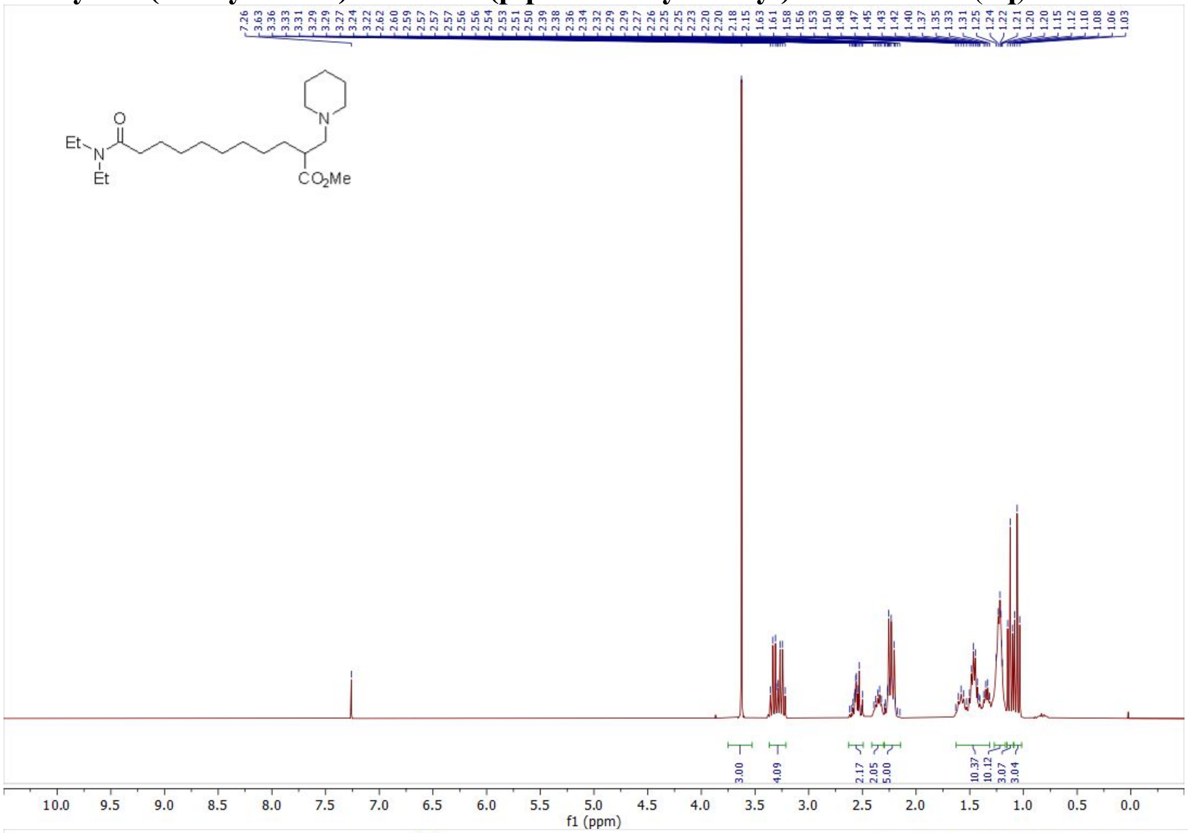

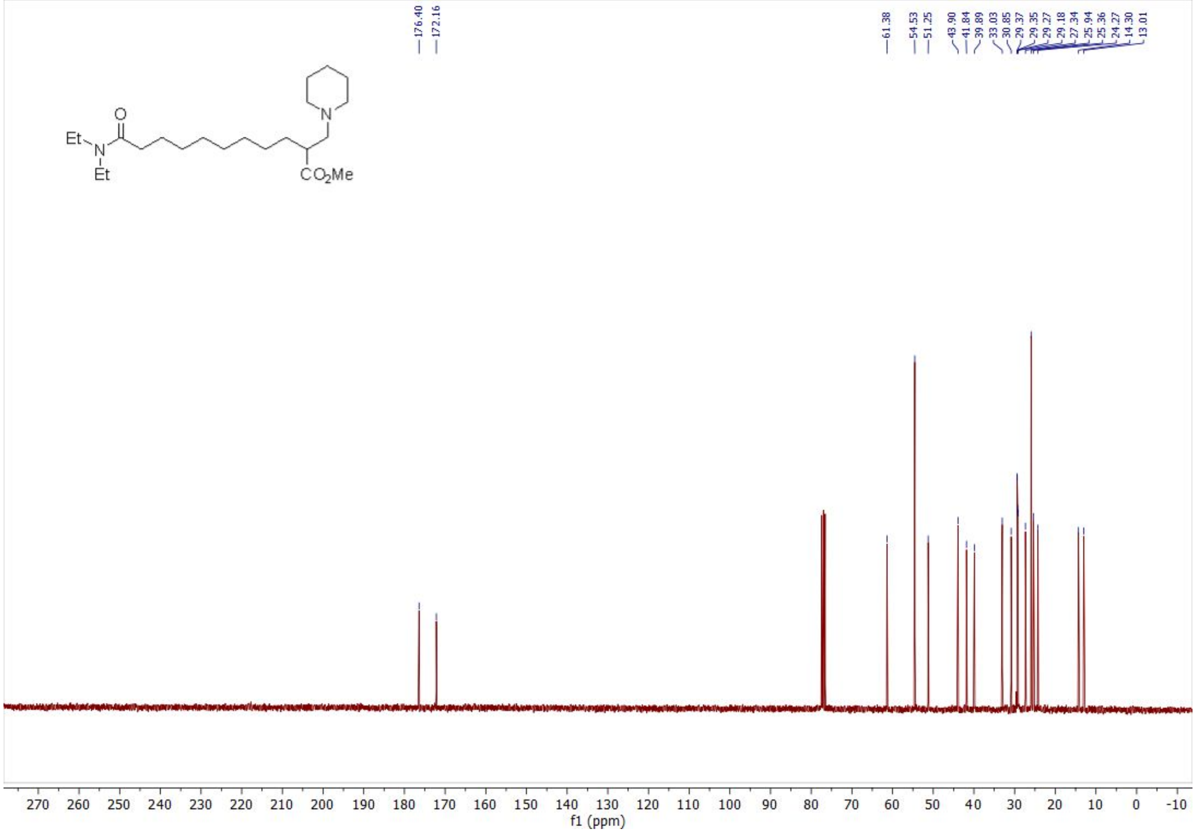

methyl 8-(1,3-dioxoisoindolin-2-yl)-2-(piperidin-1-ylmethyl)octanoate (3r)

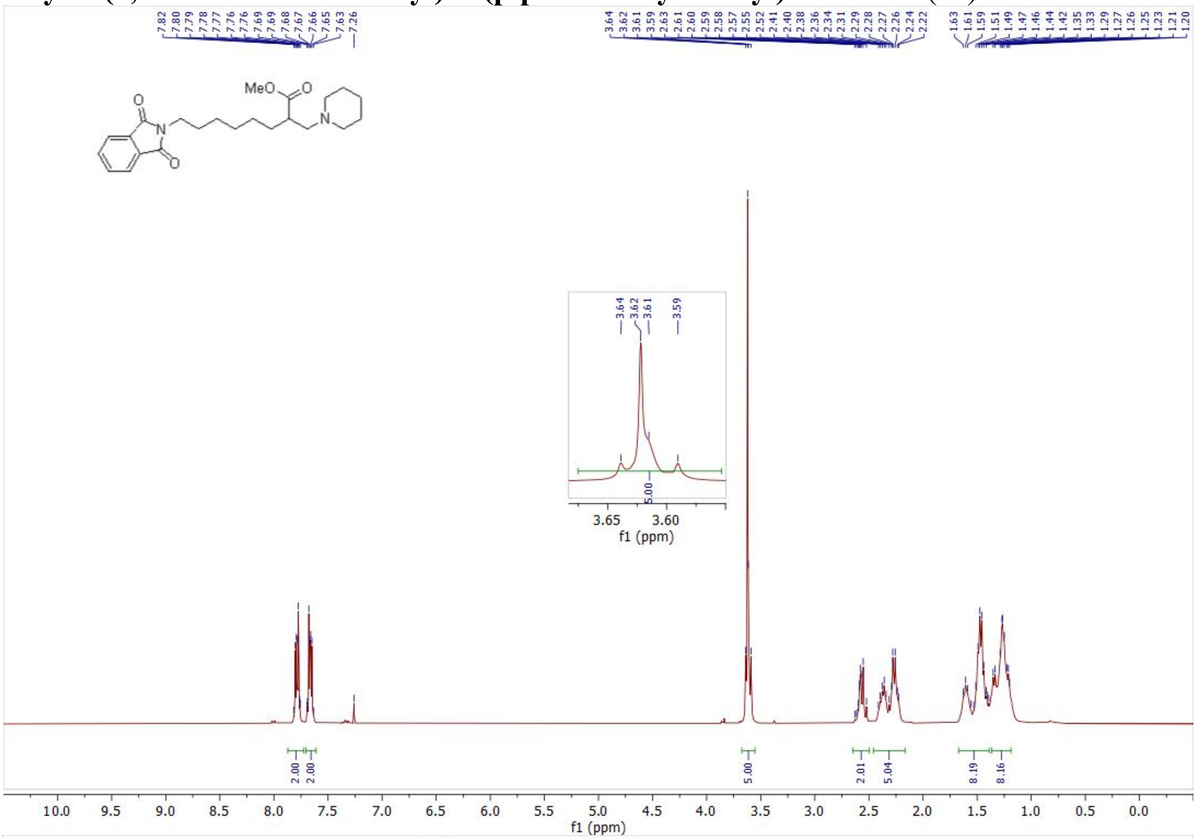

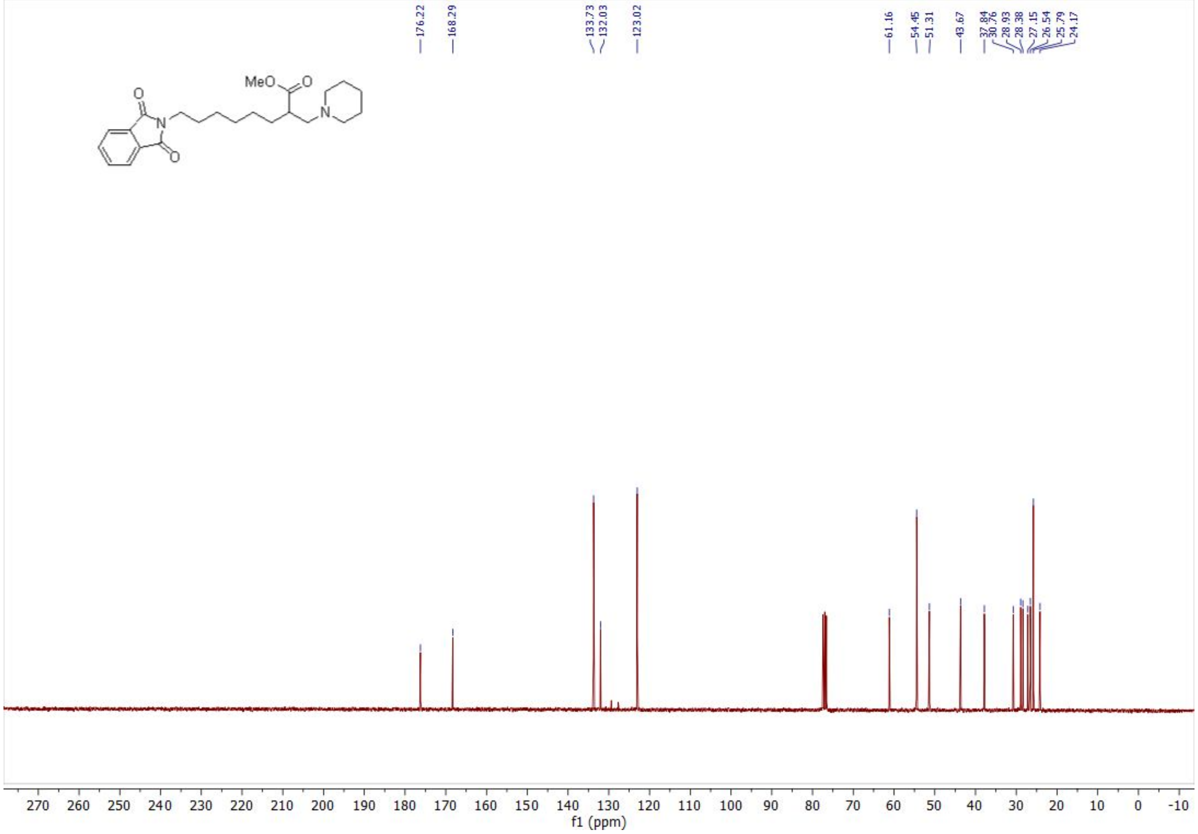

1-methyl 11-(pent-3-yn-1-yl) 2-(piperidin-1-ylmethyl)undecanedioate (3s)

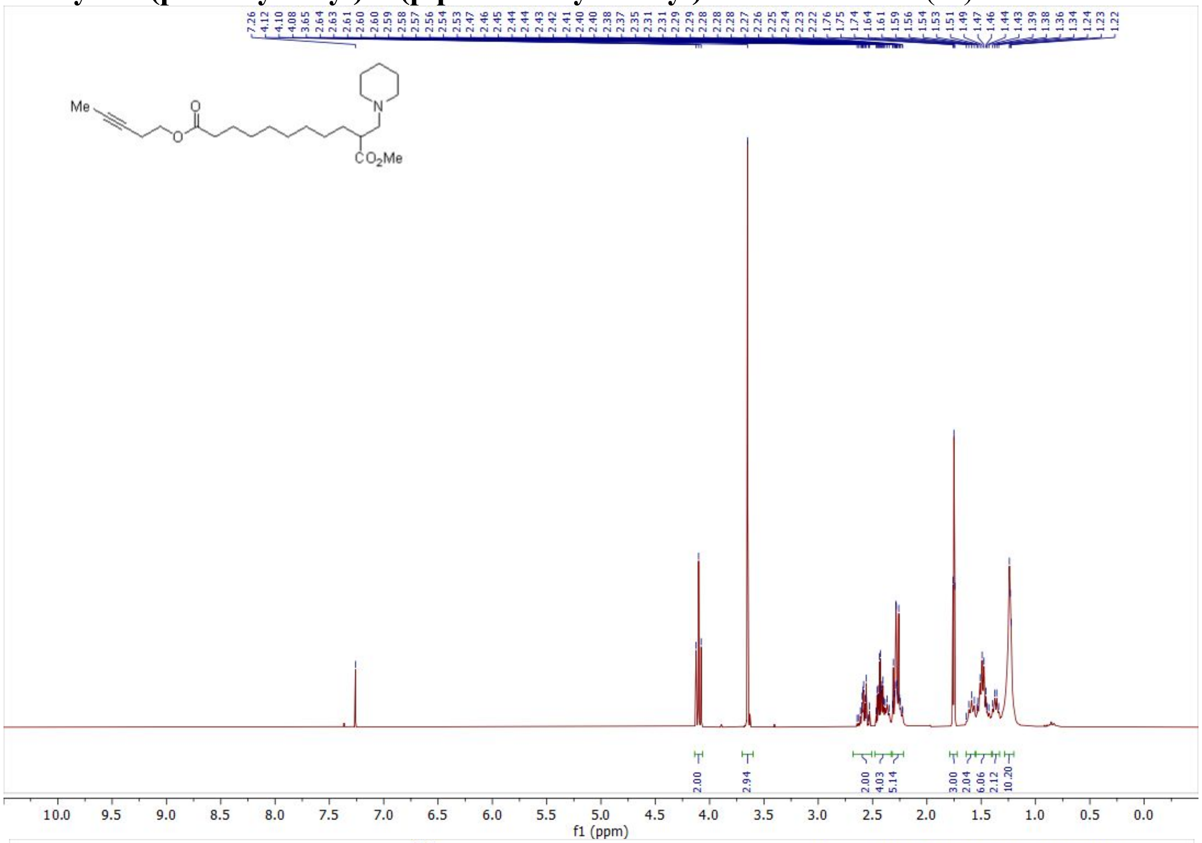

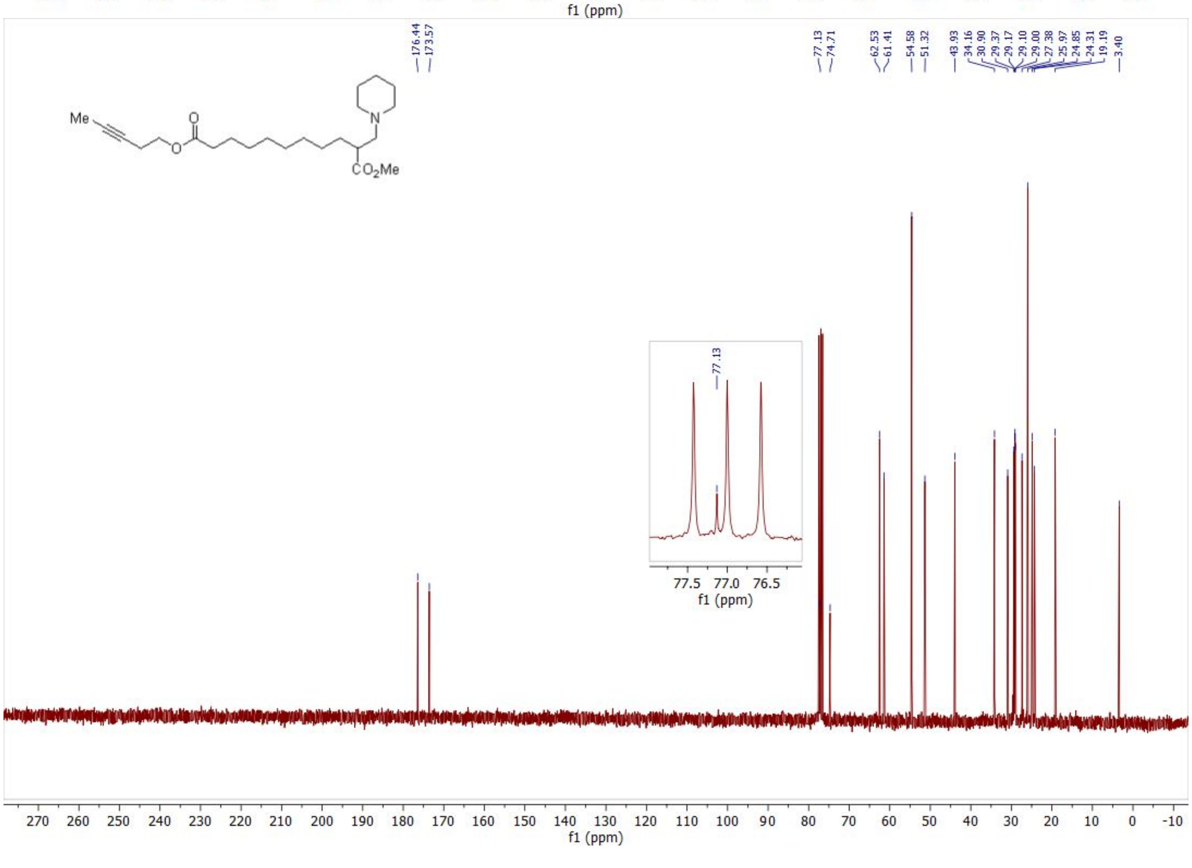

methyl 2-(piperidin-1-yl)cyclopentane-1-carboxylate (3t)

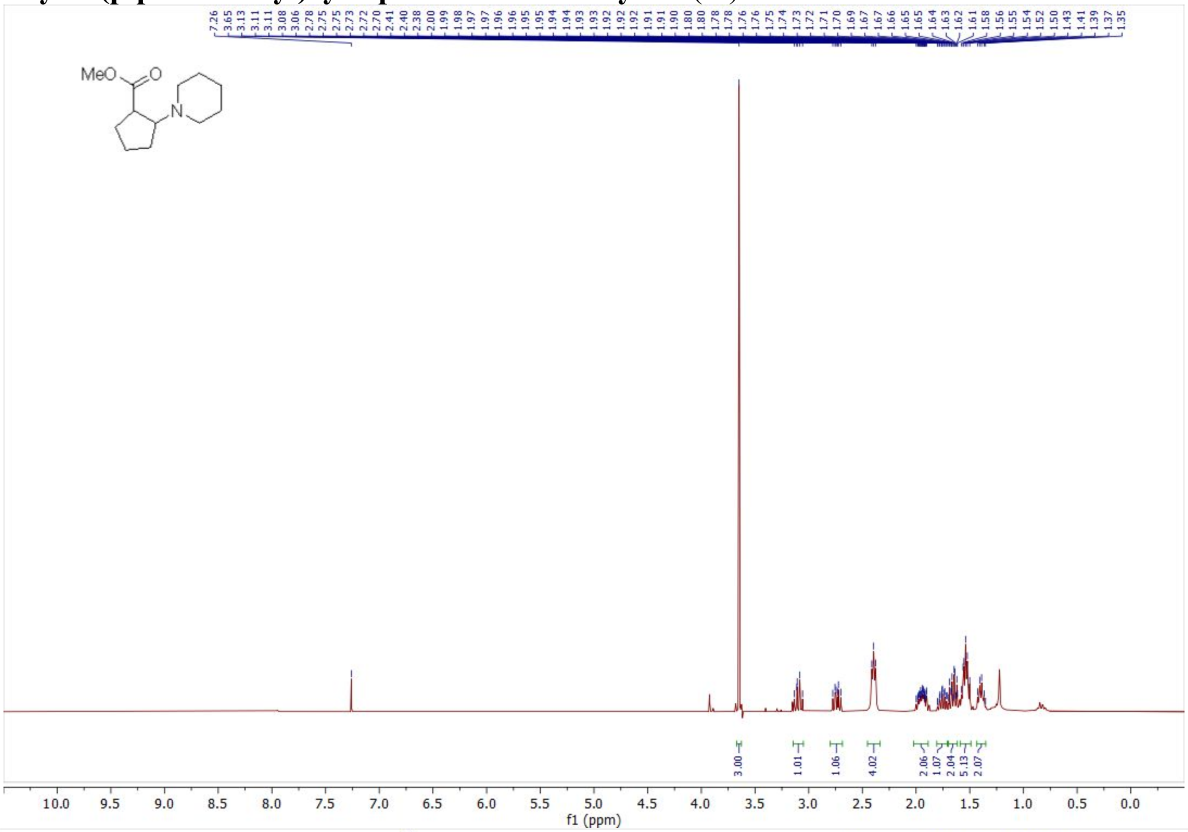

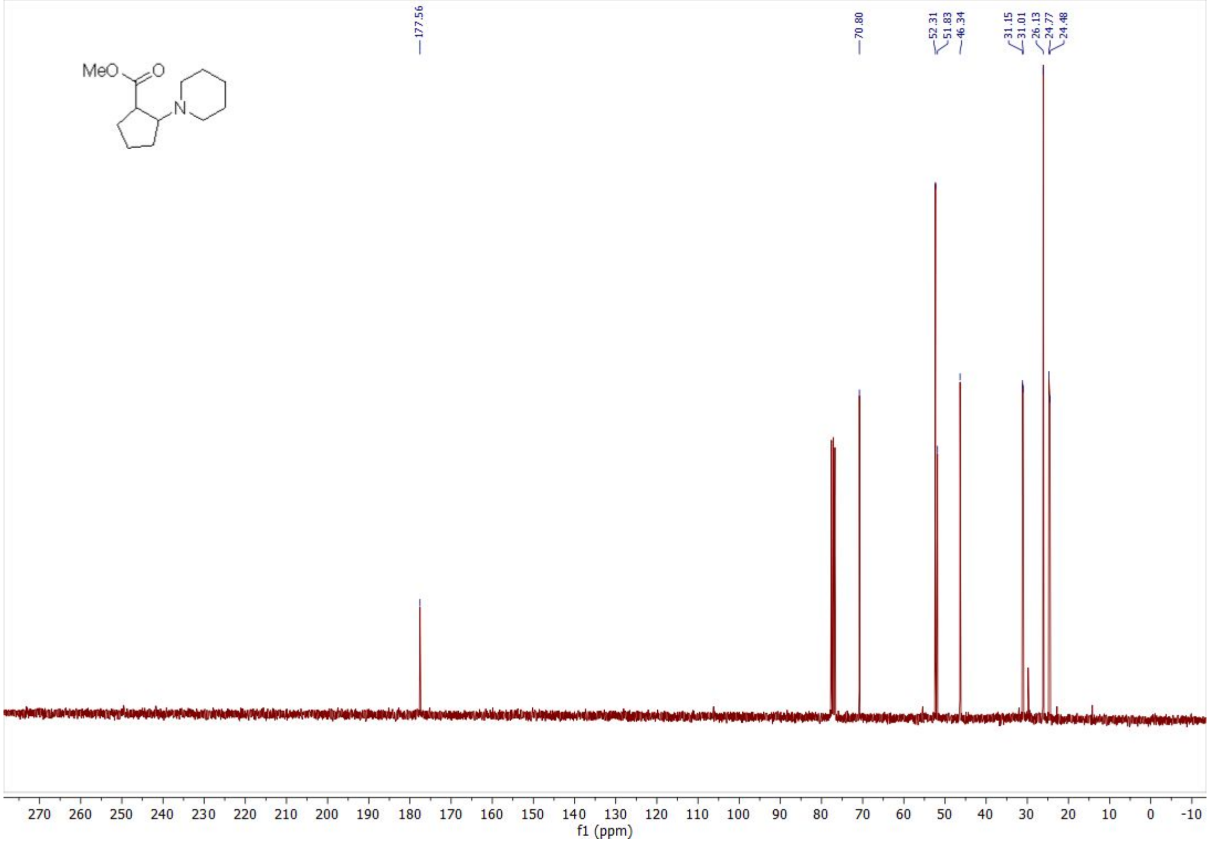

1,1-diethyl 3-methyl 4-(piperidin-1-yl)cyclopentane-1,1,3-tricarboxylate (3u)

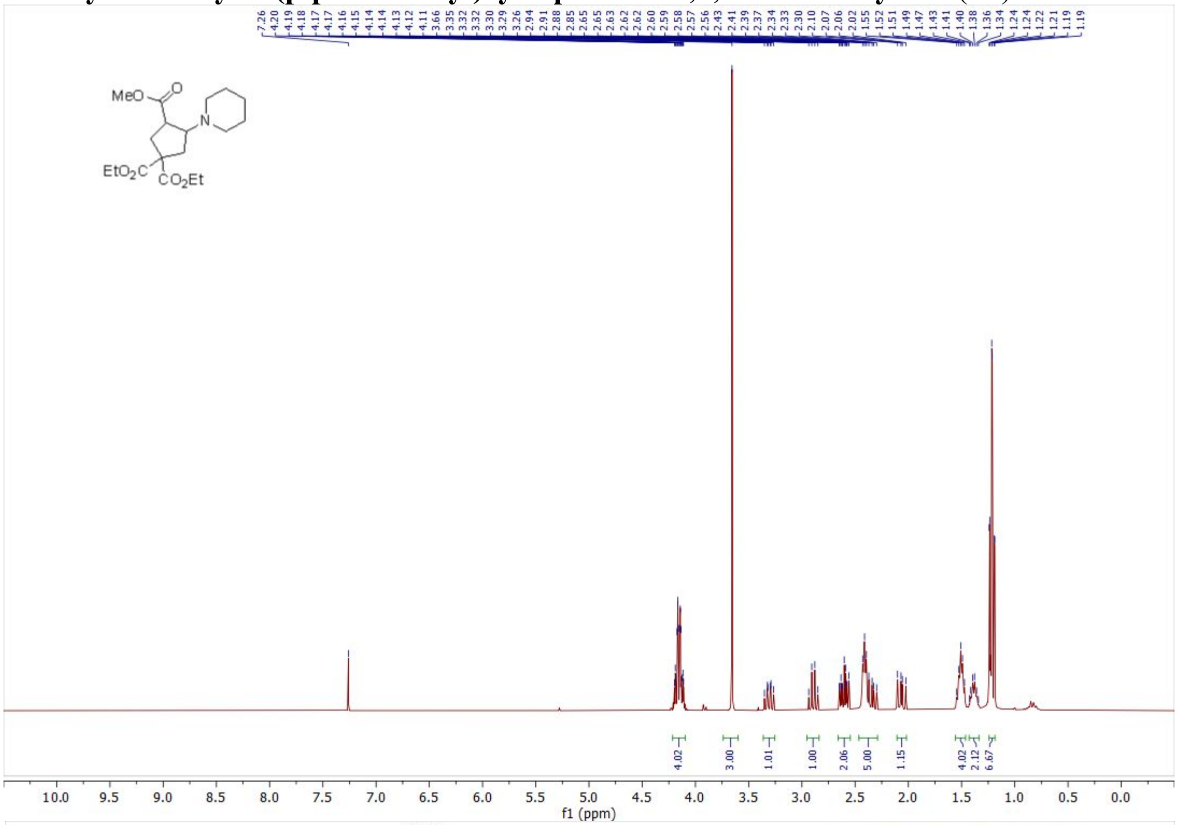

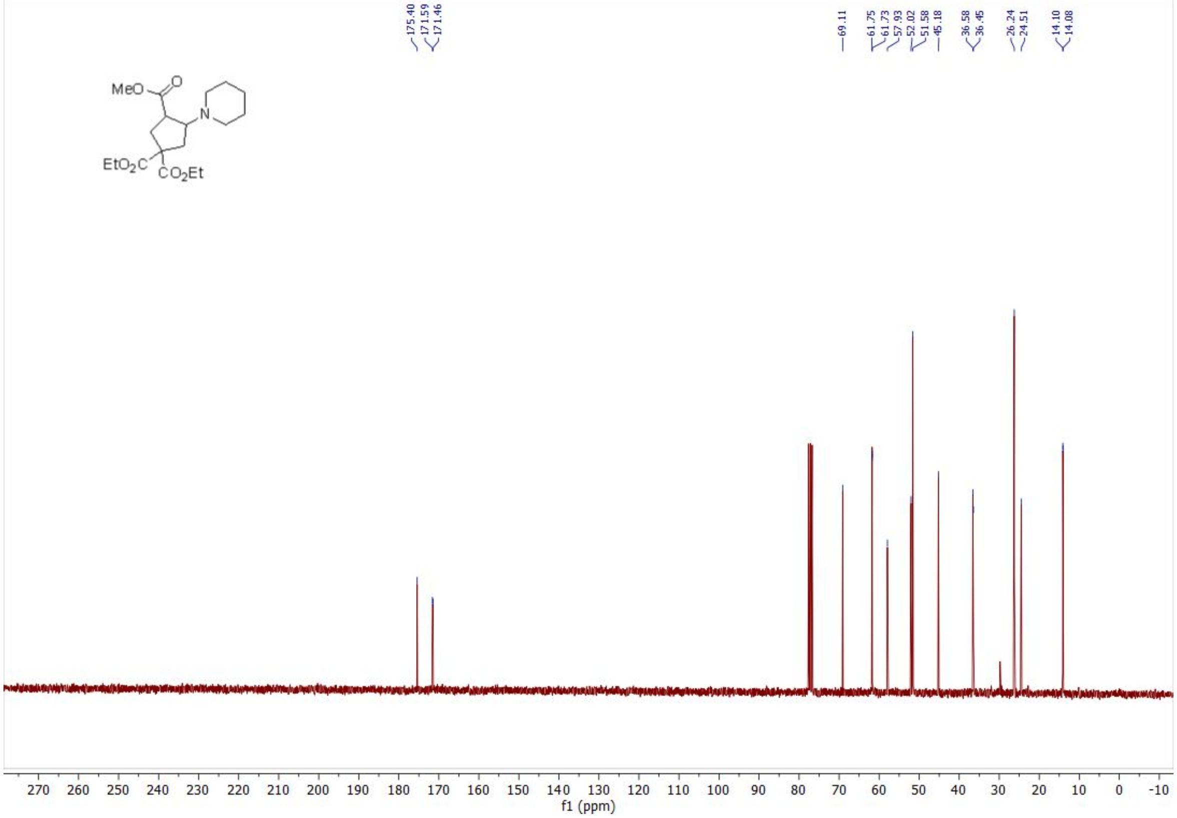

methyl 2-(piperidin-1-yl)cyclohexane-1-carboxylate (3v)

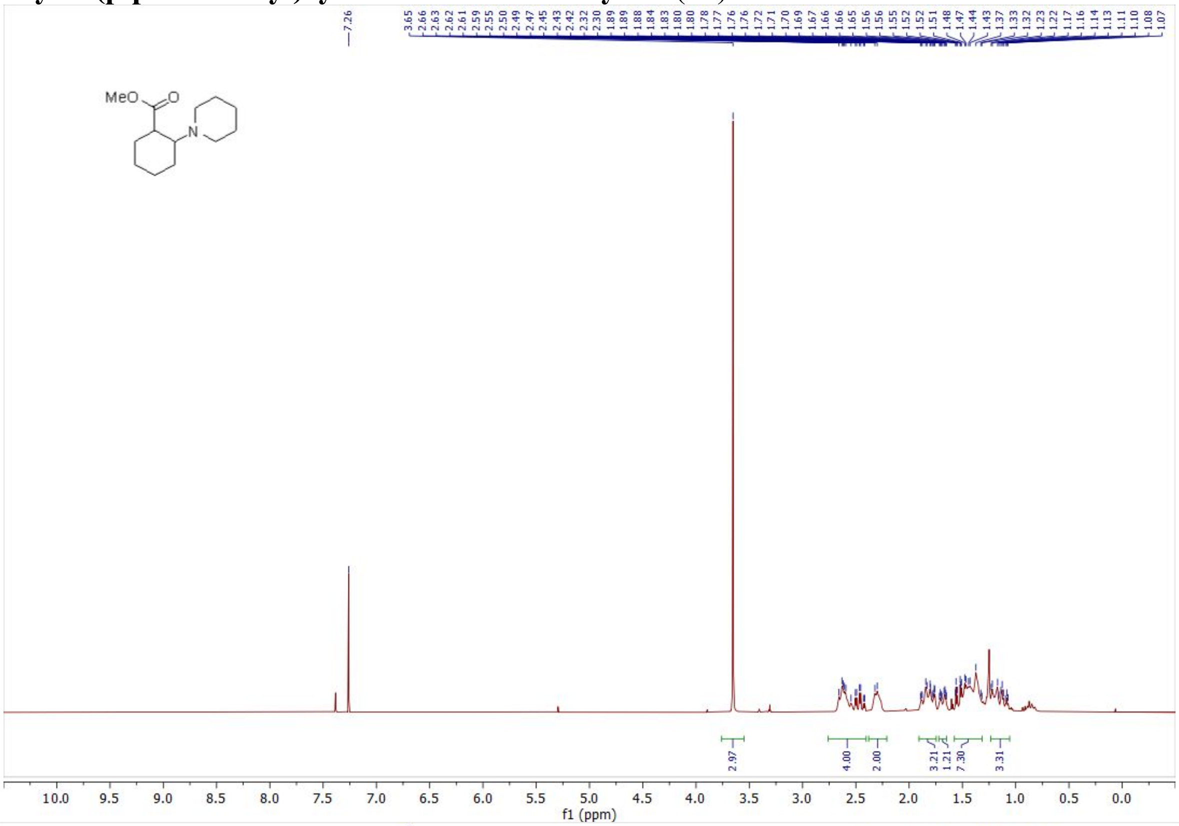

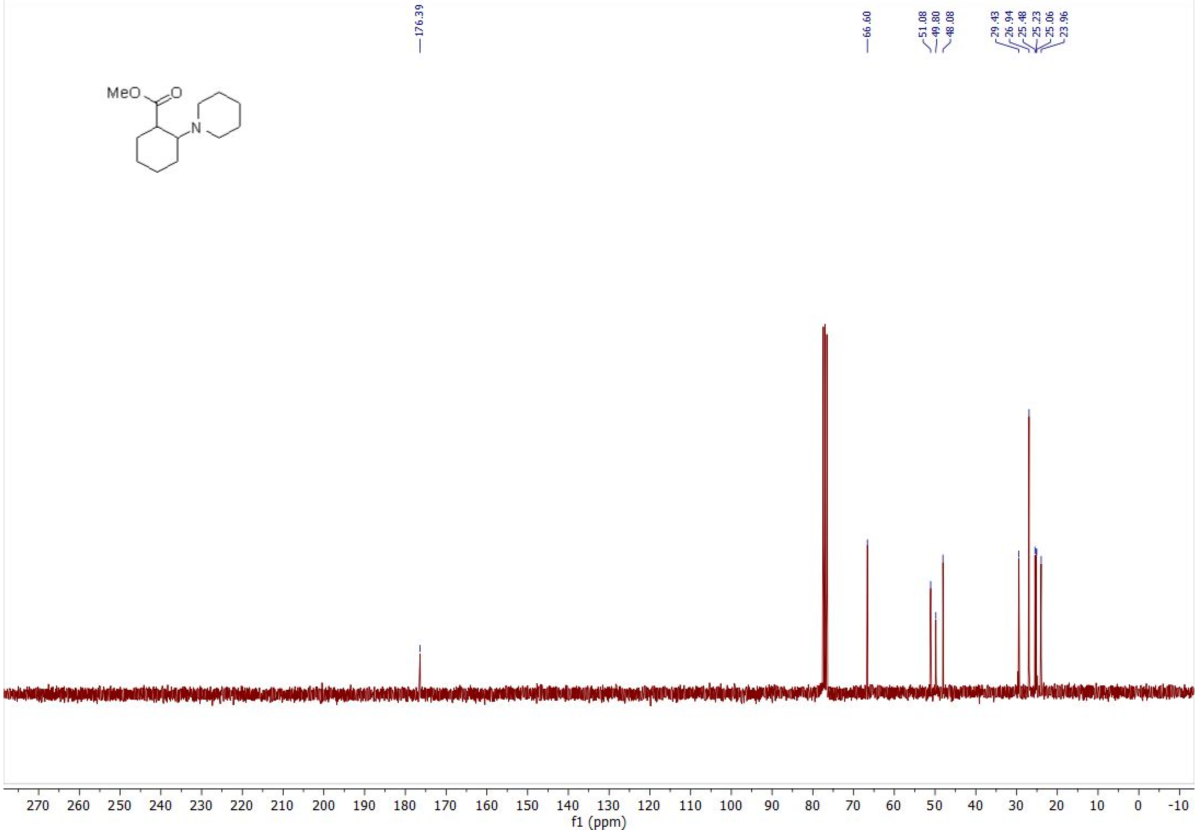

methyl 2-((4-methylpiperidin-1-yl)methyl)dodecanoate (3w)

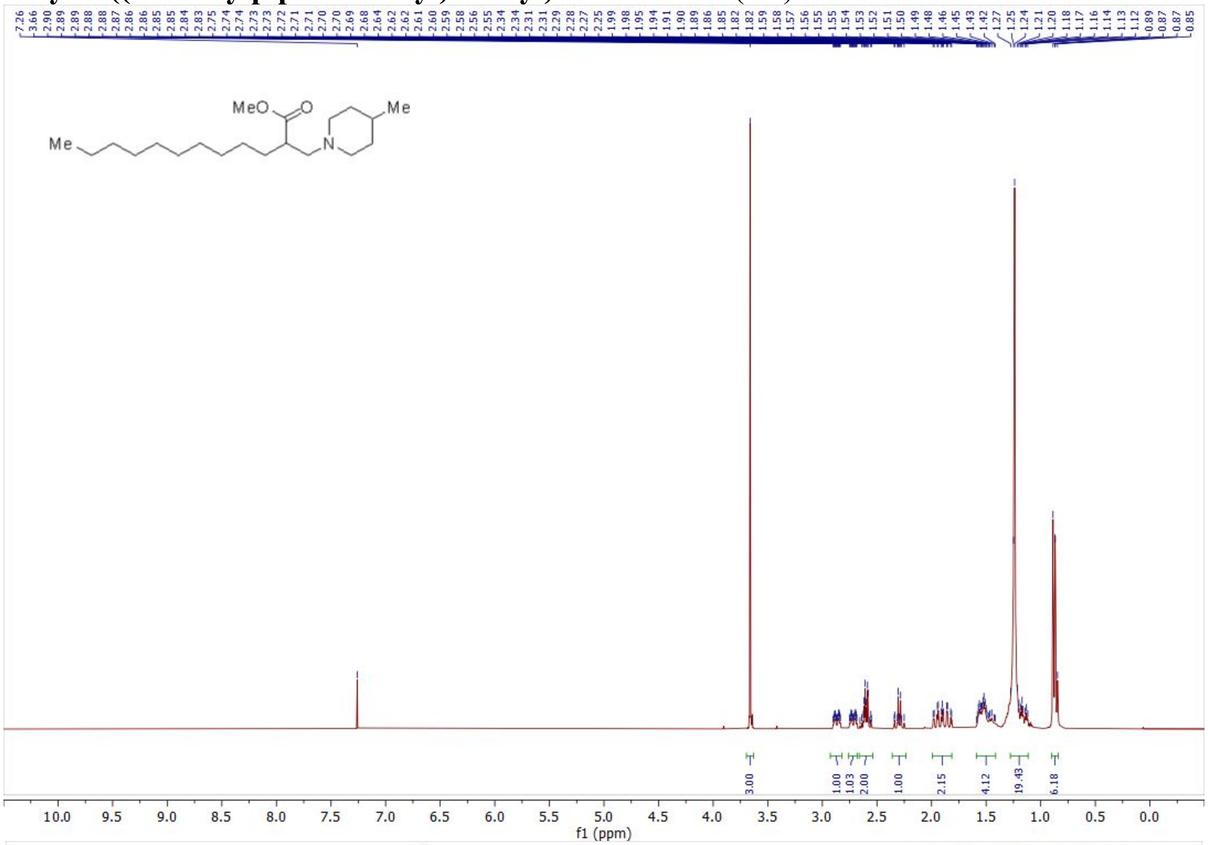

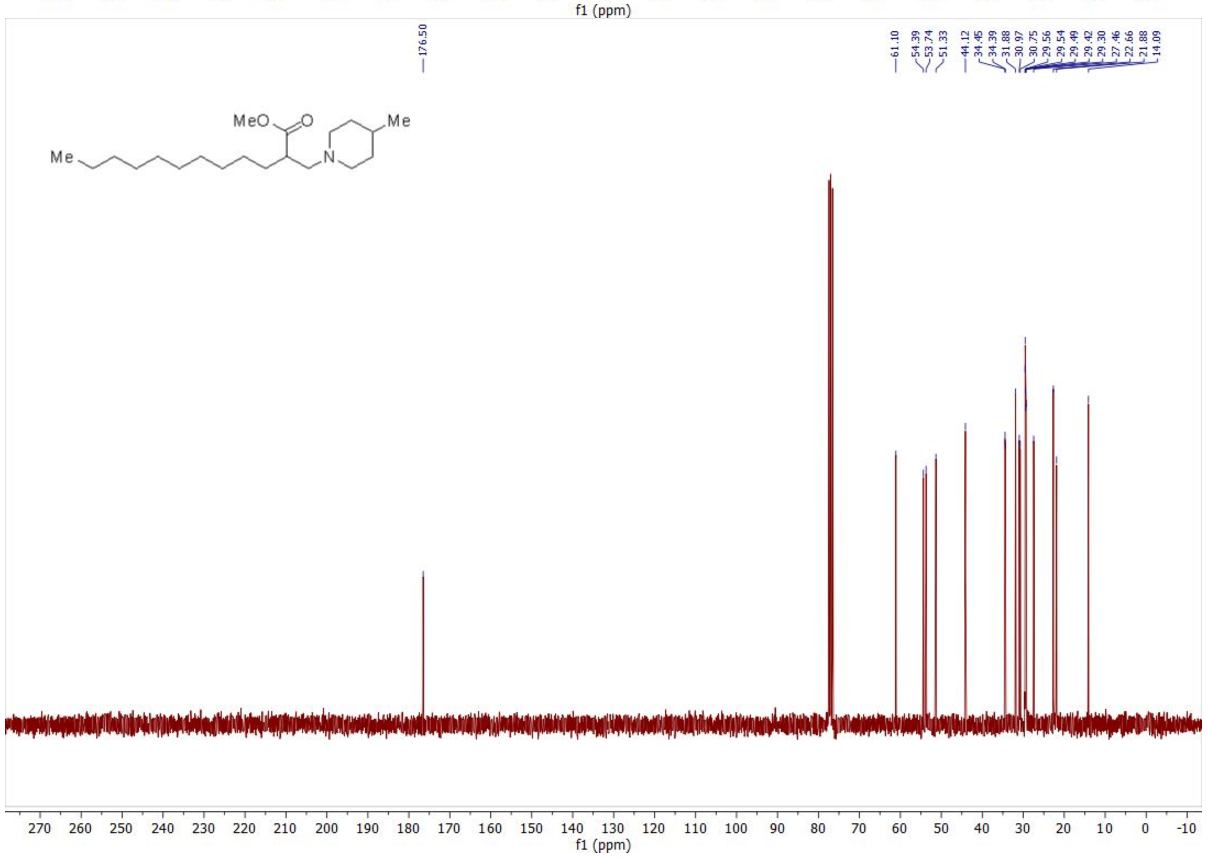

ethyl 1-(2-(methoxycarbonyl)dodecyl)piperidine-4-carboxylate (3x)

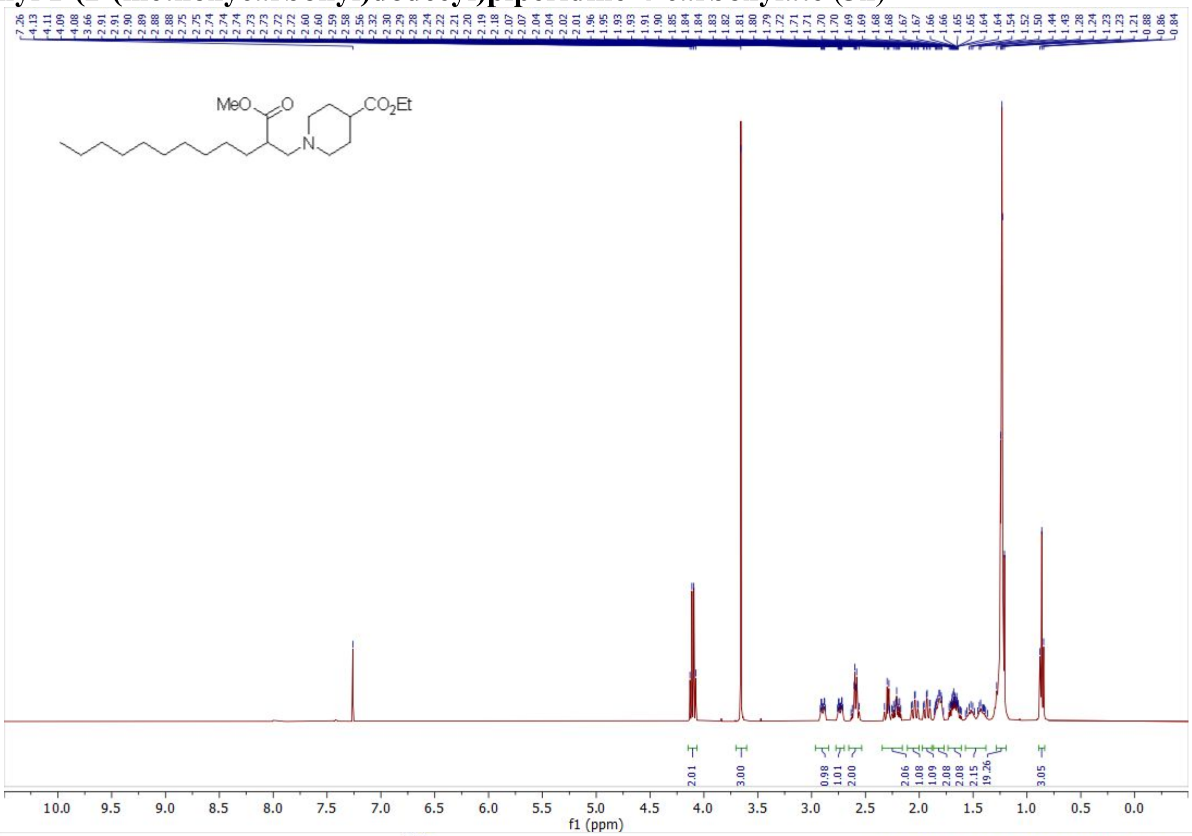

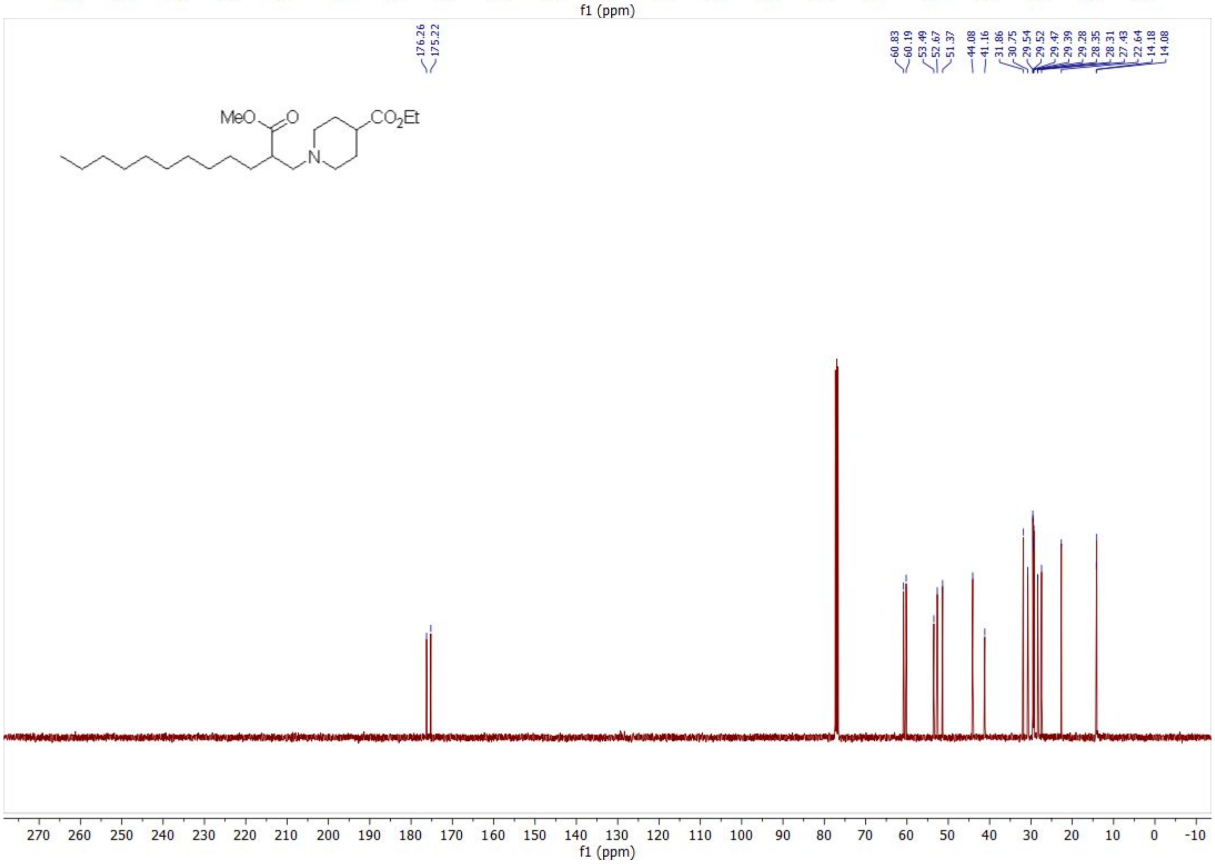

methyl 2-(morpholinomethyl)dodecanoate (3y)

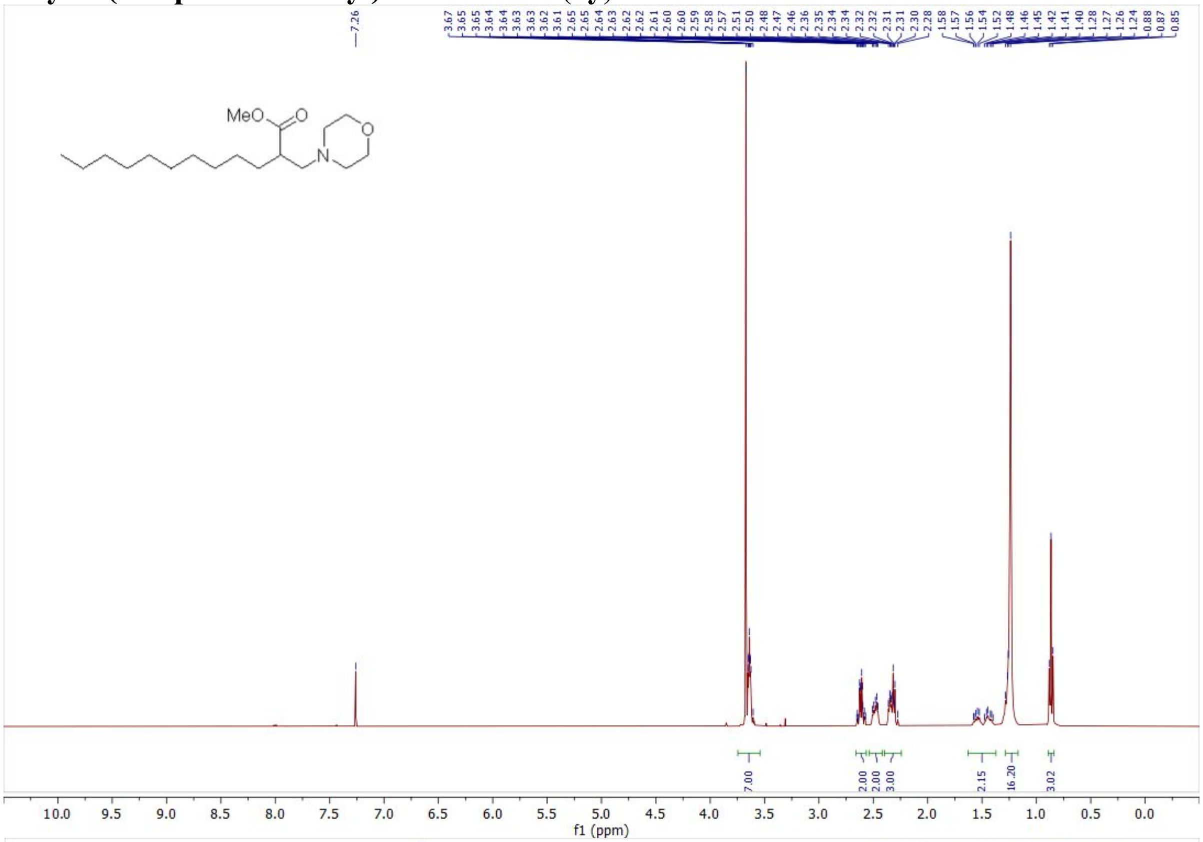

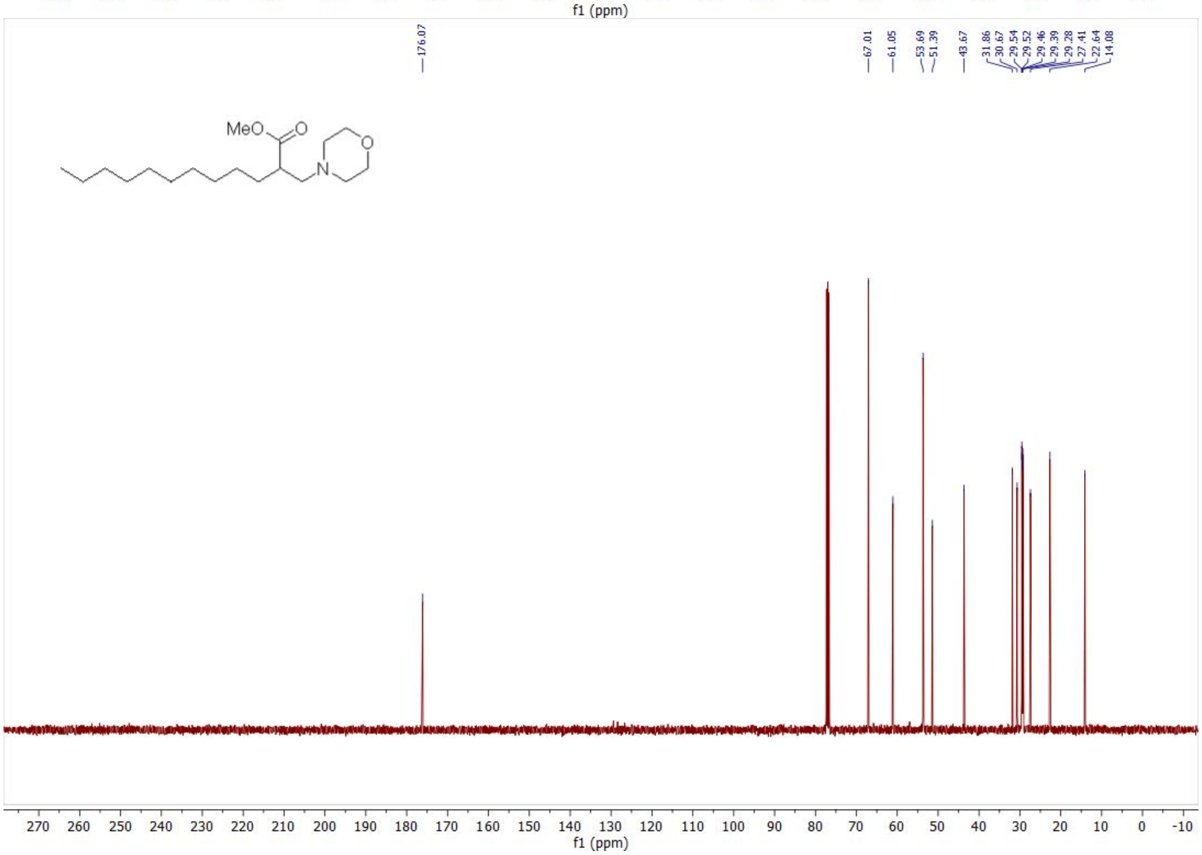

methyl 2-((2,6-dimethylmorpholino)methyl)dodecanoate (3z)

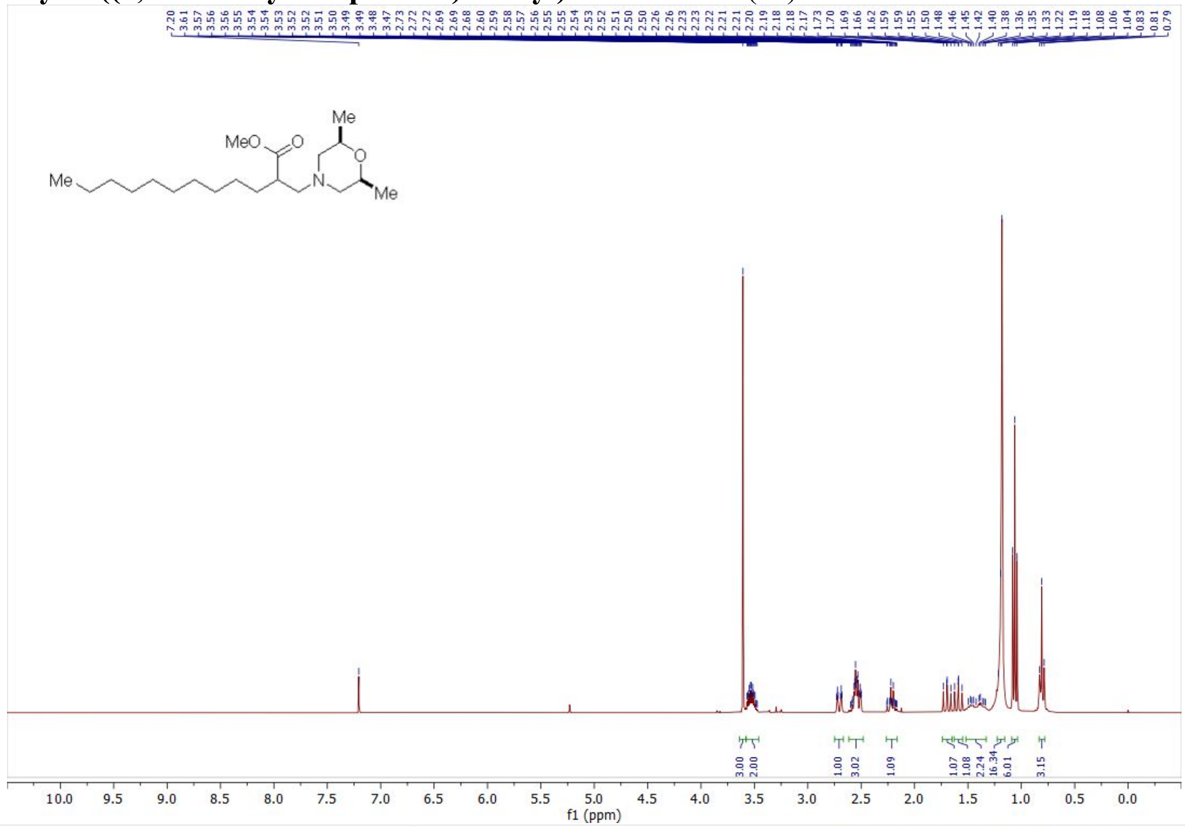

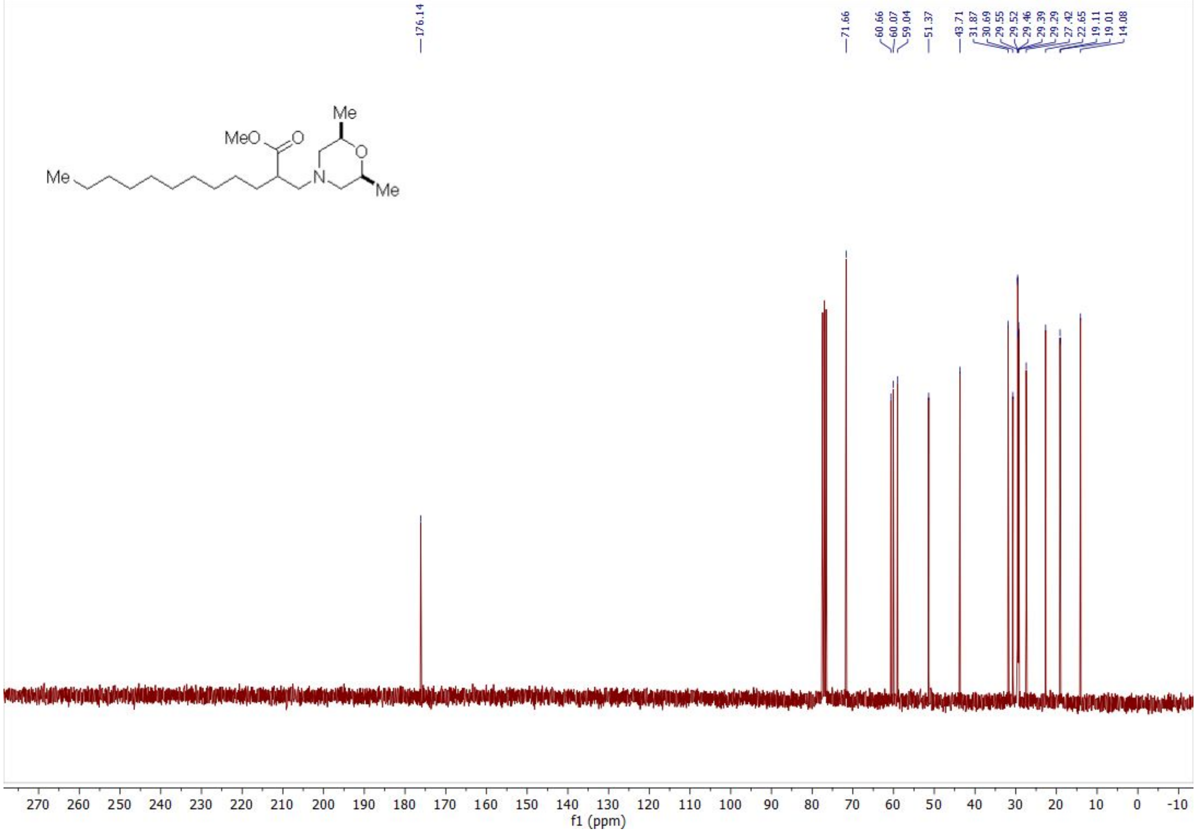

methyl 2-(azepan-1-ylmethyl)dodecanoate (3aa)

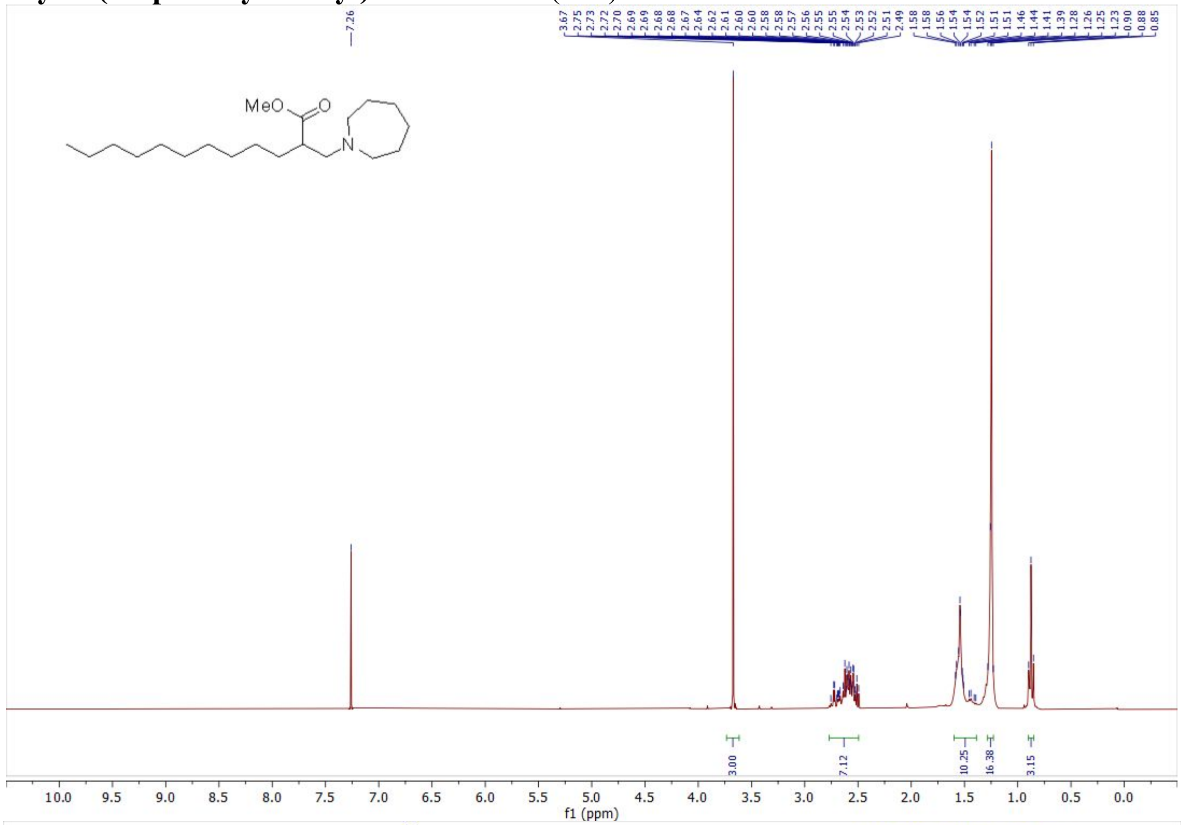

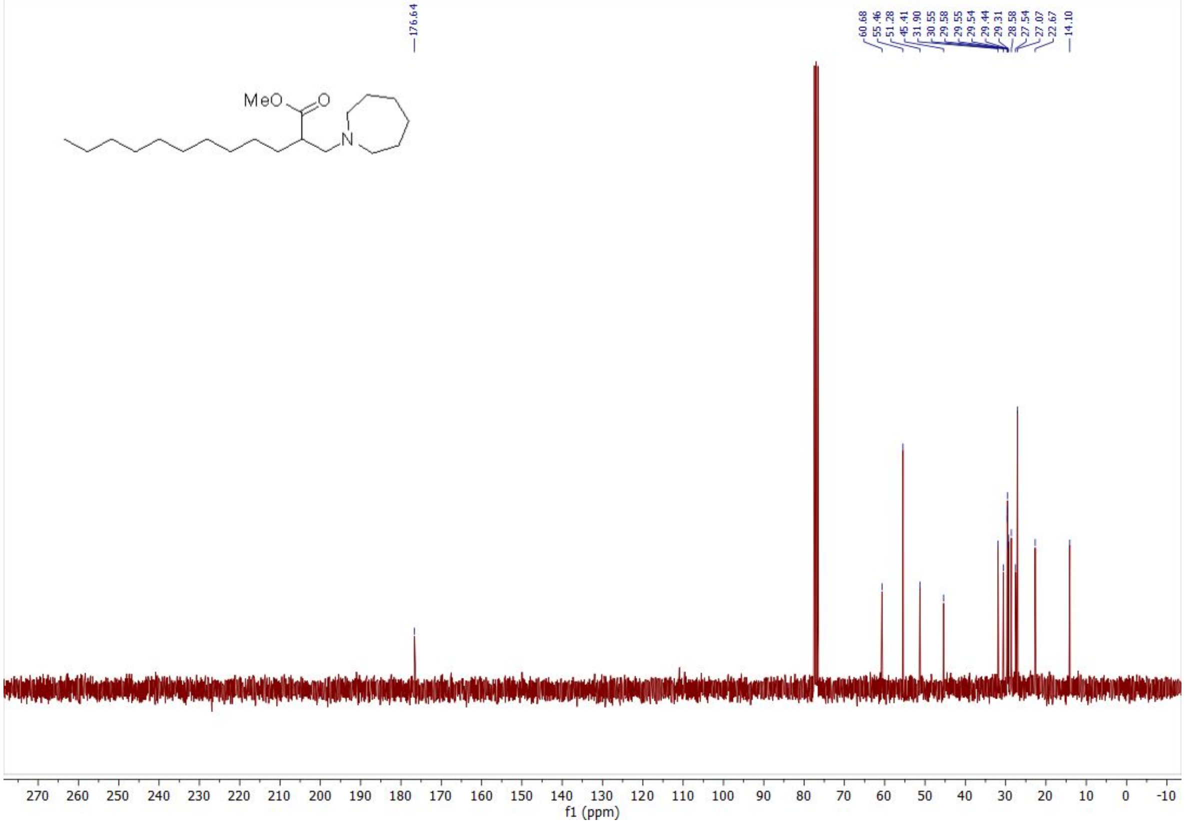

methyl 2-(pyrrolidin-1-ylmethyl)dodecanoate (3bb)

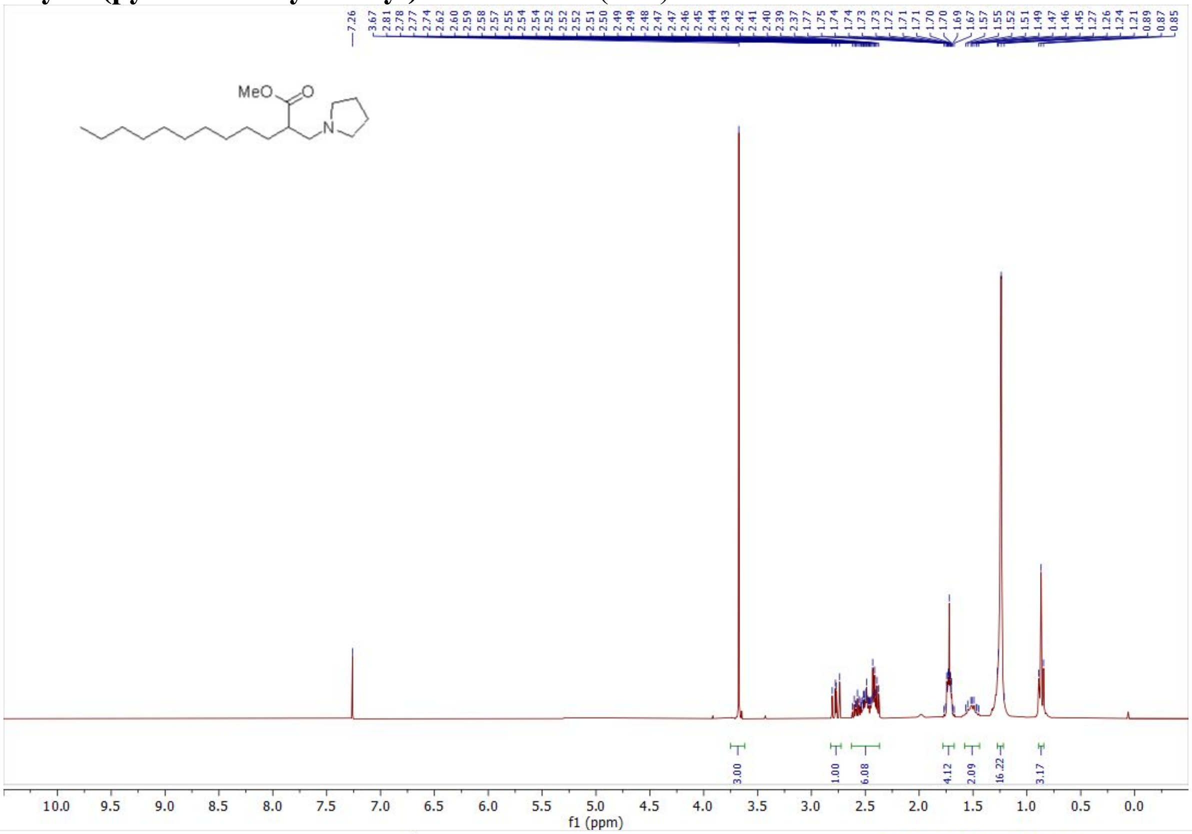

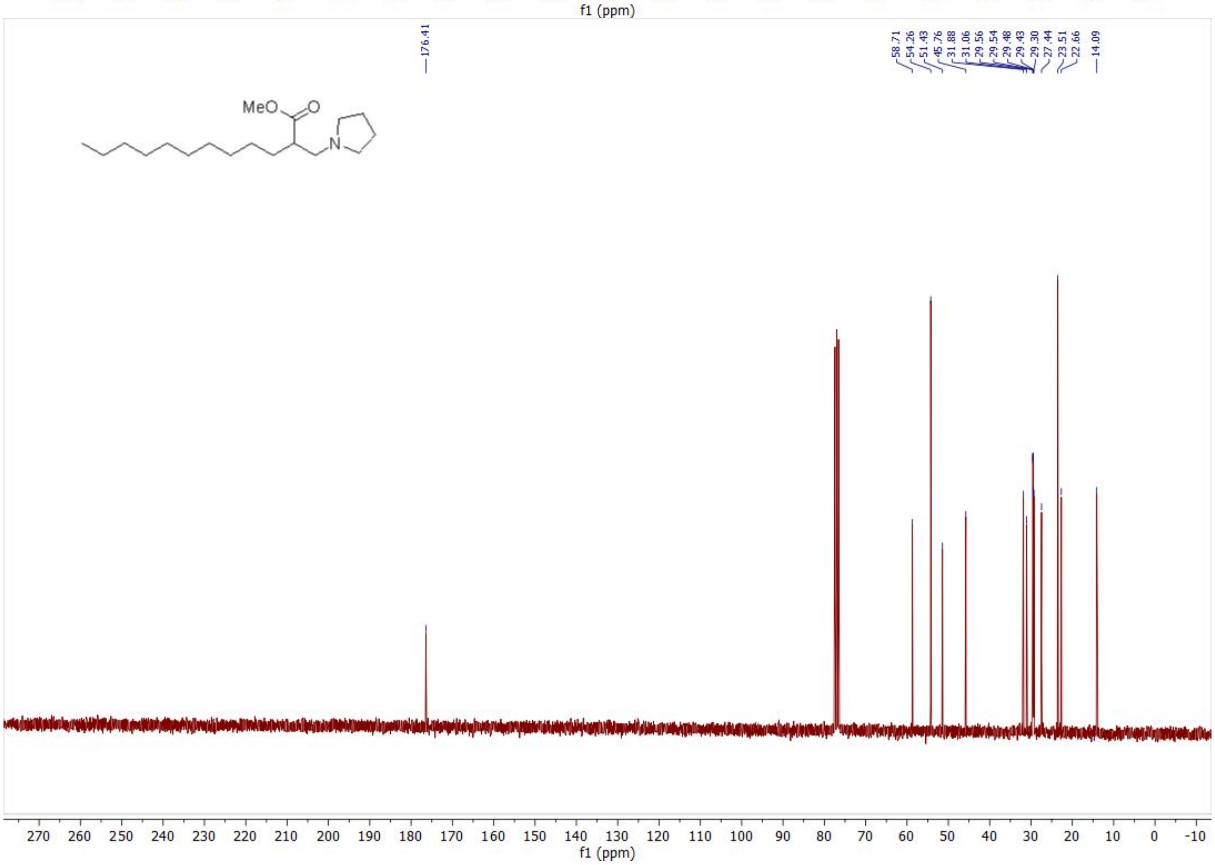

benzyl 4-(2-(methoxycarbonyl)dodecyl)piperazine-1-carboxylate (3cc)

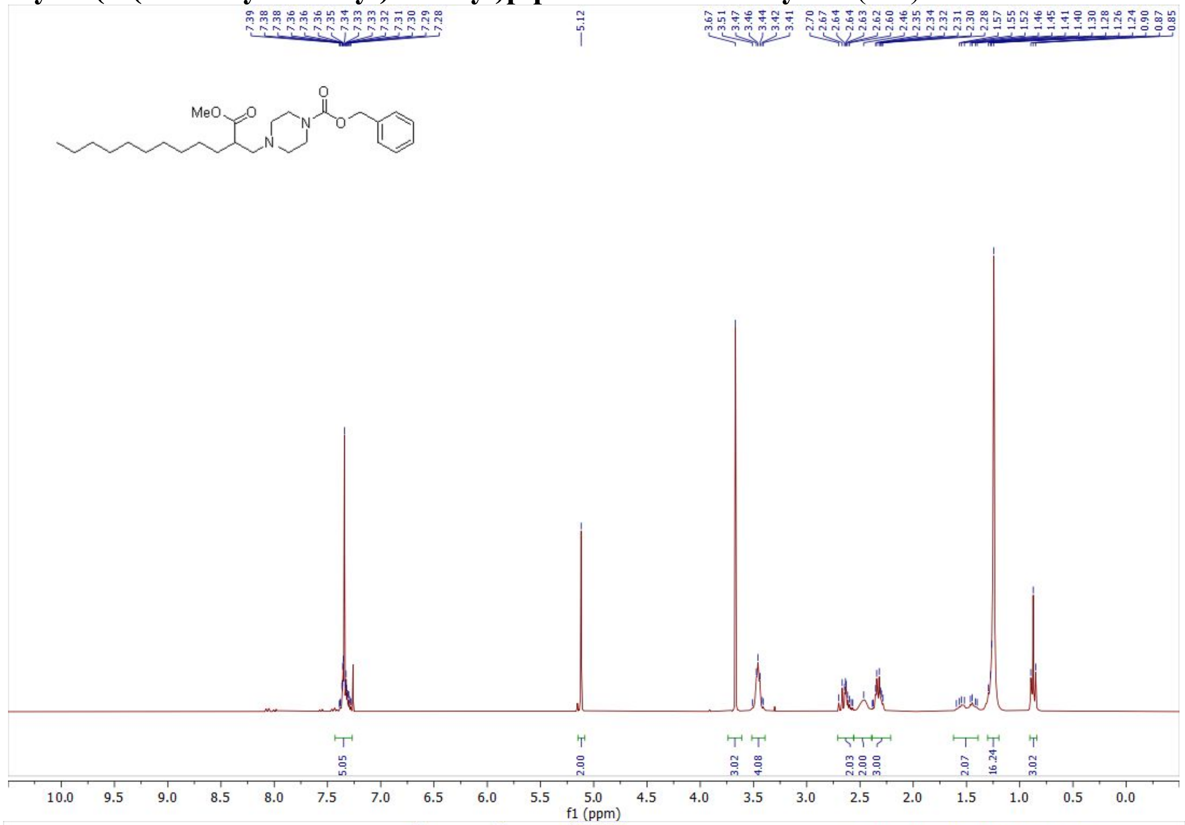

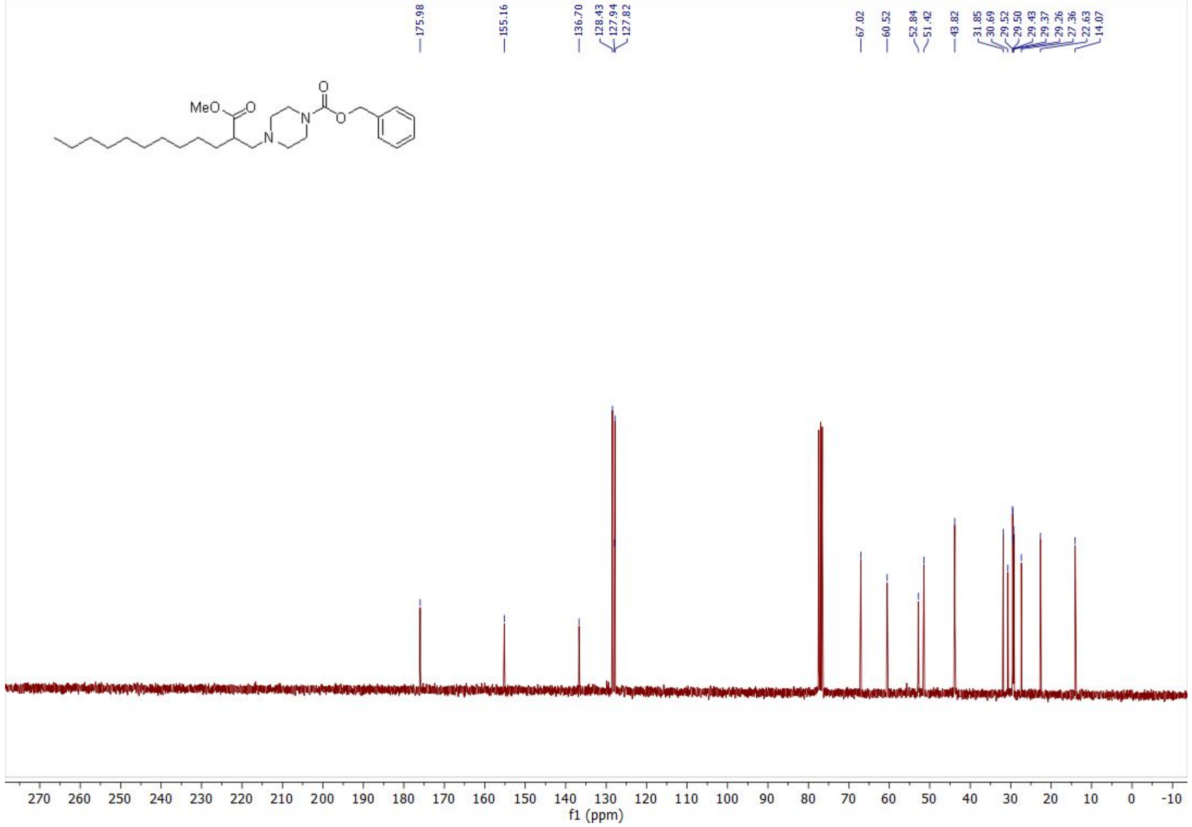

methyl 2-((4-benzoylpiperazin-1-yl)methyl)dodecanoate (3dd)

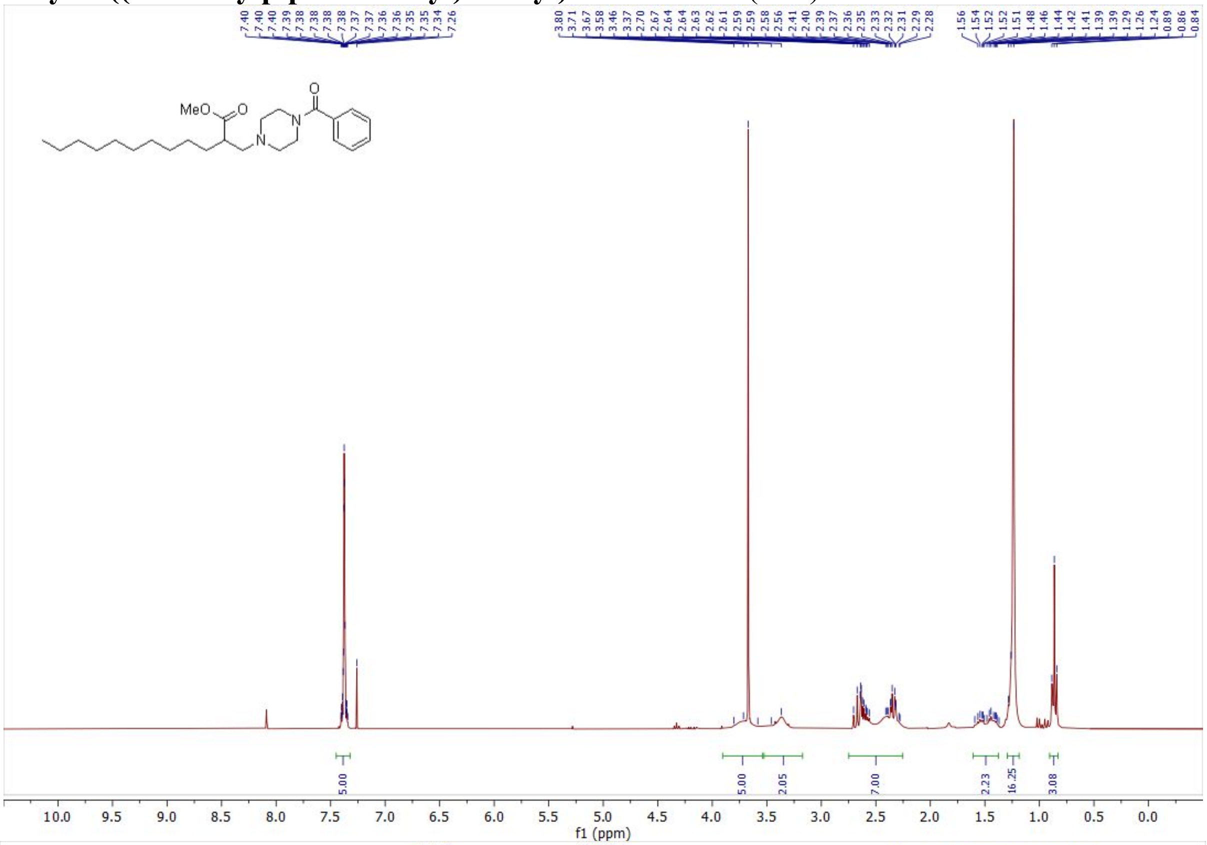

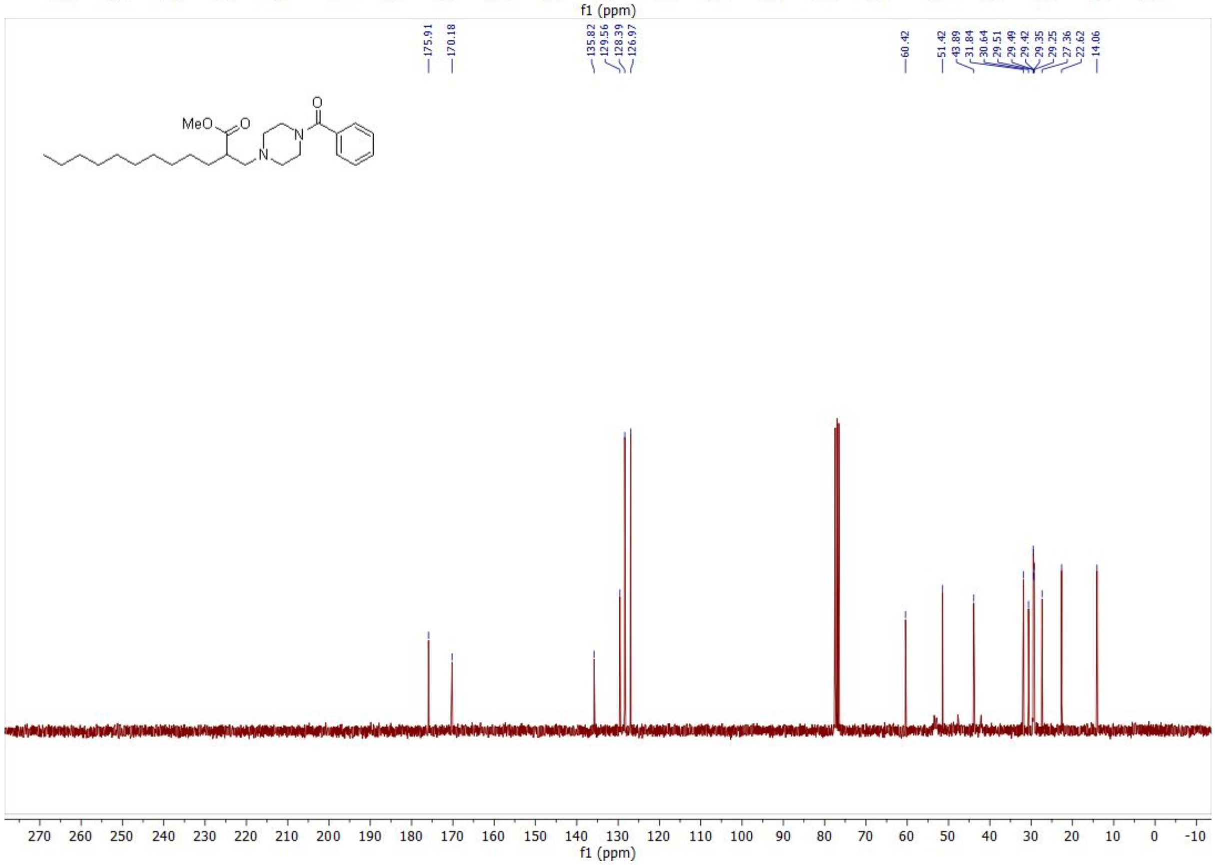

***tert*-butyl 4-(2-(methoxycarbonyl)dodecyl)piperazine-1-carboxylate (3ee)**

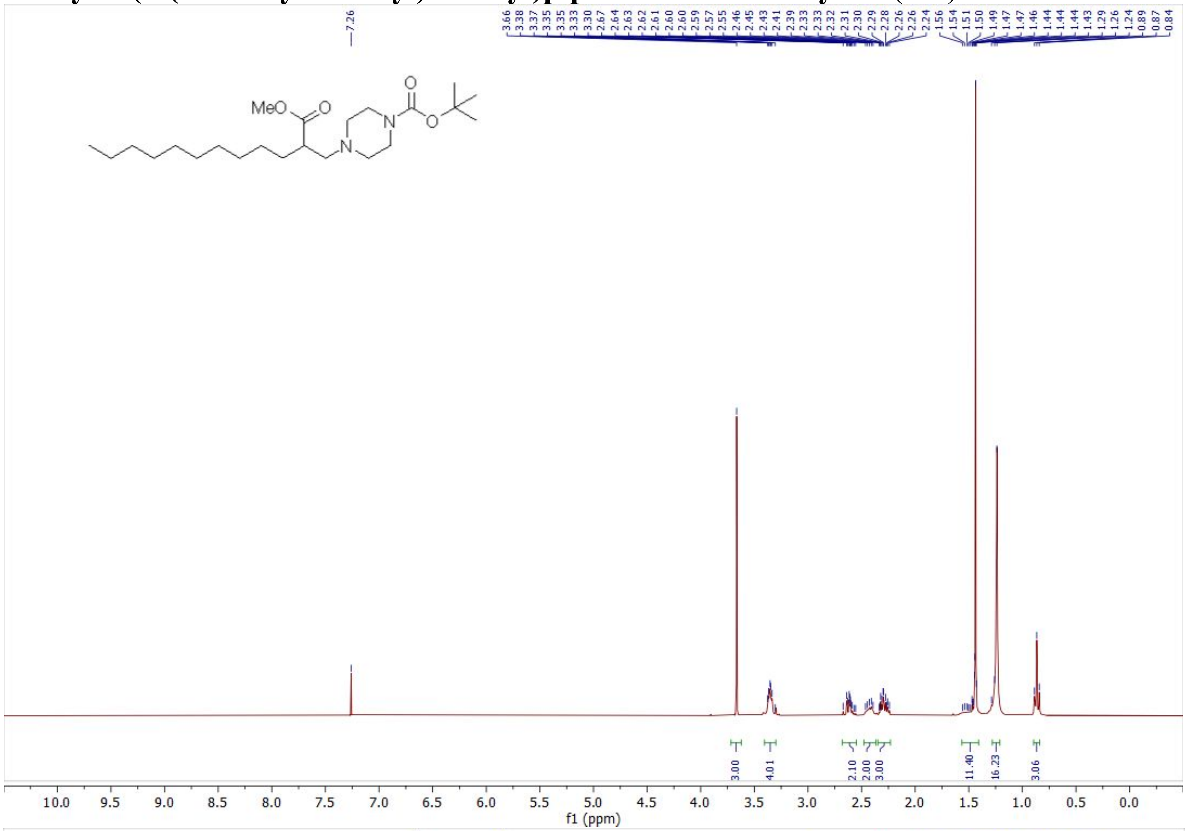

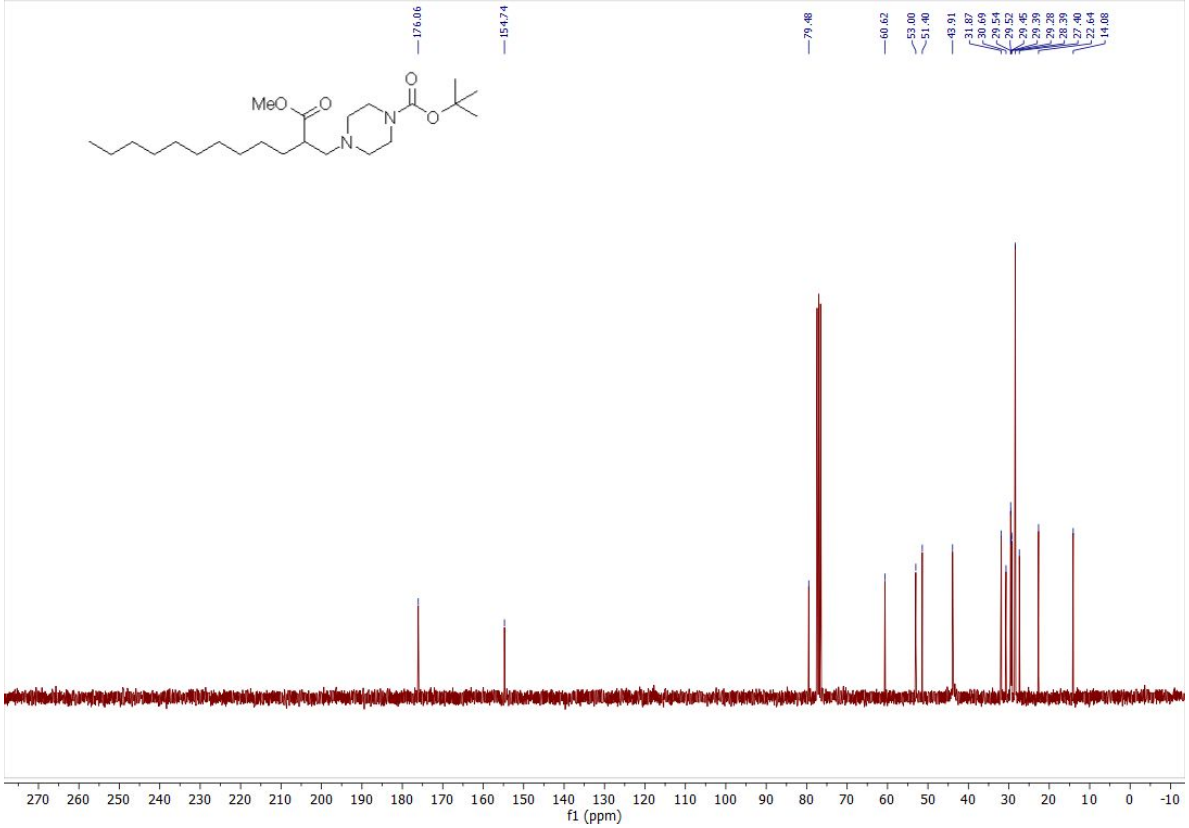

methyl 2-((diethylamino)methyl)dodecanoate (3ff)

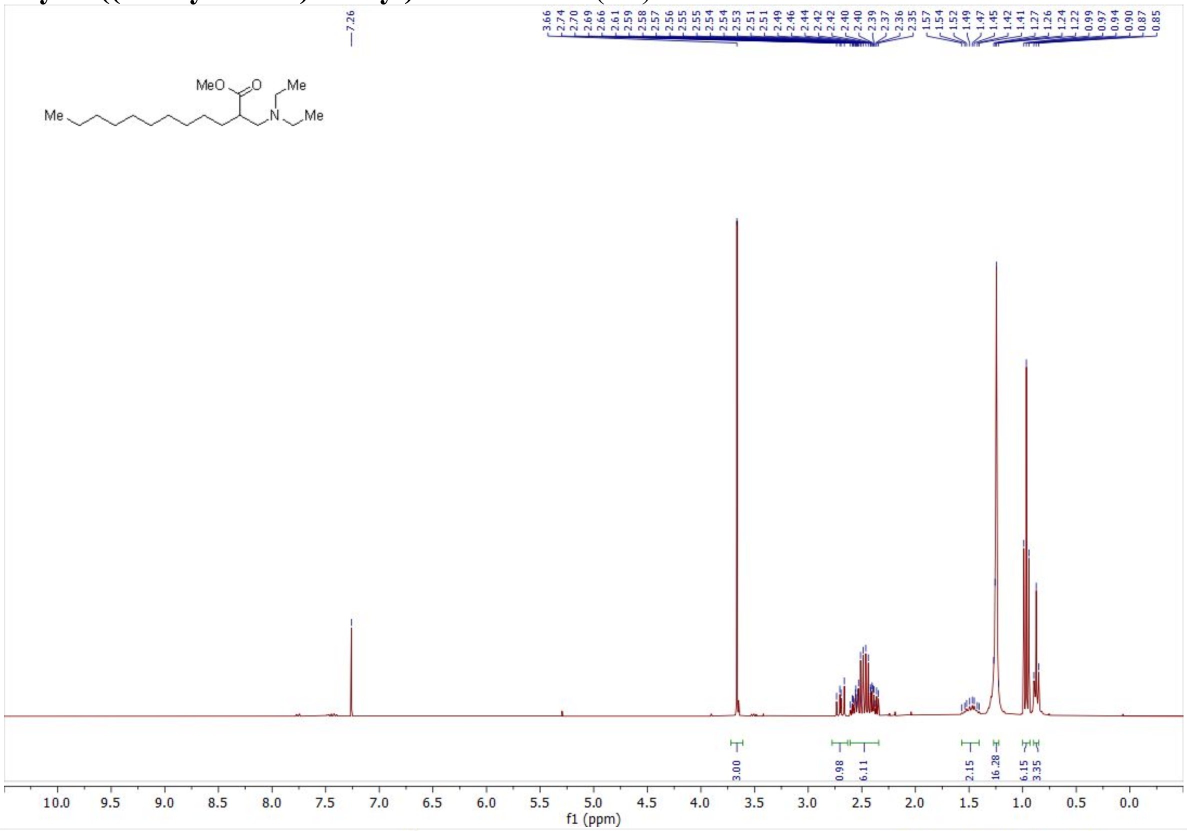

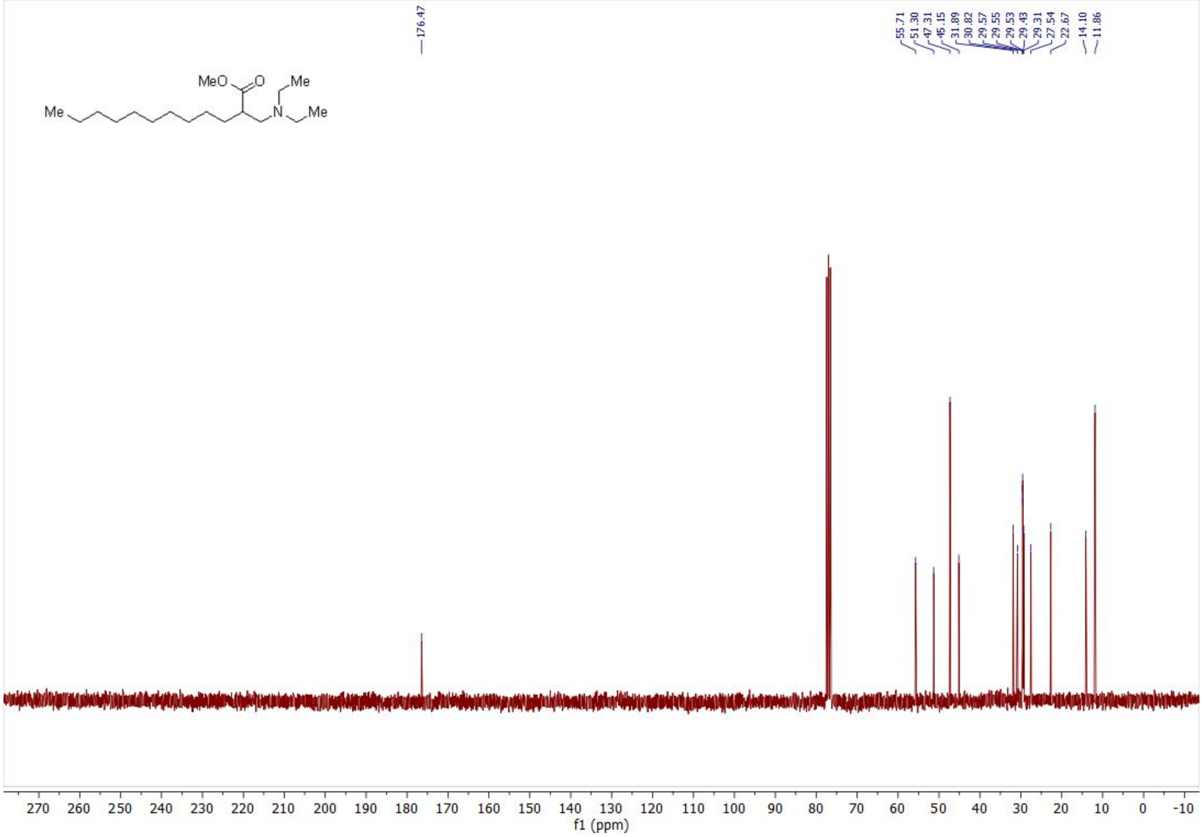

methyl 2-((4-((*S*)-(4-chlorophenyl)(pyridin-2-yl)methoxy)piperidin-1-yl)methyl)decanoate (3gg)

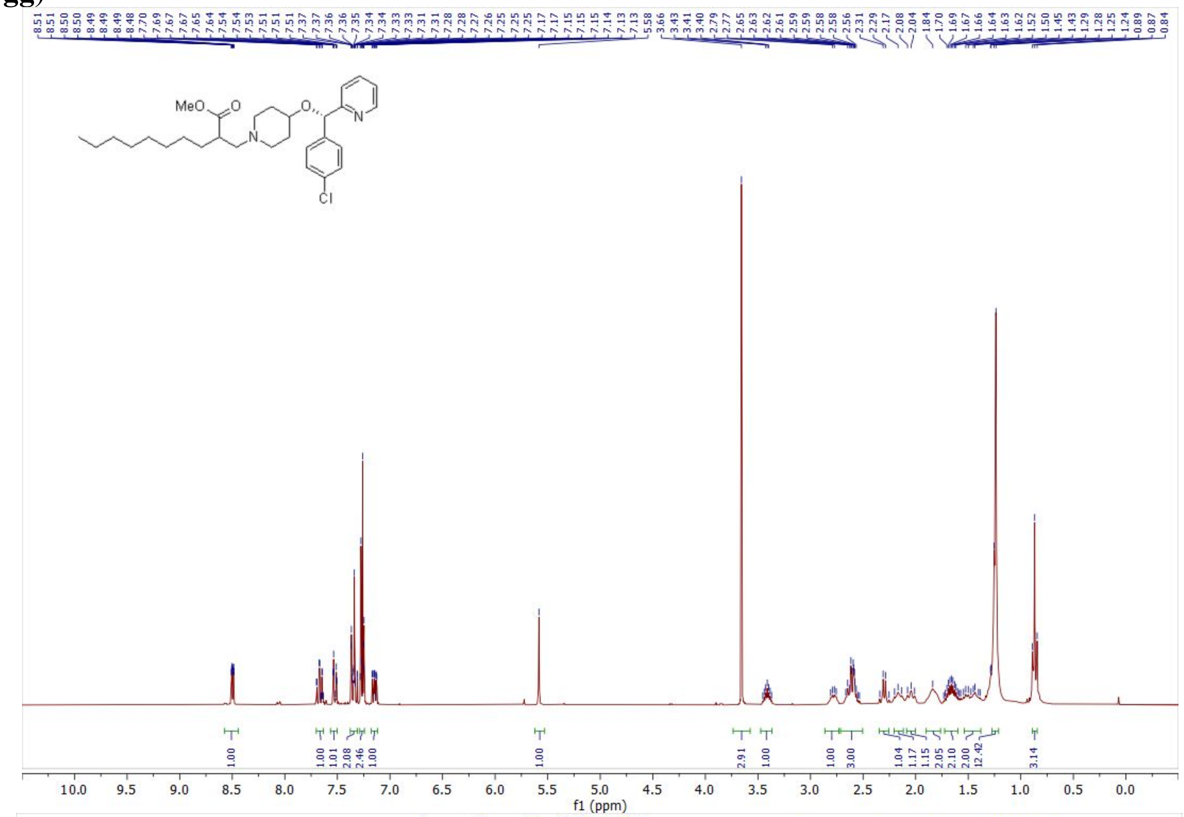

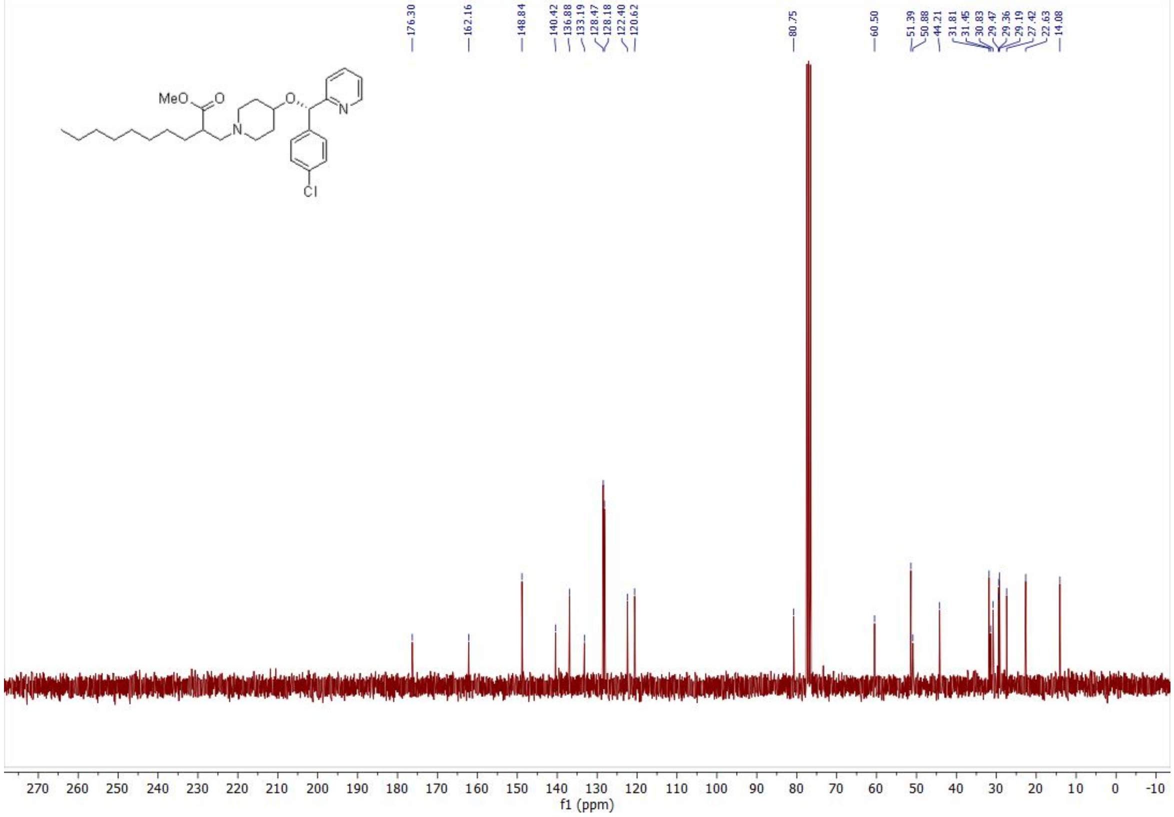

methyl 2-((((3s,5s,7s)-adamantan-1-yl)amino)methyl)decanoate (3hh)

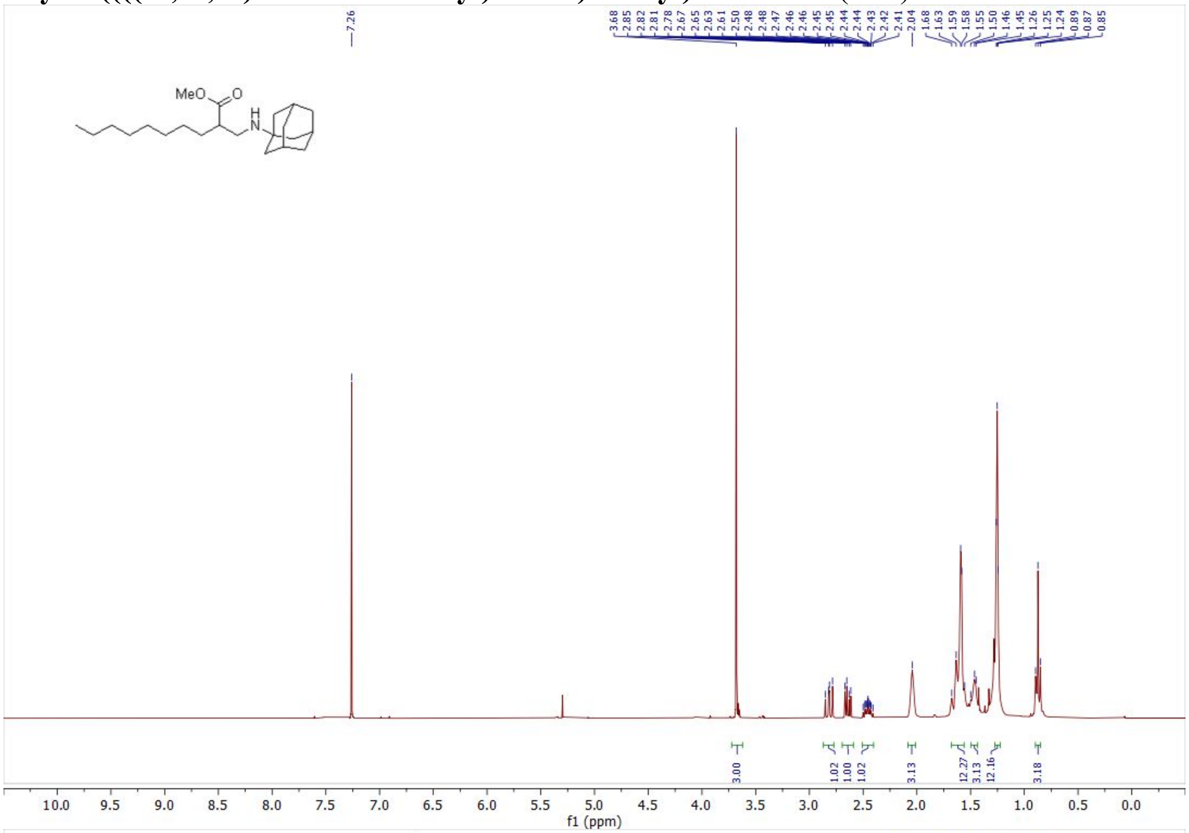

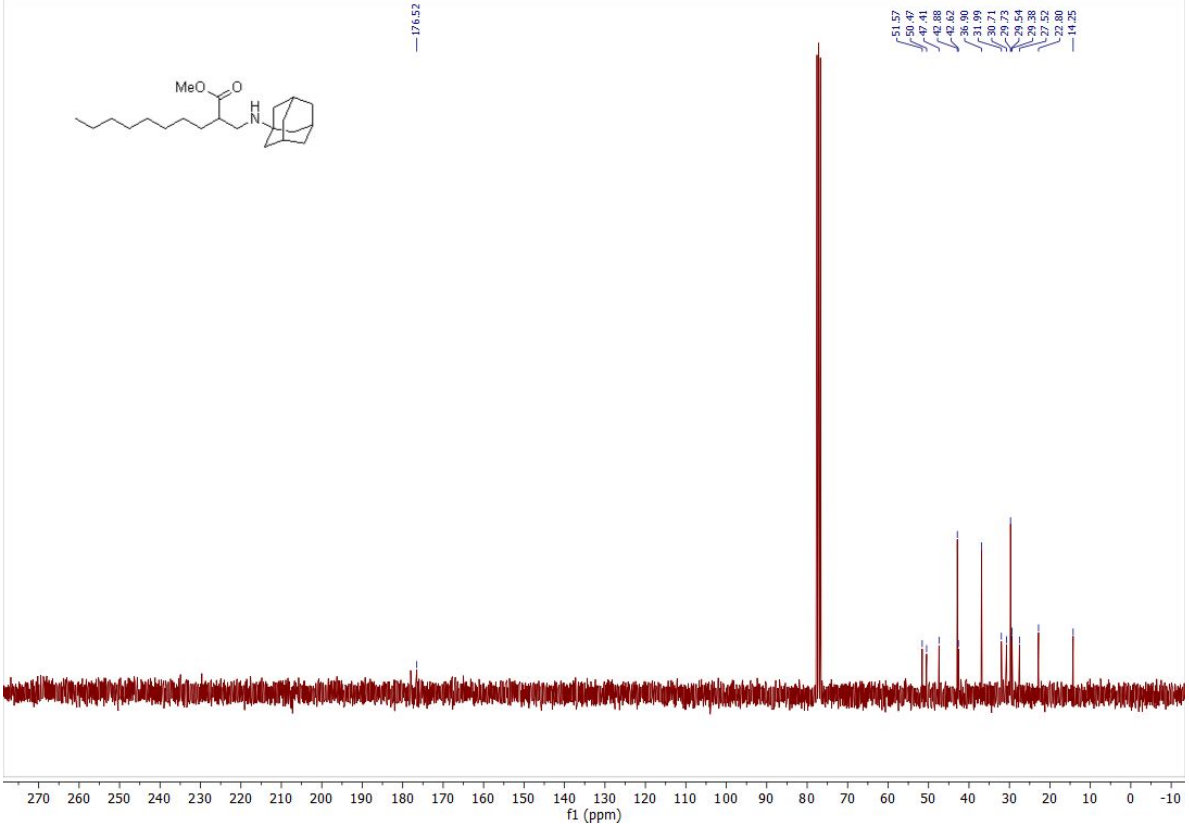

ethyl 2-(piperidin-1-ylmethyl)decanoate (3ii)

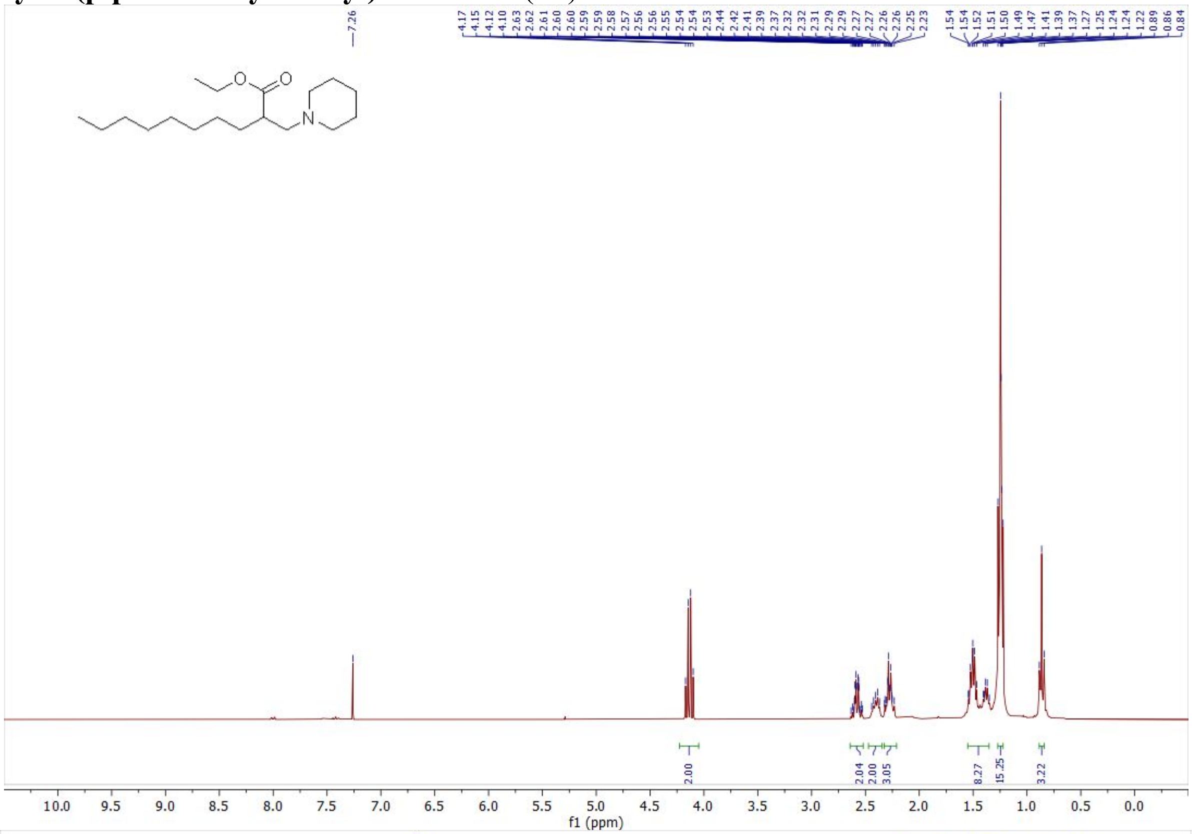

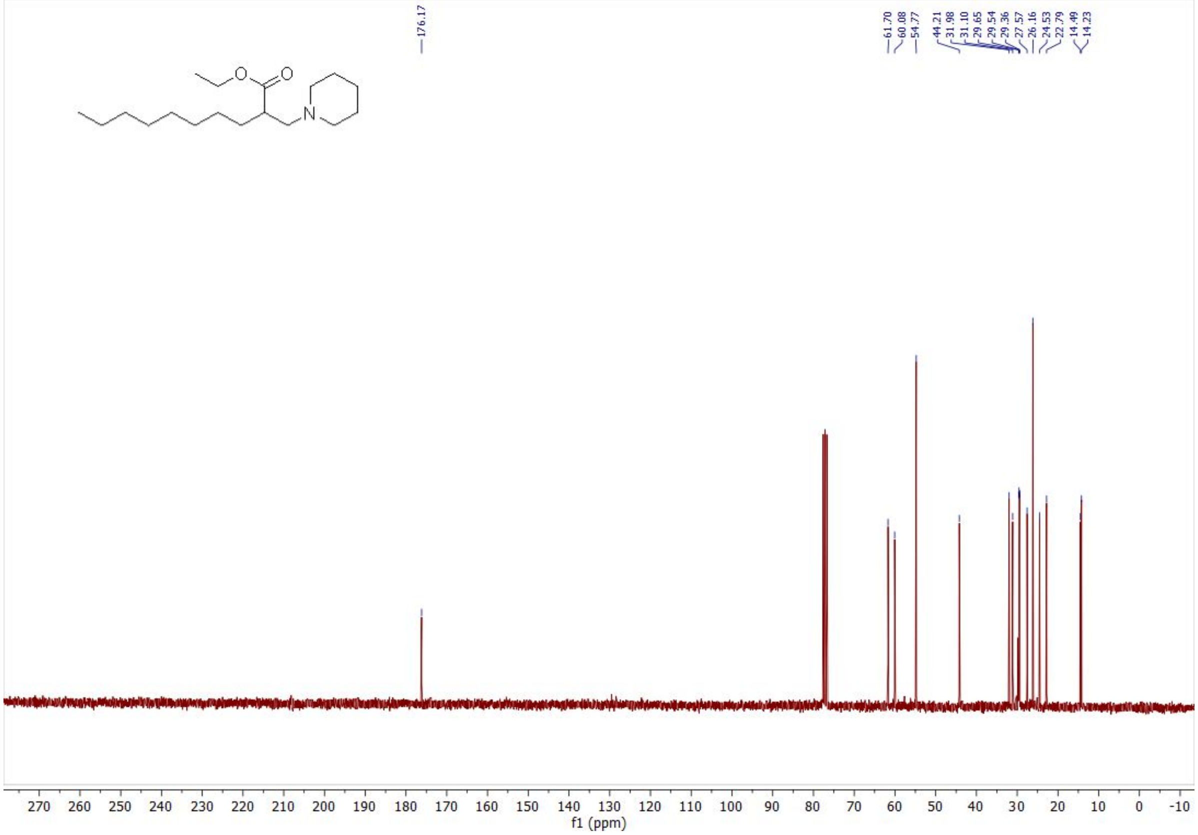

propyl 2-(piperidin-1-ylmethyl)decanoate (3jj)

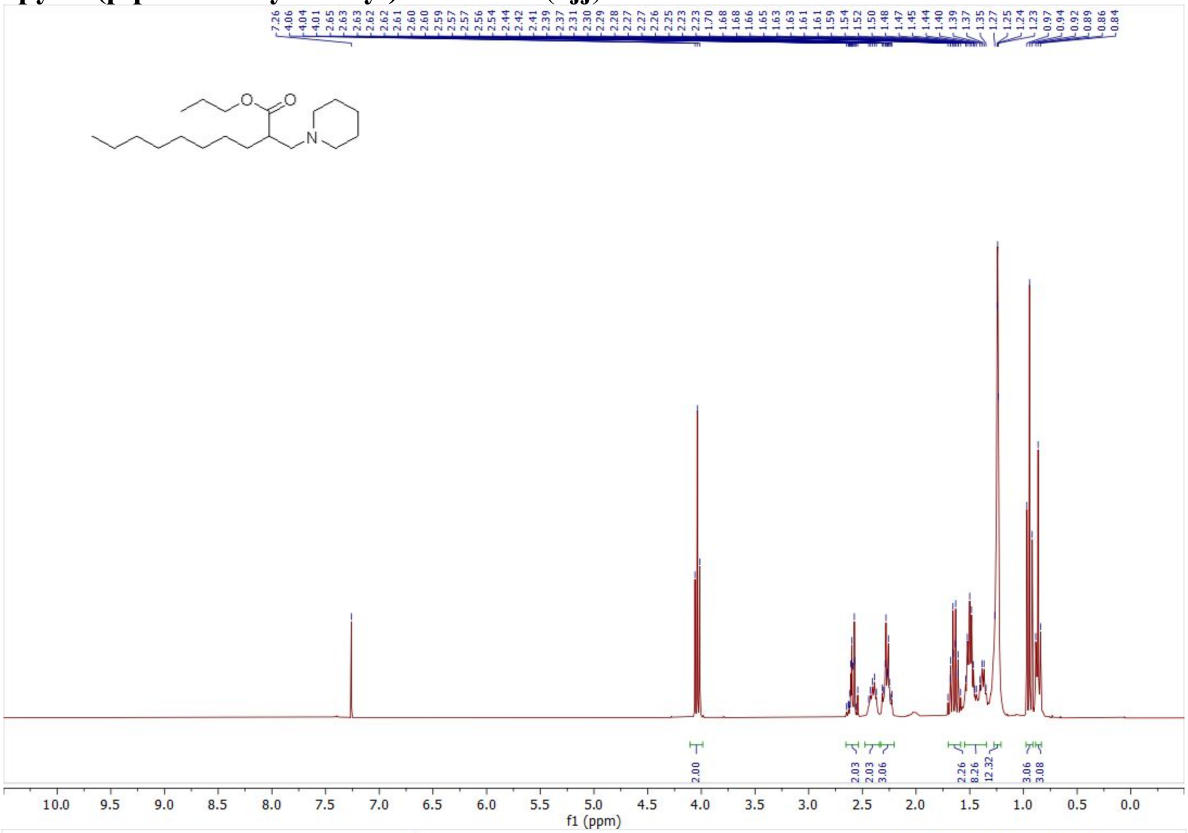

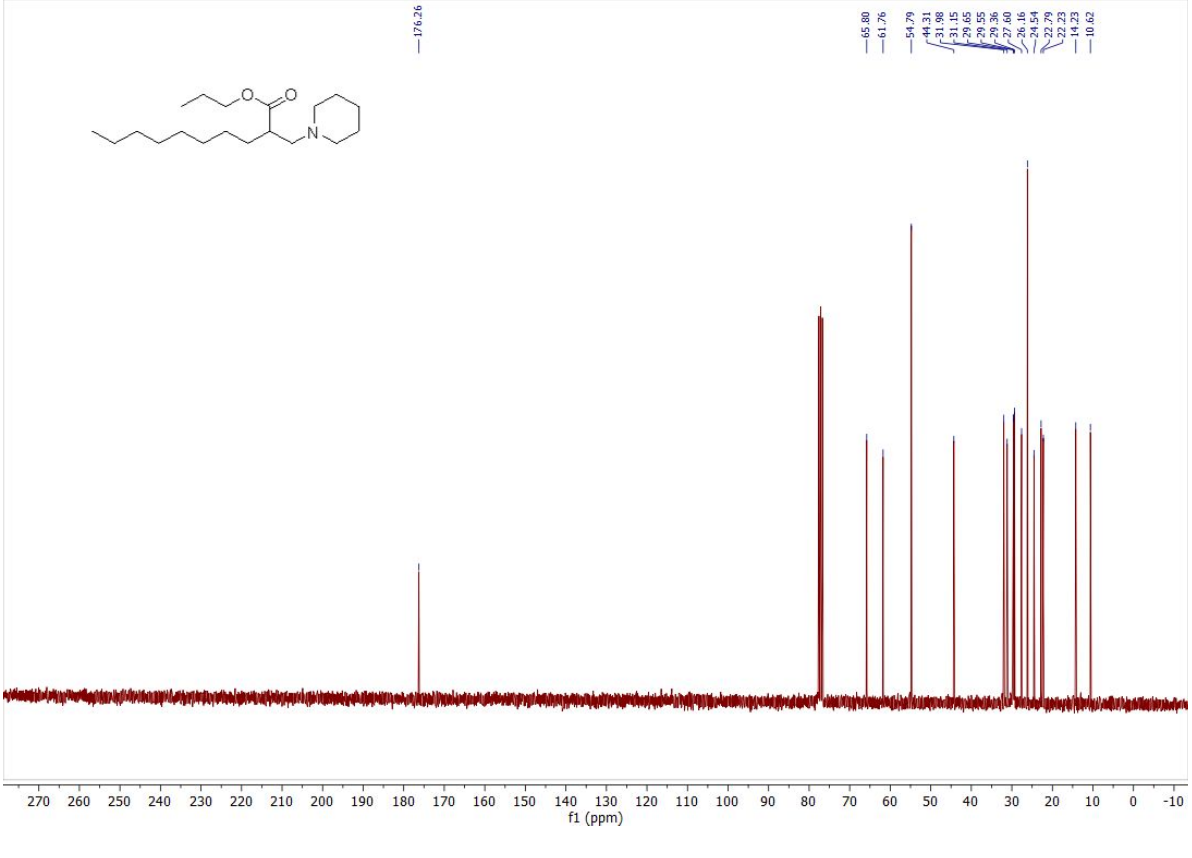

butyl 2-(piperidin-1-ylmethyl)decanoate (3kk)

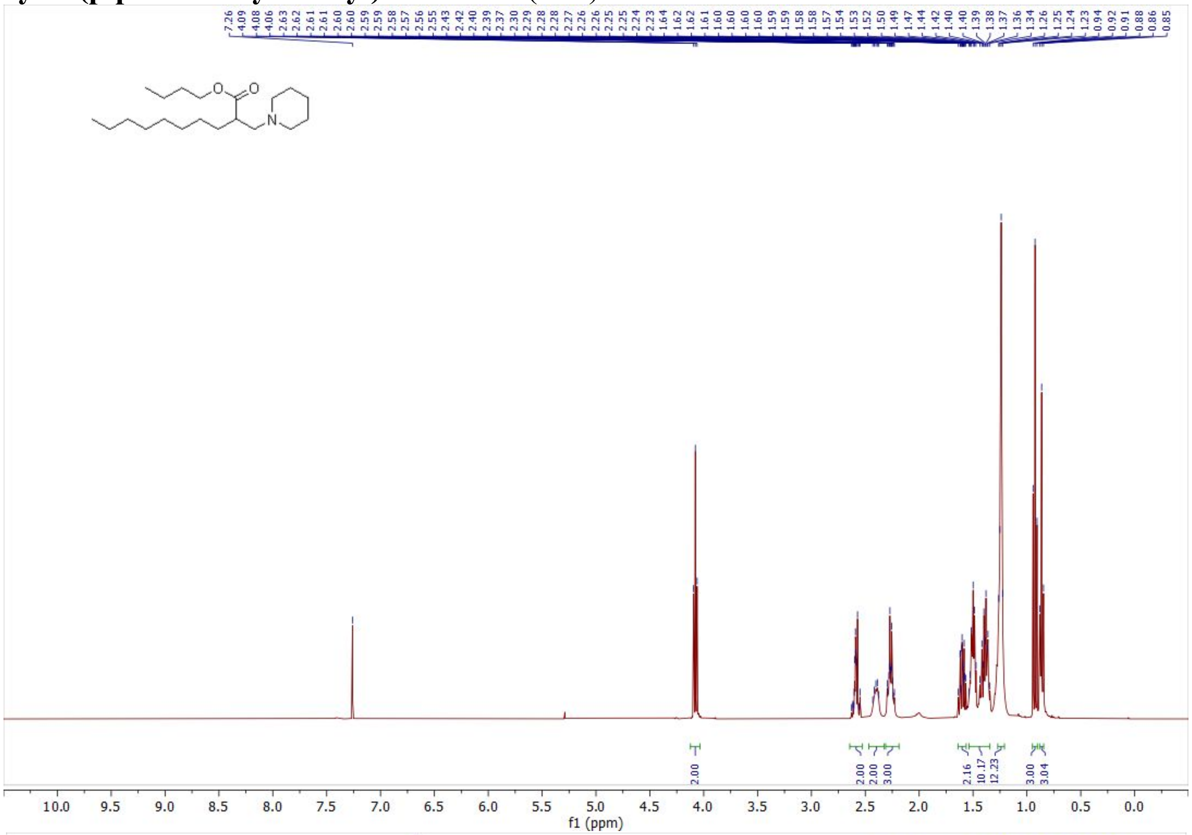

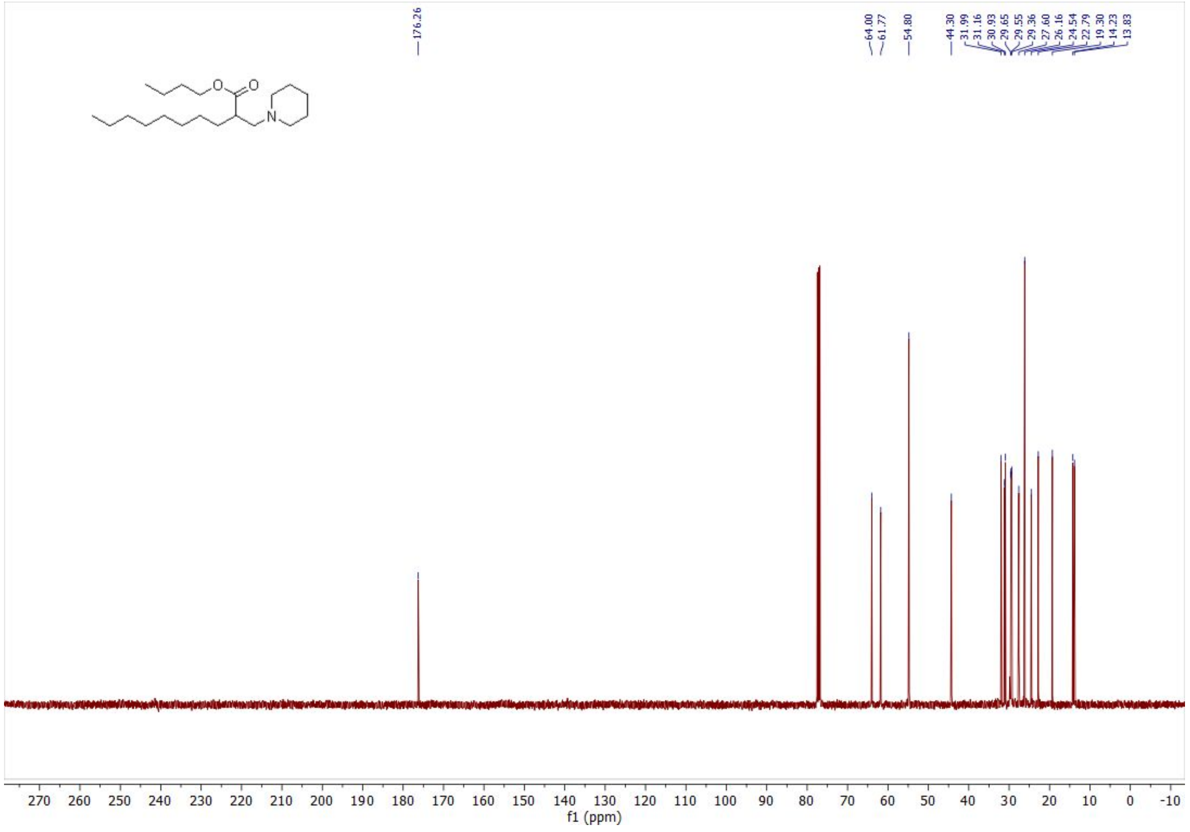

cyclohexylmethyl 2-(piperidin-1-ylmethyl)decanoate (3ll)

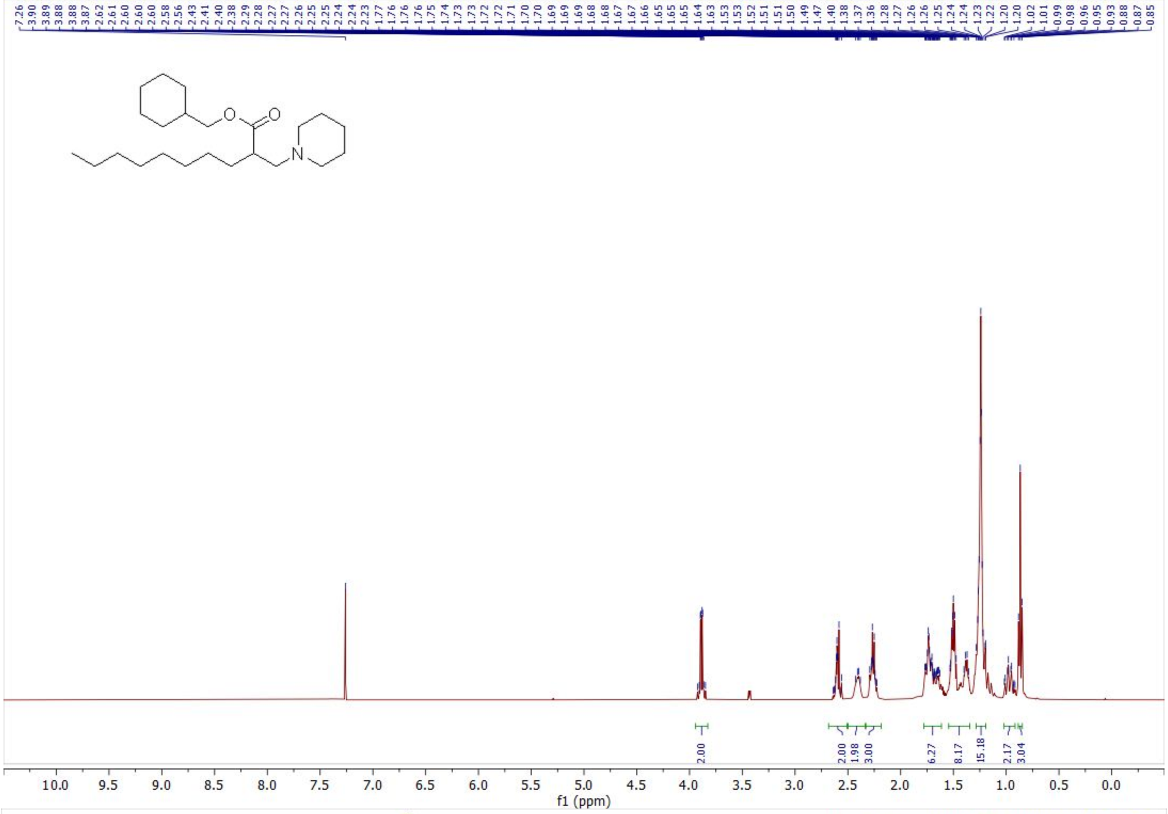

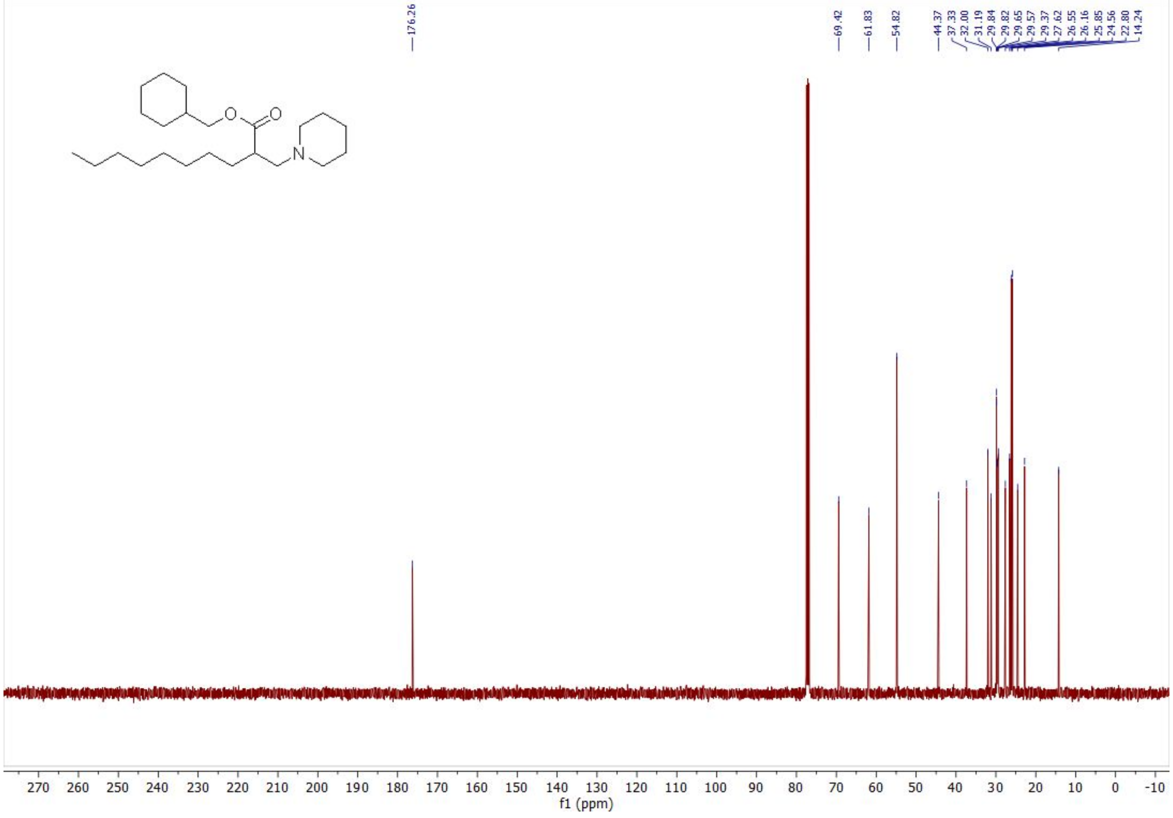

isopropyl 2-(piperidin-1-ylmethyl)decanoate (3mm)

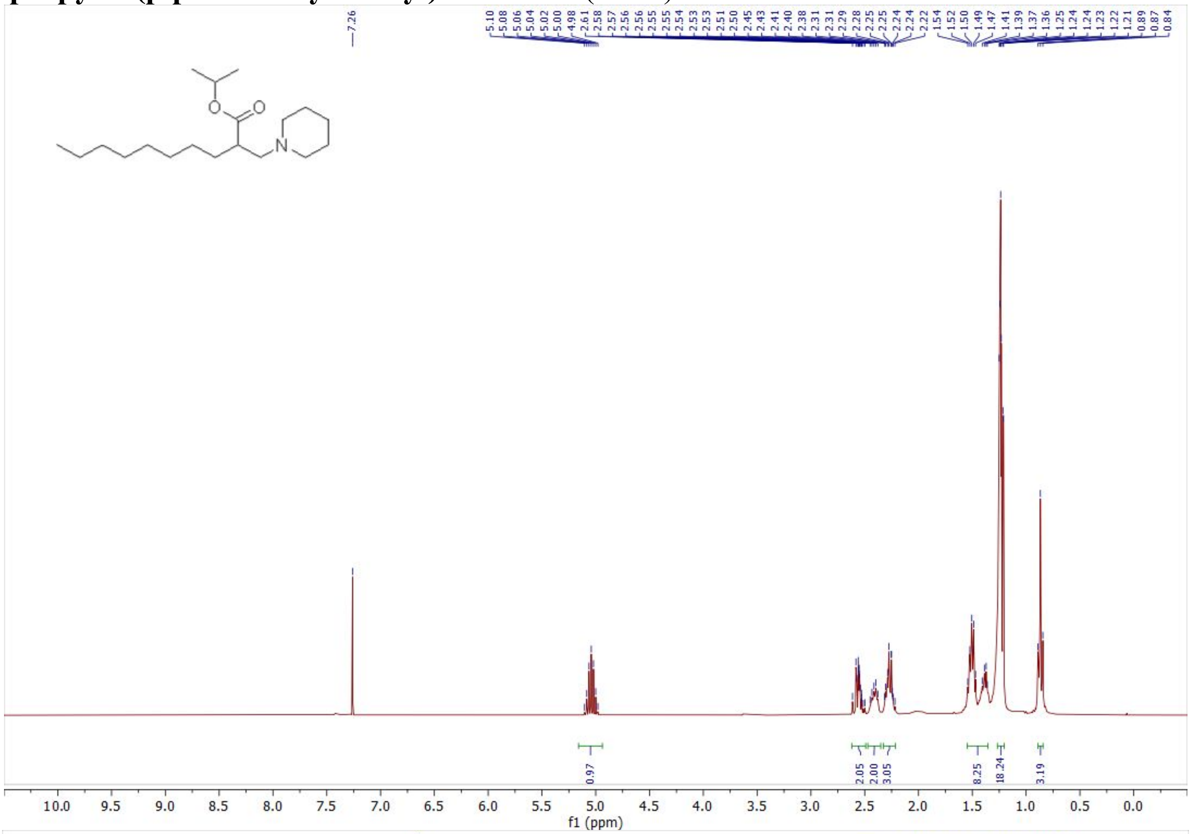

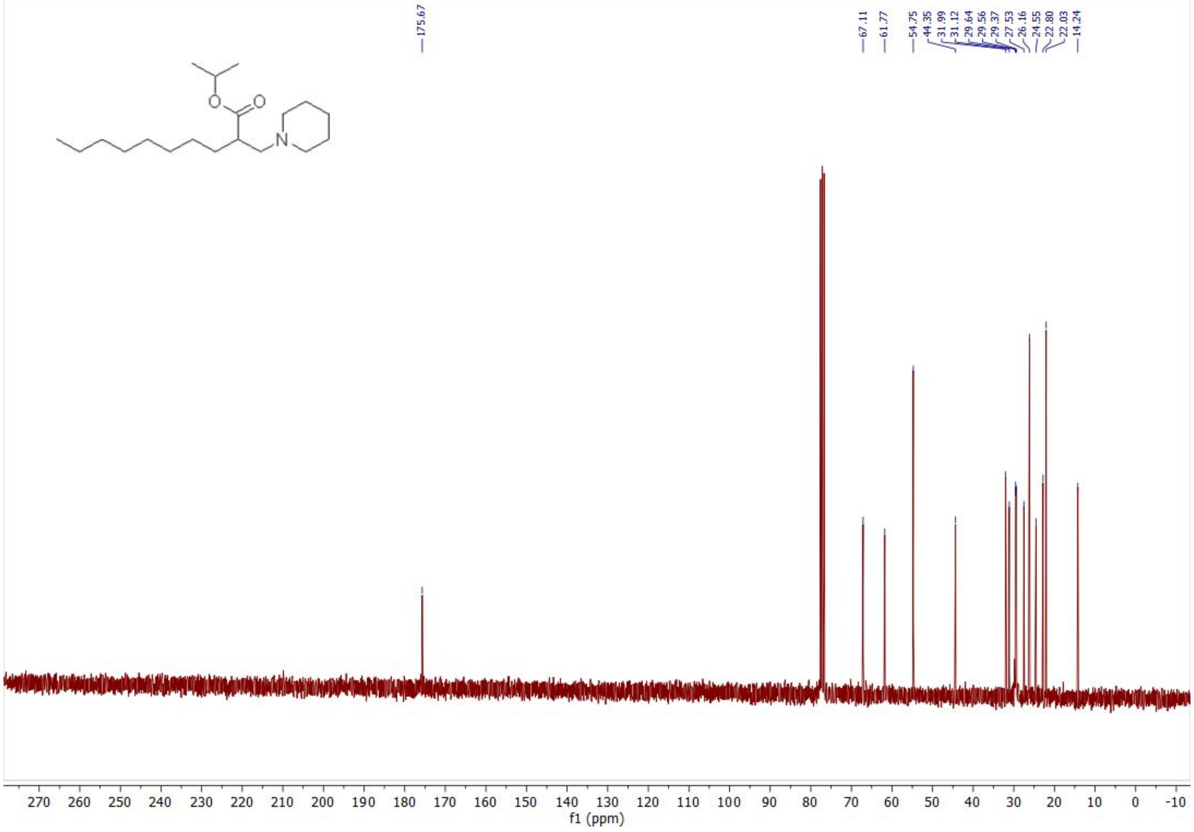

2,2,2-trifluoroethyl 2-(piperidin-1-ylmethyl)decanoate (3nn)

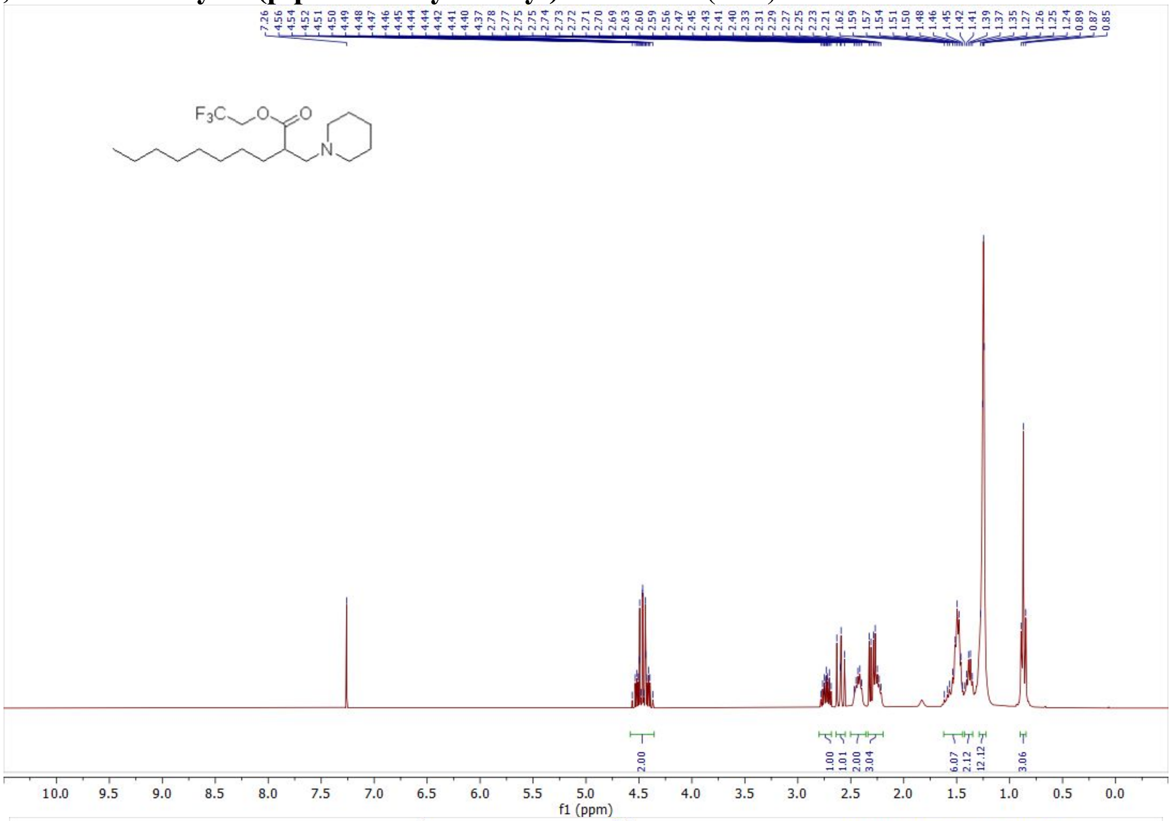

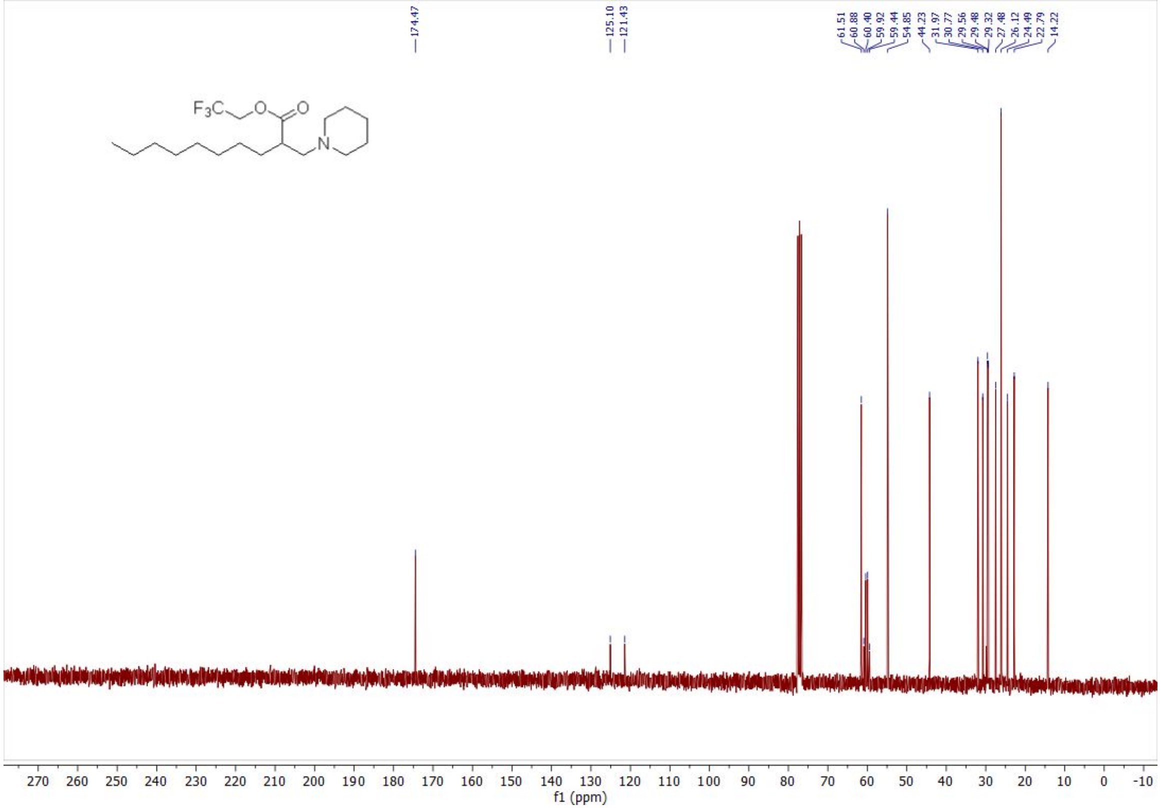

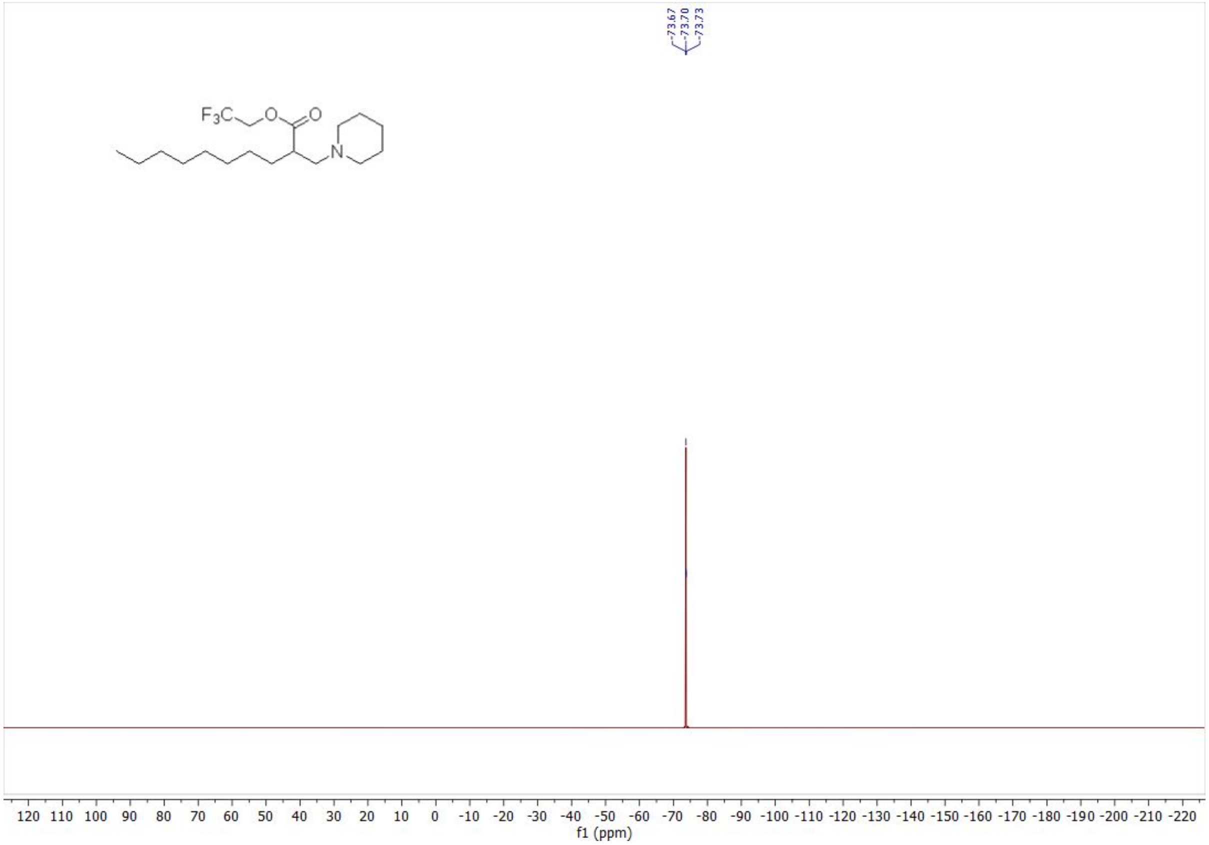

-73.67
-73.70
-73.73
F_3C
O
O
N
120 110 100 90 80 70 60 50 40 30 20 10 0 -10 -20 -30 -40 -50 -60 -70 -80 -90 -100 -110 -120 -130 -140 -150 -160 -170 -180 -190 -200 -210 -220
f1 (ppm)

11-((1*R*,2*S*,5*R*)-2-isopropyl-5-methylcyclohexyl) 1-methyl 2-(piperidin-1-ylmethyl)undecanedioate (3oo)

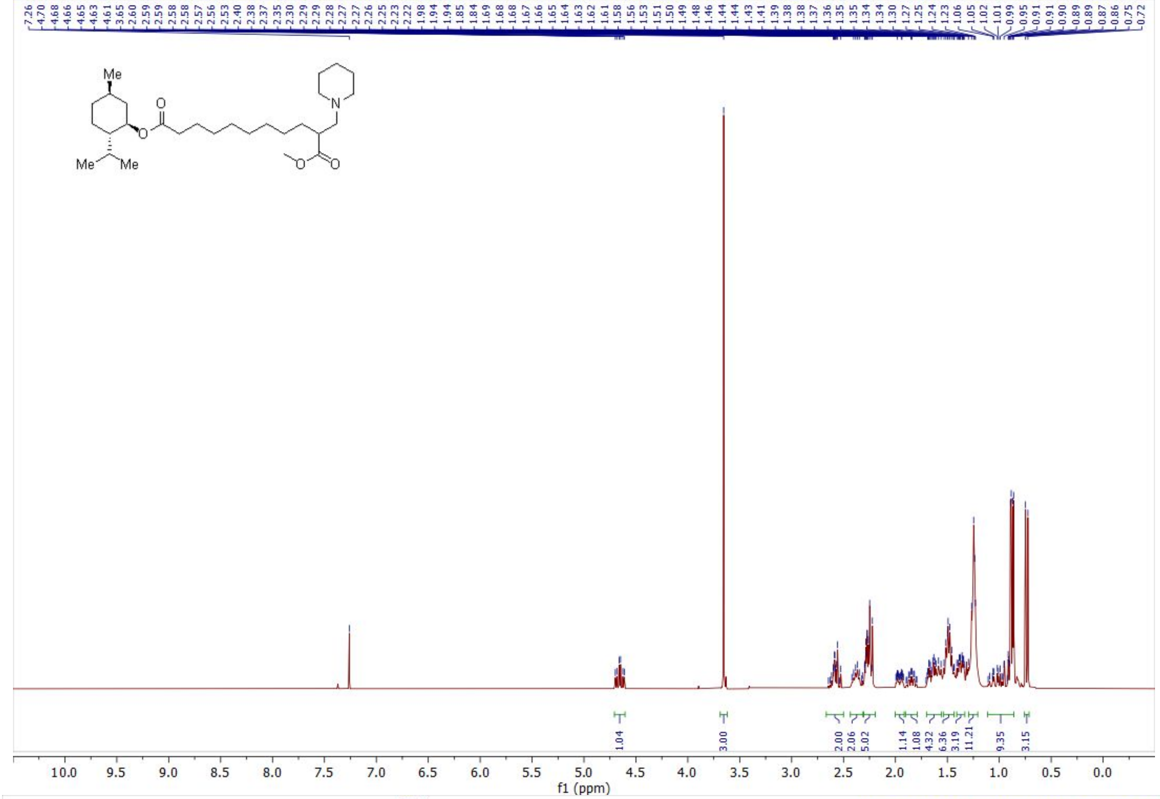

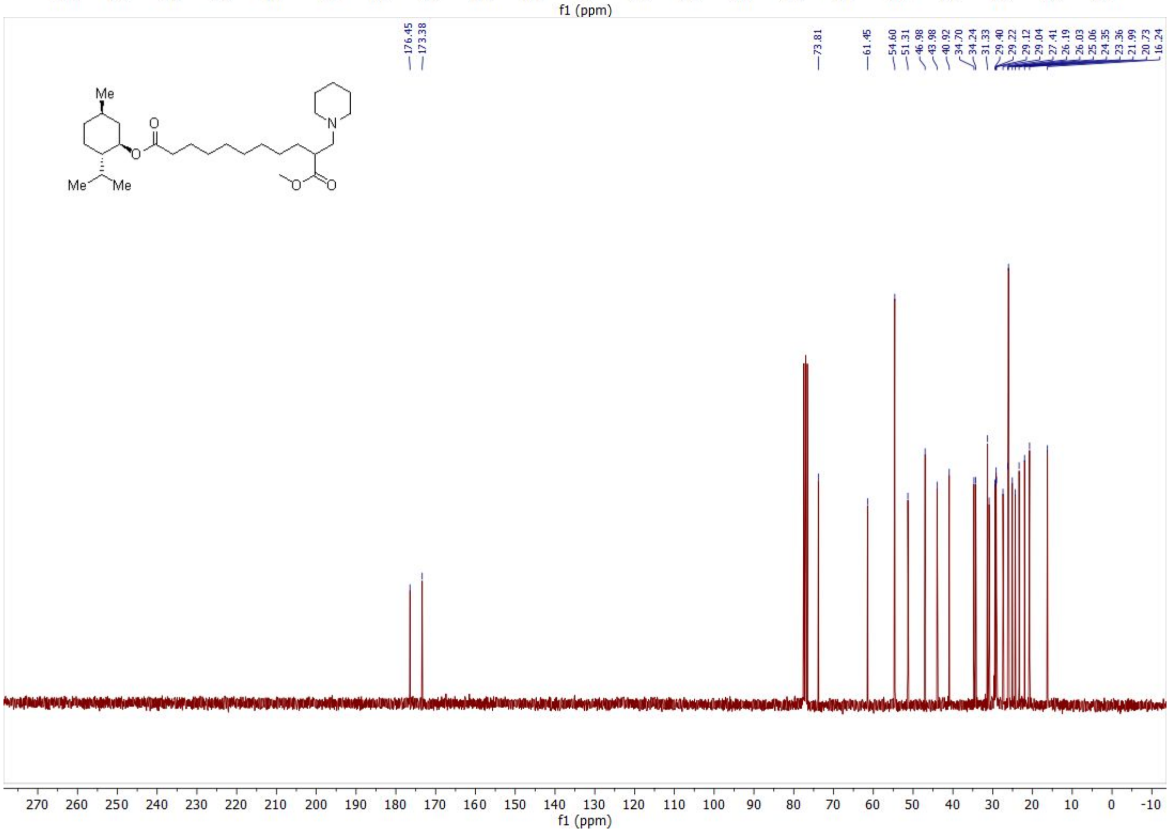

11-(3,7-dimethyloct-6-en-1-yl) 1-methyl 2-(piperidin-1-ylmethyl)undecanedioate (3pp)

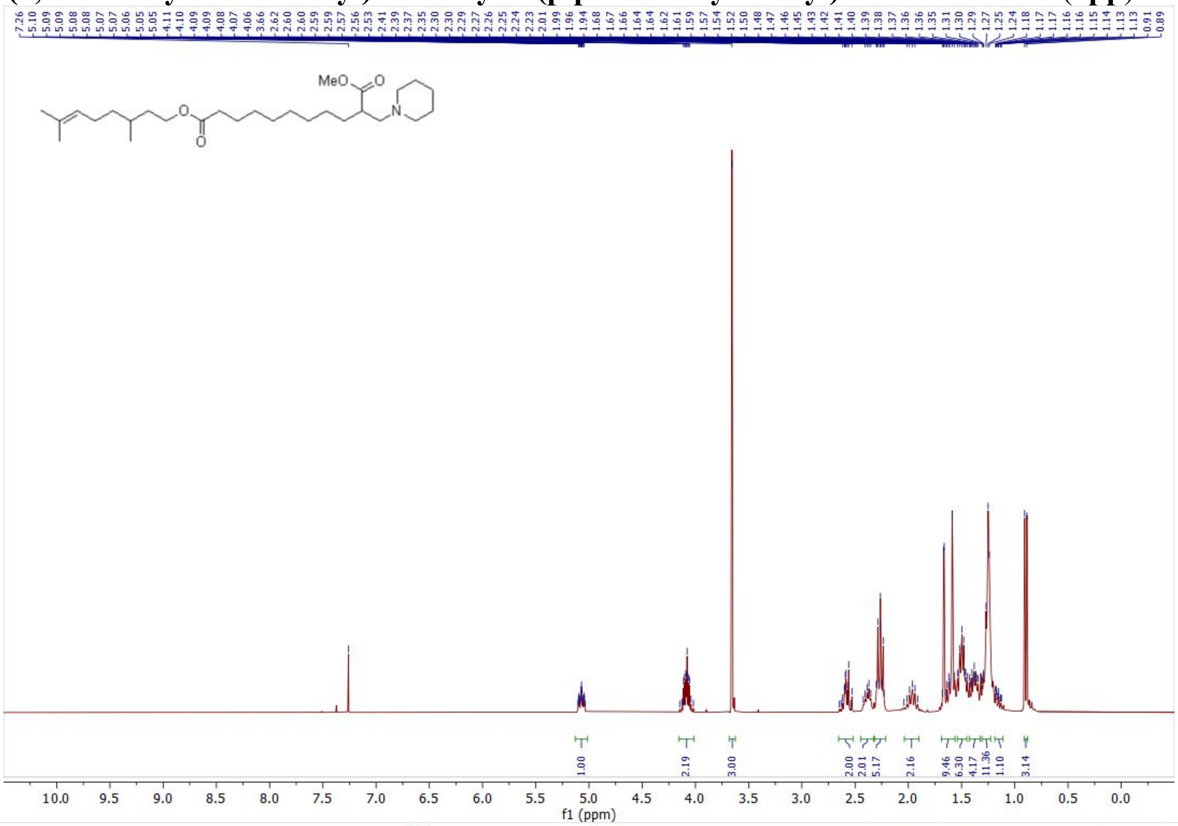

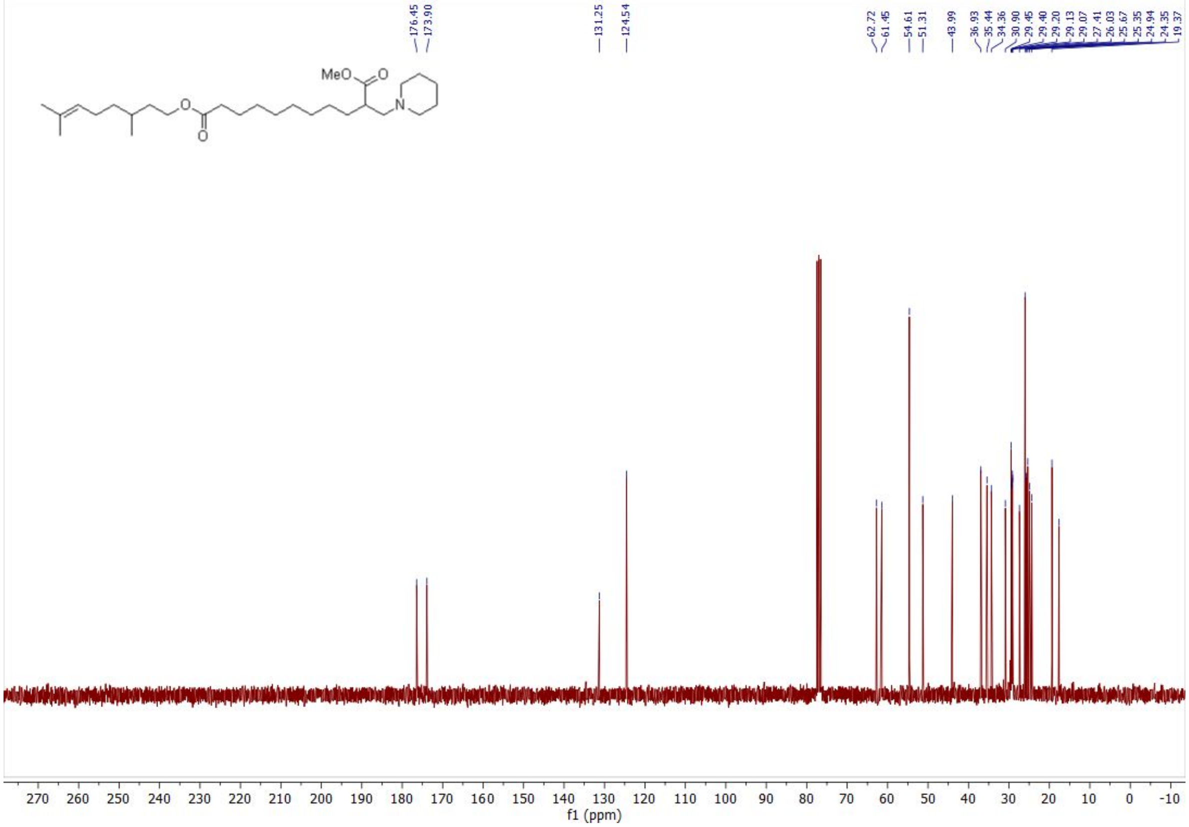

methyl 6-((2-(4-isobutylphenyl)propanoyl)oxy)-2-(piperidin-1-ylmethyl)hexanoate (3qq)

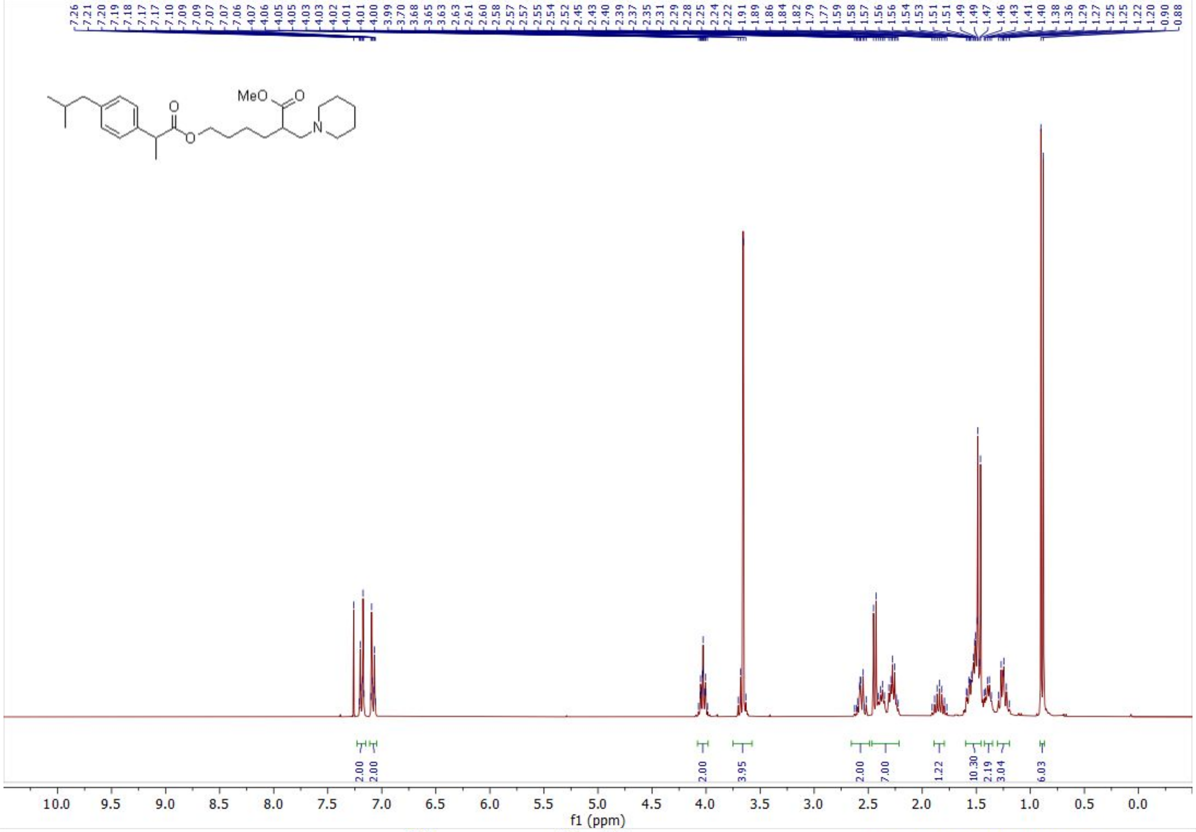

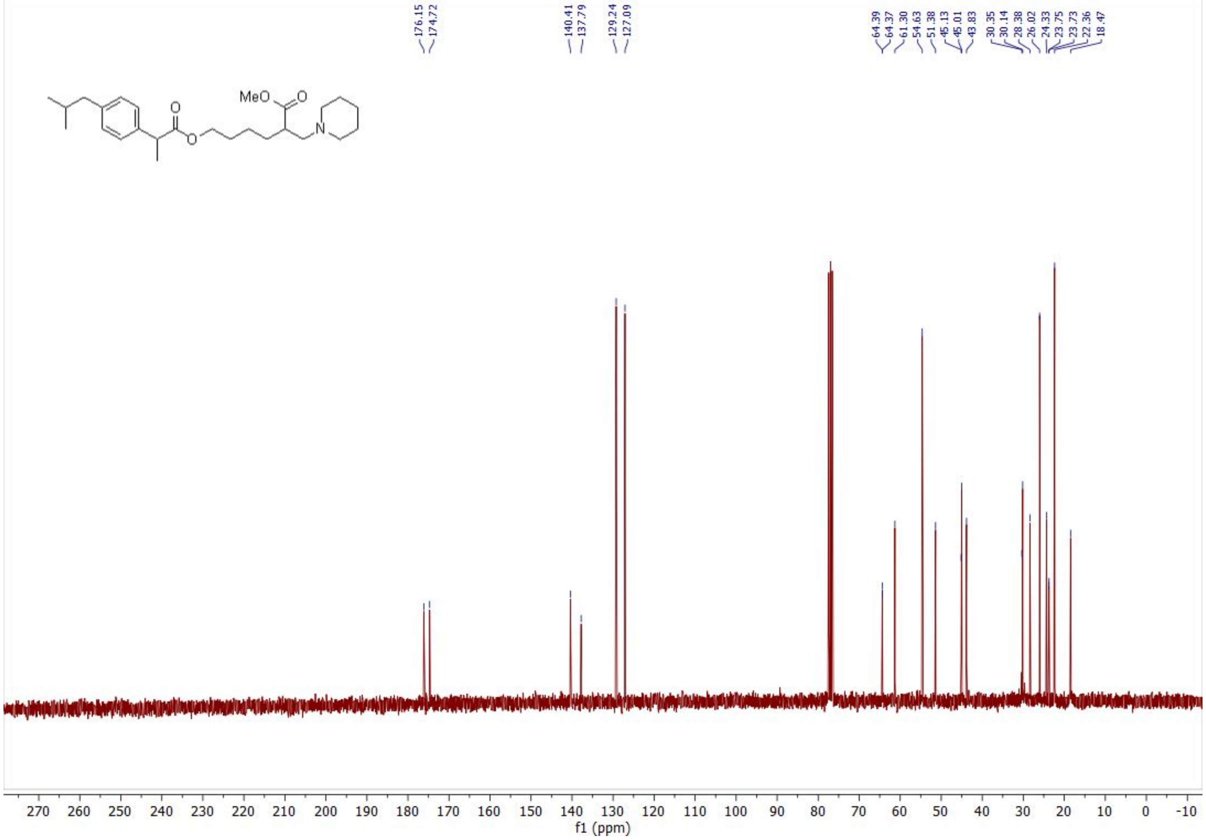

11-((3a*R*,5*R*,6*S*,6a*R*)-5-((*R*)-2,2-dimethyl-1,3-dioxolan-4-yl)-2,2-dimethyltetrahydrofuro[2,3-*d*][1,3]dioxol-6-yl) 1-methyl 2-(piperidin-1-ylmethyl)undecanedioate (3rr)

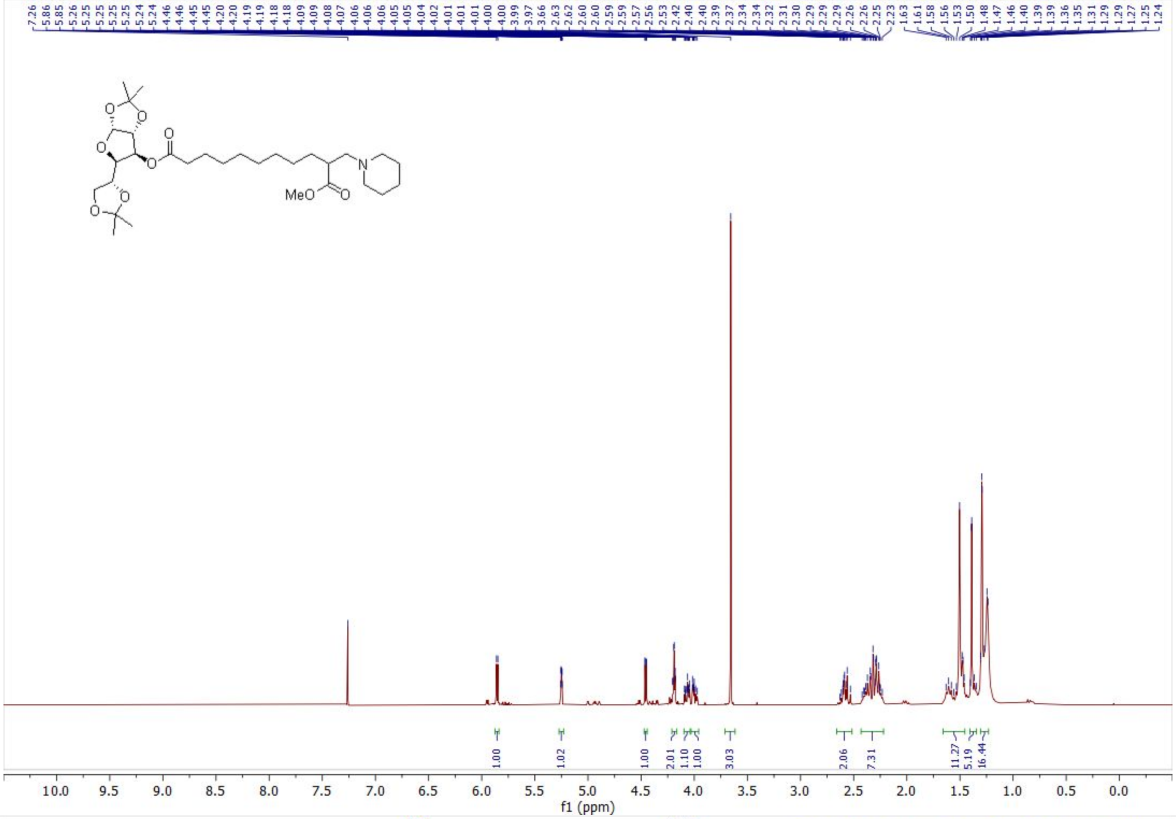

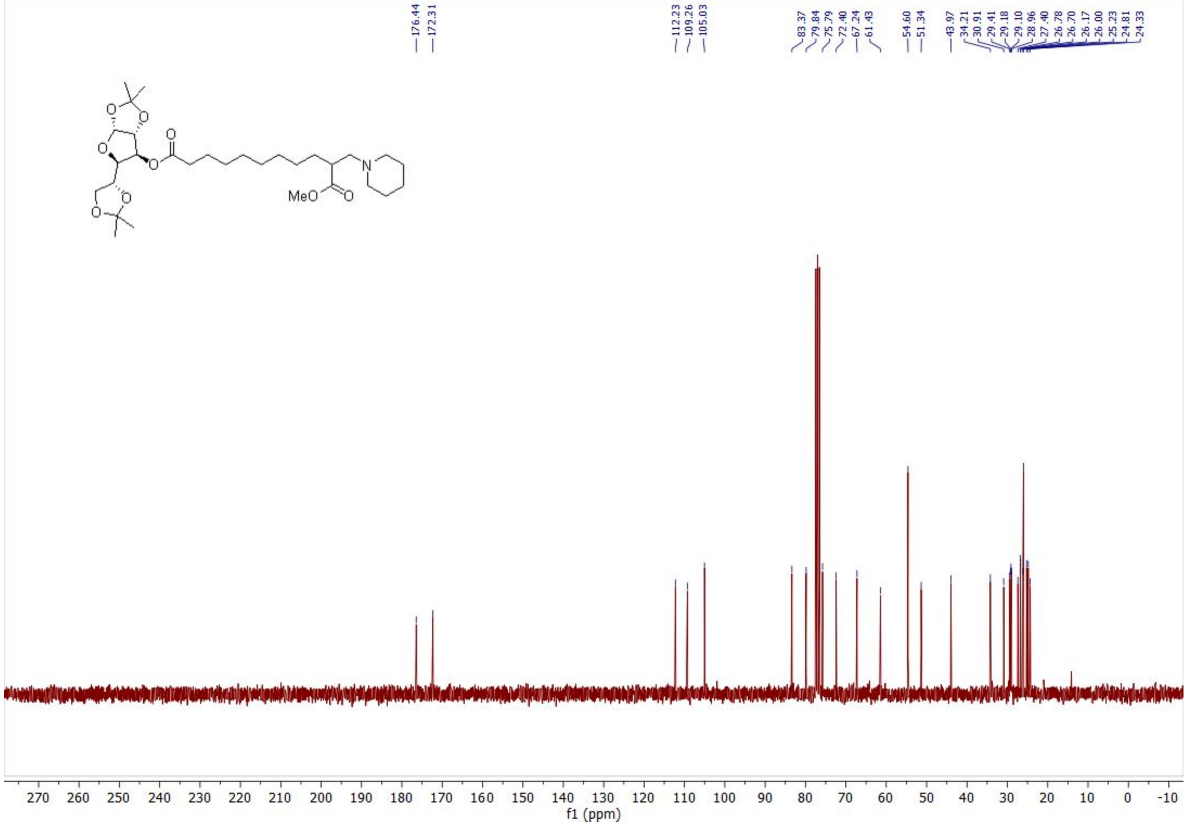

methyl 6-(2-(11-oxo-6,11-dihydrodibenzo[*b*,*e*]oxepin-2-yl)acetoxy)-2-(piperidin-1-ylmethyl)hexanoate (3ss)

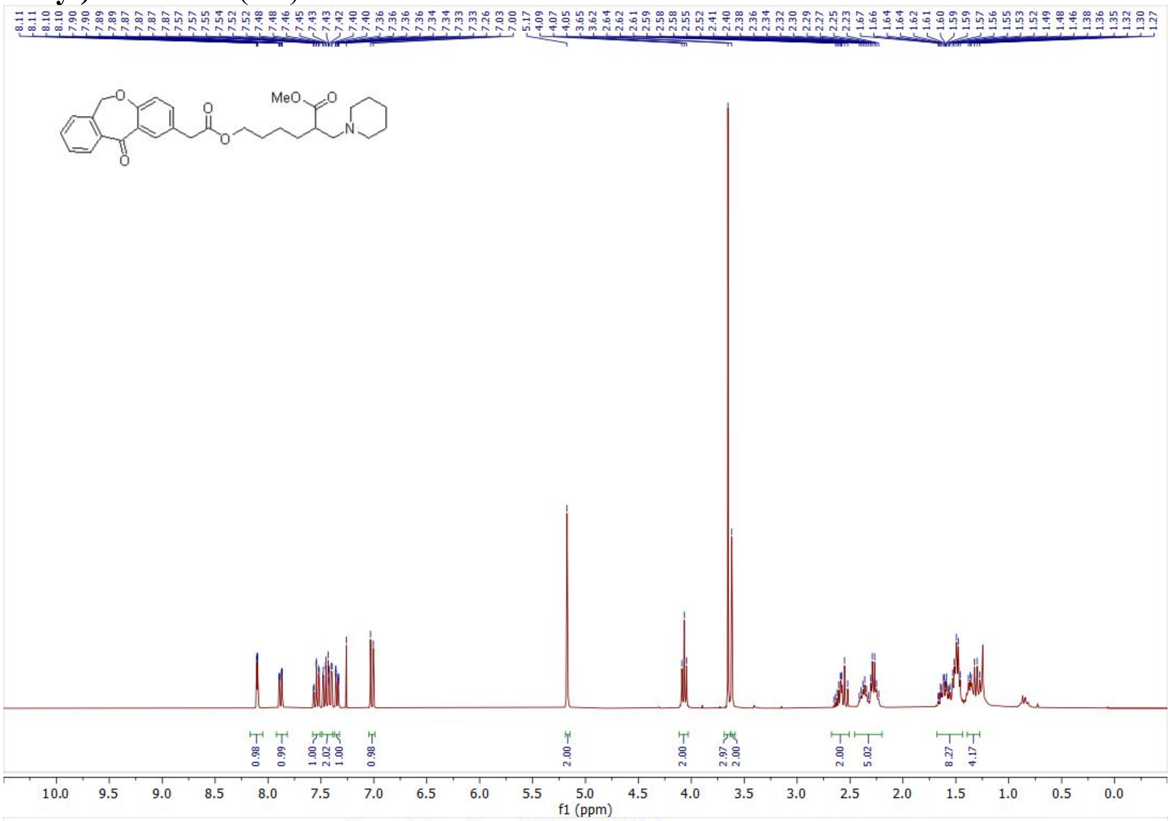

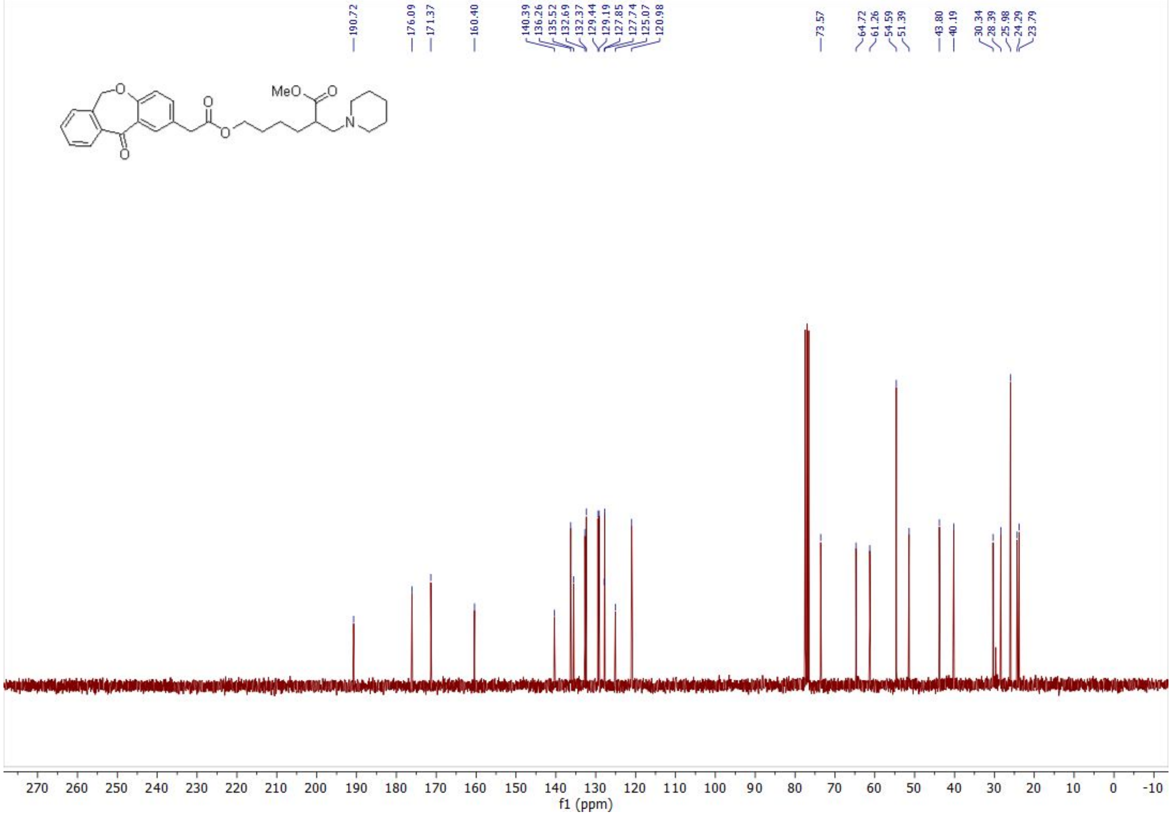

methyl 6-((3-(4,5-diphenyloxazol-2-yl)propanoyl)oxy)-2-(piperidin-1-ylmethyl)hexanoate (3tt)

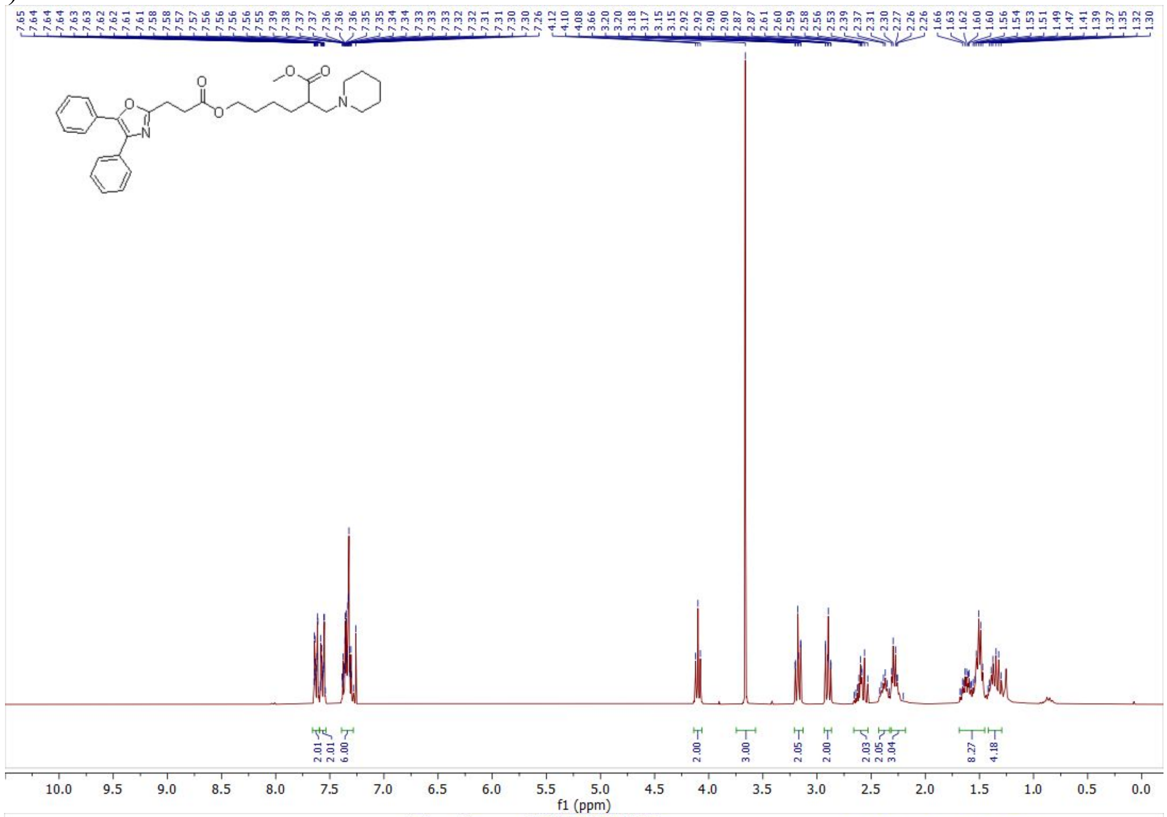

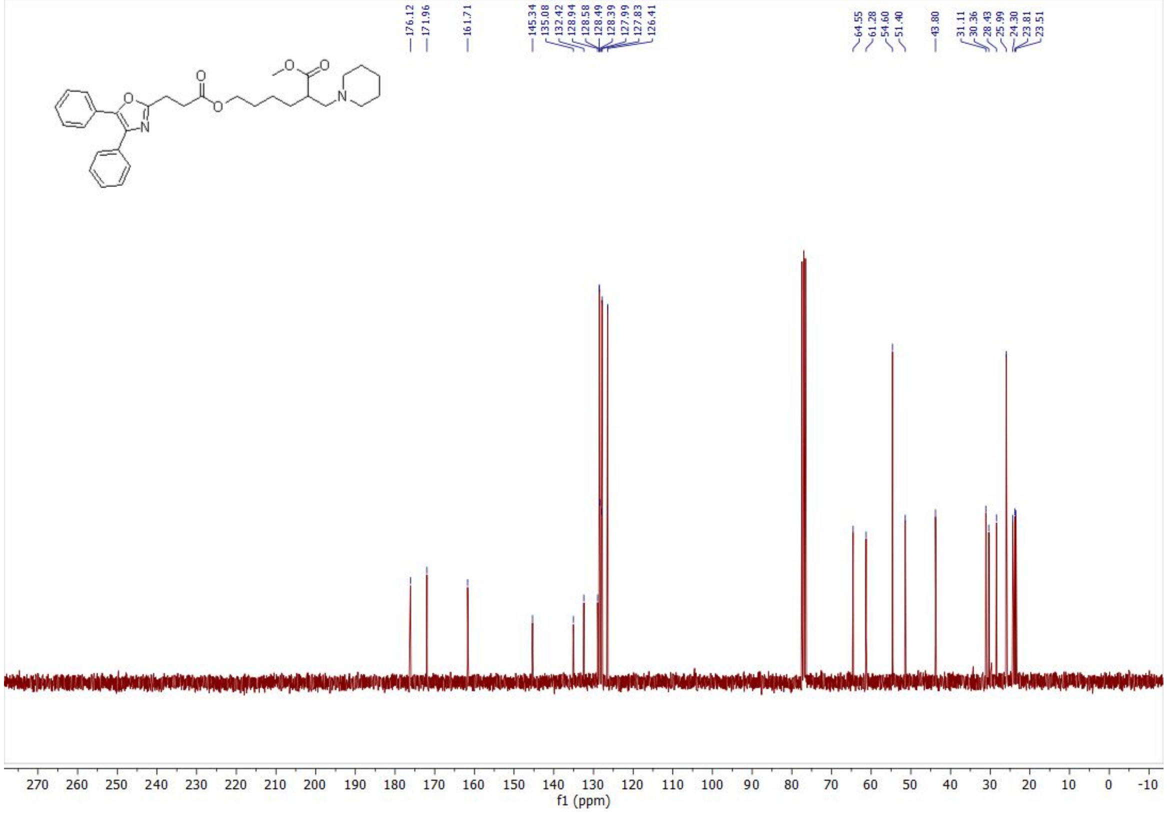

11-((3*S*,8*S*,9*S*,10*R*,13*R*,14*S*,17*R*)-10,13-dimethyl-17-((*R*)-6-methylheptan-2-yl)-2,3,4,7,8,9,10,11,12,13,14,15,16,17-tetradecahydro-1*H*-cyclopenta[a]phenanthren-3-yl) 1-methyl 2-(piperidin-1-ylmethyl)undecanedioate (3uu)

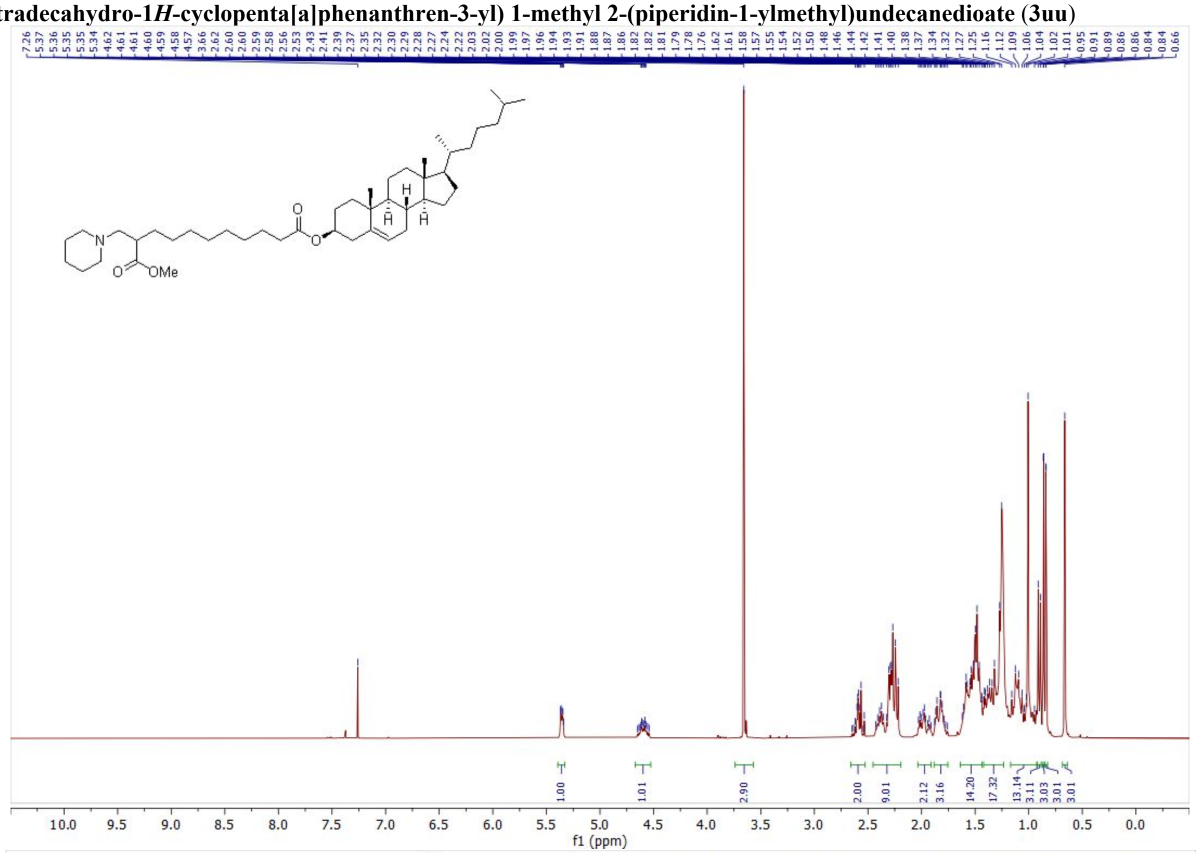

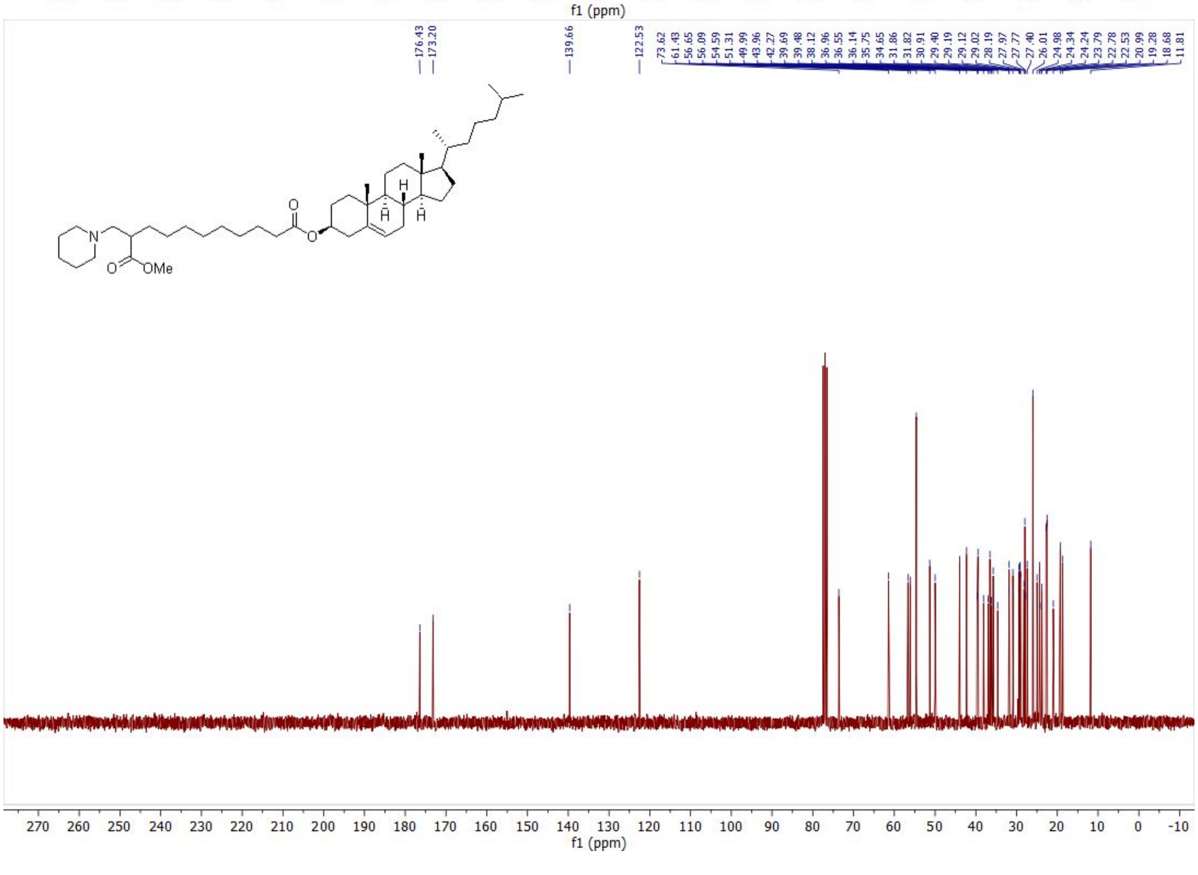

methyl 6-(((*tert*-butoxycarbonyl)-*L*-leucyl)oxy)-2-(piperidin-1-ylmethyl)hexanoate (3vv)

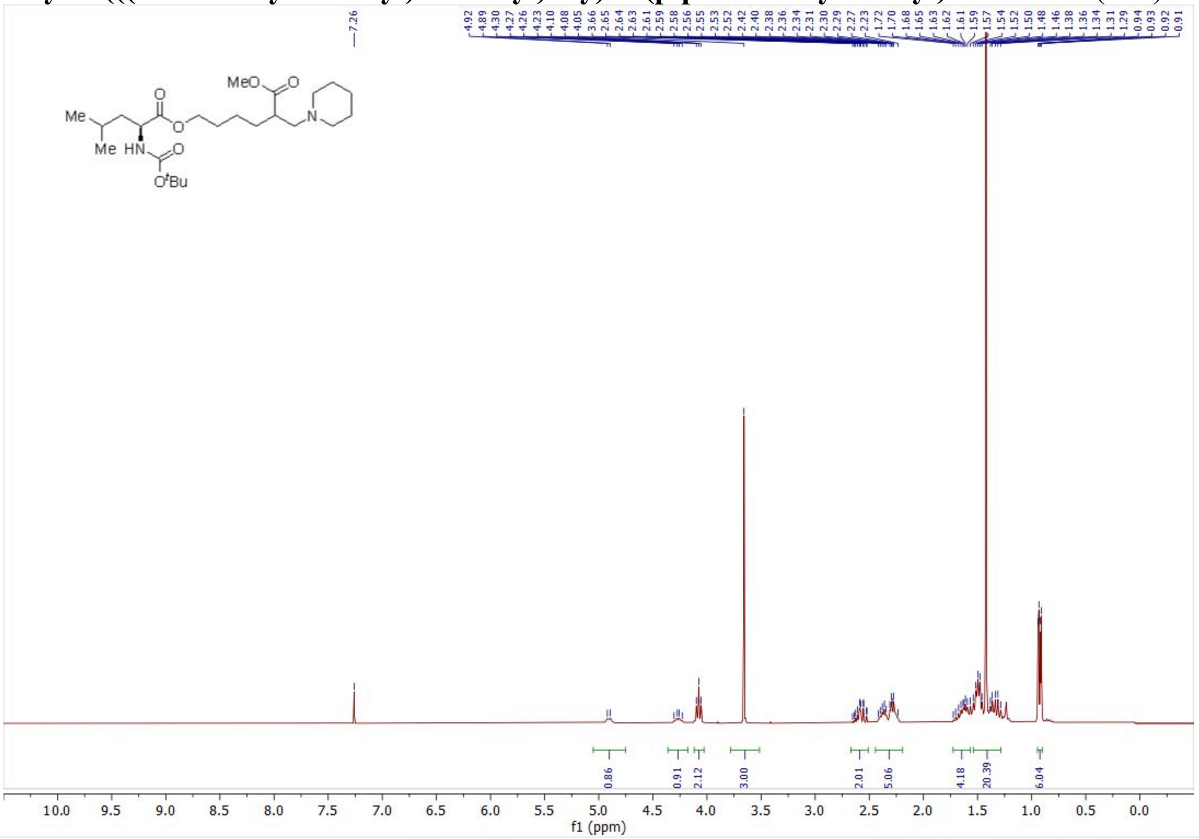

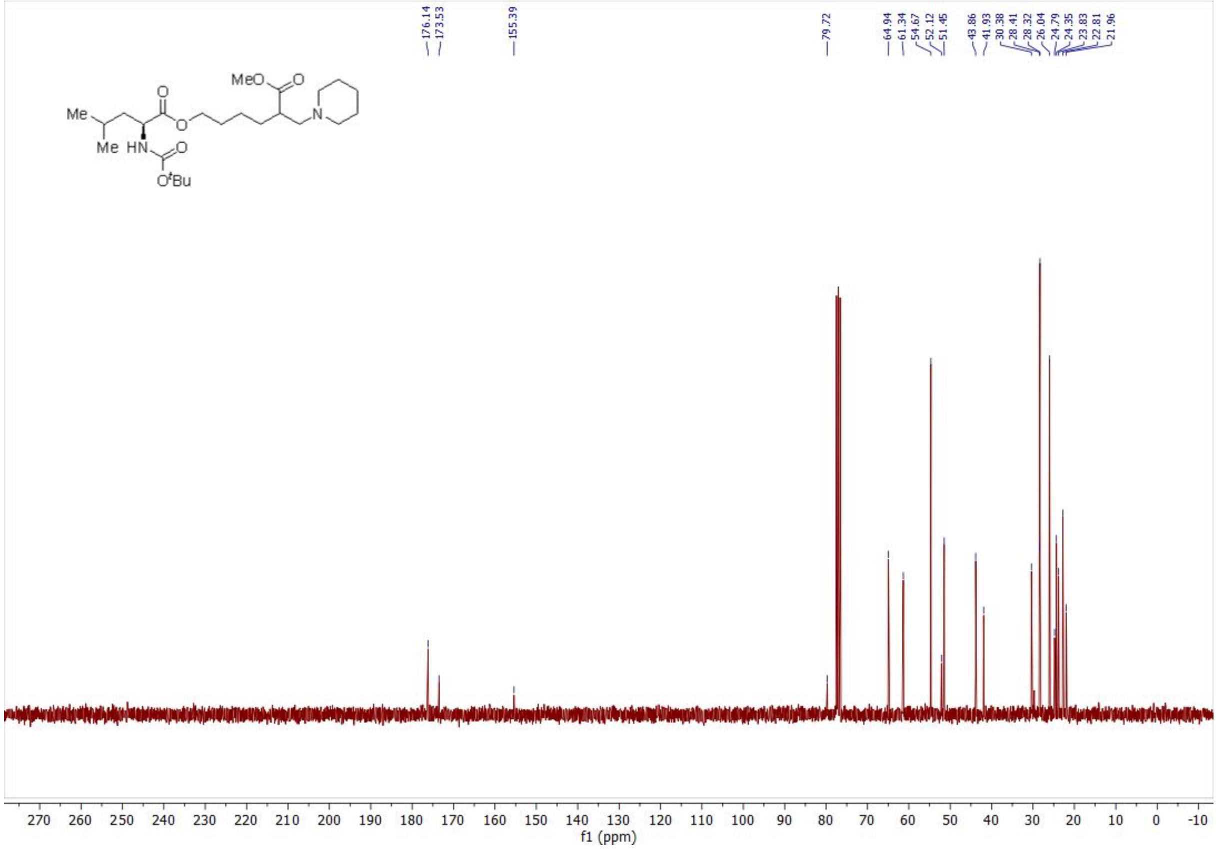

2-morpholinocyclopentyl benzoate (8a)

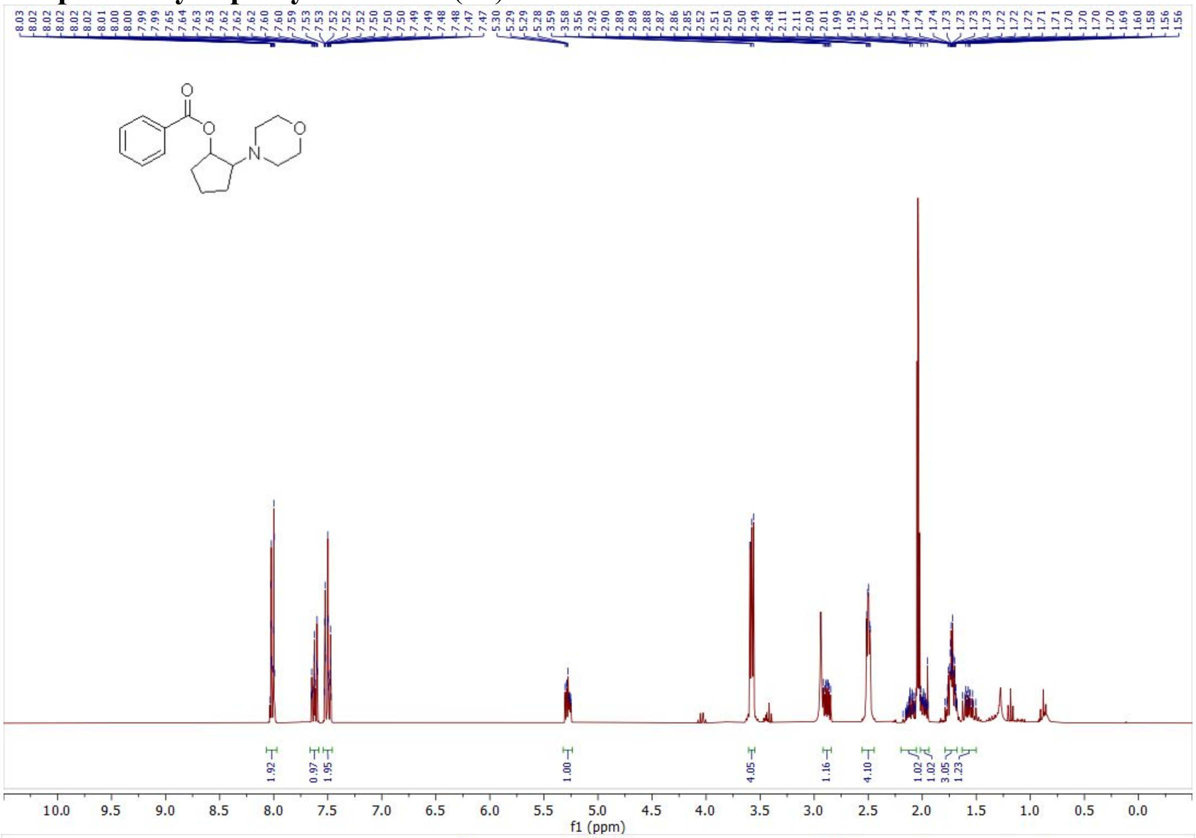

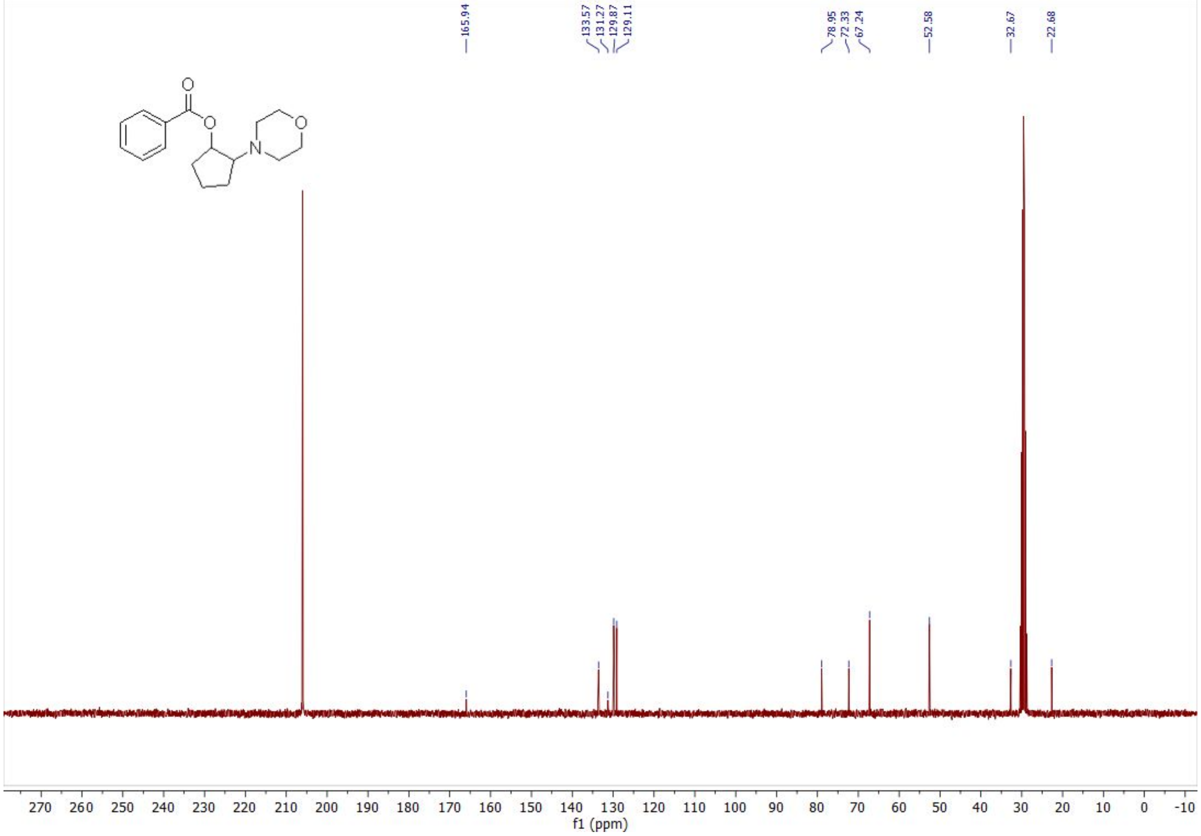

2-(2,6-dimethylmorpholino)cyclopentyl benzoate (8b)

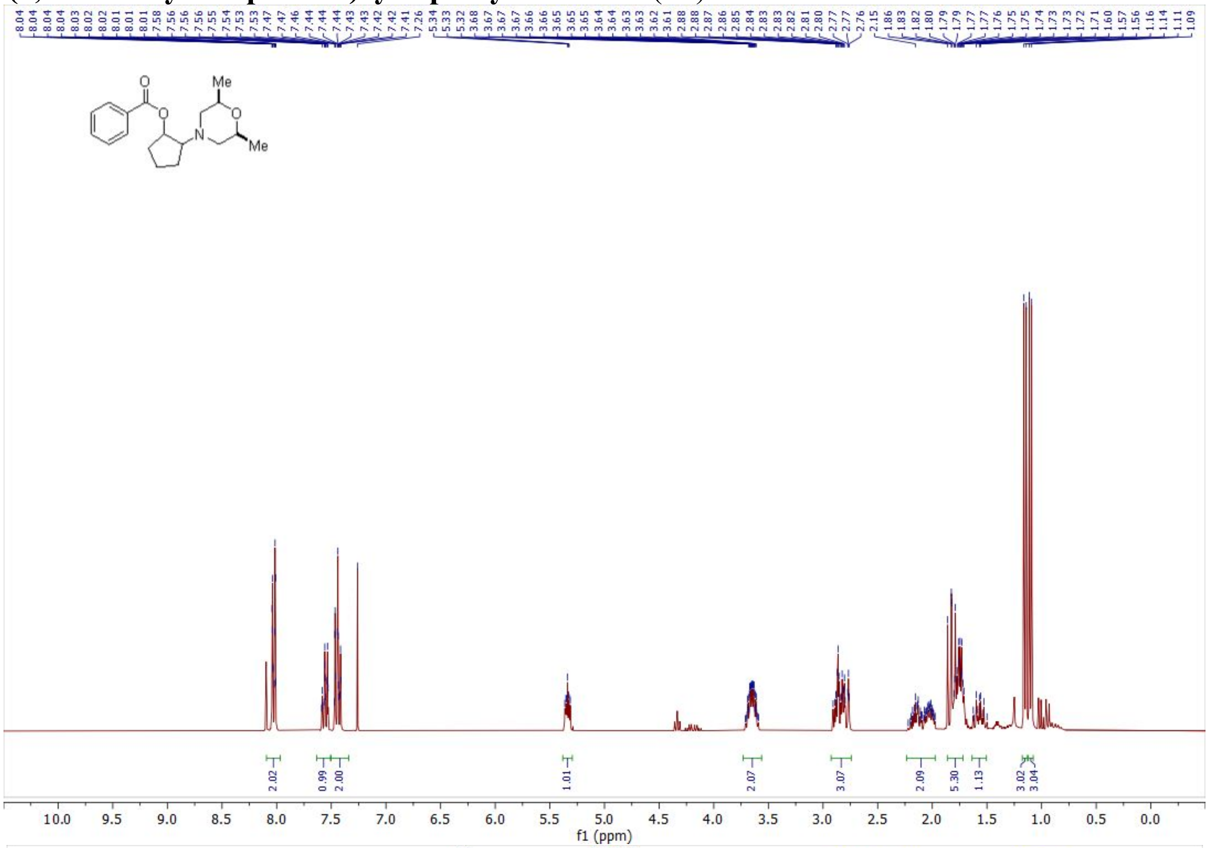

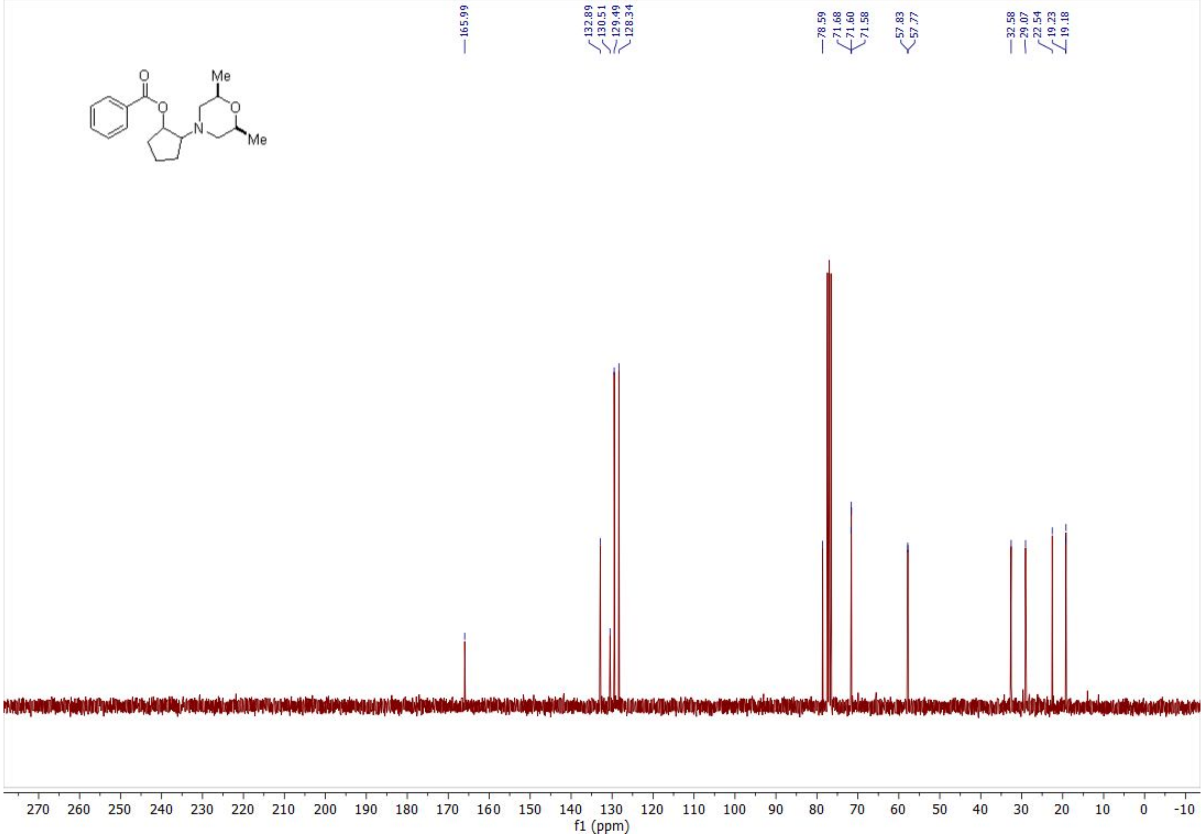

4-(methoxycarbonyl)-2-morpholinocyclopentyl benzoate (8c)

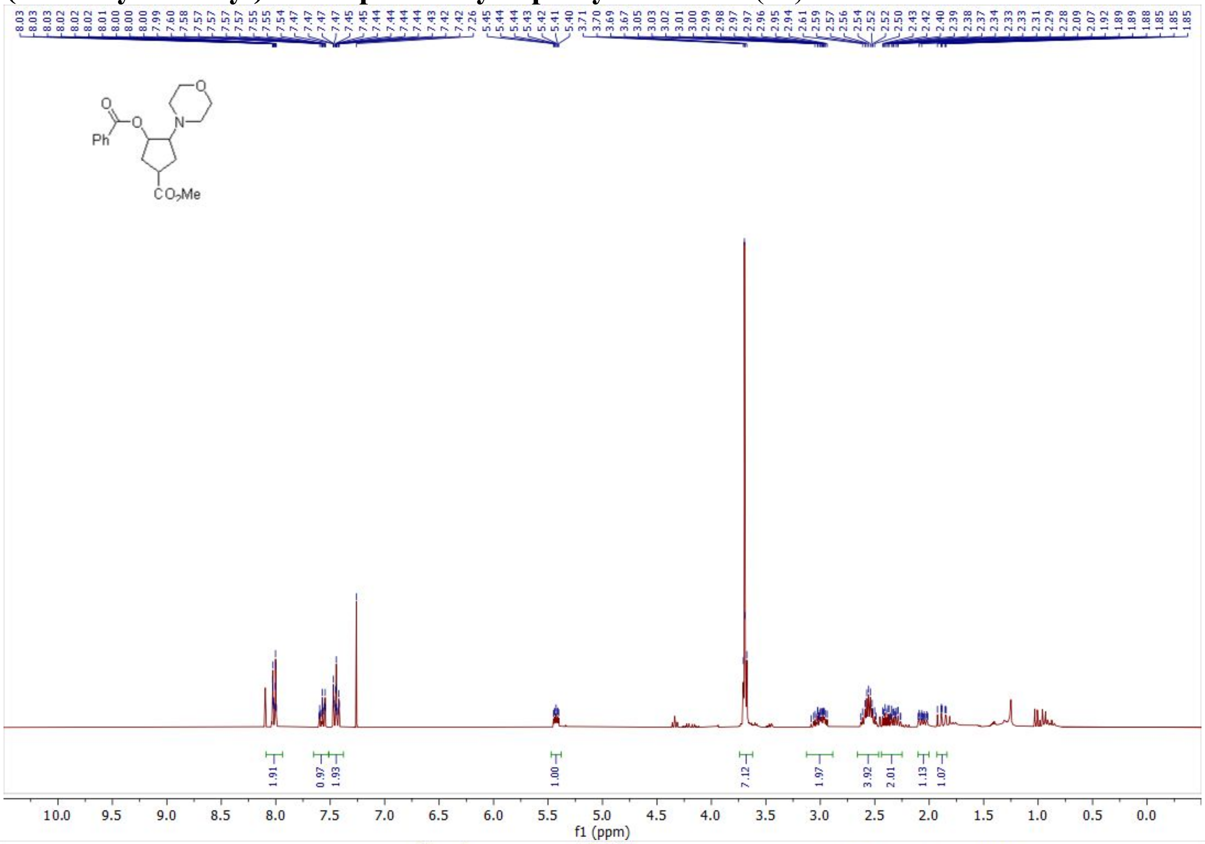

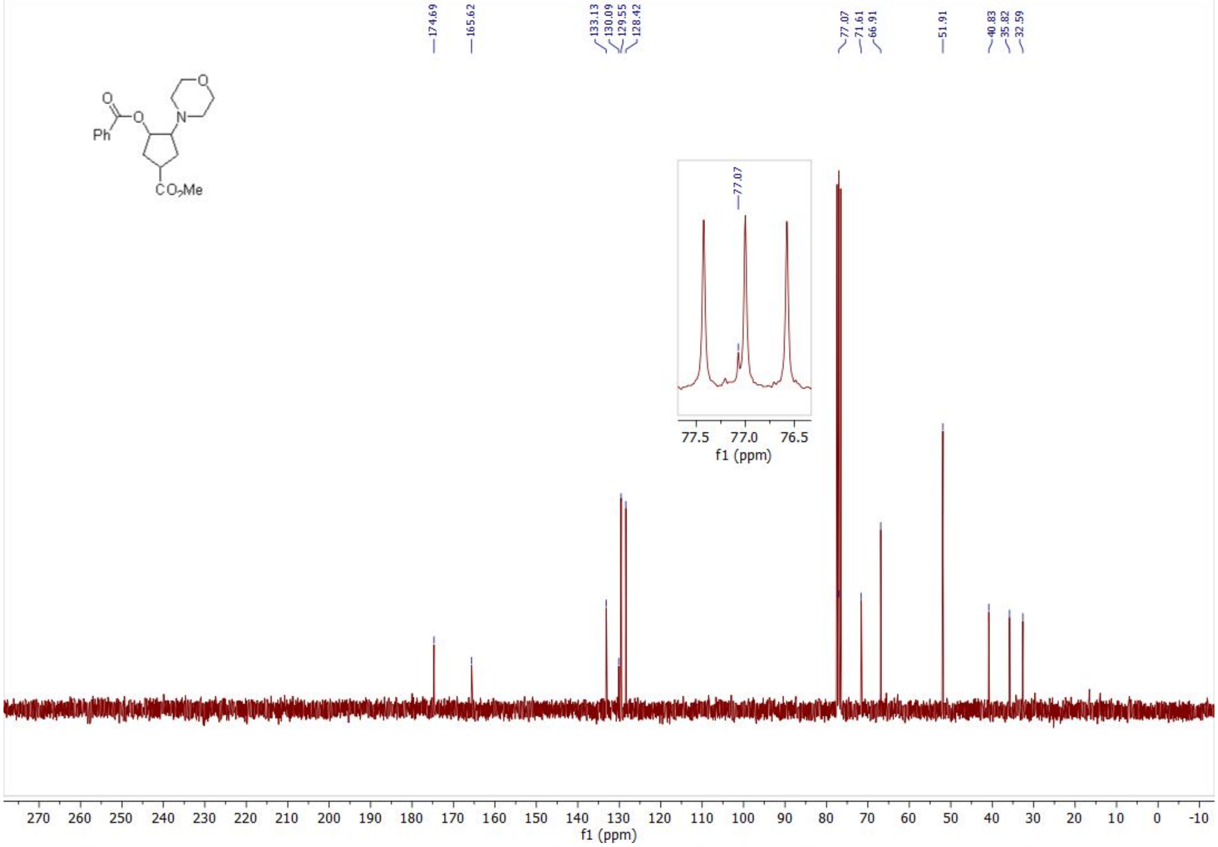

diethyl 3-(benzoyloxy)-4-morpholinocyclopentane-1,1-dicarboxylate (8d)

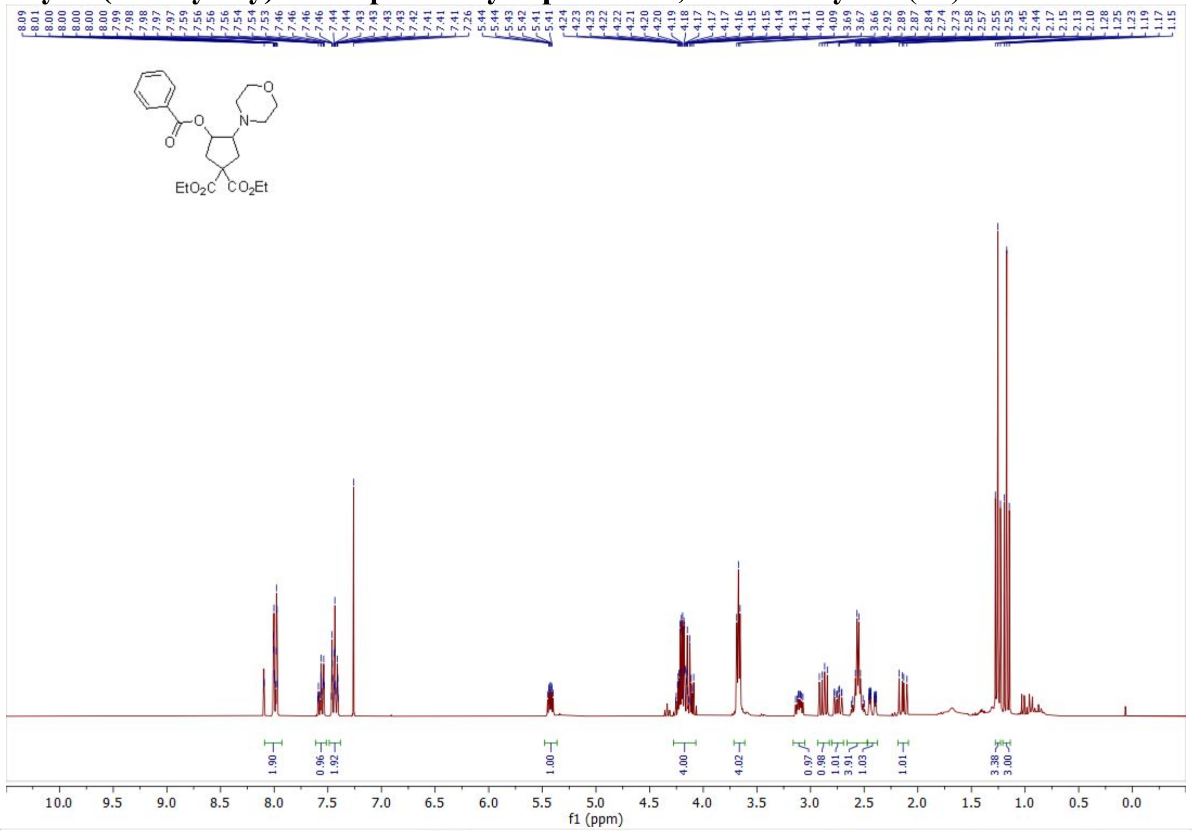

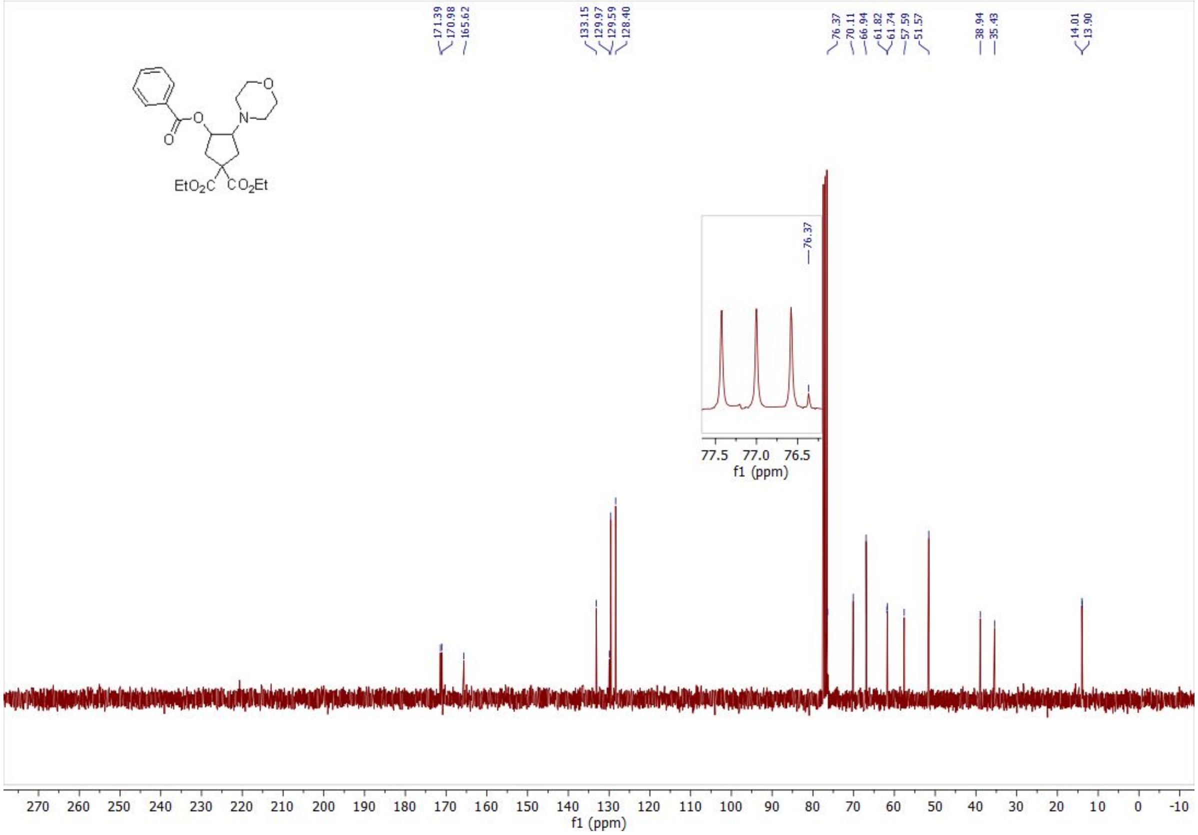

2-morpholinocyclohexyl benzoate (8e)

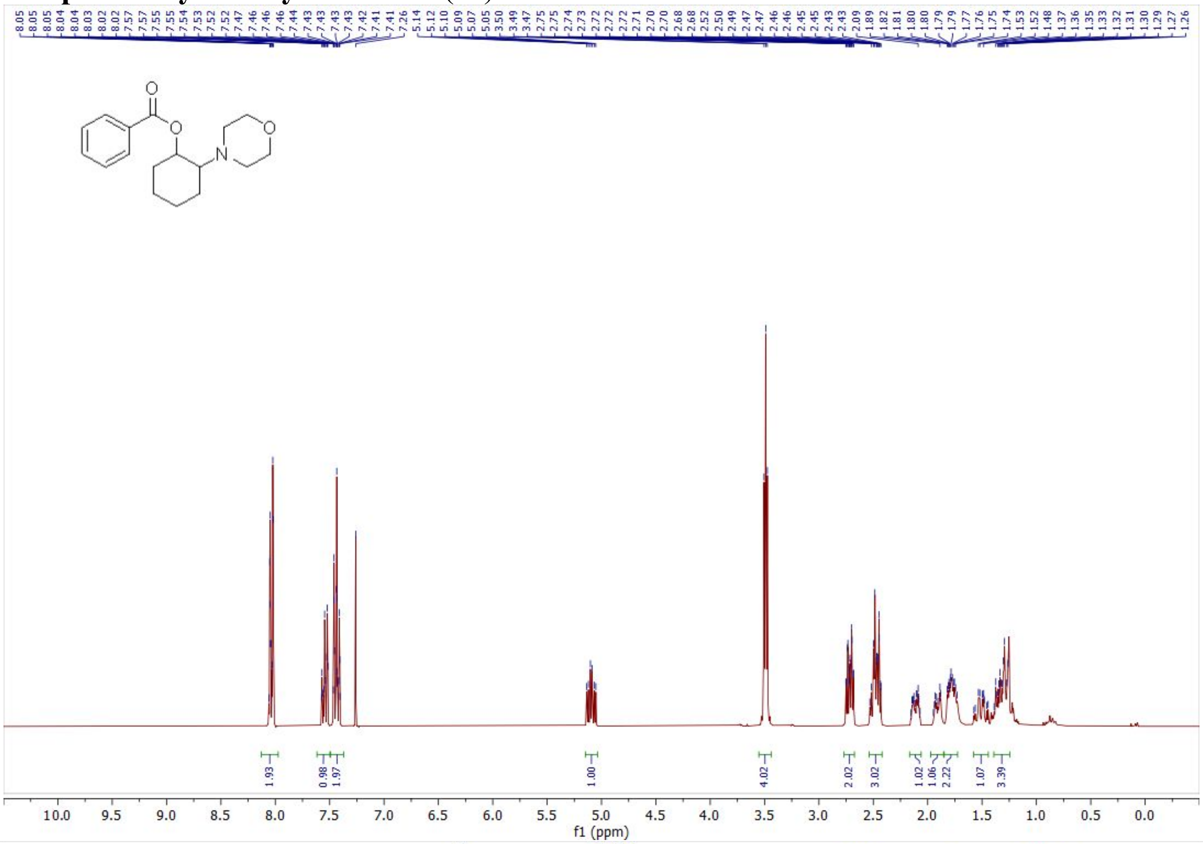

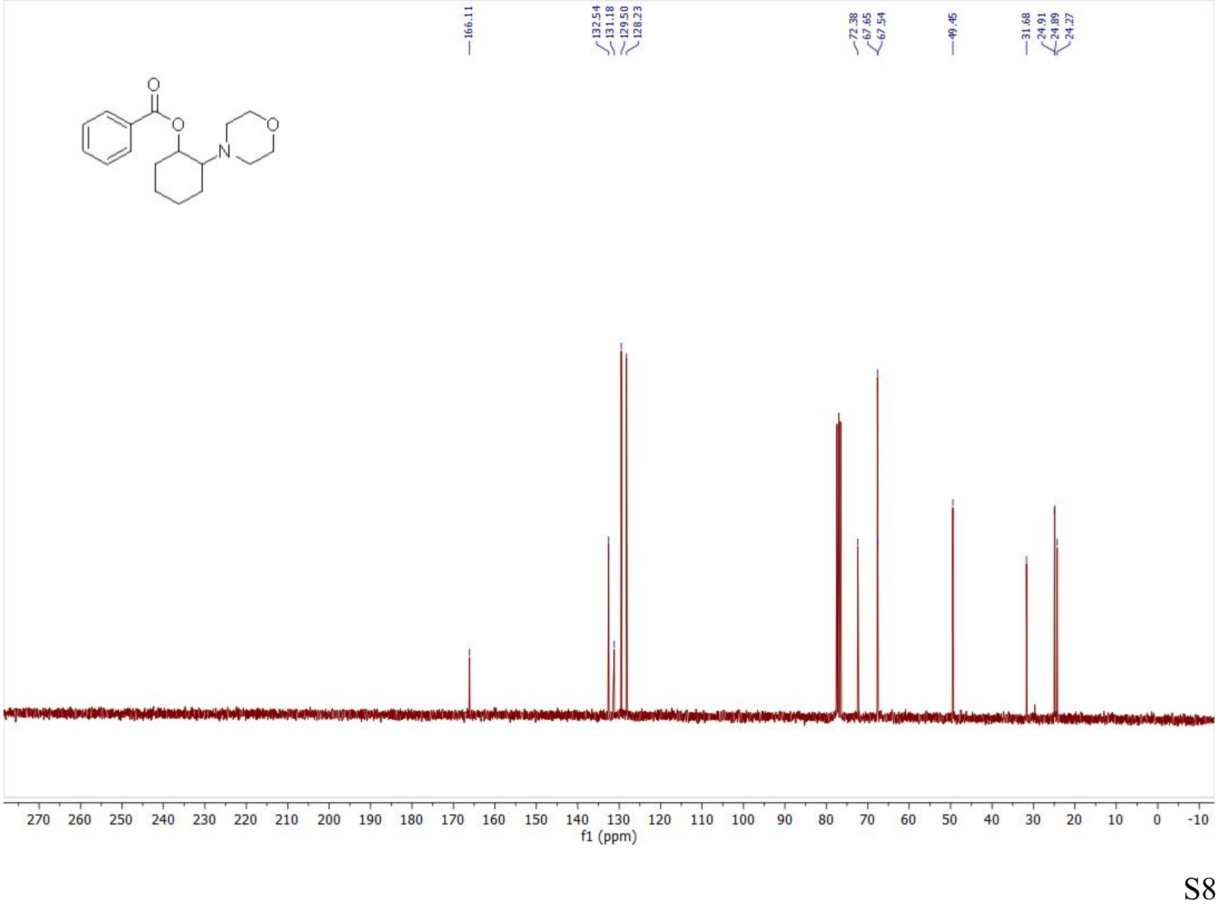

2-morpholinodecyl benzoate (8f-1) and **1-morpholinodecan-2-yl benzoate (8f-2)**

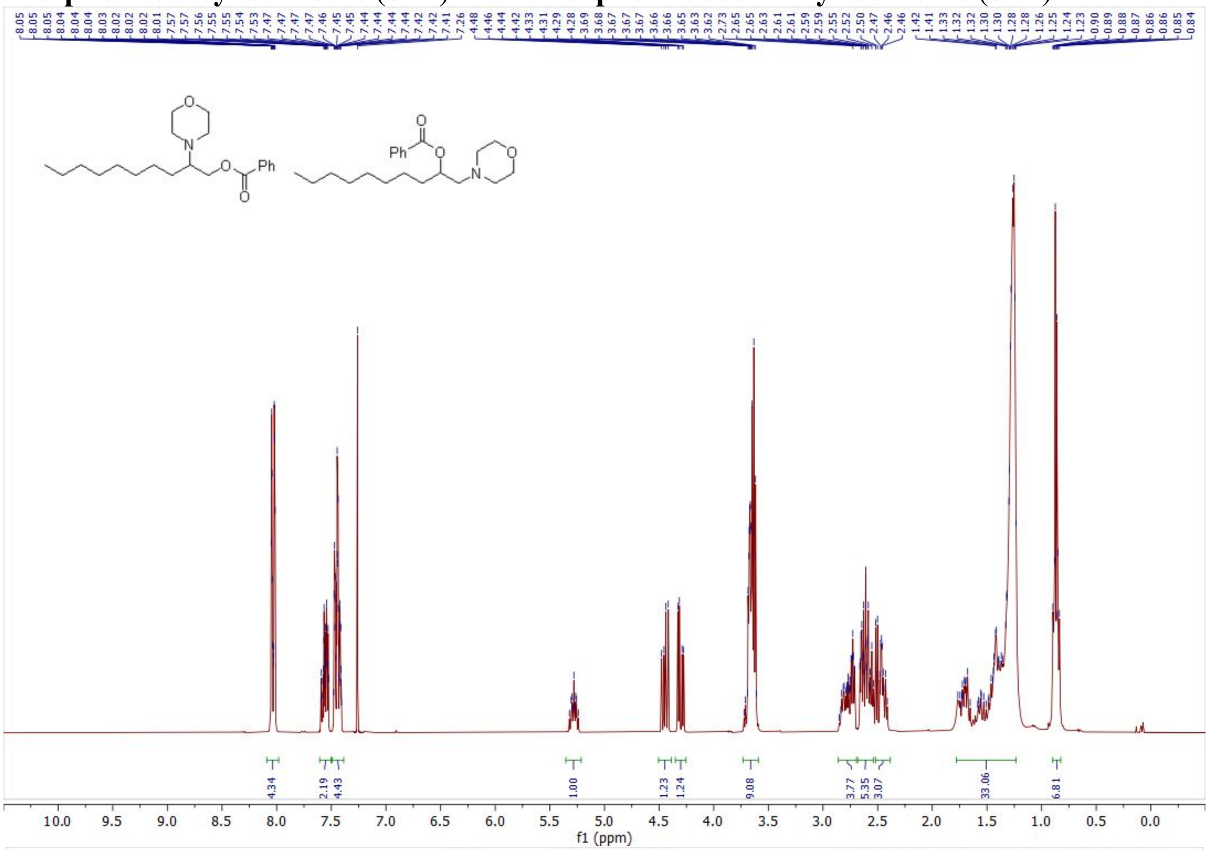

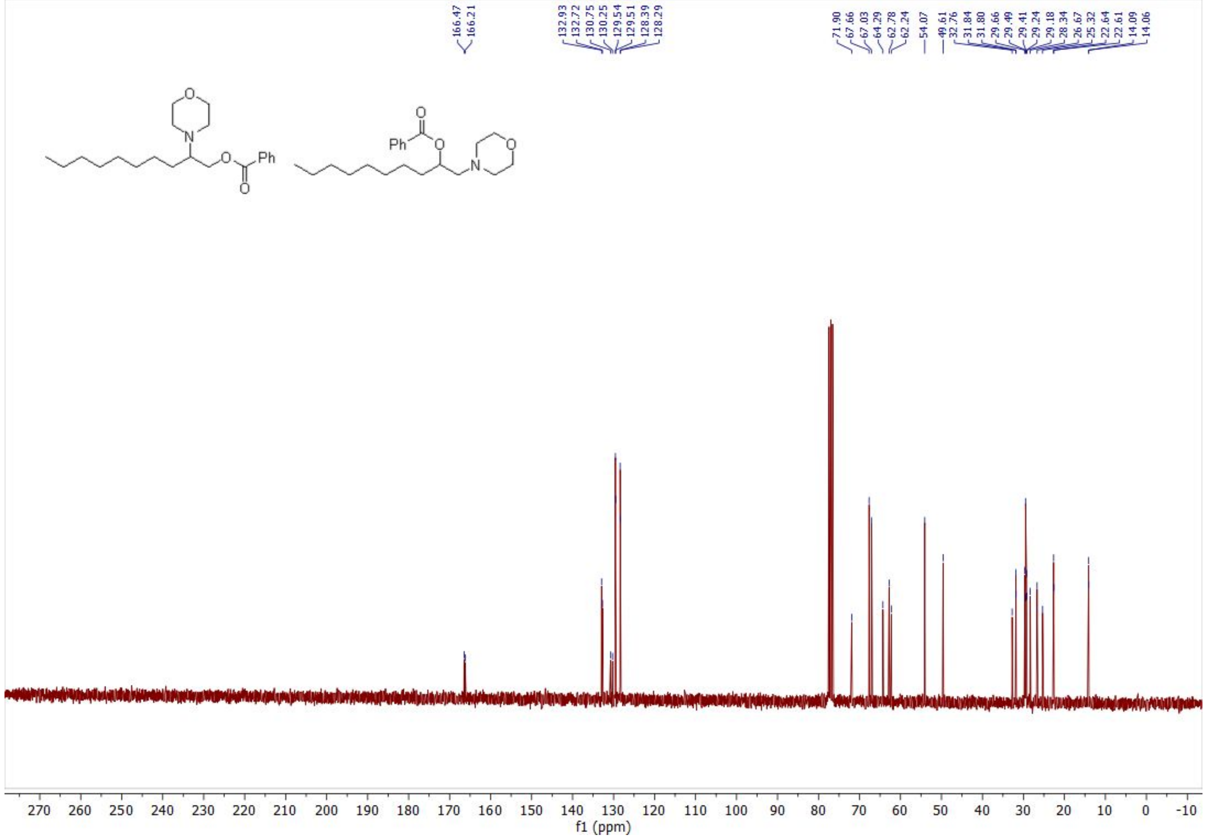

3-cyclohexyl-2-morpholinopropyl benzoate (**8g-1**) and **1-cyclohexyl-3-morpholinopropan-2-yl benzoate** (**8g-2**)

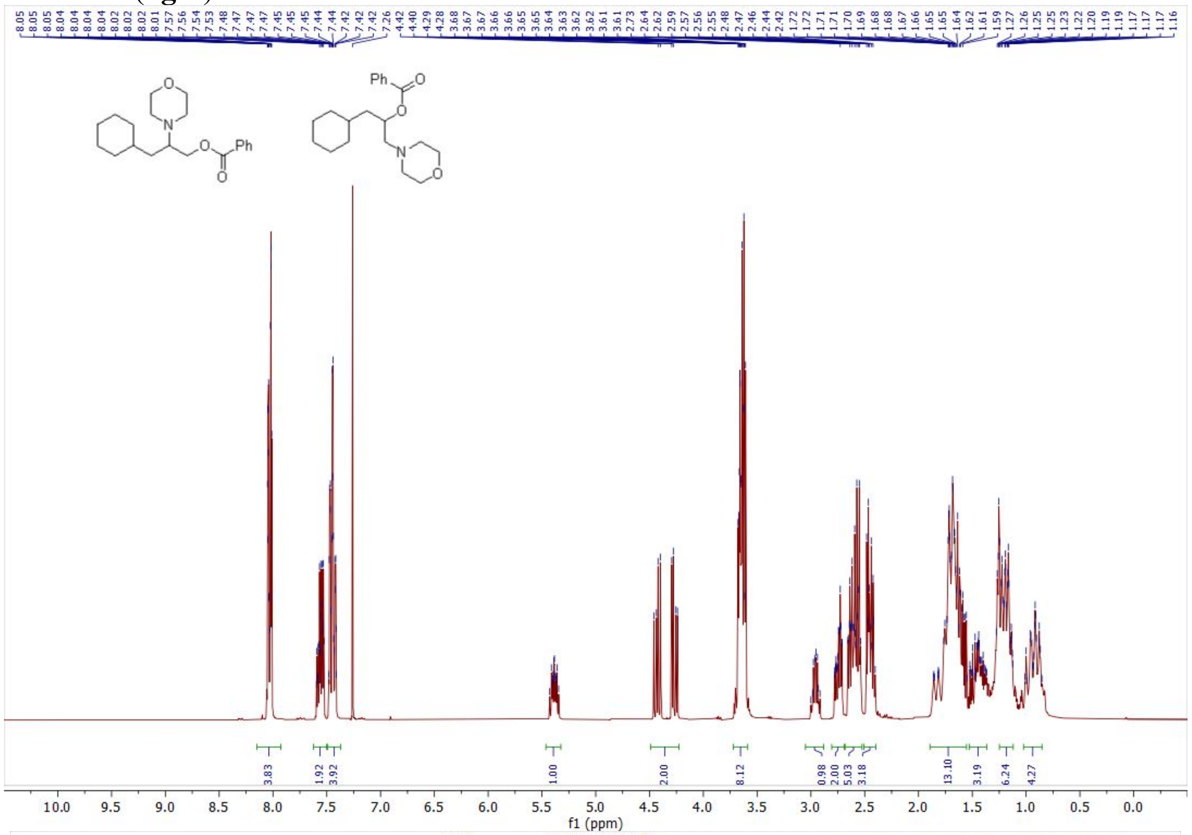

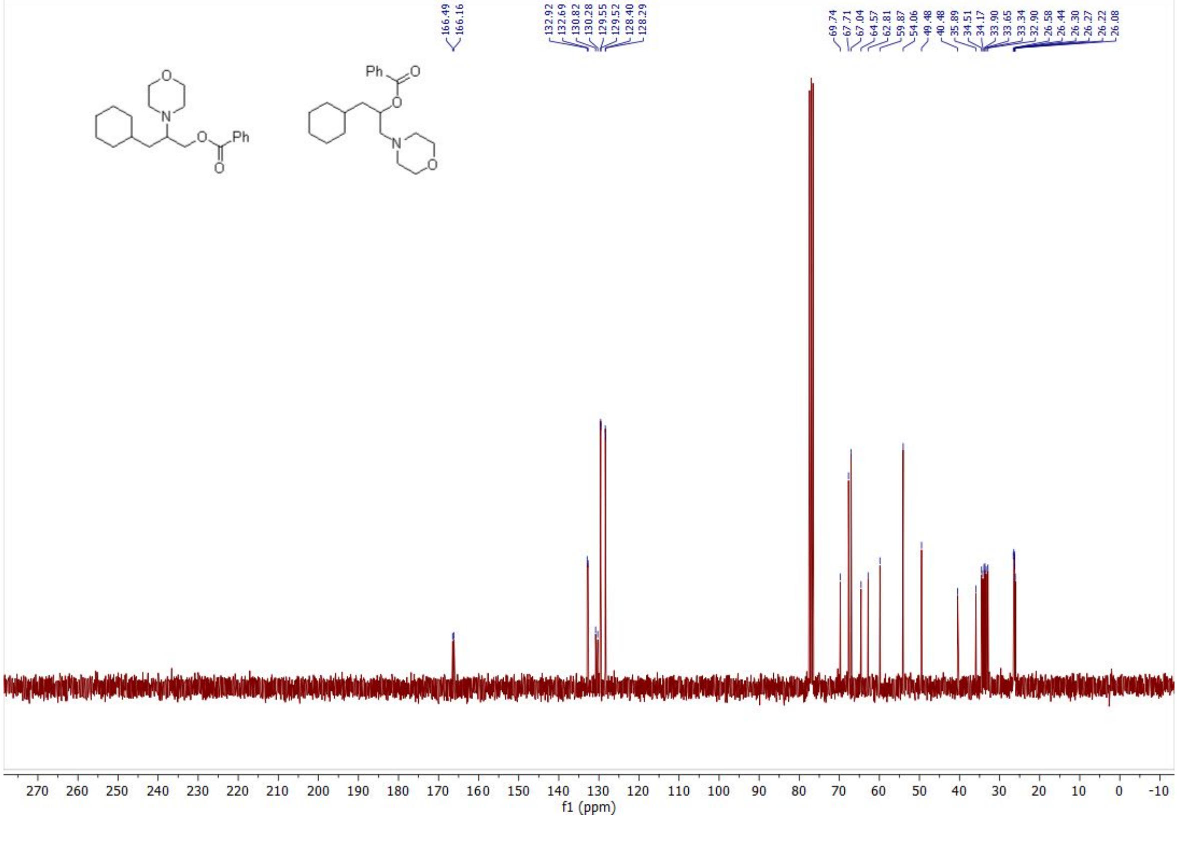

2,6-di-*tert*-butyl-4-methyl-4-(1-(piperidin-1-yl)octan-2-yl)cyclohexa-2,5-dien-1-one (5)

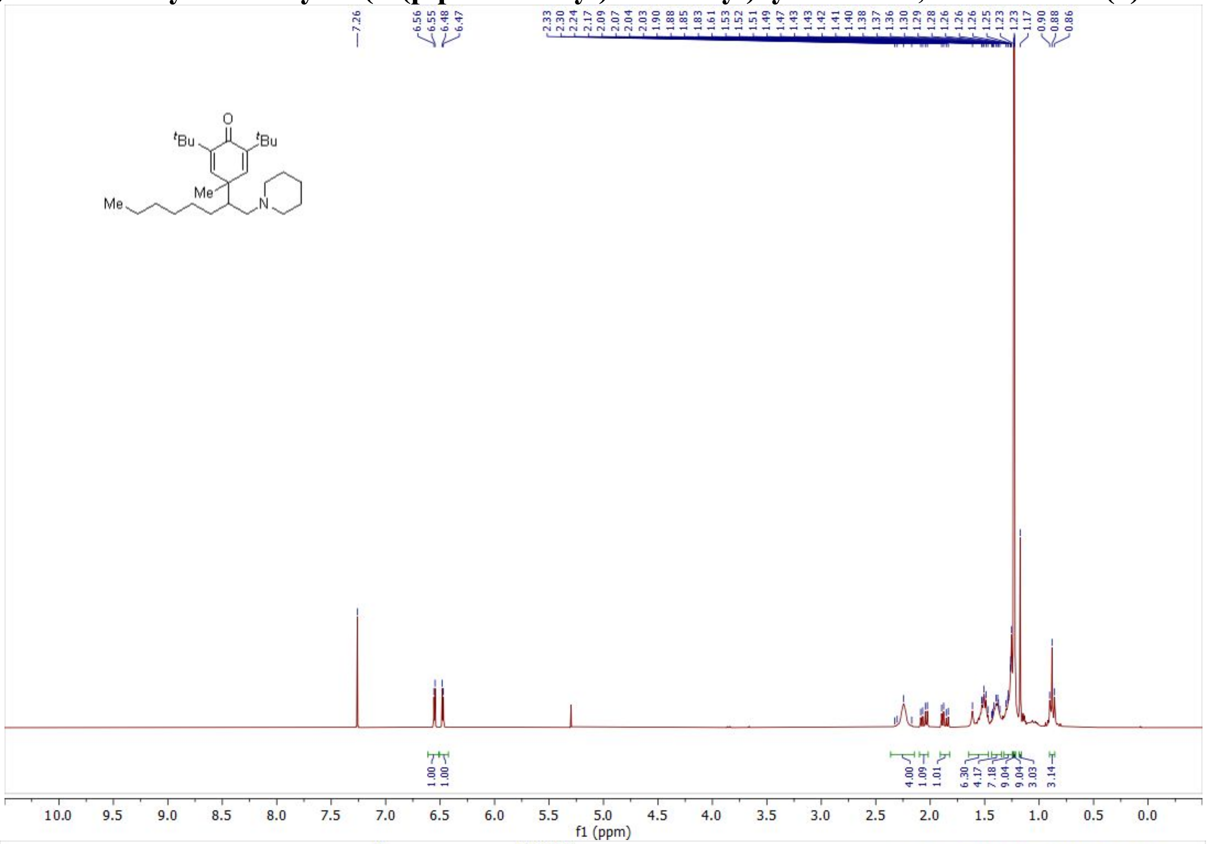

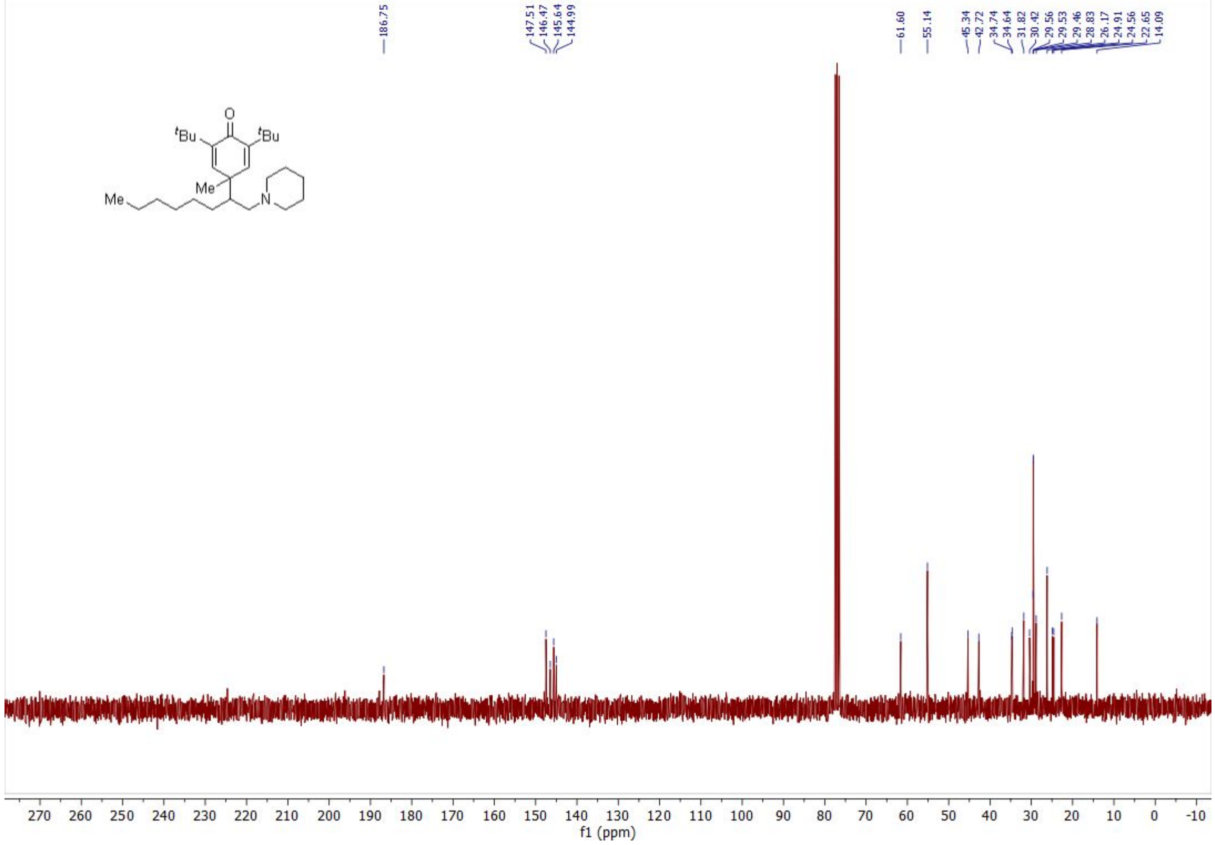

2,6-di-tert-butyl-4-(piperidin-1-ylmethyl)phenol (6)

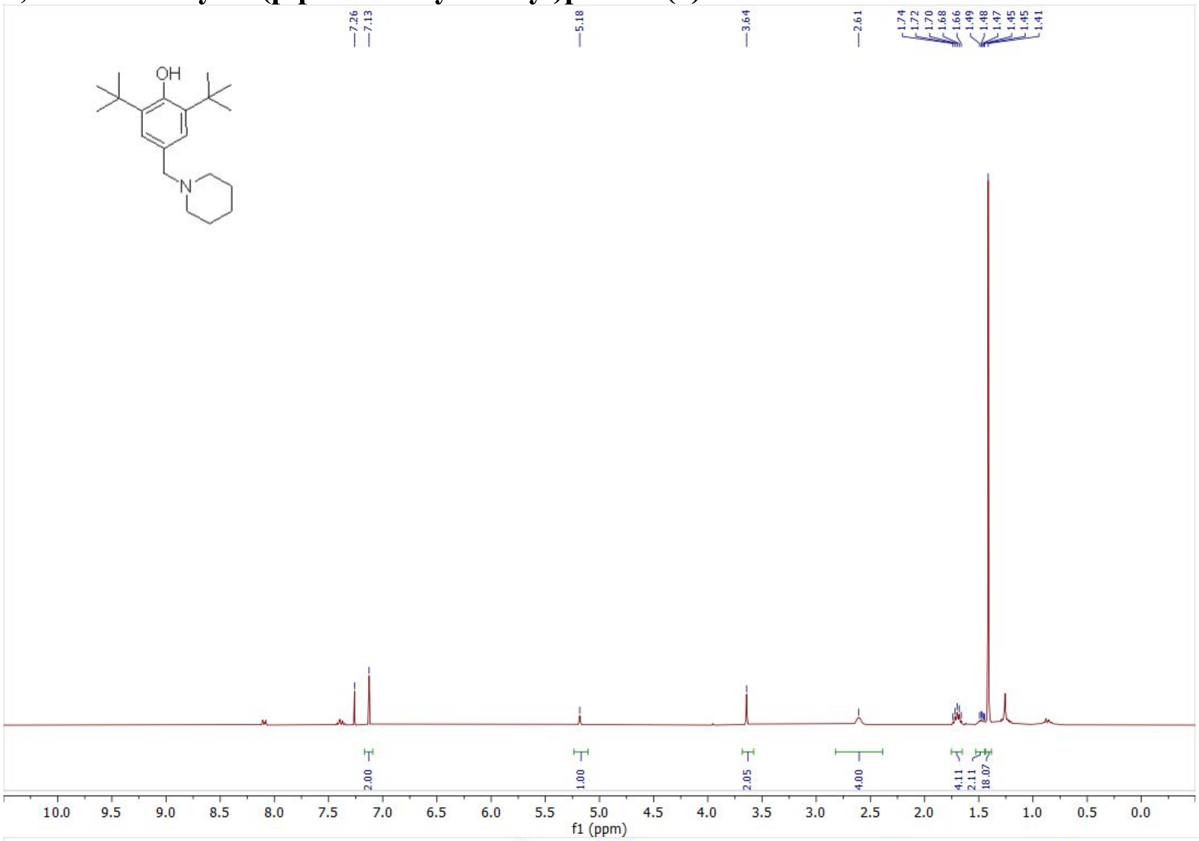

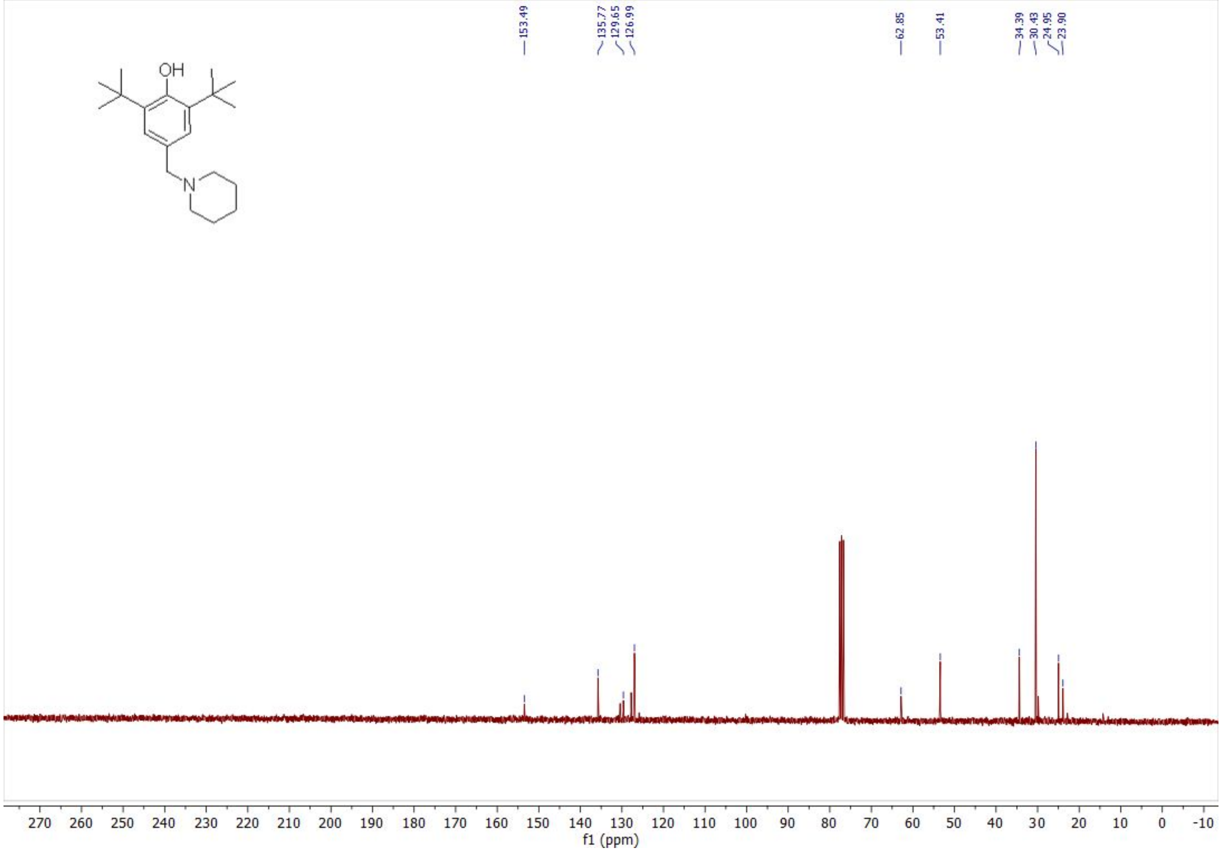

diethyl 3-(2-methoxy-2-oxoethyl)-4-(piperidin-1-ylmethyl)cyclopentane-1,1-dicarboxylate (3ww) and **diethyl 3-methyl-4-(piperidin-1-ylmethyl)cyclopentane-1,1-dicarboxylate (7)**

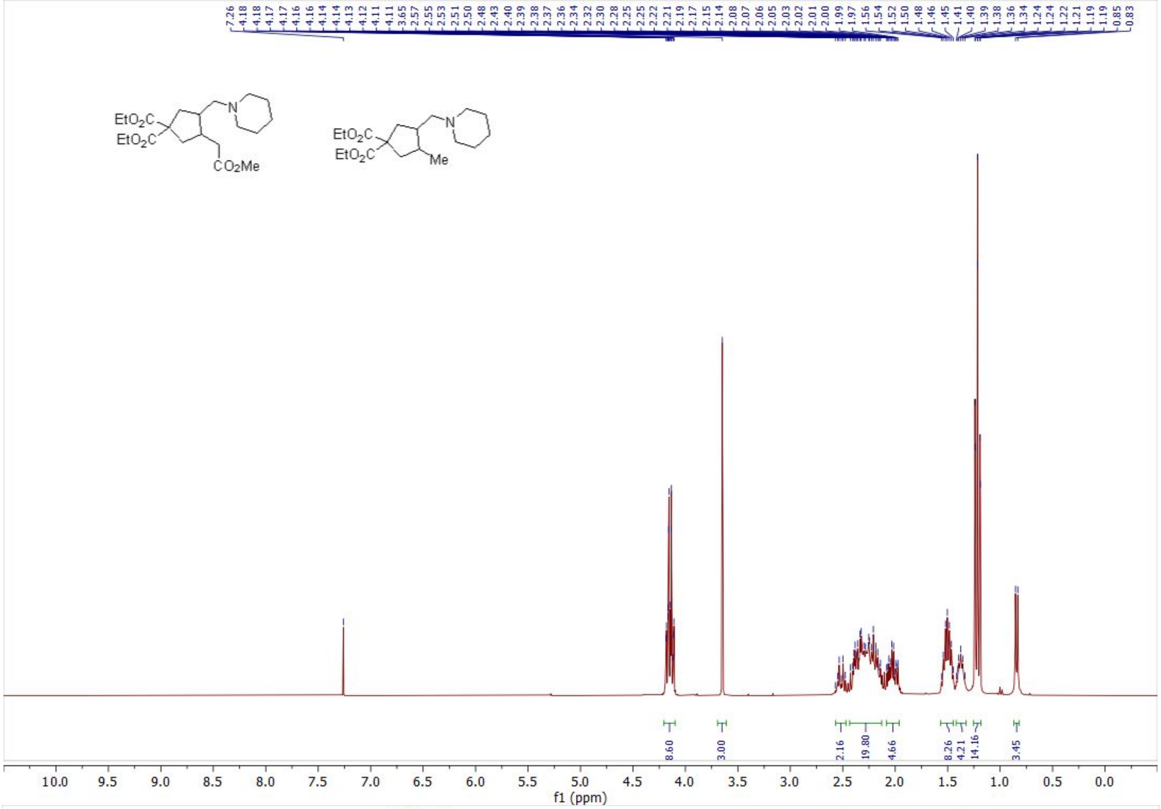

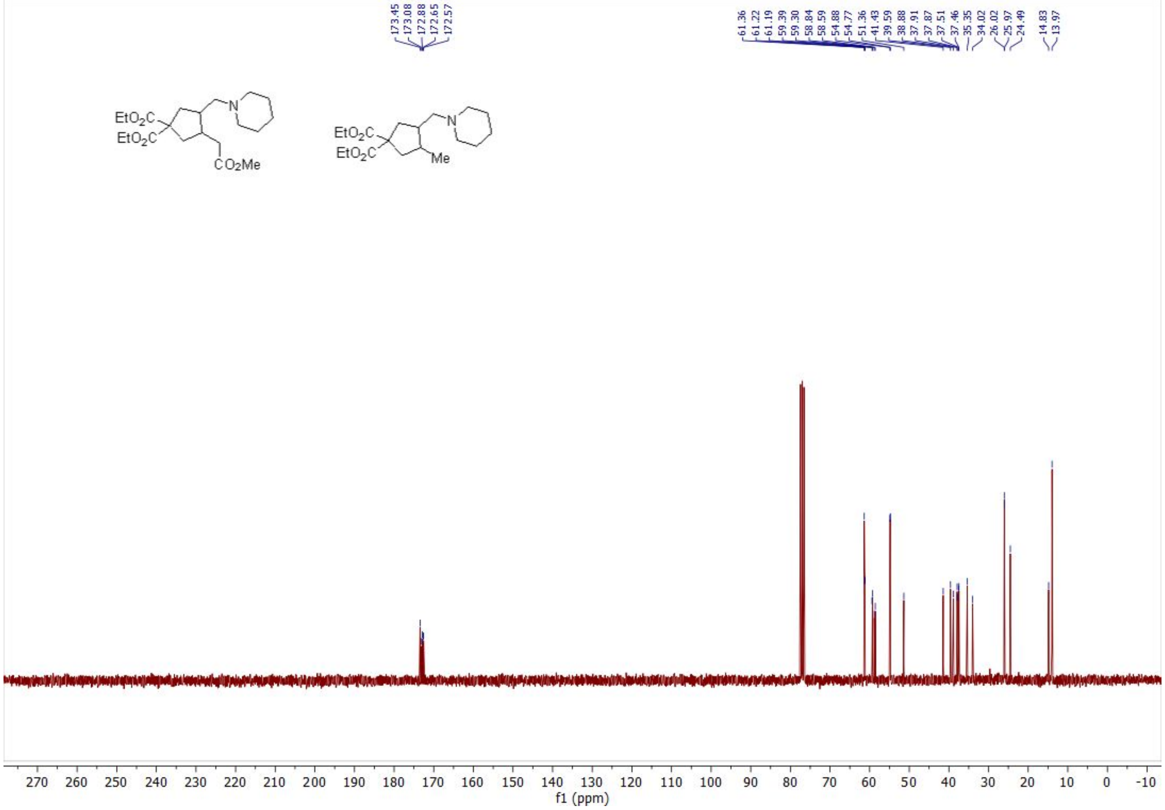

Supplement: Supplementary file 1 — ja4c13723_si_001.pdf [file ja4c13723_si_001.pdf]
